# Micronutrient Supplementation and Fortification Interventions on Health and Development Outcomes among Children Under-Five in Low- and Middle-Income Countries: A Systematic Review and Meta-Analysis

**DOI:** 10.3390/nu12020289

**Published:** 2020-01-21

**Authors:** Emily Tam, Emily C. Keats, Fahad Rind, Jai K. Das, Zulfiqar A. Bhutta

**Affiliations:** 1 Centre for Global Child Health, Hospital for Sick Children, Toronto, ON M5G 0A4, Canada; emilytam7@gmail.com (E.T.); emily.keats@sickkids.ca (E.C.K.); 2 Centre of Excellence in Women and Child’s Health, Aga Khan University, Karachi 74800, Pakistan; dr.fahadrind@gmail.com; 3 Division of Women and Child Health, Aga Khan University, Karachi 74800, Pakistan; jai.das@aku.edu

**Keywords:** micronutrient, iron, vitamin A, zinc, lipid-based nutrient supplement, fortification, under-five, efficacy, effectiveness

## Abstract

Micronutrient deficiencies continue to be widespread among children under-five in low- and middle-income countries (LMICs), despite the fact that several effective strategies now exist to prevent them. This kind of malnutrition can have several immediate and long-term consequences, including stunted growth, a higher risk of acquiring infections, and poor development outcomes, all of which may lead to a child not achieving his or her full potential. This review systematically synthesizes the available evidence on the strategies used to prevent micronutrient malnutrition among children under-five in LMICs, including single and multiple micronutrient (MMN) supplementation, lipid-based nutrient supplementation (LNS), targeted and large-scale fortification, and point-of-use-fortification with micronutrient powders (MNPs). We searched relevant databases and grey literature, retrieving 35,924 papers. After application of eligibility criteria, we included 197 unique studies. Of note, we examined the efficacy and effectiveness of interventions. We found that certain outcomes, such as anemia, responded to several intervention types. The risk of anemia was reduced with iron alone, iron-folic acid, MMN supplementation, MNPs, targeted fortification, and large-scale fortification. Stunting and underweight, however, were improved only among children who were provided with LNS, though MMN supplementation also slightly increased length-for-age z-scores. Vitamin A supplementation likely reduced all-cause mortality, while zinc supplementation decreased the incidence of diarrhea. Importantly, many effects of LNS and MNPs held when pooling data from effectiveness studies. Taken together, this evidence further supports the importance of these strategies for reducing the burden of micronutrient malnutrition in children. Population and context should be considered when selecting one or more appropriate interventions for programming.

## 1. Introduction

### 1.1. Background

Micronutrients (vitamins and minerals) are an essential component of the diet and are necessary for normal cellular and molecular function [1]. While micronutrients are only needed in trace amounts, their deficiency can result in wide-ranging negative health effects. Micronutrient deficiencies are especially a concern in low- and middle-income countries (LMICs), owing to inadequate consumption of food, a lack of dietary diversity, and poor absorption of nutrients due to infection, inflammation, and chronic illness [1]. Concurrent deficiencies are also relatively common. Children under-five are particularly vulnerable, as rapid growth and development necessitates a higher demand for micronutrients [1]. Worldwide, an estimated 43% of children under-five have anemia [2], 29% of children under-five in LMICs are deficient in vitamin A [3], 30% of school-aged children have insufficient iodine intake [4], and 17% of the population are deficient in zinc [5], despite a considerable degree of uncertainty in these estimates. Assessing micronutrient status in children under-five is challenging, especially in LMICs, due to a lack of resources, inconsistent definitions and tools for measuring nutritional status, and inappropriate aggregation of country level data.

Micronutrient deficiencies are associated with undesirable short- and long-term effects, including physical, developmental, and cognitive impairment, increased susceptibility to infections, higher morbidity and mortality, and decreased productivity later in life [6,7,8,9]. For example, iron-deficiency anemia in infancy and early childhood has been associated with poor motor development and irreversible cognitive defects, which impair learning and decrease educational attainment [7,10]. Iodine deficiency in childhood has also been shown to be a risk factor for developmental delay [11]. Vitamin A deficiency increases the risk of blindness in children and death from common conditions, such as diarrhea and measles [12]. Finally, zinc deficiency has been linked to impaired growth and depressed immune function, resulting in stunting, wasting, and more severe infections [13,14]. Collectively, it is estimated that undernutrition, including micronutrients deficiencies, stunting, and wasting, causes approximately 45% of deaths in all children or 3.1 million deaths annually [15].

### 1.2. Current Strategies and Interventions

Several strategies exist to address the problem of micronutrient malnutrition in children under-five in LMICs. These include micronutrient supplementation, lipid-based nutrient supplementation (LNS), large-scale fortification, targeted fortification, and point-of-use fortification with micronutrient powders (MNPs). Fortification is the recommended long-term strategy for increasing the dietary intake of certain micronutrients in the general population, and may be particularly successful if mandated by the government with support from the food industry [1]. However, as safety regulations limit the amount of added micronutrients to that below the tolerable upper intake level [16], individuals who consume small quantities of food, such as young children, may not achieve sufficient micronutrient intake from fortified foods. By contrast, supplementation is an effective short-term solution for preventing and addressing micronutrient deficiencies in specific at-risk groups [1]. An advantage of supplementation and MNPs is that a single dose or serving satisfies daily micronutrient needs.

Micronutrient supplementation involves the provision of a single micronutrient (iodine, iron, folic acid, vitamin A, vitamin B12, vitamin D, zinc) or multiple micronutrients in the form of capsules, tablets, drops, or syrup. Multiple micronutrient (MMN) supplements are defined as a single administration of three or more different micronutrients [17]. LNS are products containing micronutrients embedded in a food base that provides energy, essential fatty acids, and protein, and are usually given in an amount of 20–50 g/day [18]. Small-quantity LNS (SQ-LNS), such as NutriButter (20 g/day), which provides 110–150 kcal/day, are intended for use as home fortificants in LMICs to overcome nutrient deficiencies in the local diet [18]. Large-scale fortification is the process of adding micronutrients to commonly consumed foods (i.e., staple foods and condiments) during central processing to increase their nutritional value. Wheat, flour, rice, salt, sugar, oil, and milk are often selected as vehicles for large-scale fortification because of their widespread consumption in target populations. Targeted fortification is the practice of adding micronutrients to foods designed for specific subgroups of the population, such as infant formula for infants less than 6 months of age, complementary foods for children over 6 months of age, and foods for institutional programs aimed at school- and preschool-aged children [16]. Targeted fortification does not include complementary foods fortified at the household level. Finally, MNPs are used to add micronutrients to foods at point-of-use, usually when preparing meals in the home or in schools. MNPs are packaged as single-serving sachets containing a dry mixture of micronutrients that can be directly added onto soft or semi-solid foods ready for consumption [19].

### 1.3. Importance of This Review

Many systematic reviews have been performed to summarize the abundant primary research on micronutrient interventions in children under-five in LMICs [12,13,14,20,21,22,23,24,25,26,27,28,29,30,31,32,33,34,35]. However, we identified several major gaps in the literature. The most critical among these is that the effects of individual micronutrient interventions remain inconclusive, owing to the inclusion of a limited number of trials that are typically individually underpowered. Several large-scale randomized controlled trials have been recently completed, and the inclusion of these studies in the evidence base will enable a larger sample from which to draw robust conclusions. Second, some outcomes have not been studied or remain inadequately studied. For example, the effects of vitamin A supplementation on morbidity and mortality, but not child growth, have been studied in a recent Cochrane review [12]. Third, there remains a lack of effectiveness data to inform real-life benefits of micronutrient intervention programs. The inclusion of such data, as well as understanding of an intervention’s biological potential through synthesis of trial efficacy data, will better guide policy making and inform recommendations of the interventions that are likely to be successful in uncontrolled environments. Therefore, the aim of this review was to comprehensively summarize the available evidence from trials and programs on common micronutrient interventions in children under-five in LMICs. The main objectives were to examine the efficacy and effectiveness of five types of interventions on child health and nutritional status: single and MMN supplementation, LNS, targeted fortification, large-scale food fortification, and point-of-use fortification with MNPs.

## 2. Materials and Methods

### 2.1. Search Strategy

The protocol has been previously published [36] and was conducted in accordance with PRISMA guidelines. Relevant papers were identified by searching the following electronic databases: African Index Medicus, CAB Abstracts, CINAHL, Cochrane Central Register of Controlled Trials (CENTRAL), Embase, International Initiative for Impact Evaluations (3ie), LILACS, MEDLINE and WHO (eLENA). We also searched clinicaltrials.gov, ProQuest Dissertations and Theses Global, and the WHO International Clinical Trials Registry Platform for relevant non-published studies. We restricted papers to those in English, French, Spanish, or Persian, based on feasibility of translation, and those that had been published on or after 1995. Additional searches for relevant program evaluations and trials were conducted in Google, Google Scholar, and websites of key international nutrition agencies including UNICEF, GAIN, Emergency Nutrition Network, IZiNCG, World Food Program, Sight and Life, Nutrition International, Hellen Keller International, and the Iodine Global Network. The reference lists of reviews and included studies were manually searched to identify any missed papers. Searches were initially completed in June 2018 and were updated on 29 October 2019. The MEDLINE search strategy can be found in Text S1.

### 2.2. Study Selection and Eligibility Criteria

Studies were eligible for inclusion if they (i) were a randomized or non-randomized controlled trial, controlled before-after study, or interrupted time series study in which outcomes were assessed at a minimum of three times before and after the implementation of the intervention, (ii) were conducted in healthy children 1 month to 5 years of age living in an LMIC, (iii) compared the effect of a micronutrient supplementation intervention with that of an inactive (i.e., placebo, no intervention, or standard of care) or active control intervention (i.e., different composition of micronutrients or comparison of MMN supplementation with LNS), and (iv) examined one of the following primary or secondary outcomes. Primary outcomes include all-cause mortality, cause-specific mortality (diarrhea, lower respiratory tract infection including pneumonia, malaria, measles, meningitis, and other), and nutritional status (anemia, stunting, wasting, and underweight). Secondary outcomes include morbidity (diarrhea and lower respiratory tract infection), micronutrient concentrations/deficiencies (folate, iron, hemoglobin concentration, vitamin A, vitamin D, and zinc), growth (height, weight, length-for-age, weight-for-age, weight-for-height, head circumference, mid-upper arm circumference, and body mass index), mental and motor development, and adverse effects (bulging fontanelle, fever, gastrointestinal, headache, irritability, kidney stones, stained teeth, and other).

The micronutrient supplementation may be a single micronutrient (folic acid, iodine, iron, vitamin A, vitamin D, vitamin B12, or zinc) or MMN supplementation (≥3 micronutrients), iron-folic acid supplementation, or LNS, administered by any route and at any frequency in the form of tablets, capsules, drops, syrups, or foodlets. We also considered large-scale food fortification of staple foods or condiments, targeted fortification of formula (for infants under 6 months of age) or complementary food (for children 6 months of age and older), and point-of-use fortification with MNPs. LMICs were defined using the classification by the World Bank Group in the year of the study initiation. Studies in children outside the 1 month to 5 years age range were eligible if it was possible to isolate data for participants within the specified age range or if the age of children at baseline (mean or median) was within that range. Studies with co-interventions (e.g., anthelmintics or nutrition education) were eligible only if identical co-interventions were given to the intervention and comparison group. For trials with relevant interventions in multiple arms (e.g., varying formulations or dosages), we included only the intervention–control comparison that was most similar to other studies of that intervention type. However, for studies in which it was difficult to determine the most similar intervention arm, we combined the relevant groups into a single pairwise comparison. Apart from interrupted time-series studies, for which a comparator control group was not necessary, studies must meet all four criteria to be eligible for inclusion. We excluded single-arm trials with no control groups, studies in which the intervention was administered to the mother before or during pregnancy rather than to the infant or child, studies in disease-specific populations, and studies on the effect of supplementation for the purpose of treatment of micronutrient deficiency rather than for prevention.

Titles, abstracts, and full texts were screened for inclusion using the specified inclusion/exclusion criteria by two independent reviewers using an online systematic review platform (Covidence). After removal of duplicates, titles were screened independently, and if the title provided insufficient information, then the abstract was screened to determine eligibility for full text screening. Disagreements at the full text screening stage were resolved by consensus of the two reviewers or independently by a third reviewer.

### 2.3. Data Collection and Measurement

Using a piloted data collection sheet, two reviewers independently extracted information from eligible studies. Data extracted included general study characteristics (study setting, study population, study design, duration of data collection, study attrition), details on the intervention (type, duration, frequency, dosage, formulation, level of implementation, food vehicle) and control, funding sources, information on quality assessment, and quantitative outcomes (subgroups, subgroup sample sizes, outcome measures, effect estimates, and measures of uncertainty). For program evaluations, we also collected information on indicators of activity (policies, production and supply, delivery systems, quality control, and behavior change communication) and output (access and coverage, and knowledge and appropriate use). When necessary, data for each outcome were converted into a consistent format (e.g., means and standard deviations) with the same units, such that the direction of effect always corresponded to an increase or decrease in the measure. We extracted both final (post-intervention) measurements and change from baseline scores.

To avoid double counting of participants, studies were grouped by setting, population, intervention type, and program (if applicable). Studies that included participants from the same population were combined and coded as a single study.

### 2.4. Risk of Bias and Quality Assessment

Two independent reviewers used the Cochrane Risk of Bias Tool and the Cochrane Effective Practice and Organization of Care (EPOC) guidelines to assess risk of bias of eligible studies as high, low, or unclear for each criterion [37,38]. Randomized controlled trials were assessed on the following: random sequence generation, concealment of allocation, blinding of participants, personnel and outcome assessors, selective reporting, and other sources of bias. We also considered the following criteria for non-randomized controlled trials and controlled before–after studies: similarity of baseline characteristics, similarity of baseline outcome characteristics, adequate prevention of knowledge of allocated interventions, and adequate protection against contamination. Interrupted time-series studies were assessed on the following: intervention independent of other changes, shape of intervention effect pre-specified, intervention unlikely to affect data collection, adequate prevention of knowledge of the allocated interventions, incomplete outcome data, selective outcome reporting, and other sources of bias. All disagreements were resolved by a third reviewer.

The GRADE tool was used to assess the quality of evidence for each primary and secondary outcome on five criteria: study limitations, consistency of effect, imprecision, indirectness, and publication bias. Quality of evidence may be upgraded if there was a large magnitude of effect, a dose response gradient, or an effect of residual confounding. Quality of evidence may be downgraded if there was a high risk of bias in individual studies, indirectness of evidence, inconsistent results across studies, imprecision of results, or publication bias.

### 2.5. Statistical Analyses

Statistical analyses were performed using Review Manager 5.3. We performed standard meta-analyses to generate a summary risk ratio (events per child) or rate ratio (events per child year) and 95% confidence interval (CI) for each dichotomous outcome or a mean difference (MD, if studies used the same scale to assess the outcome) or a standardized mean difference (SMD, if studies used different scales to assess the outcome) and 95% CI for each continuous outcome. Final (post-intervention) measurements and change from baseline scores were pooled in meta-analyses with MDs where applicable but not with SMDs, as differences in standard deviation in the latter case do not represent differences in the measurement scale, but rather in the reliability of the measurement. For continuous outcomes, the inverse variance random effects model was used due to expected variability in interventions, settings, and methods across studies, and to weight studies by variance of the effect estimate. Effect estimates for dichotomous outcomes were calculated using the Mantel–Haenszel random effects model. However, in some cases, the inverse variance model was used if at least one study in the analysis reported effect estimates only. We pooled the most adjusted estimates (when possible). Study characteristics of studies deemed inappropriate for meta-analysis due to substantial methodological or statistical heterogeneity were summarized in a table (Table S1). We followed an intention to treat analysis for randomized controlled trials. Author-defined control groups (e.g., placebo or no intervention) were used and pooled for each outcome. If a study had multiple eligible arms that received different interventions, they were included in the meta-analyses as separate intervention–control comparisons. Findings from non-randomized controlled trials, controlled before–after studies, and interrupted time-series designs were analyzed and reported separately. Clustered randomized effectiveness trials were pooled with both efficacy and effectiveness studies. Outcomes with data reported by fewer than 3 studies were not meta-analyzed. Studies that incorporated a cluster randomized design were adjusted for clustering by reducing the size of the trial to its effective sample size or by inflating the standard error. Adjustments were not made if authors had adjusted for clustering. Statistical heterogeneity was assessed by visual inspection of forest plots, I^2^ statistic and Chi^2^ test (*p* value of <0.05 was considered statistically significant). For outcomes with data reported by more than 10 studies, we assessed publication bias visually by inspection of funnel plots and statistically by Egger regression [39].

Subgroup meta-analyses were conducted on the primary outcomes according to a priori defined sources of potential clinical and methodological heterogeneity: age of participants at baseline (1–5 months, 6–11 months, 12–23 months, and 24–59 months), gender (males and females), World Health Organization (WHO) regions (African, European, Eastern Mediterranean, Region of the Americas, Southeast Asia, and Western Pacific), nutritional status at baseline (anemic and non-anemic, stunted and non-stunted, and underweight and normal weight), intervention duration (<3 months, 3–5 months, and 6–12 months), and intervention frequency (daily and intermittent). A subgroup meta-analysis was conducted only if there was at least one subgroup with 3 or more studies.

## 3. Results

### 3.1. Literature Search and Study Characteristics

**Figure 1:**
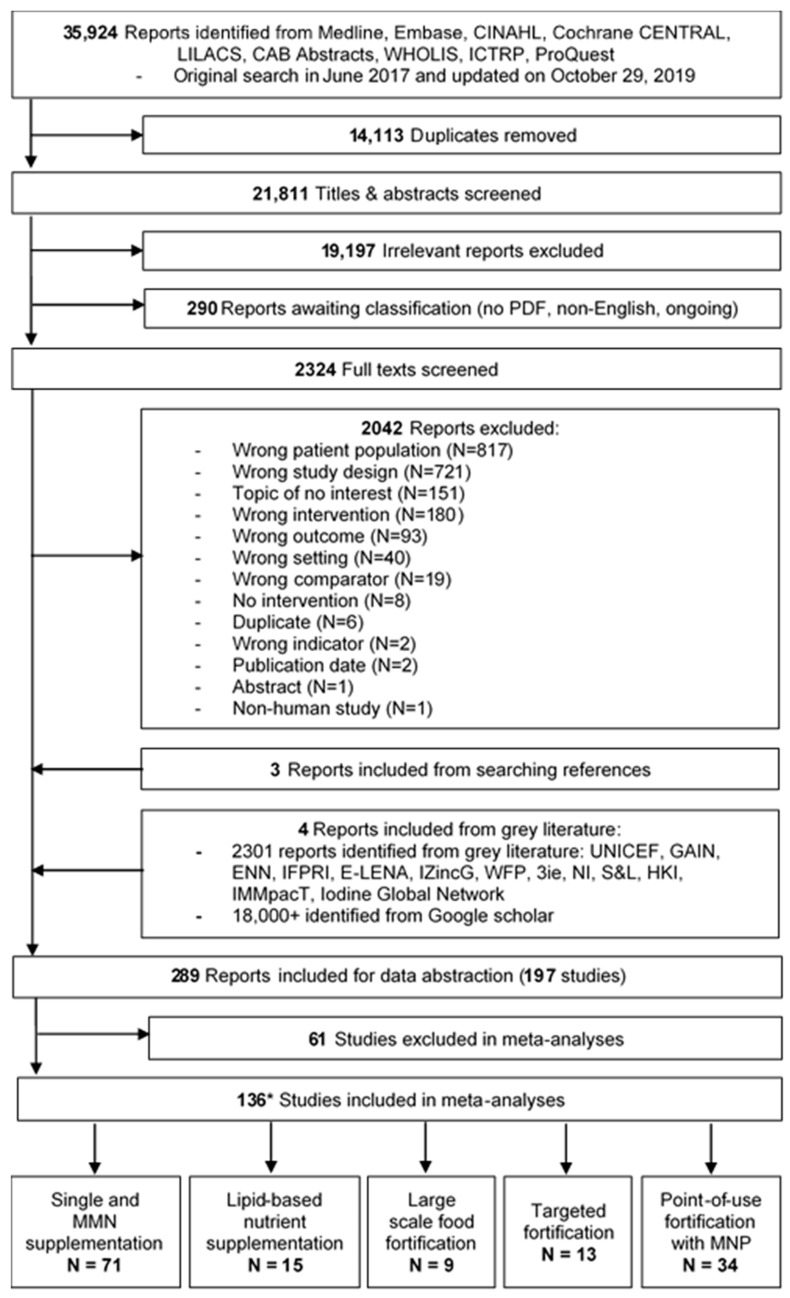
Screening and selection of studies included in the meta-analysis. *Some studies had multiple intervention arms that included different intervention types.

Of the 35,924 papers identified in the database search and 2301 identified from grey literature, 197 unique studies were eligible for inclusion (Figure 1). Of these studies, 121 studies were randomized controlled trials [40,41,42,43,44,45,46,47,48,49,50,51,52,53,54,55,56,57,58,59,60,61,62,63,64,65,66,67,68,69,70,71,72,73,74,75,76,77,78,79,80,81,82,83,84,85,86,87,88,89,90,91,92,93,94,95,96,97,98,99,100,101,102,103,104,105,106,107,108,109,110,111,112,113,114,115,116,117,118,119,120,121,122,123,124,125,126,127,128,129,130,131,132,133,134,135,136,137,138,139,140,141,142,143,144,145,146,147,148,149,150,151,152,153,154,155,156,157,158,159,160], 57 were cluster randomized controlled trials [161,162,163,164,165,166,167,168,169,170,171,172,173,174,175,176,177,178,179,180,181,182,183,184,185,186,187,188,189,190,191,192,193,194,195,196,197,198,199,200,201,202,203,204,205,206,207,208,209,210,211,212,213,214,215,216,217], 11 were non-randomized controlled trials [218,219,220,221,222,223,224,225,226,227,228], 2 were natural experiments [229,230], 4 were controlled before-after studies [231,232,233,234], 1 was an interrupted time-series study [235], and 1 was a propensity score matched retrospective cohort study [236]. 86 studies were conducted in Asia [43,48,49,54,55,57,59,60,61,62,69,72,73,74,75,76,78,79,80,83,86,87,92,97,98,107,110,117,119,120,121,122,123,129,131,134,138,139,141,143,144,146,148,149,151,152,153,156,157,159,161,162,164,169,176,177,178,180,184,187,190,191,192,193,194,196,197,199,200,201,204,205,206,207,208,213,215,218,223,224,228,229,230,231,234,235], 64 in Africa [40,41,42,45,46,47,50,53,56,63,65,66,67,68,70,71,82,84,85,88,94,95,96,99,101,104,105,108,109,111,112,113,114,115,116,118,127,130,136,137,140,142,147,150,155,158,165,172,174,175,181,183,185,186,188,203,209,211,212,214,216,226,233,236], 26 in South America [58,64,91,100,102,128,132,135,145,166,167,168,170,171,179,182,189,195,210,217,219,220,221,222,225,227] and 20 in North America [51,52,77,81,89,90,93,103,106,124,125,126,133,154,160,163,173,198,202,232]. One trial spanned three continents: Africa, Asia, and South America [44].

Sixty-one studies were on interventions that were insufficient to be pooled or reported outcomes in a format that could not be incorporated in the meta-analysis. These studies are summarized in a table format only (Table S1). The remaining 136 unique studies contributed data to the meta-analyses: 34 studies were on MNPs (32 efficacy and 9 effectiveness intervention–control comparisons), 13 studies on targeted fortification (all efficacy intervention–control comparisons), 15 studies on LNS supplementation (15 efficacy and 6 effectiveness intervention–control comparisons), 9 studies on large-scale fortification (all efficacy intervention–control comparisons), 14 studies on MMN supplementation (all efficacy intervention–control comparisons), 28 on iron supplementation (all efficacy intervention–control comparisons), 31 on zinc supplementation (all efficacy intervention-control), 4 on iron-folic acid supplementation (all efficacy intervention–control comparisons), and 16 on vitamin A supplementation (all efficacy intervention–control comparisons). Study characteristics of all included studies can be found in Table S2. All forest plots that are not presented in the results can be found in Figure S1.

### 3.2. Meta-Analysis

#### 3.2.1. Efficacy of Vitamin A Supplementation

Compared with placebo/no intervention, vitamin A supplementation was found to reduce the risk of all-cause mortality by 10% when cumulative incidence data was combined (RR 0.90, 95% CI 0.80 to 1.02; I^2^ = 26%, *p* = 0.10), though the upper CI has just crossed the line of no effect. However, we noted no significant effect on all-cause mortality when incidence rate data was combined. For secondary outcomes, vitamin A significantly increased plasma retinol concentration (MD 0.33 µmol/L, 95% CI 0.01 to 0.65; I^2^ = 99%, *p* = 0.04). No significant effects were observed for the other secondary outcomes.

#### 3.2.2. Efficacy of Zinc Supplementation

Zinc supplementation had no significant effect on the risk of anemia, stunting, wasting, and all-cause mortality. As expected, zinc supplementation decreased the risk of zinc deficiency (RR 0.37, 95% CI 0.22 to 0.62; I^2^ = 93%, *p* = 0.0001). Zinc supplementation also decreased the incidence of diarrhea (RR 0.89, 95% CI 0.82 to 0.97; I^2^ = 86%, *p* < 0.008). No significant effects were observed for any other secondary outcome.

#### 3.2.3. Efficacy of Iron Supplementation

**Figure 2:**
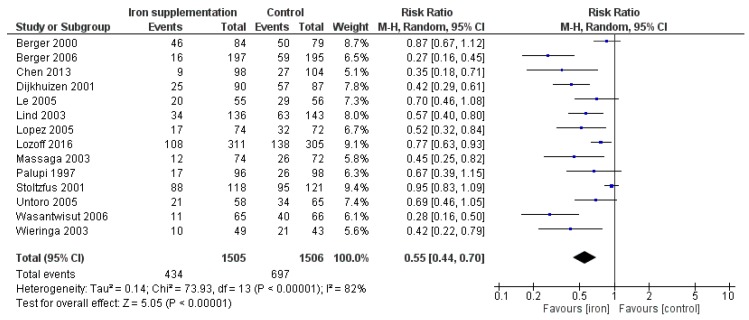
Iron supplementation versus placebo/no intervention (efficacy studies) on the risk of anemia (hemoglobin concentration < 110 g/L).

Iron supplementation was associated with a reduced risk of anemia (RR 0.55, 95% CI 0.44 to 0.70; I^2^ = 82%, *p* < 0.00001) (Figure 2). Subgroup analyses by age at baseline showed a trend towards a greater reduction in the risk of anemia among younger (1–5-month and 6–11-month age groups), as compared to older (24–59-month), children, though the test for subgroup differences was not significant (*p* for subgroup differences = 0.22). A greater reduction in the risk of anemia was observed among non-anemic, versus anemic, children at baseline (*p* for subgroup differences = 0.07) and among those who took iron supplements for 3–6 months, versus 6–12 months, (*p* for subgroup differences = 0.006), though data in the latter groups is lacking. No significant effects of iron supplementation were observed for the other primary outcomes (stunting and wasting). For the secondary outcomes, iron supplementation increased hemoglobin concentration (MD 6.02 g/L, 95% CI 4.28 to 7.76; I^2^ = 97%, *p* < 0.00001), plasma/serum ferritin concentrations (MD 20.48 µg/L, 95% CI 13.41 to 27.55; I^2^ = 95%, *p* < 0.00001), mental development (SMD 0.14, 95% CI 0.01 to 0.28; I^2^ = 3%, *p* = 0.04), and motor development (SMD 0.28, 95% CI 0.15 to 0.40; I^2^ = 0%, *p* < 0.0001), and decreased risk of iron deficiency (RR 0.21, 95% CI 0.12 to 0.39; I^2^ = 94%, *p* <0.00001) and iron deficiency anemia (RR 0.14, 95% CI 0.04 to 0.54; I^2^ = 88%, *p* = 0.004). There were no significant effects of iron supplementation on other secondary outcomes.

#### 3.2.4. Efficacy of Iron-Folic Acid Supplementation

**Figure 3:**
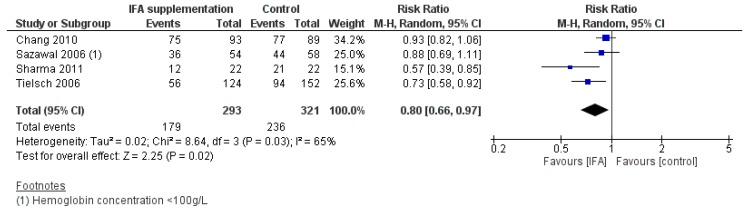
Iron-folic acid supplementation versus placebo/no intervention (efficacy studies) on the risk of anemia (hemoglobin concentration < 110 g/L).

Iron-folic acid supplementation was found to significantly reduce the risk of anemia compared with placebo/no intervention (RR 0.80, 95% CI 0.66 to 0.97; I^2^ = 65%, *p* = 0.02) (Figure 3). Subgroup analyses by frequency of supplementation showed a significant difference in risk of anemia by daily versus weekly iron-folic acid supplementation, with weekly regimens showing greater benefit (*p* for subgroup differences = 0.01), though only 1 study contributed data to the latter group. For the secondary outcomes, supplementation with iron-folic acid increased hemoglobin concentration (MD 3.06 g/L, 95% CI 1.16 to 4.97; I^2^ = 92%, *p* = 0.002). No significant difference between supplementation and control groups was observed for incidence of diarrhea.

#### 3.2.5. Efficacy of MMN Supplementation

**Figure 4:**
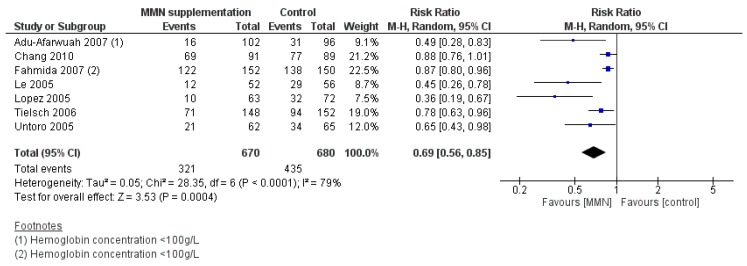
MMN supplementation versus placebo/no intervention (efficacy studies) on the risk of anemia (hemoglobin concentration < 110 g/L).

MMN supplementation was associated with a reduced risk of anemia (RR 0.69, 95% CI 0.56 to 0.85; I^2^ = 79%, *p* = 0.0004) (Figure 4). There appeared to be a greater reduction in risk of anemia among children aged 6–11 months compared to those aged 1–5 months at baseline (*p* for subgroup differences = 0.03), and among children who took MMN supplementation daily as compared to intermittently (*p* for subgroup differences = 0.05). Significant differences were also observed by region, where greater reductions in the risk of anemia were noted among children in the Americas, Western Pacific, and African regions, as compared to Southeast Asia (*p* for subgroup differences = 0.001), though sample size was not evenly dispersed among regions. MMN supplementation was associated with increased height (MD 0.36 cm, 95% CI 0.01 to 0.71; I^2^ = 0%, *p* = 0.04), length-for-age (z-score) (MD 0.09, 95% CI 0.00 to 0.17; I^2^ = 43%, *p* = 0.04), hemoglobin concentration (MD 4.40 g/L, 95% CI 2.91 to 5.90; I^2^ = 97%, *p* < 0.00001), plasma/serum ferritin concentration (MD 12.55 µg/L, 95% CI 3.93 to 21.27; I^2^ = 62%, *p* = 0.004), plasma/serum retinol concentration (MD 0.11 µmol/L, 95% CI 0.07 to 0.16; I^2^ = 87%, *p* < 0.00001), and plasma/serum zinc concentration (MD 0.95 µmol/L, 95% CI 0.23 to 1.67; I^2^ = 94%, *p* = 0.01), and decreased soluble transferrin receptor concentration (MD −0.19 mg/L log, 95% CI −0.30 to −0.09; I^2^ = 61%, *p* = 0.0002) and risk of iron deficiency (RR 0.41, 95% CI 0.25 to 0.66; I^2^ = 72%, *p* = 0.0003). MMN supplementation had no significant effects on other secondary outcomes.

#### 3.2.6. Efficacy of MNP Supplementation

**Figure 5:**
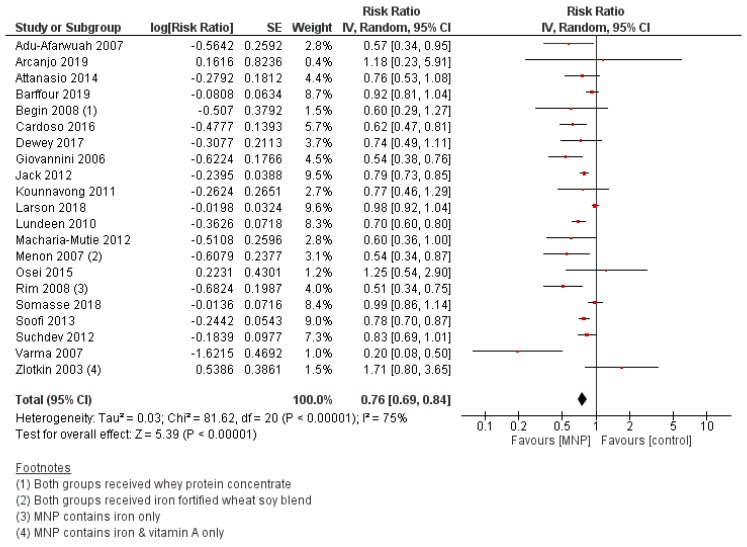
MNP supplementation versus placebo/no intervention (efficacy studies) on the risk of anemia (hemoglobin concentration < 110 g/L).

MNP supplementation was associated with a lower risk of anemia compared with no intervention/placebo (RR 0.76, 95% CI 0.69 to 0.84; I^2^ = 75%, *p* < 0.00001) (Figure 5). Subgroup analyses by age at baseline showed a trend towards a greater reduction in anemia risk among the older children (24–59-month age group) when compared to younger children (6–11 months and 12–23 months) (*p* for subgroup differences = 0.09). However, subgroup analyses by WHO region, intervention frequency (daily versus intermittent), intervention duration (3–5 months, 6–11 months, 12–17 months, and 18+ months), and nutritional status at baseline (anemic versus non-anemic) showed no significant differences between groups. No significant effects of MNP supplementation were observed for the other primary outcomes, including stunting, underweight, and wasting. For the secondary outcomes, MNP supplementation increased hemoglobin concentration (MD 1.85 g/L, 95% CI 1.24 to 2.47; I^2^ = 85%, *p* < 0.00001), serum/plasma ferritin (MD 11.08 µg/L, 95% CI 10.58 to 11.58; I^2^ = 95%, *p* < 0.00001), and risk of diarrhea (RR 1.30, 95% CI 1.11 to 1.53; I^2^ = 0%, *p* = 0.002), and decreased soluble transferrin receptor concentration (MD −0.86 mg/L, 95% CI −1.46 to −0.26; I^2^ = 84%, *p* = 0.005), risk of iron deficiency (RR 0.50 95% CI 0.40 to 0.63; I^2^ = 77%, *p* < 0.00001), and risk of iron-deficiency anemia (RR 0.45 95% CI 0.34 to 0.58; I^2^ = 23%, *p* < 0.00001). No significant effects were observed for other secondary outcomes.

#### 3.2.7. Efficacy of LNS Supplementation

**Figure 6:**
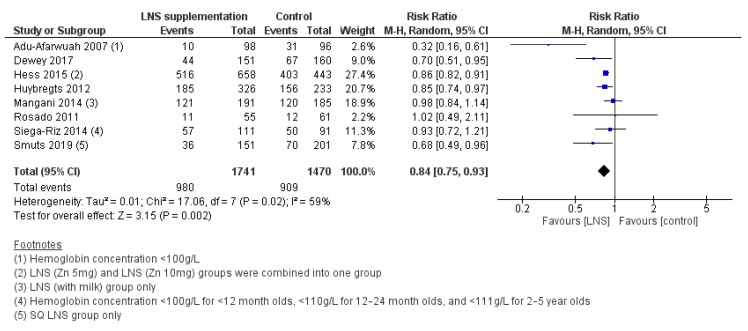
LNS supplementation versus placebo/no intervention (efficacy studies) on the risk of anemia (hemoglobin concentration < 110 g/L).

**Figure 7:**
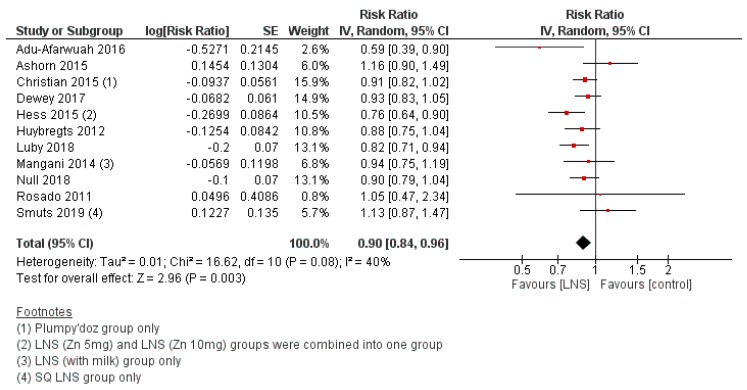
LNS supplementation versus placebo/no intervention (efficacy studies) on the risk of stunting (length-for-age z-score < −2).

LNS supplementation was associated with a reduced risk of anemia (RR 0.84, 95% CI 0.75 to 0.93; I^2^ = 59%, *p* = 0.002) (Figure 6), stunting (RR 0.90, 95% CI 0.84 to 0.96; I^2^ = 40%, *p* = 0.003) (Figure 7), and underweight (RR 0.90, 95% CI 0.81 to 1.01; I^2^ = 88%, *p* = 0.06), though the upper CI just crossed the line of no effect. There was no significant impact of LNS on wasting. Subgroup analyses by WHO region showed no differences between regions for anemia, stunting, wasting, or underweight. No other subgroup analyses were possible. For secondary outcomes, LNS supplementation led to an increase in length-for-age (z-score) (MD 0.11, 95% CI 0.05 to 0.17; I^2^ = 72%, *p* = 0.0002), weight-for-age (z-score) (MD 0.10, 95% CI 0.04 to 0.16; I^2^ = 68%, *p* = 0.001), and weight-for-height (z-score) (MD 0.09, 95% CI 0.04 to 0.14; I^2^ = 55%, *p* = 0.0009). LNS also led to improvements in mental development scores for language (SMD 0.13, 95% CI 0.02 to 0.23; I^2^ = 52%, *p* = 0.02) and personal-social/socioemotional (SMD 0.12, 95% CI -0.00 to 0.24; I^2^ = 65%, *p* = 0.05), along with improvements in motor development generally (SMD 0.13, 95% CI 0.00 to 0.25; I^2^ = 67%, *p* = 0.04). There were no significant effects of LNS supplementation on other secondary outcomes.

#### 3.2.8. Efficacy of Targeted Fortification

**Figure 8:**
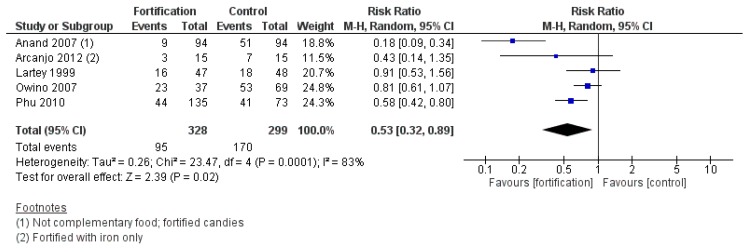
Targeted fortification versus placebo/no intervention (efficacy studies) on the risk of anemia (hemoglobin concentration < 110 g/L).

Targeted fortification was associated with a reduced risk of anemia compared with control (RR 0.53, 95% CI 0.32 to 0.89; I^2^ = 83%, *p* = 0.02) (Figure 8). Subgroup analyses were not done because all subgroups had less than three studies. For the secondary outcomes, targeted fortification increased hemoglobin (MD 4.97 g/L, 95% CI 1.81 to 8.12; I^2^ = 89%, *p* = 0.002) and serum/plasma ferritin (MD 8.19 µg/L, 95% CI 1.35 to 15.03; I^2^ = 99%, *p* = 0.02), and likely improved serum/plasma retinol concentrations (MD 2.31 µg/dL, 95% CI -0.02 to 4.63; I^2^ = 68%, *p* = 0.05), though the lower confidence interval just crossed the line of no effect. Targeted fortification, when compared to placebo or no intervention, also reduced the risk of iron-deficiency anemia (RR 0.28, 95% CI 0.14 to 0.59; I^2^ = 61%, *p* = 0.0007) and iron deficiency (RR 0.36, 95% CI 0.24 to 0.56; I^2^ = 69%, *p* < 0.00001). No significant effects were observed for other secondary outcomes.

#### 3.2.9. Efficacy of Large-Scale Food Fortification

**Figure 9:**
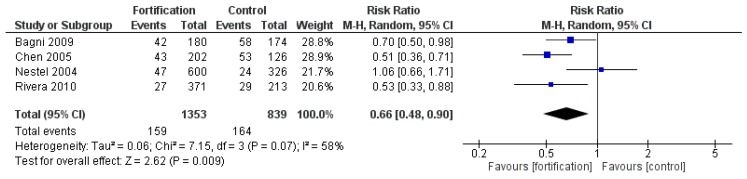
Large-scale food fortification with iron versus placebo/no intervention (efficacy studies) on the risk of anemia (hemoglobin concentration < 110 g/L).

Compared with placebo/no intervention, large-scale food fortification with MMN increased serum/plasma ferritin concentration (MD 2.80 µg/L, 95% CI 0.11 to 5.50; I^2^ = 88%, *p* = 0.04) but had no significant effects on hemoglobin and serum/plasma zinc concentrations. Large-scale food fortification with iron significantly decreased the risk of anemia (RR 0.66, 95% CI 0.48 to 0.90; I^2^ = 58%, *p* = 0.009) (Figure 9). However, there were an insufficient number of studies to do subgroup analyses for this primary outcome. Large-scale food fortification with iron had no significant effect on hemoglobin levels.

#### 3.2.10. Effectiveness of MNP Supplementation

**Figure 10:**
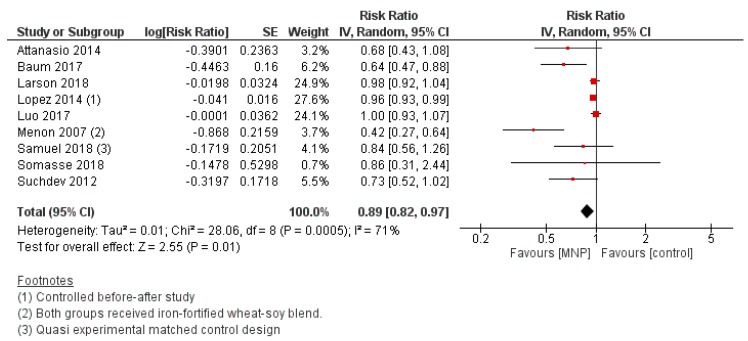
MNP supplementation versus placebo/no intervention (effectiveness studies) on the risk of anemia (hemoglobin concentration < 110 g/L).

Compared with placebo/no intervention, MNP supplementation was associated with a lower risk of anemia (RR 0.89, 95% CI 0.82 to 0.97; I^2^ = 71%, *p* = 0.01) (Figure 10). In subgroup analyses by WHO region, a trend towards a greater reduction in anemia was observed for children in Africa and the Americas, compared to Southeast Asia and Western Pacific regions (*p* for subgroup differences = 0.06). Subgroup analyses by intervention duration revealed a greater reduction in anemia risk among children who had been supplemented for less time (<3 months and 3–5 months) compared to children who had been supplemented for 6+ months (*p* for subgroup differences = 0.03). There were no significant differences in anemia risk by nutritional status at baseline (anemic versus non-anemic). MNP supplementation had no significant effect on hemoglobin concentration.

#### 3.2.11. Effectiveness of LNS Supplementation

**Figure 11:**
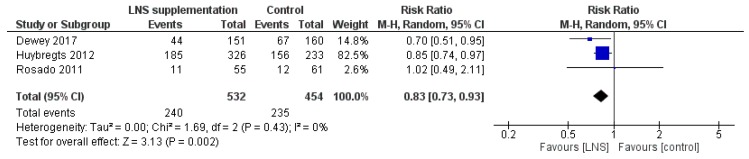
LNS supplementation versus placebo/no intervention (effectiveness studies) on the risk of anemia (hemoglobin concentration < 110 g/L).

LNS supplementation was associated with a reduced risk of anemia (RR 0.83, 95% CI 0.73 to 0.93; I^2^ = 0%, *p* = 0.002) (Figure 11). Subgroup analyses were not conducted on this primary outcome because no subgroups had three or more studies. No significant effect was observed for the other primary outcome (stunting). For the secondary outcomes, LNS supplementation led to an increase in weight-for-height (z-score) (MD 0.09, 95% CI 0.03 to 0.15; I^2^ = 62%, *p* = 0.006), weight-for-age (z-score) (MD 0.10, 95% CI 0.04 to 0.17; I^2^ = 66%, *p* = 0.002), and length-for-age (z-score) (MD 0.11, 95% CI 0.02 to 0.19; I^2^ = 75%, *p* = 0.02). There were no significant effects of LNS supplementation on other secondary outcomes.

## 4. Discussion

The purpose of this review was to summarize the available and up-to-date information on micronutrient supplementation and fortification interventions among children under-five in LMICs. To do so, we have undertaken an extensive number of analyses to examine both the efficacy and effectiveness (where data allowed) of these interventions on improving child health and development outcomes, including mortality, nutritional indicators (stunting, wasting, underweight, anemia), morbidities (lower respiratory tract infections, diarrhea), micronutrient deficiencies, and mental and motor skill development.

Though mortality data was not common, we were able to assess all-cause mortality among children taking zinc supplements and those taking vitamin A supplements, when compared to those with no intervention or placebo. With zinc, there was no significant difference between groups, and heterogenous estimates led to an uncertain pooled effect estimate with extremely wide confidence intervals. We performed two meta-analyses of vitamin A supplementation based on how the data was reported. When combining estimates as rate ratios, there was no difference between groups. However, when combining estimates as risk ratios (cumulative incidence), then there was a 10% reduction in the risk of mortality among the group supplemented with vitamin A, although the upper confidence interval just crossed the line of no effect (RR 0.90, 95% CI: 0.80 to 1.02). Of note, there were more participants in the risk ratio analysis when compared to the rate ratio analysis, with one cluster-RCT alone reporting 122,813 deaths within a 5-year trial in north India [169]. This finding is consistent with that of a Cochrane review, whereby the risk of all-cause mortality was reduced by 12% (RR 0.88, 95% CI: 0.83 to 0.93) [12]. It should be noted that data from 19 trials were included in the analysis, many of which were excluded from ours because of date restrictions (we included data from papers published after 1995). We were able to examine all-cause mortality by sex and, while there was a trend towards a greater reduction in mortality among girls versus boys, the test for subgroup differences was not significant (*p* = 0.13). A study of vitamin A supplementation in Guinea-Bissau has noted a more pronounced effect of the intervention on mortality that advantages girls [237]. However, a review of neonatal supplementation found lower mortality among boys [238], and the Cochrane review cited above found no difference between sexes, underscoring the need for more research on this subject.

Although vitamin A supplementation is thought to improve mortality largely by decreasing childhood infections, we found no difference in the incidence of diarrhea or lower respiratory tract infections among children supplemented with vitamin A compared to those who were not. However, we did note that the rate of diarrhea was reduced by over 10% and there was a 63% reduction in the risk of zinc deficiency among children taking zinc compared to those who did not.

For iron supplementation compared to placebo or no intervention, all of the indicators of iron status were significantly improved, including hemoglobin concentration, serum/plasma ferritin, anemia, iron deficiency, and iron-deficiency anemia. In addition, there were small improvements in mental development scores (SMD 0.14, 95% CI: 0.01 to 0.28) and slightly larger improvements in motor development scores (SMD 0.28, 95% CI: 0.15 to 0.40) among children who had been supplemented with iron. Caution should be taken when interpreting these results because of the heterogeneity in time to follow-up. For example, for our mental development analysis, two studies [239,240] followed children up for 6 months, one [137] for 12 months, and one [241] for 8 years. A standardized mean difference was used as the effect measure to account for the different scales used, a method that assumes that differences in standard deviations are due to measurement differences, when there may be actual variability among the study population. There have been mixed findings of iron supplementation on development outcomes. One review among children aged 4–34 months found no differential effect on mental or psychomotor development [242], and a previous Cochrane review looking at intermittent iron supplementation among children under 12 years of age was not able to meta-analyze these types of outcomes [20]. However, other reviews have supported the claim that iron improves later cognition and intelligence of children under-five and school-aged children [243,244]. Despite this, all evidence points to the benefits of iron supplementation for improving hemoglobin and iron status, which has led to development of WHO guidelines that recommend daily iron supplementation in under-five populations where anemia prevalence is 40% or higher [245]. However, oral iron should not be given to children in malaria-endemic areas, where appropriate surveillance and preventive/management measures for malaria are not available [245].

Only four included studies examined the effects of iron-folic acid compared to placebo or no intervention and, while there was a significant reduction in anemia (20% reduction) and improvement in hemoglobin, there was no impact on diarrhea incidence with this intervention.

Among RCTs that provided MNPs containing multiple micronutrients, the risk of anemia among children under-five was substantially reduced, by 24%. Among effectiveness studies of MNPs, the risk of anemia was reduced by 11%, demonstrating the robustness of this intervention for improving anemia even in less controlled settings. MNPs did not have an effect on other nutritional indicators including stunting, wasting, and underweight, though risk of iron deficiency and iron-deficiency anemia was reduced by 50% and 55%, respectively. Of note, there was a 30% increased risk of diarrhea with MNPs when compared to placebo or no intervention. These findings are mostly in line with the recent Cochrane review on point-of-use fortification, though the authors found no significant increase or decrease on diarrhea among participants [26]. Our results differ due to the inclusion of two additional trials in our analysis. Of these four trials, the dose of included zinc ranged from 0 mg [51] to 4.1 mg [196,203] to 10 mg [204]. Given that even 10 mg is lower than the 20 mg daily dose recommended by WHO to treat diarrhea [245], there may be room to lower this risk by increasing the dose of zinc in MNPs. However, a balance must be struck because of the notion that high zinc can lead to competition for absorption with iron. Knowing the significant contribution of diarrhea to deaths among children under-five, this is a finding that warrants further investigation. Some trials have previously reported an increase in diarrheal episodes directly following intervention initiation, with a subsequent decline in diarrhea in the following days [26], underscoring a window of time where children should be closely monitored. Interestingly, in our analysis of MMN supplementation (not in the form of powder for point-of-use fortification) versus placebo or no intervention, there was no increased risk of diarrhea incidence (RR 0.97, 95% CI: 0.87 to 1.09) and the zinc dosages ranged from 5 mg [104] to 10 mg [59,130,205] to 20 mg [49]. MMN also showed improvements in plasma/serum retinol and zinc and increases in length-for-age and weight-for-age z-scores, underscoring benefits of MMN that were not noted for MNPs.

For children under-five, we found that the LNS was effective at improving nutritional outcomes including anemia (16% reduction), stunting (10% reduction), and underweight (10% reduction). These findings are in line with the recent Cochrane review, which found a 21% reduction in anemia, a 7% and 15% reduction in moderate and severe stunting respectively, an 18% reduction in moderate wasting, and a 15% reduction in moderate underweight [29]. Five LNS studies examined the effectiveness of this intervention and found similar results in terms of anemia and length-for-age, weight-for-age, and weight-for-height z-scores, underscoring the utility of this intervention for preventative, community-based programs. Efficacy studies also noted some positive effects of LNS on mental and motor development outcomes, highlighting the link between early nutrition and development.

There were several studies that examined the efficacy of targeted fortification interventions and fewer that looked at fortification of staple foods. For both comparisons, we meta-analyzed vehicles that had been fortified with multiple, as opposed to single, micronutrients because this was more common. Similar to the effects of MNPs and MMN, we found that delivering multiple micronutrients to children through complementary foods or staple foods improved anemia and some other indicators of iron status (e.g., serum/plasma ferritin and iron deficiency), but had no impact on other nutritional indicators such as length-for-age, weight-for-age, or weight-for-height.

There are many strengths of this review, including its comprehensive nature and inclusion of efficacy and effectiveness studies. Though most program evaluations that we came across did not meet our study design criteria, as they were cross-sectional surveys before and after implementation, we were able to include several effectiveness trials of MNPs and LNS that utilized health facility- or community-based platforms to deliver interventions, which is a good representation of real-world scenarios. We have examined within this review interventions that are most relevant to children under-five in LMICs, and many of our findings extend upon or align with those of other evidence synthesis exercises. This inclusion of several different interventions and comparisons may also be a limitation of this review, as it is bulky in size, with over 130 different analyses undertaken. This has made it difficult to discuss findings completely, as interventions and populations represented by the data are so heterogenous. One must be careful not to directly compare the efficacy of one intervention to another, as this would require network meta-analysis, which we have not undertaken.

It has become clear that for micronutrient interventions to be maximally effective, it will be critical to consider context. We have outlined some strategies, such as LNS, that improve anemia, stunting, and underweight, whereas others, such as zinc supplementation, work to reduce diarrhea. We have demonstrated the reduction in all-cause mortality with vitamin A, and improvements in iron status and anemia with iron or multiple micronutrients (delivered in several forms). Considering the high prevalence of multiple deficiencies among children in LMICs, and the frequency with which these outcomes occur, it is probable that several of these strategies should be used concurrently, and in a complementary fashion. However, in some situations, it might be necessary to consider the cost-benefit and other trade-offs of using one intervention over another to improve a specific outcome. Programs will also need to take into account the contextual factors that will ensure coverage, benefit, and sustainability of an intervention. These are factors that we were not able to examine in this review, and include cost, feasibility of implementation, strategies for monitoring and evaluation, and population-specific factors including gender-related barriers to uptake and prevalence of deficiencies at a sub-national level (which are often masked by national-level estimates). A careful diagnostic assessment should be undertaken to understand which strategies will be most beneficial for a target population. Nevertheless, the results of this review have further added to the evidence base which advocates for micronutrient supplementation and fortification strategies for improving health and development outcomes among children under-five. In particular, the positive findings emanating from the meta-analyses of effectiveness studies should support and bolster efforts in countries to reach more children with these interventions.

## Supplementary Materials

The following are available online at https://www.mdpi.com/2072-6643/12/2/289/s1, Figure S1: Additional Forest Plots, Table S1: Eligible Studies Excluded from Meta-Analyses, Table S2: Eligible Studies Included in Meta-Analyses, Text S1: MEDLINE Search Strategy.

## References

[1] Bailey R.L., West K.P., Black R.E. (2015). The epidemiology of global micronutrient deficiencies. Ann. Nutr. Metab..

[2] Stevens G.A., Finucane M.M., De-Regil L.M., Paciorek C.J., Flaxman S.R., Branca F., Pena-Rosas J.P., Bhutta Z.A., Ezzati M., Nutrition Impact Model Study Group (2013). Global, regional, and national trends in haemoglobin concentration and prevalence of total and severe anaemia in children and pregnant and non-pregnant women for 1995–2011: A systematic analysis of population-representative data. Lancet Glob. Health.

[3] Stevens G.A., Bennett J.E., Hennocq Q., Lu Y., De-Regil L.M., Rogers L., Danaei G., Li G., White R.A., Flaxman S.R. (2015). Trends and mortality effects of vitamin A deficiency in children in 138 low-income and middle-income countries between 1991 and 2013: A pooled analysis of population-based surveys. Lancet Glob. Health.

[4] Andersson M., Karumbunathan V., Zimmermann M.B. (2012). Global iodine status in 2011 and trends over the past decade. J. Nutr..

[5] Wessells K.R., Brown K.H. (2012). Estimating the global prevalence of zinc deficiency: Results based on zinc availability in national food supplies and the prevalence of stunting. PLoS ONE.

[6] World Health Organization (2017). Nutritional Anaemias: Tools for Effective Prevention and Control.

[7] Lozoff B. (2007). Iron deficiency and child development. Food Nutr. Bull..

[8] Sanghvi T., Van Ameringen M., Baker J., Fiedler J., Borwankar R., Phillips M., Houston R., Ross J., Heymann H., Dary O. (2007). Vitamin and mineral deficiencies technical situation analysis: A report for the Ten Year Strategy for the Reduction of Vitamin and Mineral Deficiencies. Food Nutr. Bull..

[9] Black R.E., Allen L.H., Bhutta Z.A., Caulfield L.E., de Onis M., Ezzati M., Mathers C., Rivera J. (2008). Maternal and Child Undernutrition Study Group. Maternal and child undernutrition: Global and regional exposures and health consequences. Lancet.

[10] Christian P., Murray-Kolb L.E., Khatry S.K., Katz J., Schaefer B.A., Cole P.M., Leclerq S.C., Tielsch J.M. (2010). Prenatal micronutrient supplementation and intellectual and motor function in early school-aged children in Nepal. JAMA.

[11] Walker S.P., Wachs T.D., Grantham-McGregor S., Black M.M., Nelson C.A., Huffman S.L., Baker-Henningham H., Chang S.M., Hamadani J.D., Lozoff B. (2011). Inequality in early childhood: Risk and protective factors for early child development. Lancet.

[12] Imdad A., Mayo-Wilson E., Herzer K., Bhutta Z.A. (2017). Vitamin A supplementation for preventing morbidity and mortality in children from six months to five years of age. Cochrane Database Syst. Rev..

[13] Mayo-Wilson E., Junior J.A., Imdad A., Dean S., Chan X.H., Chan E.S., Jaswal A., Bhutta Z.A. (2014). Zinc supplementation for preventing mortality, morbidity, and growth failure in children aged 6 months to 12 years of age. Cochrane Database Syst. Rev..

[14] Brown K.H., Peerson J.M., Baker S.K., Hess S.Y. (2009). Preventive zinc supplementation among infants, preschoolers, and older prepubertal children. Food Nutr. Bull..

[15] Black R.E., Victora C.G., Walker S.P., Bhutta Z.A., Christian P., de Onis M., Ezzati M., Grantham-McGregor S., Katz J., Martorell R. (2013). Maternal and child undernutrition and overweight in low-income and middle-income countries. Lancet.

[16] World Health Organization (2006). Guidelines on Food Fortification with Micronutrients.

[17] Kawai K., Spiegelman D., Shankar A.H., Fawzi W.W. (2011). Maternal multiple micronutrient supplementation and pregnancy outcomes in developing countries: Meta-analysis and meta-regression. Bull. World Health Organ..

[18] Dewey K.G., Arimond M. (2012). Lipid-based nutrient supplements: How can they combat child malnutrition?. PLoS Med..

[19] Home Fortification Technical Advisory Group (2016). Home Fortification with Micronutrient Powders Sight and Life.

[20] De-Regil L.M., Jefferds M.E., Sylvetsky A.C., Dowswell T. (2011). Intermittent iron supplementation for improving nutrition and development in children under 12 years of age. Cochrane Database Syst. Rev..

[21] Sachdev H., Gera T., Nestel P. (2005). Effect of iron supplementation on mental and motor development in children: Systematic review of randomised controlled trials. Public Health Nutr..

[22] Gogia S., Sachdev H.S. (2012). Zinc supplementation for mental and motor development in children. Cochrane Database Syst. Rev..

[23] Yakoob M.Y., Salam R.A., Khan F.R., Bhutta Z.A. (2016). Vitamin D supplementation for preventing infections in children under five years of age. Cochrane Database Syst. Rev..

[24] Allen L.H., Peerson J.M., Olney D.K. (2009). Provision of multiple rather than two or fewer micronutrients more effectively improves growth and other outcomes in micronutrient-deficient children and adults. J. Nutr..

[25] Ramakrishnan U., Nguyen P., Martorell R. (2009). Effects of micronutrients on growth of children under 5 y of age: Meta-analyses of single and multiple nutrient interventions. Am. J. Clin. Nutr..

[26] De-Regil L.M., Jefferds M.E.D., Pena-Rosas J.P. (2017). Point-of-use fortification of foods with micronutrient powders containing iron in children of preschool and school-age. Cochrane Database Syst. Rev..

[27] De-Regil L.M., Suchdev P.S., Vist G.E., Walleser S., Pena-Rosas J.P. (2011). Home fortification of foods with multiple micronutrient powders for health and nutrition in children under two years of age. Cochrane Database Syst. Rev..

[28] Salam R.A., MacPhail C., Das J.K., Bhutta Z.A. (2013). Effectiveness of Micronutrient Powders (MNP) in women and children. BMC Public Health.

[29] Das J.K., Salam R.A., Hadi Y.B., Sadiq Sheikh S., Bhutta A.Z., Weise Prinzo Z., Bhutta Z.A. (2019). Preventive lipid-based nutrient supplements given with complementary foods to infants and young children 6 to 23 months of age for health, nutrition, and developmental outcomes. Cochrane Database Syst. Rev..

[30] Stewart C.P., Wessells K.R., Arnold C.D., Huybregts L., Ashorn P., Becquey E., Humphrey J.H., Dewey K.G. (2019). Lipid-based nutrient supplements and all-cause mortality in children 6-24 months of age: A meta-analysis of randomized controlled trials. Am. J. Clin. Nutr..

[31] Eichler K., Wieser S., Ruthemann I., Brugger U. (2012). Effects of micronutrient fortified milk and cereal food for infants and children: A systematic review. BMC Public Health.

[32] Ramakrishnan U., Goldenberg T., Allen L.H. (2011). Do multiple micronutrient interventions improve child health, growth, and development?. J. Nutr..

[33] Das J.K., Salam R.A., Kumar R., Bhutta Z.A. (2013). Micronutrient fortification of food and its impact on woman and child health: A systematic review. Syst. Rev..

[34] Keats E.C., Neufeld L.M., Garrett G.S., Mbuya M.N.N., Bhutta Z.A. (2019). Improved micronutrient status and health outcomes in low- and middle-income countries following large-scale fortification: Evidence from a systematic review and meta-analysis. Am. J. Clin. Nutr..

[35] Matsuyama M., Harb T., David M., Davies P.S., Hill R.J. (2017). Effect of fortified milk on growth and nutritional status in young children: A systematic review and meta-analysis. Public Health Nutr..

[36] Keats E.C., Imdad A., Das J.K., Bhutta Z.A. (2018). Protocol: Efficacy and effectiveness of micronutrient supplementation and fortification interventions on the health and nutritional status of children under-five in low and middle-income countries. Campbell Syst. Rev..

[37] Higgins J.P., Altman D.G., Gotzsche P.C., Juni P., Moher D., Oxman A.D., Savovic J., Schulz K.F., Weeks L., Sterne J.A. (2011). The Cochrane Collaboration’s tool for assessing risk of bias in randomised trials. BMJ.

[38] Cochrane Effective Practice Organisation of Care (EPOC) (2017). Suggested risk of bias criteria for EPOC reviews. EPOC Resources for Review Authors.

[39] Egger M., Davey Smith G., Schneider M., Minder C. (1997). Bias in meta-analysis detected by a simple, graphical test. BMJ.

[40] Adom T., Steiner-Asiedu M., Sakyi-Dawson E., Anderson A.K. (2010). Effect of fortification of maize with cowpea and iron on growth and anaemia status of children. Afr. J. Food Sci..

[41] Adu-Afarwuah S., Lartey A., Brown K.H., Zlotkin S., Briend A., Dewey K.G. (2007). Randomized comparison of 3 types of micronutrient supplements for home fortification of complementary foods in Ghana: Effects on growth and motor development. Am. J. Clin. Nutr..

[42] Adu-Afarwuah S., Lartey A., Okronipa H., Ashorn P., Peerson J.M., Arimond M., Ashorn U., Zeilani M., Vosti S., Dewey K.G. (2016). Small-quantity, lipid-based nutrient supplements provided to women during pregnancy and 6 mo postpartum and to their infants from 6 mo of age increase the mean attained length of 18-mo-old children in semi-urban Ghana: A randomized controlled trial. Am. J. Clin. Nutr..

[43] Anand K., Lakshmy R., Janakarajan V.N., Ritvik A., Misra P., Pandey R.M., Kapoor S.K., Sankar R., Bulusu S. (2007). Effect of consumption of micronutrient fortified candies on the iron and vitamin A status of children aged 3-6 years in rural Haryana. Indian Pediatr..

[44] (1998). Randomised trial to assess benefits and safety of vitamin A supplementation linked to immunisation in early infancy. WHO/CHD Immunisation-Linked Vitamin A Supplementation Study Group. Lancet.

[45] Ashorn P., Alho L., Ashorn U., Cheung Y.B., Dewey K.G., Gondwe A., Harjunmaa U., Lartey A., Phiri N., Phiri T.E. (2015). Supplementation of Maternal Diets during Pregnancy and for 6 Months Postpartum and Infant Diets Thereafter with Small-Quantity Lipid-Based Nutrient Supplements Does Not Promote Child Growth by 18 Months of Age in Rural Malawi: A Randomized Controlled Trial. J. Nutr..

[46] Asibey-Berko E., Zlotkin S.H., Yeung G.S., Nti-Nimako W., Ahunu B., Kyei-Faried S., Johnston J.L., Tondeur M.C., Mannar V. (2007). Dual fortification of salt with iron and iodine in women and children in rural Ghana. East Afr. Med. J..

[47] Ayah R.A., Mwaniki D.L., Magnussen P., Tedstone A.E., Marshall T., Alusala D., Luoba A., Kaestel P., Michaelsen K.F., Friis H. (2007). The effects of maternal and infant vitamin A supplementation on vitamin A status: A randomised trial in Kenya. Br. J. Nutr..

[48] Baqui A.H., de Francisco A., Arifeen S.E., Siddique A.K., Sack R.B. (1995). Bulging fontanelle after supplementation with 25,000 IU of vitamin A in infancy using immunization contacts. Acta Paediatr..

[49] Baqui A.H., Zaman K., Persson L.A., El Arifeen S., Yunus M., Begum N., Black R.E. (2003). Simultaneous weekly supplementation of iron and zinc is associated with lower morbidity due to diarrhea and acute lower respiratory infection in Bangladeshi infants. J. Nutr..

[50] Barth-Jaeggi T., Moretti D., Kvalsvig J., Holding P.A., Njenga J., Mwangi A., Chhagan M.K., Lacroix C., Zimmermann M.B. (2015). In-home fortification with 2.5 mg iron as NaFeEDTA does not reduce anaemia but increases weight gain: A randomised controlled trial in Kenyan infants. Matern. Child Nutr..

[51] Begin F., Santizo M.C., Peerson J.M., Torun B., Brown K.H. (2008). Effects of bovine serum concentrate, with or without supplemental micronutrients, on the growth, morbidity, and micronutrient status of young children in a low-income, peri-urban Guatemalan community. Eur. J. Clin. Nutr..

[52] Bentley M.E., Caulfield L.E., Ram M., Santizo M.C., Hurtado E., Rivera J.A., Ruel M.T., Brown K.H. (1997). Zinc supplementation affects the activity patterns of rural Guatemalan infants. J. Nutr..

[53] Berger J., Dyck J.L., Galan P., Aplogan A., Schneider D., Traissac P., Hercberg S. (2000). Effect of daily iron supplementation on iron status, cell-mediated immunity, and incidence of infections in 6-36 month old Togolese children. Eur. J. Clin. Nutr..

[54] Berger J., Ninh N.X., Khan N.C., Nhien N.V., Lien D.K., Trung N.Q., Khoi H.H. (2006). Efficacy of combined iron and zinc supplementation on micronutrient status and growth in Vietnamese infants. Eur. J. Clin. Nutr..

[55] Bhandari N., Bahl R., Taneja S., Strand T., Molbak K., Ulvik R.J., Sommerfelt H., Bhan M.K. (2002). Substantial reduction in severe diarrheal morbidity by daily zinc supplementation in young north Indian children. Pediatrics.

[56] Bisimwa G., Owino V.O., Bahwere P., Dramaix M., Donnen P., Dibari F., Collins S. (2012). Randomized controlled trial of the effectiveness of a soybean-maize-sorghum-based ready-to-use complementary food paste on infant growth in South Kivu, Democratic Republic of Congo. Am. J. Clin. Nutr..

[57] Brooks W.A., Santosham M., Naheed A., Goswami D., Wahed M.A., Diener-West M., Faruque A.S., Black R.E. (2005). Effect of weekly zinc supplements on incidence of pneumonia and diarrhoea in children younger than 2 years in an urban, low-income population in Bangladesh: Randomised controlled trial. Lancet.

[58] Caulfield L.E., Zavaleta N., Chen P., Colombo J., Kannass K. (2013). Mineral status of non-anemic Peruvian infants taking an iron and copper syrup with or without zinc from 6 to 18 months of age: A randomized controlled trial. Nutrition.

[59] Chang S., El Arifeen S., Bari S., Wahed M.A., Rahman K.M., Rahman M.T., Mahmud A.B., Begum N., Zaman K., Baqui A.H. (2010). Supplementing iron and zinc: Double blind, randomized evaluation of separate or combined delivery. Eur. J. Clin. Nutr..

[60] Chen C.M., Wang Y.Y., Chang S.Y. (2010). Effect of in-home fortification of complementary feeding on intellectual development of Chinese children. Biomed. Environ. Sci..

[61] Chen K., Chen X.R., Zhang L., Luo H.Y., Gao N., Wang J., Fu G.Y., Mao M. (2013). Effect of simultaneous supplementation of vitamin A and iron on diarrheal and respiratory tract infection in preschool children in Chengdu City, China. Nutrition.

[62] Chen K., Li T.Y., Chen L., Qu P., Liu Y.X. (2008). Effects of vitamin A, vitamin A plus iron and multiple micronutrient-fortified seasoning powder on preschool children in a suburb of Chongqing, China. J. Nutr. Sci. Vitaminol. (Tokyo).

[63] Drammeh B.S., Marquis G.S., Funkhouser E., Bates C., Eto I., Stephensen C.B. (2002). A randomized, 4-month mango and fat supplementation trial improved vitamin A status among young Gambian children. J. Nutr..

[64] Dutra-de-Oliveira J.E., de Almeida C.A. (2002). Domestic drinking water—An effective way to prevent anemia among low socioeconomic families in Brazil. Food Nutr. Bull..

[65] Ekvall H., Premji Z., Bjorkman A. (2000). Micronutrient and iron supplementation and effective antimalarial treatment synergistically improve childhood anaemia. Trop. Med. Int. Health.

[66] Ermis B., Demirel F., Demircan N., Gurel A. (2002). Effects of three different iron supplementations in term healthy infants after 5 months of life. J. Trop. Pediatr..

[67] Esamai F., Liechty E., Ikemeri J., Westcott J., Kemp J., Culbertson D., Miller L.V., Hambidge K.M., Krebs N.F. (2014). Zinc absorption from micronutrient powder is low but is not affected by iron in Kenyan infants. Nutrients.

[68] Faber M., Kvalsvig J.D., Lombard C.J., Benade A.J. (2005). Effect of a fortified maize-meal porridge on anemia, micronutrient status, and motor development of infants. Am. J. Clin. Nutr..

[69] Fahmida U., Rumawas J.S., Utomo B., Patmonodewo S., Schultink W. (2007). Zinc-iron, but not zinc-alone supplementation, increased linear growth of stunted infants with low haemoglobin. Asia Pac. J. Clin. Nutr..

[70] Fawzi W.W., Herrera M.G., Willett W.C., Nestel P., el Amin A., Mohamed K.A. (1997). The effect of vitamin A supplementation on the growth of preschool children in the Sudan. Am. J. Public Health.

[71] Fisker A.B., Bale C., Jorgensen M.J., Balde I., Hornshoj L., Bibby B.M., Aaby P., Benn C.S. (2013). High-dose vitamin A supplementation administered with vaccinations after 6 months of age: Sex-differential adverse reactions and morbidity. Vaccine.

[72] Giovannini M., Sala D., Usuelli M., Livio L., Francescato G., Braga M., Radaelli G., Riva E. (2006). Double-blind, placebo-controlled trial comparing effects of supplementation with two different combinations of micronutrients delivered as sprinkles on growth, anemia, and iron deficiency in cambodian infants. J. Pediatr. Gastroenterol. Nutr..

[73] Gokcay G., Ozden T., Karakas Z., Karabayir N., Yildiz I., Abali S., Sahip Y. (2012). Effect of iron supplementation on development of iron deficiency anemia in breastfed infants. J. Trop. Pediatr..

[74] Gupta D.N., Mondal S.K., Ghosh S., Rajendran K., Sur D., Manna B. (2003). Impact of zinc supplementation on diarrhoeal morbidity in rural children of West Bengal, India. Acta Paediatr..

[75] Gupta D.N., Rajendran K., Mondal S.K., Ghosh S., Bhattacharya S.K. (2007). Operational feasibility of implementing community-based zinc supplementation: Impact on childhood diarrheal morbidity. Pediatr. Infect. Dis. J..

[76] Hamadani J.D., Fuchs G.J., Osendarp S.J., Khatun F., Huda S.N., Grantham-McGregor S.M. (2001). Randomized controlled trial of the effect of zinc supplementation on the mental development of Bangladeshi infants. Am. J. Clin. Nutr..

[77] Iannotti L.L., Dulience S.J., Green J., Joseph S., Francois J., Antenor M.L., Lesorogol C., Mounce J., Nickerson N.M. (2014). Linear growth increased in young children in an urban slum of Haiti: A randomized controlled trial of a lipid-based nutrient supplement. Am. J. Clin. Nutr..

[78] Nagpal J., Sachdev H.P., Singh T., Mallika V. (2004). A randomized placebo-controlled trial of iron supplementation in breastfed young infants initiated on complementary feeding: Effect on haematological status. J. Health Popul. Nutr..

[79] Kapur D., Sharma S., Agarwal K.N. (2003). Effectiveness of nutrition education, iron supplementation or both on iron status in children. Indian Pediatr..

[80] Kartasasmita C.B., Rosmayudi O., Deville W., Demedts M. (1995). Plasma retinol level, vitamin A supplementation and acute respiratory infections in children of 1-5 years old in a developing country. Respiratory Diseases Working Group. Tuber. Lung Dis..

[81] Kemmer T.M., Omer P.S., Gidvani-Diaz V.K., and Coello M., Donald S. (2012). Acceptance and Effect of Ferrous Fumarate Containing Micronutrient Sprinkles on Anemia, Iron Deficiency and Anthropometrics in Honduran Children. Anemia.

[82] Kikafunda J.K., Walker A.F., Allan E.F., Tumwine J.K. (1998). Effect of zinc supplementation on growth and body composition of Ugandan preschool children: A randomized, controlled, intervention trial. Am. J. Clin. Nutr..

[83] Kounnavong S., Sunahara T., Mascie-Taylor C.G., Hashizume M., Okumura J., Moji K., Boupha B., Yamamoto T. (2011). Effect of daily versus weekly home fortification with multiple micronutrient powder on haemoglobin concentration of young children in a rural area, Lao People’s Democratic Republic: A randomised trial. Nutr. J..

[84] Kujinga P., Galetti V., Onyango E., Jakab V., Buerkli S., Andang’o P., Brouwer I.D., Zimmermann M.B., Moretti D. (2018). Effectiveness of zinc-fortified water on zinc intake, status and morbidity in Kenyan pre-school children: A randomised controlled trial. Public Health Nutr..

[85] Lartey A., Manu A., Brown K.H., Peerson J.M., Dewey K.G. (1999). A randomized, community-based trial of the effects of improved, centrally processed complementary foods on growth and micronutrient status of Ghanaian infants from 6 to 12 mo of age. Am. J. Clin. Nutr..

[86] Hop L.T., Berger J. (2005). Multiple micronutrient supplementation improves anemia, micronutrient nutrient status, and growth of Vietnamese infants: Double-blind, randomized, placebo-controlled trial. J. Nutr..

[87] Lind T., Lonnerdal B., Stenlund H., Ismail D., Seswandhana R., Ekstrom E.C., Persson L.A. (2003). A community-based randomized controlled trial of iron and zinc supplementation in Indonesian infants: Interactions between iron and zinc. Am. J. Clin. Nutr..

[88] Lo N.B., Aaron G.J., Hess S.Y., Dossou N.I., Guiro A.T., Wade S., Brown K.H. (2011). Plasma zinc concentration responds to short-term zinc supplementation, but not zinc fortification, in young children in Senegal1,2. Am. J. Clin. Nutr..

[89] Long K.Z., Montoya Y., Hertzmark E., Santos J.I., Rosado J.L. (2006). A double-blind, randomized, clinical trial of the effect of vitamin A and zinc supplementation on diarrheal disease and respiratory tract infections in children in Mexico City, Mexico. Am. J. Clin. Nutr..

[90] Long K.Z., Rosado J.L., DuPont H.L., Hertzmark E., Santos J.I. (2007). Supplementation with vitamin A reduces watery diarrhoea and respiratory infections in Mexican children. Br. J. Nutr..

[91] Lopez de Romana G., Cusirramos S., Lopez de Romana D., Gross R. (2005). Efficacy of multiple micronutrient supplementation for improving anemia, micronutrient status, growth, and morbidity of Peruvian infants. J. Nutr..

[92] Lozoff B., Jiang Y., Li X., Zhou M., Richards B., Xu G., Clark K.M., Liang F., Kaciroti N., Zhao G. (2016). Low-Dose Iron Supplementation in Infancy Modestly Increases Infant Iron Status at 9 Mo without Decreasing Growth or Increasing Illness in a Randomized Clinical Trial in Rural China. J. Nutr..

[93] Lozoff B., Wolf A.W., Jimenez E. (1996). Iron-deficiency anemia and infant development: Effects of extended oral iron therapy. J. Pediatr..

[94] Luabeya K.K., Mpontshane N., Mackay M., Ward H., Elson I., Chhagan M., Tomkins A., Van den Broeck J., Bennish M.L. (2007). Zinc or multiple micronutrient supplementation to reduce diarrhea and respiratory disease in South African children: A randomized controlled trial. PLoS ONE.

[95] Macharia-Mutie C.W., Moretti D., Van den Briel N., Omusundi A.M., Mwangi A.M., Kok F.J., Zimmermann M.B., Brouwer I.D. (2012). Maize porridge enriched with a micronutrient powder containing low-dose iron as NaFeEDTA but not amaranth grain flour reduces anemia and iron deficiency in Kenyan preschool children. J. Nutr..

[96] Maleta K.M., Phuka J., Alho L., Cheung Y.B., Dewey K.G., Ashorn U., Phiri N., Phiri T.E., Vosti S.A., Zeilani M. (2015). Provision of 10-40 g/d Lipid-Based Nutrient Supplements from 6 to 18 Months of Age Does Not Prevent Linear Growth Faltering in Malawi. J. Nutr..

[97] Malik A., Taneja D.K., Devasenapathy N., Rajeshwari K. (2013). Short-course prophylactic zinc supplementation for diarrhea morbidity in infants of 6 to 11 months. Pediatrics.

[98] Manaseki-Holland S., Maroof Z., Bruce J., Mughal M.Z., Masher M.I., Bhutta Z.A., Walraven G., Chandramohan D. (2012). Effect on the incidence of pneumonia of vitamin D supplementation by quarterly bolus dose to infants in Kabul: A randomised controlled superiority trial. Lancet.

[99] Mangani C., Ashorn P., Maleta K., Phuka J., Thakwalakwa C., Dewey K., Manary M., Puumalainen T., Cheung Y.B. (2014). Lipid-based nutrient supplements do not affect the risk of malaria or respiratory morbidity in 6- to 18-month-old Malawian children in a randomized controlled trial. J. Nutr..

[100] Martinez-Estevez N.S., Alvarez-Guevara A.N., Rodriguez-Martinez C.E. (2016). Effects of zinc supplementation in the prevention of respiratory tract infections and diarrheal disease in Colombian children: A 12-month randomised controlled trial. Allergol. Immunopathol. (Madr.).

[101] Massaga J.J., Kitua A.Y., Lemnge M.M., Akida J.A., Malle L.N., Ronn A.M., Theander T.G., Bygbjerg I.C. (2003). Effect of intermittent treatment with amodiaquine on anaemia and malarial fevers in infants in Tanzania: A randomised placebo-controlled trial. Lancet.

[102] Matias S.L., Vargas-Vasquez A., Bado Perez R., Alcazar Valdivia L., Aquino Vivanco O., Rodriguez Martin A., Novalbos Ruiz J.P. (2017). Effects of lipid-based nutrient supplements v. micronutrient powders on nutritional and developmental outcomes among Peruvian infants. Public Health Nutr..

[103] Mazariegos M., Hambidge K.M., Westcott J.E., Solomons N.W., Raboy V., Das A., Goco N., Kindem M., Wright L.L., Krebs N.F. (2010). Neither a zinc supplement nor phytate-reduced maize nor their combination enhance growth of 6- to 12-month-old Guatemalan infants. J. Nutr..

[104] McDonald C.M., Manji K.P., Kisenge R., Aboud S., Spiegelman D., Fawzi W.W., Duggan C.P. (2015). Daily Zinc but Not Multivitamin Supplementation Reduces Diarrhea and Upper Respiratory Infections in Tanzanian Infants: A Randomized, Double-Blind, Placebo-Controlled Clinical Trial. J. Nutr..

[105] Menendez C., Kahigwa E., Hirt R., Vounatsou P., Aponte J.J., Font F., Acosta C.J., Schellenberg D.M., Galindo C.M., Kimario J. (1997). Randomised placebo-controlled trial of iron supplementation and malaria chemoprophylaxis for prevention of severe anaemia and malaria in Tanzanian infants. Lancet.

[106] Menon P., Ruel M.T., Loechl C.U., Arimond M., Habicht J.P., Pelto G., Michaud L. (2007). Micronutrient Sprinkles reduce anemia among 9- to 24-mo-old children when delivered through an integrated health and nutrition program in rural Haiti. J. Nutr..

[107] Mitra A.K., Akramuzzaman S.M., Fuchs G.J., Rahman M.M., Mahalanabis D. (1997). Long-term oral supplementation with iron is not harmful for young children in a poor community of Bangladesh. J. Nutr..

[108] Muller O., Becher H., van Zweeden A.B., Ye Y., Diallo D.A., Konate A.T., Gbangou A., Kouyate B., Garenne M. (2001). Effect of zinc supplementation on malaria and other causes of morbidity in west African children: Randomised double blind placebo controlled trial. BMJ.

[109] Newton S., Owusu-Agyei S., Asante K.P., Amoaful E., Mahama E., Tchum S.K., Ali M., Adjei K., Davis C.R., Tanumihardjo S.A. (2016). Vitamin A status and body pool size of infants before and after consuming fortified home-based complementary foods. Arch. Public Health.

[110] Northrop-Clewes C.A., Paracha P.I., McLoone U.J., Thurnham D.I. (1996). Effect of improved vitamin A status on response to iron supplementation in Pakistani infants. Am. J. Clin. Nutr..

[111] Oelofse A., Van Raaij J.M., Benade A.J., Dhansay M.A., Tolboom J.J., Hautvast J.G. (2003). The effect of a micronutrient-fortified complementary food on micronutrient status, growth and development of 6- to 12-month-old disadvantaged urban South African infants. Int. J. Food Sci. Nutr..

[112] Ogunlade A.O., Kruger H.S., Jerling J.C., Smuts C.M., Covic N., Hanekom S.M., Mamabolo R.L., Kvalsvig J. (2011). Point-of-use micronutrient fortification: Lessons learned in implementing a preschool-based pilot trial in South Africa. Int. J. Food Sci. Nutr..

[113] Ouedraogo H.Z., Traore T., Zeba A.N., Dramaix-Wilmet M., Hennart P., Donnen P. (2010). Effect of an improved local ingredient-based complementary food fortified or not with iron and selected multiple micronutrients on Hb concentration. Public Health Nutr..

[114] Owino V.O., Kasonka L.M., Sinkala M.M., Wells J.K., Eaton S., Darch T., Coward A., Tomkins A.M., Filteau S.M. (2007). Fortified complementary foods with or without alpha-amylase treatment increase hemoglobin but do not reduce breast milk intake of 9-mo-old Zambian infants. Am. J. Clin. Nutr..

[115] Owusu-Agyei S., Newton S., Mahama E., Febir L.G., Ali M., Adjei K., Tchum K., Alhassan L., Moleah T., Tanumihardjo S.A. (2013). Impact of vitamin A with zinc supplementation on malaria morbidity in Ghana. Nutr. J..

[116] Paganini D., Uyoga M.A., Kortman G.A.M., Cercamondi C.I., Moretti D., Barth-Jaeggi T., Schwab C., Boekhorst J., Timmerman H.M., Lacroix C. (2017). Prebiotic galacto-oligosaccharides mitigate the adverse effects of iron fortification on the gut microbiome: A randomised controlled study in Kenyan infants. Gut.

[117] Palupi L., Schultink W., Achadi E., Gross R. (1997). Effective community intervention to improve hemoglobin status in preschoolers receiving once-weekly iron supplementation. Am. J. Clin. Nutr..

[118] Phuka J.C., Maleta K., Thakwalakwa C., Cheung Y.B., Briend A., Manary M.J., Ashorn P. (2008). Complementary feeding with fortified spread and incidence of severe stunting in 6- to 18-month-old rural Malawians. Arch. Pediatr. Adolesc. Med..

[119] Radhakrishna K.V., Hemalatha R., Geddam J.J., Kumar P.A., Balakrishna N., Shatrugna V. (2013). Effectiveness of zinc supplementation to full term normal infants: A community based double blind, randomized, controlled, clinical trial. PLoS ONE.

[120] Rahman M.M., Akramuzzaman S.M., Mitra A.K., Fuchs G.J., Mahalanabis D. (1999). Long-term supplementation with iron does not enhance growth in malnourished Bangladeshi children. J. Nutr..

[121] Rahman M.M., Mahalanabis D., Alvarez J.O., Wahed M.A., Islam M.A., Habte D., Khaled M.A. (1996). Acute respiratory infections prevent improvement of vitamin A status in young infants supplemented with vitamin A. J. Nutr..

[122] Rahman M.M., Vermund S.H., Wahed M.A., Fuchs G.J., Baqui A.H., Alvarez J.O. (2001). Simultaneous zinc and vitamin A supplementation in Bangladeshi children: Randomised double blind controlled trial. BMJ.

[123] Rim H., Kim S., Sim B., Gang H., Kim H., Kim Y., Kim R., Yang M., Kim S. (2008). Effect of iron fortification of nursery complementary food on iron status of infants in the DPRKorea. Asia Pac. J. Clin. Nutr..

[124] Rivera J.A., Gonzalez-Cossio T., Flores M., Romero M., Rivera M., Tellez-Rojo M.M., Rosado J.L., Brown K.H. (2001). Multiple micronutrient supplementation increases the growth of Mexican infants. Am. J. Clin. Nutr..

[125] Rosado J.L., Lopez P., Munoz E., Martinez H., Allen L.H. (1997). Zinc supplementation reduced morbidity, but neither zinc nor iron supplementation affected growth or body composition of Mexican preschoolers. Am. J. Clin. Nutr..

[126] Rosado J.L., Lopez P., Garcia O.P., Alatorre J., Alvarado C. (2011). Effectiveness of the nutritional supplement used in the Mexican Oportunidades programme on growth, anaemia, morbidity and cognitive development in children aged 12-24 months. Public Health Nutr..

[127] Ross D.A., Kirkwood B.R., Binka F.N., Arthur P., Dollimore N., Morris S.S., Shier R.P., Gyapong J.O., Smith P.G. (1995). Child morbidity and mortality following vitamin A supplementation in Ghana: Time since dosing, number of doses, and time of year. Am. J. Public Health.

[128] Sampaio D.L., Mattos A.P., Ribeiro T.C., Leite M.E., Cole C.R., Costa-Ribeiro H. (2013). Zinc and other micronutrients supplementation through the use of sprinkles: Impact on the occurrence of diarrhea and respiratory infections in institutionalized children. J. Pediatr. (Rio J.).

[129] Sazawal S., Black R.E., Bhan M.K., Jalla S., Sinha A., Bhandari N. (1997). Efficacy of zinc supplementation in reducing the incidence and prevalence of acute diarrhea—A community-based, double-blind, controlled trial. Am. J. Clin. Nutr..

[130] Sazawal S., Black R.E., Ramsan M., Chwaya H.M., Stoltzfus R.J., Dutta A., Dhingra U., Kabole I., Deb S., Othman M.K. (2006). Effects of routine prophylactic supplementation with iron and folic acid on admission to hospital and mortality in preschool children in a high malaria transmission setting: Community-based, randomised, placebo-controlled trial. Lancet.

[131] Sazawal S., Dhingra U., Dhingra P., Hiremath G., Kumar J., Sarkar A., Menon V.P., Black R.E. (2007). Effects of fortified milk on morbidity in young children in north India: Community based, randomised, double masked placebo controlled trial. BMJ.

[132] Sempertegui F., Estrella B., Camaniero V., Betancourt V., Izurieta R., Ortiz W., Fiallo E., Troya S., Rodriguez A., Griffiths J.K. (1999). The beneficial effects of weekly low-dose vitamin A supplementation on acute lower respiratory infections and diarrhea in Ecuadorian children. Pediatrics.

[133] Shamah-Levy T., Villalpando S., Rivera-Dommarco J.A., Mundo-Rosas V., Cuevas-Nasu L., Jimenez-Aguilar A. (2008). Ferrous gluconate and ferrous sulfate added to a complementary food distributed by the Mexican nutrition program Oportunidades have a comparable efficacy to reduce iron deficiency in toddlers. J. Pediatr. Gastroenterol. Nutr..

[134] Shankar A.H., Genton B., Semba R.D., Baisor M., Paino J., Tamja S., Adiguma T., Wu L., Rare L., Tielsch J.M. (1999). Effect of vitamin A supplementation on morbidity due to Plasmodium falciparum in young children in Papua New Guinea: A randomised trial. Lancet.

[135] Silva A.P., Vitolo M.R., Zara L.F., Castro C.F. (2006). Effects of zinc supplementation on 1- to 5-year old children. J. Pediatr. (Rio J.).

[136] Smuts C.M., Dhansay M.A., Faber M., van Stuijvenberg M.E., Swanevelder S., Gross R., Benade A.J. (2005). Efficacy of multiple micronutrient supplementation for improving anemia, micronutrient status, and growth in South African infants. J. Nutr..

[137] Stoltzfus R.J., Kvalsvig J.D., Chwaya H.M., Montresor A., Albonico M., Tielsch J.M., Savioli L., Pollitt E. (2001). Effects of iron supplementation and anthelmintic treatment on motor and language development of preschool children in Zanzibar: Double blind, placebo controlled study. BMJ.

[138] Surono I.S., Martono P.D., Kameo S., Suradji E.W., Koyama H. (2014). Effect of probiotic L. plantarum IS-10506 and zinc supplementation on humoral immune response and zinc status of Indonesian pre-school children. J. Trace Elem. Med. Biol..

[139] Taneja S., Strand T.A., Kumar T., Mahesh M., Mohan S., Manger M.S., Refsum H., Yajnik C.S., Bhandari N. (2013). Folic acid and vitamin B-12 supplementation and common infections in 6-30-mo-old children in India: A randomized placebo-controlled trial. Am. J. Clin. Nutr..

[140] Teshome E.M., Andang’o P.E.A., Osoti V., Terwel S.R., Otieno W., Demir A.Y., Prentice A.M., Verhoef H. (2017). Daily home fortification with iron as ferrous fumarate versus NaFeEDTA: A randomised, placebo-controlled, non-inferiority trial in Kenyan children. BMC Med..

[141] Thu B.D., Schultink W., Dillon D., Gross R., Leswara N.D., Khoi H.H. (1999). Effect of daily and weekly micronutrient supplementation on micronutrient deficiencies and growth in young Vietnamese children. Am. J. Clin. Nutr..

[142] Umeta M., West C.E., Haidar J., Deurenberg P., Hautvast J.G. (2000). Zinc supplementation and stunted infants in Ethiopia: A randomised controlled trial. Lancet.

[143] Untoro J., Karyadi E., Wibowo L., Erhardt M.W., Gross R. (2005). Multiple micronutrient supplements improve micronutrient status and anemia but not growth and morbidity of Indonesian infants: A randomized, double-blind, placebo-controlled trial. J. Nutr..

[144] Venkatarao T., Ramakrishnan R., Nair N.G., Radhakrishnan S., Sundaramoorthy L., Koya P.K., Kumar S.K. (1996). Effect of vitamin A supplementation to mother and infant on morbidity in infancy. Indian Pediatr..

[145] Warthon-Medina M., Qualter P., Zavaleta N., Dillon S., Lazarte F., Lowe N.M. (2015). The Long Term Impact of Micronutrient Supplementation during Infancy on Cognition and Executive Function Performance in Pre-School Children. Nutrients.

[146] Wasantwisut E., Winichagoon P., Chitchumroonchokchai C., Yamborisut U., Boonpraderm A., Pongcharoen T., Sranacharoenpong K., Russameesopaphorn W. (2006). Iron and zinc supplementation improved iron and zinc status, but not physical growth, of apparently healthy, breast-fed infants in rural communities of northeast Thailand. J. Nutr..

[147] Wessells K.R., Ouedraogo Z.P., Rouamba N., Hess S.Y., Ouedraogo J.B., Brown K.H. (2012). Short-term zinc supplementation with dispersible tablets or zinc sulfate solution yields similar positive effects on plasma zinc concentration of young children in Burkina Faso: A randomized controlled trial. J. Pediatr..

[148] Wieringa F.T., Dijkhuizen M.A., West C.E., Thurnham D.I., Van der Meer J.W. (2003). Redistribution of vitamin A after iron supplementation in Indonesian infants. Am. J. Clin. Nutr..

[149] Yurdakok K., Temiz F., Yalcin S.S., Gumruk F. (2004). Efficacy of daily and weekly iron supplementation on iron status in exclusively breast-fed infants. J. Pediatr. Hematol. Oncol..

[150] Zlotkin S., Antwi K.Y., Schauer C., Yeung G. (2003). Use of microencapsulated iron(II) fumarate sprinkles to prevent recurrence of anaemia in infants and young children at high risk. Bull. World Health Organ..

[151] Cobra C., Rusmil K., Rustama D., Suwardi S.S., Permaesih D., Martuti S., Semba R.D. (1997). Infant survival is improved by oral iodine supplementation. J. Nutr..

[152] Dibley M.J., Sadjimin T., Kjolhede C.L., Moulton L.H. (1996). Vitamin A supplementation fails to reduce incidence of acute respiratory illness and diarrhea in preschool-age Indonesian children. J. Nutr..

[153] Dijkhuizen M.A., Wieringa F.T., West C.E., Martuti S. (2001). Effects of iron and zinc supplementation in Indonesian infants on micronutrient status and growth. J. Nutr..

[154] Domellof M., Cohen R.J., Dewey K.G., Hernell O., Rivera L.L., Lonnerdal B. (2001). Iron supplementation of breast-fed Honduran and Swedish infants from 4 to 9 months of age. J. Pediatr..

[155] Dossa R.A., Ategbo E.A., de Koning F.L., van Raaij J.M., Hautvast J.G. (2001). Impact of iron supplementation and deworming on growth performance in preschool Beninese children. Eur. J. Clin. Nutr..

[156] Barffour M.A., Hinnouho G.M., Kounnavong S., Wessells K.R., Ratsavong K., Bounheuang B., Chanhthavong B., Sitthideth D., Sengnam K., Arnold C.D. (2019). Effects of Daily Zinc, Daily Multiple Micronutrient Powder, or Therapeutic Zinc Supplementation for Diarrhea Prevention on Physical Growth, Anemia, and Micronutrient Status in Rural Laotian Children: A Randomized Controlled Trial. J. Pediatr..

[157] Noor S., Ashraf S., Siddiqua S., Mushtaq S., Saleem M. (2018). To compare prophylactic zinc supplementation versus placebo in terms of frequency of diarrhea in infants of 6–11 months. Med. Forum Mon..

[158] Smuts C.M., Matsungo T.M., Malan L., Kruger H.S., Rothman M., Kvalsvig J.D., Covic N., Joosten K., Osendarp S.J.M., Bruins M.J. (2019). Effect of small-quantity lipid-based nutrient supplements on growth, psychomotor development, iron status, and morbidity among 6- to 12-mo-old infants in South Africa: A randomized controlled trial. Am. J. Clin. Nutr..

[159] Semba R.D., West K.P., Natadisastra G., Eisinger W., Lan Y., Sommer A. (2000). Hyporetinolemia and acute phase proteins in children with and without xerophthalmia. Am. J. Nutr..

[160] Villalpando S., Shamah T., Rivera J.A., Lara Y., Monterrubio E. (2006). Fortifying milk with ferrous gluconate and zinc oxide in a public nutrition program reduced the prevalence of anemia in toddlers. J. Nutr..

[161] Abdollahi M., Abdollahi Z., Fozouni F., Bondarianzadeh D. (2014). Oral Zinc Supplementation Positively Affects Linear Growth, But not Weight, in Children 6–24 Months of Age. Int. J. Prev. Med..

[162] Aboud F.E., Akhter S. (2011). A cluster-randomized evaluation of a responsive stimulation and feeding intervention in bangladesh. Pediatrics.

[163] Aburto N.J., Ramirez-Zea M., Neufeld L.M., Flores-Ayala R. (2010). The effect of nutritional supplementation on physical activity and exploratory behavior of Mexican infants aged 8–12 months. Eur. J. Clin. Nutr..

[164] Agarwal D.K., Pandey C.M., Agarwal K.N. (1995). Vitamin a administration and preschool child mortality. Nutr. Res..

[165] Alderman H., Ndiaye B., Linnemayr S., Ka A., Rokx C., Dieng K., Mulder-Sibanda M. (2009). Effectiveness of a community-based intervention to improve nutrition in young children in Senegal: A difference in difference analysis. Public Health Nutr..

[166] de Almeida C.A., De Mello E.D., Ramos A.P., Joao C.A., Joao C.R., Dutra-de-Oliveira J.E. (2014). Assessment of drinking water fortification with iron plus ascorbic Acid or ascorbic Acid alone in daycare centers as a strategy to control iron-deficiency anemia and iron deficiency: A randomized blind clinical study. J. Trop. Pediatr..

[167] Arcanjo F.P., Arcanjo C.C., Arcanjo F.C., Campos Lde A., Amancio O.M., Braga J.A. (2012). Milk-based cornstarch porridge fortified with iron is effective in reducing anemia: A randomized, double-blind, placebo-controlled trial. J. Trop. Pediatr..

[168] Attanasio O.P., Fernandez C., Fitzsimons E.O., Grantham-McGregor S.M., Meghir C., Rubio-Codina M. (2014). Using the infrastructure of a conditional cash transfer program to deliver a scalable integrated early child development program in Colombia: Cluster randomized controlled trial. BMJ.

[169] Awasthi S., Peto R., Read S., Clark S., Pande V., Bundy D., Team D. (2013). Vitamin A supplementation every 6 months with retinol in 1 million pre-school children in north India: DEVTA, a cluster-randomised trial. Lancet.

[170] Bagni U.V., Baiao M.R., Santos M.M., Luiz R.R., Veiga G.V. (2009). [Effect of weekly rice fortification with iron on anemia prevalence and hemoglobin concentration among children attending public daycare centers in Rio de Janeiro, Brazil]. Cad. Saude Publica.

[171] Barbosa T.N., Taddei J.A., Palma D., Ancona-Lopez F., Braga J.A. (2012). Double-blind randomized controlled trial of rolls fortified with microencapsulated iron. Rev. Assoc. Med. Bras. (1992).

[172] Batra P., Schlossman N., Balan I., Pruzensky W., Balan A., Brown C., Gamache M.G., Schleicher M.M., de Sa A.B., Saltzman E. (2016). A Randomized Controlled Trial Offering Higher- Compared with Lower-Dairy Second Meals Daily in Preschools in Guinea-Bissau Demonstrates an Attendance-Dependent Increase in Weight Gain for Both Meal Types and an Increase in Mid-Upper Arm Circumference for the Higher-Dairy Meal. J. Nutr..

[173] Baum A., Elize W., Jean-Louis F. (2017). Microfinance Institutions’ Successful Delivery Of Micronutrient Powders: A Randomized Trial In Rural Haiti. Health Aff. (Millwood).

[174] Becquey E., Ouedraogo C.T., Hess S.Y., Rouamba N., Prince L., Ouedraogo J.B., Vosti S.A., Brown K.H. (2016). Comparison of Preventive and Therapeutic Zinc Supplementation in Young Children in Burkina Faso: A Cluster-Randomized, Community-Based Trial. J. Nutr..

[175] Bougma K., Aboud F.E., Lemma T.M., Frongillo E.A., Marquis G.S. (2018). Introduction of iodised salt benefits infants’ mental development in a community-based cluster-randomised effectiveness trial in Ethiopia. Br. J. Nutr..

[176] Chen J., Zhao X., Zhang X., Yin S., Piao J., Huo J., Yu B., Qu N., Lu Q., Wang S. (2005). Studies on the effectiveness of NaFeEDTA-fortified soy sauce in controlling iron deficiency: A population-based intervention trial. Food Nutr. Bull..

[177] Chen L., Liu Y.F., Gong M., Jiang W., Fan Z., Qu P., Chen J., Liu Y.X., Li T.Y. (2012). Effects of vitamin A, vitamin A plus zinc, and multiple micronutrients on anemia in preschool children in Chongqing, China. Asia Pac. J. Clin. Nutr..

[178] Christian P., Shaikh S., Shamim A.A., Mehra S., Wu L., Mitra M., Ali H., Merrill R.D., Choudhury N., Parveen M. (2015). Effect of fortified complementary food supplementation on child growth in rural Bangladesh: A cluster-randomized trial. Int. J. Epidemiol..

[179] de Almeida C.A., Dutra-De-Oliveira J.E., Crott G.C., Cantolini A., Ricco R.G., Del Ciampo L.A., Baptista M.E. (2005). Effect of fortification of drinking water with iron plus ascorbic acid or with ascorbic acid alone on hemoglobin values and anthropometric indicators in preschool children in day-care centers in Southeast Brazil. Food Nutr. Bull..

[180] Dewey K.G., Mridha M.K., Matias S.L., Arnold C.D., Cummins J.R., Khan M.S., Maalouf-Manasseh Z., Siddiqui Z., Ullah M.B., Vosti S.A. (2017). Lipid-based nutrient supplementation in the first 1000 d improves child growth in Bangladesh: A cluster-randomized effectiveness trial. Am. J. Clin. Nutr..

[181] Glinz D., Hurrell R.F., Ouattara M., Zimmermann M.B., Brittenham G.M., Adiossan L.G., Righetti A.A., Seifert B., Diakite V.G., Utzinger J. (2015). The effect of iron-fortified complementary food and intermittent preventive treatment of malaria on anaemia in 12- to 36-month-old children: A cluster-randomised controlled trial. Malar. J..

[182] Hadler M.C., Sigulem D.M., Alves Mde F., Torres V.M. (2008). Treatment and prevention of anemia with ferrous sulfate plus folic acid in children attending daycare centers in Goiania, Goias State, Brazil: A randomized controlled trial. Cad. Saude Publica.

[183] Hess S.Y., Abbeddou S., Jimenez E.Y., Some J.W., Vosti S.A., Ouedraogo Z.P., Guissou R.M., Ouedraogo J.B., Brown K.H. (2015). Small-quantity lipid-based nutrient supplements, regardless of their zinc content, increase growth and reduce the prevalence of stunting and wasting in young burkinabe children: A cluster-randomized trial. PLoS ONE.

[184] Hettiarachchi M., Lekamwasam S., Liyanage C. (2010). Long-term cereal-based nutritional supplementation improved the total spine bone mineral density amongst Sri Lankan preschool children: A randomized controlled study. J. Pediatr. Endocrinol. Metab..

[185] Huybregts L., Houngbe F., Salpeteur C., Brown R., Roberfroid D., Ait-Aissa M., Kolsteren P. (2012). The effect of adding ready-to-use supplementary food to a general food distribution on child nutritional status and morbidity: A cluster-randomized controlled trial. PLoS Med..

[186] Isanaka S., Barnhart D.A., McDonald C.M., Ackatia-Armah R.S., Kupka R., Doumbia S., Brown K.H., Menzies N.A. (2019). Cost-effectiveness of community-based screening and treatment of moderate acute malnutrition in Mali. BMJ Glob. Health.

[187] Jack S.J., Ou K., Chea M., Chhin L., Devenish R., Dunbar M., Eang C., Hou K., Ly S., Khin M. (2012). Effect of micronutrient sprinkles on reducing anemia: A cluster-randomized effectiveness trial. Arch. Pediatr. Adolesc. Med..

[188] Le Port A., Bernard T., Hidrobo M., Birba O., Rawat R., Ruel M.T. (2017). Delivery of iron-fortified yoghurt, through a dairy value chain program, increases hemoglobin concentration among children 24 to 59 months old in Northern Senegal: A cluster-randomized control trial. PLoS ONE.

[189] Della Lucia C.M., Santos L.L.M., Silva B.P.d., Anunciação P.C., Alfenas R.d.C.G., Franceschini S.d.C.C., Martino H.S.D., Sant’Ana H.M.P. (2017). Impact of rice fortified with iron, zinc, thiamine and folic acid on laboratory measurements of nutritional status of preschool children. Ciência Saúde Coletiva.

[190] Lundeen E., Schueth T., Toktobaev N., Zlotkin S., Hyder S.M., Houser R. (2010). Daily use of Sprinkles micronutrient powder for 2 months reduces anemia among children 6 to 36 months of age in the Kyrgyz Republic: A cluster-randomized trial. Food Nutr. Bull..

[191] Luo R., Yue A., Zhou H., Shi Y., Zhang L., Martorell R., Medina A., Rozelle S., Sylvia S. (2017). The effect of a micronutrient powder home fortification program on anemia and cognitive outcomes among young children in rural China: A cluster randomized trial. BMC Public Health.

[192] Ma J., Sun Q., Liu J., Hu Y., Liu S., Zhang J., Sheng X., Hambidge K.M. (2016). The Effect of Iron Fortification on Iron (Fe) Status and Inflammation: A Randomized Controlled Trial. PLoS ONE.

[193] Nestel P., Nalubola R., Sivakaneshan R., Wickramasinghe A.R., Atukorala S., Wickramanayake T. (2004). The use of iron-fortified wheat flour to reduce anemia among the estate population in Sri Lanka. Int. J. Vitam. Nutr. Res..

[194] Nguyen X.N., Berger J., Dao T.Q., Nguyen C.K., Traissac P., Ha H.K. (2002). [Efficacy of daily and weekly iron supplementation for the control of iron deficiency anaemia in infants in rural Vietnam]. Sante.

[195] Nogueira Arcanjo F.P., Santos P.R., Arcanjo C.P., Amancio O.M., Braga J.A. (2012). Use of iron-fortified rice reduces anemia in infants. J. Trop. Pediatr..

[196] Osei A.K., Pandey P., Spiro D., Adhikari D., Haselow N., De Morais C., Davis D. (2015). Adding multiple micronutrient powders to a homestead food production programme yields marginally significant benefit on anaemia reduction among young children in Nepal. Matern. Child Nutr..

[197] Phu P.V., Hoan N.V., Salvignol B., Treche S., Wieringa F.T., Khan N.C., Tuong P.D., Berger J. (2010). Complementary foods fortified with micronutrients prevent iron deficiency and anemia in Vietnamese infants. J. Nutr..

[198] Rivera J.A., Shamah T., Villalpando S., Monterrubio E. (2010). Effectiveness of a large-scale iron-fortified milk distribution program on anemia and iron deficiency in low-income young children in Mexico. Am. J. Clin. Nutr..

[199] Sazawal S., Dhingra P., Dhingra U., Gupta S., Iyengar V., Menon V.P., Sarkar A., Black R.E. (2014). Compliance with home-based fortification strategies for delivery of iron and zinc: Its effect on haematological and growth markers among 6-24 months old children in north India. J. Health Popul. Nutr..

[200] Sharieff W., Yin S.A., Wu M., Yang Q., Schauer C., Tomlinson G., Zlotkin S. (2006). Short-term daily or weekly administration of micronutrient Sprinkles has high compliance and does not cause iron overload in Chinese schoolchildren: A cluster-randomised trial. Public Health Nutr..

[201] Sharma K., Parikh P., Desai F. (2011). Effect of daily versus weekly iron folic acid supplementation on the haemoglobin levels of children 6 to 36 months of urban slums of Vadodara. Indian J. Community Med..

[202] Siega-Riz A.M., Estrada Del Campo Y., Kinlaw A., Reinhart G.A., Allen L.H., Shahab-Ferdows S., Heck J., Suchindran C.M., Bentley M.E. (2014). Effect of supplementation with a lipid-based nutrient supplement on the micronutrient status of children aged 6–18 months living in the rural region of Intibuca, Honduras. Paediatr. Perinat. Epidemiol..

[203] Somasse Y.E., Dramaix M., Traore B., Ngabonziza I., Toure O., Konate M., Diallo M., Donnen P. (2018). The WHO recommendation of home fortification of foods with multiple-micronutrient powders in children under 2 years of age and its effectiveness on anaemia and weight: A pragmatic cluster-randomized controlled trial. Public Health Nutr..

[204] Soofi S., Cousens S., Iqbal S.P., Akhund T., Khan J., Ahmed I., Zaidi A.K., Bhutta Z.A. (2013). Effect of provision of daily zinc and iron with several micronutrients on growth and morbidity among young children in Pakistan: A cluster-randomised trial. Lancet.

[205] Tielsch J.M., Khatry S.K., Stoltzfus R.J., Katz J., LeClerq S.C., Adhikari R., Mullany L.C., Shresta S., Black R.E. (2006). Effect of routine prophylactic supplementation with iron and folic acid on preschool child mortality in southern Nepal: Community-based, cluster-randomised, placebo-controlled trial. Lancet.

[206] Varma J.L., Das S., Sankar R., Mannar M.G., Levinson F.J., Hamer D.H. (2007). Community-level micronutrient fortification of a food supplement in India: A controlled trial in preschool children aged 36–66 months. Am. J. Clin. Nutr..

[207] Vijay J., Sharma S. (2014). Impact of micronutrients sprinkle on weight and height of children aged 6–36 months in Tonk district of Rajasthan state. Indian J Community Health.

[208] West K.P., Katz J., Shrestha S.R., LeClerq S.C., Khatry S.K., Pradhan E.K., Adhikari R., Wu L.S., Pokhrel R.P., Sommer A. (1995). Mortality of infants < 6 mo of age supplemented with vitamin A: A randomized, double-masked trial in Nepal. Am. J. Clin. Nutr..

[209] Zlotkin S., Newton S., Aimone A.M., Azindow I., Amenga-Etego S., Tchum K., Mahama E., Thorpe K.E., Owusu-Agyei S. (2013). Effect of iron fortification on malaria incidence in infants and young children in Ghana: A randomized trial. JAMA.

[210] Arcanjo F.P.N., da Costa Rocha T.C., Arcanjo C.P.C., Santos P.R. (2019). Micronutrient Fortification at Child-Care Centers Reduces Anemia in Young Children. J. Diet. Suppl..

[211] Galasso E., Weber A.M., Stewart C.P., Ratsifandrihamanana L., Fernald L.C.H. (2019). Effects of nutritional supplementation and home visiting on growth and development in young children in Madagascar: A cluster-randomised controlled trial. Lancet Glob. Health.

[212] Ghosh S.A., Strutt N.R., Otoo G.E., Suri D.J., Ankrah J., Johnson T., Nsiah P., Furuta C., Murakami H., Perera G. (2019). A macro- and micronutrient-fortified complementary food supplement reduced acute infection, improved haemoglobin and showed a dose-response effect in improving linear growth: A 12-month cluster randomised trial. J. Nutr. Sci..

[213] Larson L.M., Young M.F., Bauer P.J., Mehta R., Girard A.W., Ramakrishnan U., Verma P., Chaudhuri I., Srikantiah S., Martorell R. (2018). Effectiveness of a home fortification programme with multiple micronutrients on infant and young child development: A cluster-randomised trial in rural Bihar, India. Br. J. Nutr..

[214] Suchdev P.S., Ruth L.J., Woodruff B.A., Mbakaya C., Mandava U., Flores-Ayala R., Jefferds M.E., Quick R. (2012). Selling Sprinkles micronutrient powder reduces anemia, iron deficiency, and vitamin A deficiency in young children in Western Kenya: A cluster-randomized controlled trial. Am. J. Clin. Nutr..

[215] Luby S.P., Rahman M., Arnold B.F., Unicomb L., Ashraf S., Winch P.J., Stewart C.P., Begum F., Hussain F., Benjamin-Chung J. (2018). Effects of water quality, sanitation, handwashing, and nutritional interventions on diarrhoea and child growth in rural Bangladesh: A cluster randomised controlled trial. Lancet Glob. Health.

[216] Null C., Stewart C.P., Pickering A.J., Dentz H.N., Arnold B.F., Arnold C.D., Benjamin-Chung J., Clasen T., Dewey K.G., Fernald L.C.H. (2018). Effects of water quality, sanitation, handwashing, and nutritional interventions on diarrhoea and child growth in rural Kenya: A cluster-randomised controlled trial. Lancet Glob. Health.

[217] Olney D.K., Leroy J., Bliznashka L., Ruel M.T. (2018). PROCOMIDA, a Food-Assisted Maternal and Child Health and Nutrition Program, Reduces Child Stunting in Guatemala: A Cluster-Randomized Controlled Intervention Trial. J. Nutr..

[218] Abdelrazik N., Al-Haggar M., Al-Marsafawy H., Abdel-Hadi H., Al-Baz R., Mostafa A.H. (2007). Impact of long-term oral iron supplementation in breast-fed infants. Indian J. Pediatr..

[219] Assis A.M.O., Santos L.M.P., Prado M.d.S., Martins M.C., Barreto M.L. (2000). Tolerance of vitamin A application associated with mass immunization of children in Northeast Brazil. Cad. Saude Publica.

[220] Cardoso M.A., Augusto R.A., Bortolini G.A., Oliveira C.S., Tietzman D.C., Sequeira L.A., Hadler M.C., Peixoto Mdo R., Muniz P.T., Vitolo M.R. (2016). Effect of Providing Multiple Micronutrients in Powder through Primary Healthcare on Anemia in Young Brazilian Children: A Multicentre Pragmatic Controlled Trial. PLoS ONE.

[221] Lutter C.K., Rodriguez A., Fuenmayor G., Avila L., Sempertegui F., Escobar J. (2008). Growth and micronutrient status in children receiving a fortified complementary food. J. Nutr..

[222] Monteiro C.A., Szarfarc S.C., Brunken G.S., Gross R., Conde W.L. (2001). Long-term preventive mass prescription of weekly doses of iron sulfate may be highly effective to reduce endemic child anemia. Rev. Bras. Epidemiol..

[223] Muslihah N., Khomsan A., Briawan D., Riyadi H. (2016). Complementary food supplementation with a small-quantity of lipid-based nutrient supplements prevents stunting in 6–12-month-old infants in rural West Madura Island, Indonesia. Asia Pac. J. Clin. Nutr..

[224] Nair V.K.M., Balamurugan A.S. (2017). Effect of iron prophylaxis in preventing iron deficiency anaemia in term infants. J. Evol. Med. Dent. Sci..

[225] Oliveira C.S., Sampaio P., Muniz P.T., Cardoso M.A., Group E.W. (2016). Multiple micronutrients in powder delivered through primary health care reduce iron and vitamin A deficiencies in young Amazonian children. Public Health Nutr..

[226] Akrour-Aissou C., Dupre T., Grangaud J.P., Assami M.K. (2019). Impact of vitamin D supplementation model on the circulating levels of 25 (OH) D in Algerian children aged 1-23 months. J. Steroid Biochem. Mol. Biol..

[227] Lopez de Romana D., Verona S., Vivanco O.A., Gross R. (2006). Protective effect of multimicronutrient supplementation against anemia among children, women, and adolescent girls in lower-income areas of Chiclayo, Peru. Food Nutr. Bull..

[228] Rooze S., Mathieu F., Claus W., Yangzom T., Yangzom D., Goyens P., de Maertelaer V. (2016). Effect of calcium and vitamin D on growth, rickets and Kashin-Beck disease in 0- to 5-year-old children in a rural area of central Tibet. Trop. Med. Int. Health.

[229] Acharya Y. (2018). The impact of vitamin A supplementation in childhood on adult outcomes: An exploration of mechanisms, timing of exposure, and heterogeneous effects. Soc. Sci. Med..

[230] Swami H.M., Thakur J.S., Bhatia S.P. (2007). Impact of mass supplementation of vitamin A. Indian J. Pediatr..

[231] Yadav R.J., Pandey A., Singh P. (2012). An evaluation of the ICDS food fortification in Uttarakhand. Indian J. Community Health.

[232] Lopez Boo F., Palloni G., Urzua S. (2014). Cost-benefit analysis of a micronutrient supplementation and early childhood stimulation program in Nicaragua. Ann. N. Y. Acad. Sci..

[233] Samuel A., Brouwer I.D., Feskens E.J.M., Adish A., Kebede A., De-Regil L.M., Osendarp S.J.M. (2018). Effectiveness of a Program Intervention with Reduced-Iron Multiple Micronutrient Powders on Iron Status, Morbidity and Growth in Young Children in Ethiopia. Nutrients.

[234] DeLong G.R., Leslie P.W., Wang S.H., Jiang X.M., Zhang M.L., Rakeman M., Jiang J.Y., Ma T., Cao X.Y. (1997). Effect on infant mortality of iodination of irrigation water in a severely iodine-deficient area of China. Lancet.

[235] Bloem M.W., Hye A., Wijnroks M., Ralte A., West K.P., Sommer A. (1995). The role of universal distribution of vitamin A capsules in combatting vitamin A deficiency in Bangladesh. Am. J. Epidemiol..

[236] Gebremedhin S. (2014). Effect of a single high dose vitamin A supplementation on the hemoglobin status of children aged 6-59 months: Propensity score matched retrospective cohort study based on the data of Ethiopian Demographic and Health Survey 2011. BMC Pediatr.

[237] Fisker A.B., Aaby P., Rodrigues A., Frydenberg M., Bibby B.M., Benn C.S. (2011). Vitamin A supplementation at birth might prime the response to subsequent vitamin A supplements in girls. Three year follow-up of a randomized trial. PLoS ONE.

[238] Gogia S., Sachdev H.S. (2009). Neonatal vitamin A supplementation for prevention of mortality and morbidity in infancy: Systematic review of randomised controlled trials. BMJ.

[239] Black M.M., Baqui A.H., Zaman K., Ake Persson L., El Arifeen S., Le K., McNary S.W., Parveen M., Hamadani J.D., Black R.E. (2004). Iron and zinc supplementation promote motor development and exploratory behavior among Bangladeshi infants. Am. J. Clin. Nutr..

[240] Lind T., Lonnerdal B., Stenlund H., Gamayanti I.L., Ismail D., Seswandhana R., Persson L.A. (2004). A community-based randomized controlled trial of iron and zinc supplementation in Indonesian infants: Effects on growth and development. Am. J. Clin. Nutr..

[241] Pongcharoen T., DiGirolamo A.M., Ramakrishnan U., Winichagoon P., Flores R., Martorell R. (2011). Long-term effects of iron and zinc supplementation during infancy on cognitive function at 9 y of age in northeast Thai children: A follow-up study. Am. J. Clin. Nutr..

[242] Pasricha S.R., Hayes E., Kalumba K., Biggs B.A. (2013). Effect of daily iron supplementation on health in children aged 4-23 months: A systematic review and meta-analysis of randomised controlled trials. Lancet Glob. Health.

[243] Low M., Farrell A., Biggs B.A., Pasricha S.R. (2013). Effects of daily iron supplementation in primary-school-aged children: Systematic review and meta-analysis of randomized controlled trials. CMAJ.

[244] Thompson J., Biggs B.A., Pasricha S.R. (2013). Effects of daily iron supplementation in 2- to 5-year-old children: Systematic review and meta-analysis. Pediatrics.

[245] WHO (2016). Guideline: Daily Iron Supplementation in Infants and Children.

