# Supplementary material for: Micronutrient Supplementation and Fortification Interventions on Health and Development Outcomes among Children Under-Five in Low- and Middle-Income Countries: A Systematic Review and Meta-Analysis
Source: Nutrients. 2020 Jan 21;12(2):289. doi: 10.3390/nu12020289 (PMC7071447; doi:10.3390/nu12020289)

Appendix 1 – MEDLINE Search Strategy

Population:

1. exp infant/ or exp child/ or exp newborn/ or exp child,preschool/ or exp pediatrics/ or (infant* or neonat* or baby* or babies or child*or newborn* or "newborn infant*" or girl* or boy* or toddler* or preschool* or "pre school*" or pre-school* or "preschool child*" or kindergarten* or kindergarden* or "nursery school" or "day care*" NOT adult* or paediatric* or pediatric* or under-five* or "under five*" or "under 5" or under-5 or "5 and under" or "five and under" or "under the age of five" or "under the age of 5").tw,kf

Single or Multiple Micronutrient Supplementation:

2. Micronutrients/ or vitamins/ or minerals/ or exp iron/ or exp iron compounds/ or iron, dietary/ or vitamin A/ or exp iodine/ or exp zinc or exp zinc compounds/ or exp vitamin D/ or (micronutrient* or multinutrient* or multi-nutrient* or "multi*nutrient" or "multimicro-nutrient*" or "multimicronutrient*" or multivitamin* or "multi-vitamin*" or multimineral* or "multi-mineral*" or MMN or "multiple micro nutrient*" or "multiple micronutrient" or micro-nutrient* or "essential vitamins*" or minerals* or "vitamin d" or "hydroxyvitamin d" or vitamin-d or "25 hydroxyvitamin d" or "25 hydroxy-vitamin d" or "25-hydroxyvitamin d" or "25-hydroxy-vitamin d" or "25-hydroxyvitamin d" or 25ohd or "25-oh-vitamin d" or 25-ohd or "vitamin d2" or vitamin-d2 or "25-hydroxyvitamin d2" or "25-hydroxy-vitamin d2" or "vitamin d3" or vitamin-d3 or "25 hydroxyvitamin d3" or "25 hydroxyvitamin d3" or "25-hydroxy-vitamin d3" or calcidiol or calcifediol or "vitamin a" or "vitamin a1" or retinol* or retinal* or Retinaldehyde or retinoid or Retinoids or retinoic or beta-carotene or "beta carotene" or iron or ferr* compounds or "dietary iron" or zinc or "zn" or "zinc acetate" or "zn acetate" or "zn sulfate" or "zn oxide" or iodine or "iod* compounds" or "ferr* compounds" or "dietary iron").tw,kf
3. Dietary supplements/ or tablets/ or ((supplement* or neutraceutical* or nutraceutical* or nutriceutical* or tablet* or syrup* or drop*).tw,kf)
4. 1 AND 2 AND 3

Lipid-Nutrient Supplementation:

5. exp Lipids/ or omega-3/ or (lipid* or soy* or peanut* or whey* or sesame* or cashew* or chickpea* or oils or protein* or butter* or fat* or "alpha-linolenic acid" or "docosahexaenoic acids" or "eicosapentaeonic acid").tw,kf
6. Dietary supplements/ or (("lipid based" or "lipid-based nutri*" or enrich* or emuls* or powder* or spread* or paste* or LNS*1 or iLiNS or supplement* or neutraceutical* or nutraceutical* or nutriceutical* or Nutributter* or Plumpy* or PlumpyNut or "ready to use" or "ready-to-use therapeutic food" or RUSF or RUTF).tw,kf)
7. 1 AND 5 AND 6

Large-Scale Fortification:

8. exp Food/ or "Food Supply"/ or exp "Agricultural Crops"/ or exp Salts/ or exp "Fish Products"/ or exp "Soy Foods" or exp Cereals/ or exp "Dietary Carbohydrates"/ or exp Oryza/ or exp Milk/ or exp Bread/ or exp Beverages/ or exp Yogurt/ or exp Margarine/ or exp Cheese/ or exp "Zea mays"/ or exp Condiments/ or exp Triticum/ or exp Spices/ or exp "Dietary Fats"/ or exp "Dairy Products"/ or (Food* or "staple foods" or crop* or flour* or salt* or sauce*or cereal* or sugar* or rice* or milk* or bread* or oil* or beverage* or yogurt* or margarine* or cheese* or maize*or corn* or wheat* or "durum wheat*" or "sorghum vulgare" or sorghum or condiment* or spices* or "curry powder*" or fat* or dairy*).tw,kf

9. Micronutrients/ or vitamins/ or minerals/ or exp iron/ or exp iron compounds/ or iron, dietary/or vitamin A/ or exp iodine/ or exp zinc/ or exp zinc compounds/ or exp vitamin D/ or exp folic acid/ or (micronutrient* or multinutrient* or multi-nutrient* or "multi*nutrient" or "multimicro-nutrient*" or "multimicronutrient*" or multivitamin* or "multi-vitamin*" or multimineral* or "multi-mineral*" or MMN or "multiple micro nutrient*" or "multiple micronutrient" or micro-nutrient* or "essential vitamins*" or minerals* or "vitamin d" or "hydroxyvitamin d" or vitamin-d or "25 hydroxyvitamin d" or "25 hydroxy-vitamin d" or "25-hydroxyvitamin d" or "25-hydroxy-vitamin d" or "25-hydroxyvitamin d" or 25ohd or "25-oh-vitamin d" or 25-ohd or "vitamin d2" or vitamin-d2 or "25-hydroxyvitamin d2" or "25 hydroxy-vitamin d2" or "vitamin d3" or vitamind3 or "25 hydroxyvitamin d3" or "25 hydroxyvitamin d3" or "25-hydroxy-vitamin d3" or calcidiol or calcifediol or "vitamin a" or "vitamin a1" or retinol* or retinal* or Retinaldehyde or retinoid or Retinoids or retinoic or beta-carotene or "beta carotene" or iron or ferr* compounds or "dietary iron" or zinc or "zn" or "zinc acetate" or "zn acetate" or "zn sulfate" or "zn oxide" or iodine or "iod* compounds" or "ferr* compounds" or "dietary iron" or "folic acid" or folate*).tw,kf.

10. ("supplemented food*" or "large scale fortif*" or enrich* or fortif*).tw,kf.

11. 1 AND 8 AND 9 AND 10

Targeted Fortification for Infants and Young Children:

12. exp "infant food"/ or milk substitutes/ or infant formula/ or soy milk/ or ("baby formula" or "artificial milk" or "complementary food*" or "blended food*" or "infant cereal" or "baby food*" or milk* or cereal* or porridge* or paste*).tw,kf

13. ("targeted fortification" or enrich* or fortif* or "supplemented food*").tw,kf

14. 1 AND 12 AND 13

Point-Of-Use Fortification with Micronutrient Powders:

15. Powders/ or vitamins/ or minerals/ or micronutrients/ or ("sprinkles powder" or Sprinkle* or powder* or foodlet* or "foodlet-based" or "crushable nutritabs" or "micronutrient powder" or "multiple micronutrient powder" or mnp).tw,kf

16. ("Point-of-use" or "home fortification" or "food fortif*" or enrich* or fortif*).tw,kf
17. 1 AND 15 AND 16
18. Developing Countries/ or ("developing country" or "developing countries" or "developing nation" or "developing nations" or "developing population" or "developing populations" or "developing world" or "less developed country" or "less developed countries" or "less developed nation" or "less developed nations" or "less developed population" or "less developed populations" or "less developed world" or "lesser developed country" or "lesser developed countries" or "lesser developed nation" or "lesser developed nations" or "lesser developed population" or "lesser developed populations" or "lesser developed world" or "under developed country" or "under developed countries" or "under developed nation" or "under developed nations" or "under developed population" or "under developed populations" or "under developed world" or "underdeveloped country" or "underdeveloped countries" or "underdeveloped nation" or "underdeveloped nations" or "underdeveloped population" or "underdeveloped populations" or "underdeveloped world" or "middle income country" or "middle income countries" or "middle income nation" or "middle income nations" or "middle income population" or "middle income populations" or "low income country" or "low income countries" or "low income nation" or "low income nations" or "low income population" or "low income populations" or "lower income country" or "lower income countries" or "lower income nation" or "lower income nations" or "lower income population" or "lower income populations" or "underserved country" or "underserved countries" or "underserved nation" or "underserved nations" or "underserved population" or "underserved populations" or "underserved world" or "under served country" or "under served countries" or "under served nation" or "under served nations" or "under served population" or "under served populations" or "under served world" or "deprived country" or "deprived countries" or "deprived nation" or "deprived nations" or "deprived population" or "deprived populations" or "deprived world" or "poor country" or "poor countries" or "poor nation" or "poor nations" or "poor population" or "poor populations" or "poor world" or "poorer country" or "poorer countries" or "poorer nation" or "poorer nations" or "poorer population" or "poorer populations" or "poorer world" or "developing economy" or "developing economies" or "less developed economy" or "less developed economies" or "lesser developed economy" or "lesser developed economies" or "under developed economy" or "under developed economies" or "underdeveloped economy" or "underdeveloped economies" or "middle income economy" or "middle income economies" or "low income economy" or "low income economies" or "lower income economy" or "lower income economies" or "low gdp" or "low gnp" or "low gross domestic" or "low gross national" or "lower gdp" or "lower gnp" or "lower gross domestic" or "lower gross national" or lmic or lmics or "third world" or "lami country" or "lami countries" or "transitional country" or "transitional countries" or Africa or Asia or "Caribbean Region" or

"West Indies" or "South America" or "Latin America" or "Central America" or Afghanistan or Albania or Algeria or Angola or Argentina or Armenia or Armenian or Azerbaijan or Bangladesh or Benin or Byelarus or Byelorussian or Belarus or Belorussian or Belorussia or Belize or Bhutan or Bolivia or Bosnia or Herzegovina or Hercegovina or Botswana or Brazil or Bulgaria or "Burkina Faso" or "Burkina Fasso" or Burundi or Urundi or Cambodia or "Khmer Republic" or Kampuchea or Cameroon or Cameroons or Cameron or Camerons or "Cape Verde" or "Central African Republic" or Chad or China or Colombia or Comoros or "Comoro Islands" or Comores or Mayotte or Congo or Zaire or "Costa Rica" or "Cote d'Ivoire" or "Ivory Coast" or Cuba or Djibouti or "French Somaliland" or Dominica or "Dominican Republic" or "East Timor" or "East Timur" or "Timor Leste" or Ecuador or Egypt or "El Salvador" or Eritrea or Ethiopia or Fiji or Gabon or "Gabonese Republic" or Gambia or Gaza or "Georgia Republic" or "Georgian Republic" or Ghana or "Gold Coast" or Grenada or Guatemala or Guinea or Guiana or Guyana or Haiti or Honduras or India or Maldives or Indonesia or Iran or Iraq or "Isle of Man" or Jamaica or Jordan or Kazakhstan or Kazakh or Kenya or Kiribati or Korea or Kosovo or Kyrgyzstan or Kirghizia or "Kyrgyz Republic" or Kirghiz or Kirgizstan or "Lao PDR" or Laos or Lebanon or Lesotho or Basutoland or Liberia or Libya or Macedonia or Madagascar or "Malagasy Republic" or Malaysia or Malaya or Malay or Sabah or Sarawak or Malawi or Nyasaland or Mali or "Marshall Islands" or Mauritania or Mauritius or Mexico or Micronesia or "Middle East" or Moldova or Moldovia or Moldovian or Mongolia or Montenegro or Morocco or Mozambique or Myanmar or Myanma or Burma or Namibia or Nepal or Nicaragua or Niger or Nigeria or Pakistan or Palau or Palestine or Panama or Paraguay or Peru or Philippines or Philipines or Phillipines or Phillippines or Romania or Rumania or Roumania or Russia or Russian or Rwanda or Ruanda or "Saint Lucia" or "St Lucia" or "Saint Vincent" or "St Vincent" or Grenadines or Samoa or "Samoan Islands" or "Navigator Island" or "Navigator Islands" or "Sao Tome" or Senegal or Serbia or Montenegro or Seychelles or "Sierra Leone" or "Sri Lanka" or Ceylon or "Solomon Islands" or Somalia or Sudan or Suriname or Surinam or Swaziland or Syria or Tajikistan or Tadzhikistan or Tadjikistan or Tadzhik or Tanzania or Thailand or Togo or "Togolese Republic" or Tonga or Tunisia or Turkey or Turkmenistan or Turkmen or Uganda or Ukraine or USSR or "Soviet Union" or "Union of Soviet Socialist Republics" or Uzbekistan or Uzbek or Vanuatu or Venezuela or Vietnam or "Viet Nam" or "West Bank" or Yemen or Yugoslavia or Zambia or Zimbabwe or Rhodesia).tw,kf

19. 4 OR 7 OR 11 OR 14 OR 17
20. 19 AND 18
21. Limit 20 to yr="1995-2017"

Append[illegible]2 – Eligible Studies Excluded from Meta-Analyses

First author, year	Study design	Country	Age range enrolled	Total study population	Intervention	Intervention dose and formulation	Intervention duration and frequency	Control	Control dose and formulation
**Abdelrazik, 2007**	Non-RCT	Egypt	4-6 months	248	MMN (with iron) supplementation	5mL of syrup: vitamin A (1200IU), vitamin D3 (100IU), thiamin (1mg), riboflavin (1mg), pyridoxine (0.5mg), vitamin C (50mg), vitamin E (1mg), nicotinamide (5mg), panthenol (2mg), calcium gluconate and phosphate (50mg), ferrous gluconate (43mg eq to elemental iron 5mg), 1 mg/kg iron - adjusted monthly according to infant weight	Daily for 12 months	MMN (without iron) supplementatio n	5ml of syrup: vitamin A (1200IU), D3 (100IU), thiamin (1mg), riboflavin (1mg), pyridoxine (0.5mg), vitamin C (50mg), vitamin E (1mg), nicotinamide (5mg), panthenol (2mg), calcium gluconate and phosphate (50mg)
**Abdollahi, 2014**	cRCT	Iron	6-24 months	838 (17 clusters)	Zinc supplementation	5 mL of syrup: zinc (5mg)	Daily for 3 months	No intervention	
**Aburto, 2010**	cRCT	Mexico	4-12 months	187	MNP, MMN supplementation or fortified food group	MNP and MMN (syrup): iron (10mg), zinc (10mg), vitamin A (400mcg), vitamin E (6mg), vitamin C (50mg), vit B2 (0.8mg), vitamin B12 (0.7mcg), folic acid (50mcg) Fortified food: also contained energy (194kcal), protein (5.8g), fat (6.6g), carbohydrate (27.9g), sodium (24.5mg).	Daily for 4 months	No intervention	
**Acharya, 2018**	Natural experiment	Nepal	6-60 months		Vitamin A supplementation	High-dose capsule	Bi-annually	No intervention	
**Adom, 2010**	RCT	Ghana	6-18 months	56	Iron fortified maize-cowpea flour	184mg/kg dry weight	Daily (3 times a day) for 6 months	Unfortified maize-cowpea flour	
**Akrour-Aissou, 2019**	Non-RCT	Algeria	1-23 months	150	Vitamin D supplementation	Syrup: 200 000 IU at 1 month of age and 200 000 IU at 6 months of age	2 doses (one at 1 month of age and another at 6 months of age)	No intervention	
**Alderman, 2009**<br><br>**Associated refs:**	cRCT	Senegal	6-59 months	200,000 households (220 clusters)	Vitamin A supplementation	Syrup: children 6-11 months of age received 100,000IU and children 12-59 months of age received 200,000IU	Bi-annually for 2 years	No intervention	

**Linnemayr, 2011**									
**Almeida, 2014**	cRCT	Brazil	12-59 months	184	MNP (with iron only) fortified drinking water	10mg of iron and 100mg of ascorbic acid per liter water (the pre-mix contained 1000 mg of FeSO4-7H2O and 2000 mg of ascorbic acid diluted in 20 ml of water)	Daily for 3 months	Unfortified drinking water	
**Asibey-Berko, 2007**	RCT	Ghana	1-5 years	157	Iodine and iron fortified salt	1000ppm iron (1g/kg) and 50ppm (50mg/kg) iodine; average daily intake is 70mg/week	Daily for 8 months	No intervention	
**Assis, 2000**	Non-RCT	Brazil	6-59 months	871	Vitamin A supplementation	Children 6-12 months of age: vitamin A (100,000IU); children >1year of age: 200,000IU	1 dose	No intervention	
**Baqui, 1995**	RCT	Bangladesh	6-7 weeks	185	Vitamin A supplementation	Liquid: 25,000IU	3 doses at DPT immunization	Placebo	
**Barth-Jaeggi, 2015**	RCT	Kenya	6 months	379	MNP (with iron only)	1 sachet: vitamin A (100ug), iron (2.5mg), vitamin D (5ug), copper (0.34mg), tocopherol equivalent (5mg), iodine (30ug), vitamin K1 (30ug), selenium (17ug), thiamine (0.5mg), zinc (2.5mg), riboflavin (0.5mg), pyridoxine (0.5mg), folic acid anhydrous (90ug), niacinamide (6mg), vitamin B12 (0.9ug), vitamin C (60mg)	Daily for 12 months	MNP (without iron)	1 sachet: vitamin A (100ug), vitamin D (5ug), copper (0.34mg), tocopherol equivalent (5mg), iodine (30ug), vitamin K1 (30ug), selenium (17ug), thiamine (0.5mg), zinc (2.5mg), riboflavin (0.5mg), pyridoxine (0.5mg), folic acid anhydrous (90ug), niacinamide (6mg), vitamin B12 (0.9ug), vitamin C (60mg)
**Bisimwa, 2012**	RCT	Democratic Republic of Congo	4-5 months	1383	LNS (RUCF)	50g: vitamin A (412IU), vitamin D (307IU), vitamin C (75mg), thiamine (0.7mg), riboflavin (1.0mg), vitamin B12 (1ug), pyridoxine (0.24mg), niacin (10.3mg), pantothenic acid (3.8mg), folic acid (0.07mg), vitamin K (0.01mg), calcium (514mg), phosphorus (265mg), iron (9.5mg), zinc (8.3mg), copper (0.3mg), iodine (0.07mg), selenium (0.003mg), magnesium (23.4mg), phytic acid (2.1mg)	Daily for 6 months	MMN fortified corn soy blend flour	70g: Vitamin A (537IU), vitamin D (140IU), vitamin C (42mg), thiamine (0.2mg), riboflavin (0.6mg), vitamin B12 (0.9ug), pyridoxine (0.11mg), niacin (3.5mg), pantothenic acid (2.0mg), folic acid (0.14mg), vitamin E (5.3IU), calcium (182mg), iron (5.6mg), zinc (8.4mg), copper (0.5mg), iodine (0.03mg), phytic acid (0.650mg)

**Bloem, 1995**	Interrupted time-series study	Bangladesh	12-59 months	~124,606	Vitamin A supplementation	1 capsule: 200,000IU	Every 6 months	No intervention	
**Bougma, 2018**	cRCT	Ethiopia	5-11 months	2136 (60 clusters)	Iodine fortified salt		Usual use for 8-10 months	Iodine (iodised salt gradually made available through regular market channels once production was sufficient and legislation was enforced in Oct 2012)	Usual use for 4-6 months
**Caufield, 2013**<br><br>**Associated refs: Colombo, 2014**	RCT	Peru	6 months	251	MMN (iron, copper, zinc) supplementation	Liquid: iron (10mg), copper (0.5mg), zinc (10mg)	Daily for 12 months	Iron and copper	Liquid: iron (10mg) and copper (0.5mg)
**Chen, 2010**	RCT	China	4-12 months	1478 in main trial; 475 in this follow up study	MNP fortified whole soybean flour	10g sachet: iron (6mg), zinc (4.1mg), calcium (385mg), vitamin B2 (0.2mg), vitamin D (7.0ug), protein (3.8g), energy (167kJ)	Daily for 12-20 months	No intervention	
**Chen, 2012**	cRCT	China	36-72 months	361	1) vitamin A and zinc supplementation<br>2) MMN supplementation	1) 1 capsule: vitamin A (25000IU), 1 tablet: zinc (10mg)<br>2) 1 tablet: vitamin A (5000IU), vitamin B1 (1.5mg), vitamin B2 (1.7mg), vitamin B6 (2mg), vitamin B12 (4ug), vitamin C (50mg), vitamin D (400IU), folic acid (100ug), niacinamide (20mg), calcium (162mg)	1) vitamin A once every 14 days and zinc 5 times a week for 6 months<br>2) 5 times every 14 days for 6 months	Vitamin A	1 capsule: 25,000IU<br>Frequency/duration: once every 14 days for 6 months
**Cobra, 1997**	RCT	Indonesia	6-10 weeks	617	Iodine supplementation	1 capsule: 100mg		Placebo	
**de Almeida, 2005**	cRCT	Brazil	12-75 months	150 (6 clusters)	MNP (with iron and vitamin C) in drinking water	10 mg of iron and 100 mg of ascorbic acid per liter water (the pre-mix contained 1000 mg of FeSO4-7H2O and 2000 mg of ascorbic acid diluted in 20 ml of water)	Usual use for 6 months	MNP (with vitamin C only) in drinking water	Water with 10mg of iron and 100mg of ascorbic acid per liter (the pre-mix contained 1000 mg of FeSO4-7H2O and 2000 mg of ascorbic acid diluted in 20 ml of water)

**DeLong, 1997**	Controlled before-after study	China			Iodine fortified irrigation water	Long Ru: 1992 - 30kg given to an area of 4 villages, 1993 - 80kg given to same areas; Bakechi: 80kg given to an area of 8 villages Tusala: 80kg given to an area of 15 villages	Long Ru: 1992, 1993 Bakechi: 1993 Tusala: 1994	No intervention	
**Dutra-de-Oliveira, 2002**	RCT	Brazil	1-6 years	21 families (88 members)	MNP (with iron and vitamin C) in drinking water	10mL iron solution to 10L of water: elemental iron (10mg) and ascorbic acid (60mg) per L of drinking water	Daily for 4 months	Placebo	
**Fawzi, 1998**	RCT	Sudan	9-72 months	28,753	Vitamin A supplementation	1 capsule: 200,000IU	Bi-annually for 18 months	Placebo	
**Galasso, 2019**	cRCT	Madagascar	6-18 months	3738 households (125 clusters)	LNS	20g	Children in cohorts A and B (−6 months to <6 months at baseline) received the full 12 months of supplementation daily. Children in cohort C (6 months to <12 months) received daily LNS for 12 months or less.	No intervention	
**Gebremedin, 2014**	Propensity score matched retrospective cohort study	Ethiopia	6-59 months	4794	Vitamin A supplementation	Children aged 6–11 and 12–59 months are given 100,000 and 200,000 IU of VA (i.e. 30 and 60 mg of retinol), respectively in the form of a capsule	1 dose (within the preceding 6 months of the survey)	No intervention	
**Hadler, 2008**<br><br>**Associated refs: Medeiros, 2015**	cRCT	Brazil	6-24 months	192 (25 clusters)	IFA supplementation	Drops: iron (1.4mg/kg/day), folic acid (50ug)	5 days a week for ~3 months	Iron and folic acid placebo	Iron (1.4mg/kg/day)
**Hettiarachchi, 2010**	cRCT	Sri Lanka	3-5 years	267	LNS food supplement Thriposha	Per 100g: energy (401.8 cal), fat (7.8g), minerals (4.0g), protein (20g), carbohydrates (61.9g), vitamin A (1700IU), vitamin D (200IU), vitamin E (6IU), vitamin C (40mg), vitamin B6 (6mg), vitamin B12 (4mcg), thiamine	Daily for 9 months	Unfortified Thriposha (corn-soya blend)	50g

						(760mcg), riboflavin (560mcg), niacin (8mg), folic acid (200mcg), pantothenic acid (3mg), calcium (900mg), phosphorus (670mg), magnesium (96mg), iron (18mg), zinc (3mg), iodine (44mcg). Children were given 50g/day			
**Iannotti, 2014**	RCT	Haiti	6-11 months	589	LNS	20g: protein (2.56g), fat (7.08g), linoleic acid (1.29g), alpha linoleic acid (0.29g), vitamin A (400ug), thiamin (0.3mg), riboflavin (0.4mg), niacin (4mg), pantothenic acid (1.8mg), vitamin B6 (0.3mg), vitamin B12 (0.5mg), folic acid (80ug), vitamin C (30mg), calcium (100mg), copper (0.2mg), iodine (90ug), iron (9mg), magnesium (16mg), manganese (0.08mg), phosphorus (82.2mg), potassium (152mg), selenium (10ug), zinc (4mg)	Daily for 6 months	No intervention	
**Isanaka, 2009**	cRCT	Niger	6-60 months	3533 (12 clusters)	LNS	92g (500 kcal/day)	Daily for 3 months	No intervention	
**Kapur, 2003**	RCT	India	9-36 months	451	Iron	20mg	Weekly for 8 weeks	Placebo	
**Kartasasmita, 1995**	RCT	Indonesia	12-54 months	269	Vitamin A supplementation	200,000IU	2 doses (one at baseline and one at 6 months)	Placebo	
**Kujinga 2018**	RCT	Kenya	2-6 years	184	Zinc fortified filtered water	8mg/L water	Usual use for 25 weeks	Placebo (filtered water)	
**Lopez, 2006**	Non-RCT	Peru	6 months-4 years	866	MMN supplementation	1 foodlet: vitamin A as acetate (750ug), vitamin D (10ug), vitamin E (12IU), vitamin C (70mg), niacin (12mg), thiamine (1mg), riboflavin (1mg), pyridoxine (1mg), vitamin B12 (1.8mg), folic acid (300ug), iron as ferrous fumarate (20mg), zinc as zinc gluconate (20mg), copper as cupric gluconate (1.2mg), iodine as potassium iodide (100ug)	Weekly for 8 weeks	No intervention	

**Luabeya, 2007**<br><br>**Associated refs: Chhagan, 2009; Chhagan, 2010**	RCT	South Africa	4-6 months	373	1) Vitamin A and zinc supplementation<br>2) MMN supplementation	1) 1 tablet: zinc (10mg), vitamin A (1250IU)<br>2) 1 tablet: zinc (10mg), vitamin A (1250IU), vitamin B1 (0.5mg), vitamin B2 (0.5mg), vitamin B6 (0.5mg), vitamin B12 (0.9ug), vitamin C (35mg), vitamin D (5ug), vitamin E (6mg), vitamin K (10ug), copper (0.6mg), folate (150ug), iodine (50ug), iron (10mg), niacin (6mg)	Daily for 18 months	Vitamin A supplementation	1 tablet: 1250IU
**Lutter, 2008**	Non-RCT	Ecuador	9-14 months	634	MMN fortified cereal and legume flour	65g	Daily for 11 months	No intervention	
**Manaseki-Holland, 2012**<br><br>**Associated refs: Aluisio, 2013**	RCT	Afghanistan	1-11 months	3046	Vitamin D supplementation	0.5mL of syrup: vitamin D (100,000IU/2.5mg)	Every 3 months for 18 months	Placebo	
**Martinez-Estevez, 2016**	RCT	Colombia	6-12 months	355	MMN supplementation	3.5mm of syrup: zinc oxide (5mg), calcium carbonate (525mg), vitamin D3 (70IU)	Daily for 12 months	Calcium and vitamin D supplementation	3.5mm of syrup: Calcium carbonate (525mg), vitamin D3 (70IU)
**Matias, 2017**	RCT	Peru	6-11 months	422	LNS	20g LNS: energy (110kcal/460.24kJ), protein (2.6g), fat (7g), linoleic acid (1.29g), α-Linolenic acid (0.29g), folic acid (80ug), niacin (4mg), pantothenic acid (1.8mg), riboflavin (0.4mg), thiamin (0.3mg), vitamin A (400ug), vitamin B12 (0.5ug), vitamin B6 (0.3mg), vitamin C (30mg), calcium (100mg), copper (0.2mg), iodine (90ug), iron (9mg), magnesium (16mg), manganese (0.08mg), phosphorus (82mg), potassium (152mg), selenium (10ug), zinc (4mg)	Daily for 6 months	MNP	1 sachet: folic acid (160ug), vitamin A (300ug), vitamin C (30mg), iron (12.5mg), zinc (5mg)
**Mitra, 1997**	RCT	Bangladesh	2-48 months	349	MMN (with iron) supplementation	5mL of syrup: 125 mg of ferrous gluconate (15mg elemental iron), vitamin A (80,000 RE/L), vitamin D as cholecalciferol (2000ug/L), vitamin C (10,000mg/L)	Daily for 15 months	MMN (without iron) supplementation	5 mL of syrup: vitamin A (80,000 RE/L), vitamin D as cholecalciferol (2000ug/L), vitamin C (10,000mg/L)

**Muslihah, 2016**	Non-RCT	Indonesia	6-12 months	269	LNS	20g LNS: energy (118kcal), protein (2.6g), lipids (9.9g), linoleic acid (2.8g), a-linolenic acid (0.58g), vitamin A (400ug), vitamin B1 (0.5mg), vitamin B2 (0.5mg), vitamin B3 (6.0mg), vitamin B5 (2.0mg), vitamin B6 (0.5mg), vitamin B12 (0.9ug), folic acid (150ug), vitamin C (30mg), vitamin D (10ug), vitamin E (6mg), vitamin K (30ug), calcium (280mg), copper (0.34mg), iodine (90ug), iron (6mg), magnesium (40mg), manganese (1.2mg), phosphorus (190mg), potassium (200mg), selenium (20ug), zinc (8mg)	Daily for 6 months	No intervention	
**Nair, 2017**	Non-RCT	India	4-6 months	44	Iron and vitamin D supplementation	Iron (2mg/kg)	Daily for 6-8 months (enrollment until 1 year of age)	Vitamin D	
**Newton, 2016**	RCT	Ghana	7-9 months	93	MNP (with vitamin A)	1 sachet: elemental iron (12.5mg) as microencapsulated ferrous fumarate, ascorbic acid (30mg), retinyl palmitate (400ug RE), zinc (5mg)	Daily for 5 months	MNP (without vitamin A)	1 sachet: elemental iron (12.5mg) as microencapsulated ferrous fumarate, ascorbic acid (30mg), zinc (5mg)
**Nogueira, 2012**	cRCT	Brazil	10-23 months	216 (2 clusters)	Iron fortified rice	50g portion: elemental iron (56.4mg) as micronized ferric pyrophosphate (MFPP)	Weekly for 18 weeks	Unfortified rice	
**Noor, 2018**	RCT	Pakistan	6-11 months	120	Zinc supplementation	20mg	Daily for 14 days	Placebo	
**Oliveira, 2016**	Non-RCT	Brazil	6-14 months	326	MNP	10 mg Fe (encapsulated ferrous fumarate), 4·1 mg Zn (zinc gluconate), 150 µg folic acid, 400 µg vitamin A (retinol equivalents), 30 mg vitamin C (ascorbic acid), 5 µg cholecalciferol, 5 mg vitamin E (tocopherol equivalents), 0·5 mg thiamin, 0·5 mg riboflavin, 0·5 mg vitamin B6, 0·9 µg vitamin B12, 6 mg niacin, 0·56 mg Cu, 90 µg iodine and 17 µg Se	Daily for 60 days	No intervention	

**Olney, 2018**	cRCT	Guatemala	Supplementation given at 6 months	~39,000 mother/child pairs	LNS	2 x 10 g sachets: 118 kcal, protein 2.6g, fat 9.6g, linoleic acid 4.5g, alpha-linoleic acid 0.6g, Vit A 400ug, Vit C 30mg, vit D 5mg, Vit E 6mg, Vit K 30mg, Vit B1 (thiamin) 0.5mg, Vitamin B2 (riboflavin) 0.5 mg, niacin mg, pantothenic acid 2 mg, vitamin B6, 0.5mg, folic acid 150 ug, vit B12 0.9 ug, iron 9mg, zinc 8mg, copper 0.3mg, selenium 20ug, iodine 90ug, calcium 280mg, magnesium 40mg, manganese 1.2mg, phosphorus 190 mg, potassium 200 mg. Intervention Arm 3: MNP (micronutrient powder) (4g or 2 x2g sachets), each containing Vit A 400ug, Vit C 30mg, vit D 5mg, Vit E 6mg, Vit K 30mg, Vit B1 (thiamin) 0.5mg, Vitamin B2 (riboflavin) 0.5 mg, niacin mg, pantothenic acid 2 mg, vitamin B6, 0.5mg, folic acid 150 ug, vit B12 0.9 ug, iron 9mg, zinc 8mg, copper 0.3mg, selenium 20ug, iodine 90ug, calcium 280mg, magnesium 40mg, manganese 1.2mg, phosphorus 190 mg, potassium 200 mg.	Daily for 18 months	Placebo	
**Ouedraogo, 2010**	RCT	Burkina Faso	6-23 months	131	MMN fortified gruel	8 mg elemental iron (as ferrous fumarate), 5 mg zinc (as zinc gluconate), 300mg vitamin A (as retinyl acetate), 30 mg ascorbic acid and 60 mg iodine (as potassium iodate)	MMN once daily (6 days a week) and gruel twice daily for 6 months	Unfortified gruel	
**Owusu-Agyei, 2013**	RCT	Ghana	6-24 months	200	Vitamin A and zinc supplementation	Vitamin A: 100,000 IU for <12mo of age or 200 000 IU for older children, and 10mg zinc capsule	Daily for 6 months	Vitamin A and zinc placebo	100,000IU for <12 mos of age or 200,000IU for older children and placebo capsule

**Paganini, 2017**	RCT	Kenya	6.5-9.5 months	155	MNP (with iron)	1 sachet: vitamin A(400ug), vitamin D(5ug), tocopherol equivalents(5mg), thiamine (0.5mg), riboflavin(0.5mg), vitaminB6(0.5mg), folic acid(90ug), vitamin C,(30mg), copper(0.56mg), iodine(90ug), selenium(17ug), zinc(4.1mg), phytase(190 FTU), Iron(as ferrous fumarate)(2.5mg), iron(as NaFeEDTA)(2.5mg)+ maltodextrin(add to 11g)	Daily for 4 months	MNP (without iron)	1 sachet: vitamin A(400ug), vitamin D(5ug), tocopherol equivalents(5mg), thiamine (0.5mg), riboflavin(0.5mg), vitamin B6 (0.5mg), folic acid (90ug), vitamin C (30mg), copper(0.56mg), iodine(90ug), selenium (17ug), zinc(4.1mg), phytase(190 FTU), Iron (as ferrous fumarate) (2.5mg), Maltodextrin(add to 11g)
**Phuka, 2008**<br><br>**Associated refs: Phuka, 2009; Phuka, 2012**	RCT	Malawi	5.5 to 6.99 months	182	LNS	50g: energy (256 kcal), protein (7.0g), carbohydrates (13.8g), fat (16.9g), retinol (400ug RE), folate (160ug), niacin (6mg), pantothenic acid (2mg), riboflavin (0.5mg), thiamin (0.5mg), vitamin B6 (0.5mg), vitamin B12 (0.9ug), vitamin C (30mg), vitamin D (5ug), calcium (366mg), copper (0.4mg), iodine (135ug), iron (8mg), magnesium (60mg), selenium (17ug), zinc (8.4mg)	Daily for 1 year	MMN fortified maize flour	71g: energy (282kcal), protein (10.3g), fat (3.1g), retinol (138ug RE), folate (43ug), niacin (3mg), riboflavin (0.3mg), thiamin (0.1mg), vitamin B6 (0.3mg), vitamin B12 (0.9ug), vitamin C (48mg), calcium (71mg), iron (5mg), zinc (3.6mg)
**Rahman, 1999**	RCT	Bangladesh	6-71 months	250	MMN (with iron) supplementation	5mL of syrup: 125 mg of ferrous gluconate (15 mg elemental iron), vitamin A [80,000 retinol equivalents (RE)/L], vitamin D (2mg cholecalciferol/L) and vitamin C (10g/L)	Daily for 12 months	MMN (without iron) supplementation	5mL of syrup: vitamin A [80,000 retinol equivalents (RE)/L], vitamin D (2mg cholecalciferol/L) and vitamin C (10g/L)
**Rooze, 2016**	Non-RCT	China (Tibet Autonomous Region)	0-5 years	207	Vitamin D supplementation	625ug	Monthly for 3 years	No intervention	
**Sampaio, 2013**	RCT	Brazil	6-48 months	143	MNP (with zinc)	1 sachet: vitamin A (vitamin A acetate) (375mcg), vitamin B1 (thiamine mononitrate) (0.5mg), vitamin B2 (riboflavin) (0.5 mg), vitamin B6 (pyridoxine) (0.5 mg), vitamin B12 (cyanocobalamin) (0.9 mcg), vitamin C (ascorbic acid) (35 mg), vitamin D3 (cholecalciferol) (5.0 mcg),	Daily for 90 days	MNP (without zinc)	1 sachet: vitamin A (vitamin A acetate) (375mcg), vitamin B1 (thiamine mononitrate) (0.5mg), vitamin B2 (riboflavin) (0.5 mg), vitamin B6 (pyridoxine) (0.5 mg), vitamin B12 (cyanocobalamin) (0.9 mcg), vitamin C (ascorbic acid) (35 mg), vitamin D3

						vitamin E (vitamin E acetate) (6.0 mg), niacin (niacinamide) (6.0 mg), copper (copper sulfate) (0.6 mg), iodine (potassium iodide) (50 mcg), iron (ferrous fumarate) (12.5 mg), zinc (zinc gluconate) (5.0 mg)			(cholecalciferol) (5.0 mcg), vitamin E (vitamin E acetate) (6.0 mg), niacin (niacinamide) (6.0 mg), copper (copper sulfate) (0.6 mg), iodine (potassium iodide) (50 mcg), iron (ferrous fumarate) (12.5 mg)
**Sazawal, 1997**<br><br>**Associated refs: Sazawal, 1998**	RCT	India	6-35 months	609	MMN (zinc) supplementation	5 mL of liquid: vitamin A (240ug retinol equivalents), thiamine (0.6 mg). riboflavin (0.5 mg), vitamin B-6 (0.5 mg), cholecalciferol (2.5ug). vitamin E (3 mg a-tocopherol equivalents), niacin (10 mg niacin equivalents), zinc gluconate (10 mg elemental Zn). During diarrheal illness, this was increased to 10mL to provide for excess stool losses	Daily for 6 months	MMN (without zinc) supplementation	5 mL of liquid: vitamin A (240ug retinol equivalents), thiamine (0.6 mg). riboflavin (0.5 mg), vitamin B-6 (0.5 mg), cholecalciferol (2.5ug). vitamin E (3 mg a-tocopherol equivalents), niacin (10 mg niacin equivalents)
**Swami, 2007**	Natural experiment	India	1-5 years	528	Vitamin A supplementation		Two rounds of vitamin A supplementation	No intervention	
**Taneja, 2013**<br><br>**Associated refs: Kumar, 2017; Kvestad, 2015; Strand, 2015**	RCT	India	6-30 months	1000	1) LNS (with folic acid only)<br>2) LNS (with vitamin B12 only)	1) 5g for children 6-11 months of age and 10g for children ≥12 months; Per 10g: energy (54.1kcal), protein (0.7g), fat (3.3g), folic acid (150ug)<br>2) 5g for children 6-11 months of age and 10g for children ≥12 months; Per 10g: energy (54.1kcal), protein (0.7g), fat (3.3g), vitamin B12 (1.8ug)	Daily for 6 months	LNS (without vitamins and minerals)	5g for children 6-11 months of age and 10g for children ≥12 months; Per 10g: energy (54.1kcal), protein (0.7g), fat (3.3g)
**Teshome, 2017**	RCT	Kenya	12-36 months	338	1) MNP (with iron as NaFeDTA)<br>2) MNP (with iron as ferrous fumarate)	1 and 2) 1 sachet: vitamin A (300ug RE) vitamin D (5ug), vitamin E (5mg), vitamin C (30mg), thiamin (0.5mg), riboflavin (0.5mg), niacin (6mg), vitamin B6 (0.5mg), vitamin B12 (0.9ug), iron (either NaFeDTA 3mg or ferrous fumarate 12.5mg), zinc (5mg), copper (0.56mg),	Daily for 30 days	MNP (without iron)	1 sachet: vitamin A (300ug RE) vitamin D (5ug), vitamin E (5mg), vitamin C (30mg), thiamin (0.5mg), riboflavin (0.5mg), niacin (6mg), vitamin B6 (0.5mg), vitamin B12 (0.9ug), zinc (5mg), copper (0.56mg), selenium (17ug), iodine (90ug)

						selenium (17ug), iodine (90ug)			
**Venkatarao, 1996**	RCT	India	At birth (suppleme ntation given at 6 months of age)	909	Vitamin A	200,000IU	1 dose at 6 months of age	Placebo	
**Warthon-Medina, 2015**	RCT	Peru	6-17 months	902 in main trial; 200 in this follow-up study	MNP	1 sachet: iron (12.5mg), zinc gluconate (10mg), folic acid (160ug), vitamin C (30mg), vitamin A (300ug)	Daily for 6 months	MNP (with iron only)	1 sachet: 12.5mg
**Yadav, 2012**	Controlled before-after study	India	12-50 months		Fortified blended food		Duration: ~30 months	No intervention	
**Zlotkin, 2013**<br><br>**Associated refs: Aimone, 2013**	cRCT	Ghana	6-35 months	1958 (1552 clusters)	MNP (with iron)	1 sachet: elemental iron (as microencapsulated ferrous fumarate) (12.5mg), ascorbic acid (30mg), vitamin A (400µg), zinc (5 mg)	Daily for 5 months	MNP (without iron)	1 sachet: ascorbic acid (30mg), vitamin A (400µg), zinc (5 mg)

Appe[illegible]x 3 – Eligible Studies Included in Meta-Analyses

First author, year	Study design	Country	Age range enrolled	Total study population	Intervention arms used in meta-analysis	Intervention dose and formulation	Intervention duration and frequency	Control	Control dose and formulation
**Aboud, 2011**	cRCT	Bangladesh	8-20 months	302 (mother and child pairs)	MNP (Efficacy)	1 sachet: Iron (12.5mg), vitamin A (300ug), folic acid (150ug), vit C (50mg), zinc (5mg)	Daily for 6 months	No intervention	
**Adu-Afarwuah, 2007**<br><br>**Associated refs: Adu-Afarwuah, 2008**	RCT	Ghana	6 months	409	1) MNP (Efficacy)<br>2) MMN supplementation (Efficacy)<br>2) LNS (Efficacy)	1) 1 sachet: beta-carotene (300mcg RE), vit C (50mg), vit D (7.5mcg), folic acid (150mcg), iron (fumarate) (12.5mg), zinc (5mg)<br>2) 1 tablet: beta-carotene (400mcg RE), vit C (30mg), folic acid (80mcg), thiamine (0.3mg), riboflavin (0.4mg), vit B3 (4mg), pantothenic acid (1.8mg), vit B6 (0.3mg), vit B12 (0.5mg), iron (sulfate) (9mg), zinc (4mg), calcium (100mg), potassium (152mg), copper (0.2mg), selenium (10mcg), iodine (90mcg)<br>3) 20g: all nutrients in intervention 2 + phosphorus (82mg), magnesium (16mg), manganese (0.08mg), energy (108kcal), linoleic acid (1.29g), linolenic acid (0.29g), phytate (82mg)	Daily for 6 months	No intervention (recruited at baseline)	
**Adu-Afarwuah, 2016**<br><br>**Associated refs: Prado, 2016a**	RCT	Ghana	At birth (supplementation given at 6 months of age)	1228 infants; 1320 mothers	LNS (Efficacy)	20g: energy (118kcal), protein (2.6g), fat (9.6g), linoleic acid (4.46g), a-linolenic acid (0.58g), vitamin A (400mg RE), thiamin (0.3mg), riboflavin (0.4mg), niacin (4mg), vitamin B-6 (0.3mg), vitamin B-12 (0.5mg), vitamin C (30mg), vitamin D (5mg), vitamin E (6mg), vitamin K (30mg), folic acid (80mg),	Daily for 12 months	No intervention	

						pantothenic acid (1.8mg), iron (6mg), zinc (8mg), copper (0.34mg), calcium (280mg), phosphorus (190mg), potassium (200mg), magnesium (40mg), selenium (20mg), iodine (90mg), and manganese (1.2mg)			
**Agarwal, 1995**	cRCT	India	1-72 months	15, 247	Vitamin A (and vitamin E) supplementation (Efficacy)	15mL of syrup: children 1-6 months of age: vitamin A (50,000IU), vitamin E (10IU); children 7-72 months of age: vitamin A (100,000IU), vitamin E (20 IU)	Every 4 months for 12 months	Placebo: vitamin E supplementatio n	15 mL of syrup: children 1-6 months of age: vitamin E (10IU); children 7-72 months of age: vitamin E (20IU)
**Anand 2007**	RCT	India	3-6 years	415	MMN fortified candy (Targeted fortification – efficacy)	1 candy: vit A (1000IU), elemental iron (14mg), folic acid (50ug), ascorbic acid (20mg)	6 days a week for 12 weeks	Placebo: Unfortified candy	
**Anonymous, 1998**<br><br>**Associated refs: Bahl, 2002**	RCT	Ghana, India, Peru	Newborns (suppleme ntation given at >2 months of age)	9424 (mother and child pairs)	Vitamin A supplementation (Efficacy)	1 capsule: 25,000IU/7.5mg	4 doses; at DPT/poliomyel itis and Measles immunization: India and Ghana: 6, 10 and 14 weeks and 9 months of age; Peru: 2, 3, 4 months and 9 months of age	Placebo & vitamin A at 9 months of age (and mothers received placebo)	Placebo (first 3 doses); 1 capsule: 100,000IU/30mg of vitamin A (4th dose)
**Arcanjo, 2012**	cRCT	Brazil	4 years	120	Iron fortified milk-based cornstarch porridge (Targeted fortification – efficacy)	600 mg ferrous sulfate in liquid form (10 mg elemental iron per portion)	Daily for 14 weeks	Unfortified milk-based cornstarch porridge	
**Arcanjo, 2019**	cRCT	Brazil	12-36 months	169 (8 clusters total)	MNP (Efficacy)	Vitamin A (400mcg), vitamin D (5mcg), vitamin E (5mg), vitamin C (30mg), vitamin B1 (0.5mg), B2 (0.5mg), B6 (0.5mg), B12 (0.9mcg), niacin (6mg), folic acid (150mcg), iron (10mg), zinc (4.1mg), copper (0.56mg), selenium (17mcg), iodine (90mcg)	5 days a week (Monday to Friday) for 12 weeks	No intervention	

**Ashorn, 2015**<br><br>**Associated refs: Prado, 2016b**	RCT	Malawi	At birth;	781 infants; 869 mothers	LNS (Efficacy)	20g LNS: energy (118kcal), protein (2.6g), fat (9.6g), linoleic acid (4.46g), a-linolenic acid (0.58g), vitamin A (400mg RE), thiamin (0.3mg), riboflavin (0.4mg), niacin (4mg), vitamin B-6 (0.3mg), vitamin B-12 (0.5mg), vitamin C (30mg), vitamin D (5mg), vitamin E (6mg), vitamin K (30mg), folic acid (80mg), pantothenic acid (1.8mg), iron (6mg), zinc (8mg), copper (0.34mg), calcium (280mg), phosphorus (190mg), potassium (200mg), magnesium (40mg), selenium (20mg), iodine (90mg), and manganese (1.2mg)	Daily for 12 months	No intervention	
**Attanasio, 2014**<br><br>**Associated refs: Andrew, 2016**	cRCT	Colombia	12-24 months	1420 (96 clusters)	MNP (Efficacy and effectiveness)	1 sachet: iron (12.5mg), zinc (5mg), vitamin A (300µg RE), folic acid (160µg), vitamin C (30mg)	Daily for 18 months	No intervention	
**Awasthi, 2013**	cRCT	India	~1-6 years	~1,000,000 (72 clusters)	Vitamin A supplementation (Efficacy)	1 capsule: 200,000IU	Every 6 months for 5 years until children reach 6 years of age	No intervention (usual care)	
**Ayah, 2007**	RCT	Kenya	At birth (suppleme ntation given at 14 weeks of age)	564 (mother and child pairs)	Vitamin A supplementation (Efficacy)	1 capsule: 100,000IU	1Braz dose at 14 weeks of age	Placebo	
**Bagni, 2009**	cRCT	Brazil	12-60 months	387 (4 clusters)	Iron fortified rice (LSFF – efficacy)	Iron (4.2mg/100g of rice); 90g of rice per meal so approx. 3.78mg of iron/week was obtained	Weekly for 16 weeks	Unfortified rice	

**Baqui, 2003**<br><br>**Associated refs: Baqui, 2005; Black, 2004; Fischer Walker 2009**	RCT	Bangladesh	6 months	799	1) Iron (and riboflavin) supplementation (Efficacy)<br>2) Zinc (and riboflavin) supplementation (Efficacy)<br>3) MMN (and riboflavin) supplementation (Efficacy)	1) Iron (20mg), riboflavin (1mg)<br>2) Zinc (20mg), riboflavin (1mg)<br>3) Iron (20mg), zinc (20mg), riboflavin (1mg), twice the WHO recommended daily intakes of iodine, copper, manganese, selenium, vitamin C, vitamin D, vitamin E, thiamin, niacin, pyridoxine, folic acid, cyanocobalamin and pantothenic acid	Weekly for 6 months	Riboflavin	1mg
**Barbosa, 2012**	cRCT	Brazil	2-6 years	324 (4 clusters)	Iron fortified wheat rolls (LSFF – efficacy)	14 mg of iron/roll; max 3 rolls (20g each) offered per child	5 days a week per 24 weeks	Unfortified wheat rolls	
**Barffour, 2019**	RCT	Lao People's Democratic Republic (Lao PDR)	6-23 months	3407	1) Zinc supplementation (Efficacy)<br>2) MNP (Efficacy)	1) 1 tablet: zinc (7mg)<br>2) 1 sachet: zinc (10mg), iron (6mg), 13 other micronutrients	Daily for ~9 months	Placebo	Powder form
**Batra, 2016**	cRCT	Guinea-Bissau	3-5 years	533 (9 clusters)	LNS (RUSF) – 15% protein from dairy sources (Efficacy and effectiveness)	92g: energy (478 kcal), protein (11.5g), fat (16.7g), vitamin A (506ug), vitamin D (6.1ug), vitamin E (15.2mg), vitamin K (25.3ug), vitamin C (75.9mg), thiamin (0.5mg), riboflavin (1.0mg), niacin (12.7mg), pantothenic acid (2.0mg), pyridoxine (1.0mg), biotin (10.1ug), folic acid (202ug), cyanocobalamin (2.0ug), sodium (<278mg), potassium (734mg), calcium (476 mg), phosphorus (430mg), magnesium (142mg), copper (0.5mg), iron (4.6mg), iodine (111ug), zinc (10.1mg), selenium (30.4ug)	5 days a week for 3 months	No intervention	

**Baum, 2017**	cRCT	Haiti	6-59 months	521 (34 clusters)	MNP (Effectiveness)	1 sachet: vitamin A, B, B2, B6, B12, C, D, and E, folic acid, niacin, copper, iodine, iron (2.5mg ferrous lactate), zinc and selenium	Daily for 3 months	No intervention	
**Becquey, 2016**	cRCT	Burkina Faso	6-30 months	7641 (36 clusters)	Zinc supplementation (Efficacy)	1 tablet: 7mg	Daily for 16, 32 or 48 weeks	No intervention/morbidity surveillance control	
**Begin, 2008**	RCT	Guatemala	6-7 months	259	MNP fortified maize based pudding (and whey protein concentrate) (Efficacy)	Vitamin A (400ug RE), vitamin B1 (0.4mg), vitamin B2 (0.5mg), niacin (0.6mg), vitamin B6 (0.6mg), vitamin B12 (0.5ug), vitamin C (35mg), vitamin D3 (400IU), folic acid (35ug), iron (10mg), zinc (5mg), iodine (50ug), selenium (15ug)	Daily for 8 months	Whey protein concentrate maize based pudding	
**Bentley, 1997**<br><br>**Associated refs: Rivera, 1998; Ruel, 1997**	RCT	Guatemala	6-9 months	108	Zinc supplementation (Efficacy)	4mL liquid: 10mg	Daily for 7 months	Placebo	
**Berger, 2000**	RCT	Togo	6-36 months	197	Iron supplementation (Efficacy)	1 tablet: 2–3 mg of elemental iron per kg of body weight	Daily for 3 months	Placebo	
**Berger, 2006**	RCT	Vietnam	4-7 months	915	1) Iron supplementation (Efficacy)<br>2) Zinc supplementation (Efficacy)	1) Syrup: 10mg<br>2) Syrup: 10mg	Daily for 6 months	Placebo	
**Bhandari, 2002a**<br><br>**Associated refs: Bhandari, 2002b; Taneja, 2005; Taneja, 2010**	RCT	India	6-30 months	2482	Zinc supplementation (Efficacy)	6 mL for infants: 10mg; 12 mL to older children: 20mg	Daily for 4 months	Placebo	

**Brooks, 2005**	RCT	Bangladesh	2-12 months	1621	Zinc supplementation (Efficacy)	Syrup: zinc (70mg)	Weekly for 12 months (endline measurements taken at 10 months to minimize attrition)	Placebo	
**Cardoso, 2016**<br><br>**Associated refs: Silva, 2017**	Non-RCT	Brazil	Intervention group: 6-8 months; control group: 10-14 months	1085	MNP (Efficacy)	1 sachet: iron (ferrous fumarate) (10mg), zinc (gluconate) (4.1mg), folic acid (150ug), vitamin A (RE) (400ug), vitamin C (30mg), vitamin D3 (5ug), vitamin E (5mg), vitamin B1 (0.5mg), vitamin B2 (0.5mg), vitamin B6 (0.5mg), vitamin B12 (0.9ug), niacin (6mg), copper (0.56mg), iodine (90ug), selenium (17ug)	Flexible use; 2 months supply (60 sachets) over a follow up period of 2-3 months (when intervention group reached the age of the control group at enrollment)	No intervention	
**Chang, 2010**	RCT	Bangladesh	6-18 months	1000	1) Daily alternating zinc and placebo supplementation (Efficacy)<br>2) Daily alternating IFA and placebo supplementation (Efficacy)<br>3) Daily alternating MMN and placebo supplementation (Efficacy)	1) 1 tablet: 10mg zinc<br>2) 1 tablet: 12.5 mg iron and 50IU folic acid<br>2) 1 tablet: 12.5mg iron, 50IU folic acid, 10mg zinc	Daily for 6 months	Placebo	
**Chen, 2005**	cRCT	China	≥3 years (3-6 years subgroup)	14,016 residents ≥3 years of age (9 clusters)	Iron fortified soy sauce (LSFF – efficacy)	The actual measured iron concentration of the fortified soy sauce was 23 mg/dL (range, 21–25). The average daily soy sauce consumption of the group consuming the fortified product was 16.4 ml per person, which provided 4.9 mg of iron	Daily for 18 months	Unfortified soy sauce	
**Chen, 2008**	RCT	China	2-6 years	282	MNP fortified seasoning	1 sachet: vitamin A (500ug), iron (12mg), thiamine (0.7mg), riboflavin (0.7mg),	5 days a week for 6 months	Vitamin A fortified	1 sachet: 500ug

**Associated refs: Chen, 2011**					powder (Efficacy)	folic acid (0.2mg), niacinamide (7mg), zinc (12mg), calcium (800mg)		seasoning powder	
**Chen, 2013**<br><br>**Associated refs: Chen, 2014**	RCT	China	3-6 years	445	1) Vitamin A supplementation (Efficacy)<br>2) Iron supplementation (Efficacy)	1) 1 capsule: 200,000IU<br>2) 1 tablet: 1-2 mg iron/kg	1) 1 dose<br>2) Daily for 6 months	No intervention	
**Christian, 2015**	cRCT	Bangladesh	6 months	5939 (596 clusters)	1) LNS (Plumpy'doz) (Efficacy)<br>2) MMN fortified wheat soy blend (Targeted fortification – efficacy)	1) ~23g for children 6-12 months; ~46g for children 12-18 months<br>2) 1 sachet (32g) for children 6-12 months; 2 sachets for children 12-18 months	Daily for 12 months	No intervention	
**Dewey, 2017**<br><br>**Associated refs: Matias, 2017; Matias, 2018; Ullah, 2019**	cRCT	Bangladesh	At birth (supplementation given at 6 months of age)	3664 children (live births); 4011 women (64 clusters)	1) LNS (Efficacy and effectiveness)<br>2) MNP (Efficacy)	1) 20g: energy (118kcal), protein (2.6g), fat (9.6g), linoleic acid (4.46g), α-linolenic acid (0.58g), vitamin A (400ug RE), thiamin (0.5mg), riboflavin (0.5mg), niacin (6mg), folic acid (150ug), pantothenic acid (2mg), vitamin B6 (0.5mg), vitamin B12 (0.9ug), vitamin C (30mg), vitamin D (5ug), vitamin E (6mg), vitamin K (30ug), calcium (280mg), copper (0.34mg), iodine (90ug), iron (9mg), magnesium (40mg), manganese (1.2mg), phosphorus (190mg), potassium (200mg), selenium (20ug), zinc (8mg)<br>2) 1 sachet: vitamin A (400ug RE), thiamin (0.5mg), riboflavin (0.5mg), niacin (6mg), folic acid (150ug), vitamin B6 (0.5mg), vitamin B12 (0.9ug), vitamin C (30mg), vitamin D (5ug), vitamin E (5mg), copper (0.56mg), iodine (90ug), iron (10mg), selenium (17ug), zinc (4.1mg)	Daily for 18 months	Placebo	

**Dibley, 1996**<br><br>**Associated refs: Hadi 2000; Hadi 2004**	RCT	Indonesia	6-47 months	1405; on average 782 participated in each cycle	Vitamin A (and vitamin E) supplementation (Efficacy)	Children <12 mos. of age: 1 capsule: 107umol (103,000IU) RE vitamin A and 40umol (17IU) vitamin E; Older participants: 1 capsule: 214umol (206,000IU) RE vitamin A and 84umol (37IU) vitamin E	Every 4 months for 6 cycles	Placebo: vitamin E	Children <12 mos. of age: 40umol (17IU); Older participants: 84umol (37IU)
**Dijkhuizen, 20001**	RCT	Indonesia	4 months	478	1) Iron supplementation (Efficacy)<br>2) Zinc supplementation (Efficacy)	1) Syrup: 10mg<br>2) Syrup: 10mg	5 days a week for 6 months	Placebo	
**Domellof, 2001**<br><br>**Associated refs: Domellof 2002; Dewey 2002**	RCT	Honduras	4 months	142	Iron supplementation (Efficacy)	Liquid: 1 mg/kg iron - adjusted monthly according to infant weight	Daily for 5 months	Placebo	
**Dossa, 2001**	RCT	Benin	3-5 years	177	Iron supplementation (Efficacy)	elemental iron (60mg)	Daily for 3 months	Placebo	
**Drammeh, 2002**	RCT	The Gambia	2-7 years	176	Vitamin A (and vitamin E) supplementation (Efficacy)	1 Capsule: vitamin A (60,000ug) and vitamin E (40mg)	1 dose at baseline	Placebo: vitamin E	1 capsule: 40mg
**Ekvall, 2000**	RCT	Tanzania	5 months-3 years	207	MMN supplementation (Efficacy)	Liquid: vitamin A (1500IU), vitamin D (400IU), vitamin E (5IU), vitamin C (35mg), vitamin B1 (0.5mg), vitamin B2 (0.6mg), niacin (8mg), vitamin B6 (0.4mg), iron (10mg)	3 times a week for 5 months (56 doses total)	Placebo	
**Ermis, 2002**	RCT	Turkey	5 months	113	Iron supplementation (Efficacy)	Drops: 1 mg/kg iron - adjusted monthly according to infant weight	Daily for 4 months	Placebo	

**Esamai, 2014**	RCT	Kenya	5 months	45	MNP (with iron) (Efficacy)	1 sachet: zinc (5mg), vitamin A (300ug), vitamin C (30mg), folic acid (160ug), iron (12.5mg)	Daily for 3 months	Placebo	
**Faber, 2005**	RCT	South Africa	6-12 months	361	MMN fortified maize porridge (Targeted fortification – efficacy)	40g of dry product: β-carotene (3mg), iron (11mg), zinc (3mg), copper (11ug), selenium (10ug), riboflavin (0.4mg), vitamin B6 (0.15mg), vitamin B12 (0.25ug), vitamin E (2.5mg), 2.5 mg vitamin E	Daily for 6 months	Unfortified maize porridge	
**Fahmida, 2007**	RCT	Indonesia	3-6 months	800	1) Zinc supplementation (Efficacy)<br>2) MMN supplementation (Efficacy)	1) 2mL of syrup: zinc (10mg)<br>2) 2mL of syrup: iron (10mg), zinc (10mg), vitamin A (1000IU)	Daily for 6 months	Placebo	
**Fisker, 2013**<br><br>**Associated refs: Fisker, 2014**	RCT	Guinea-Bissau	6-17 months	7587	Vitamin A (and vitamin E) supplementation (Efficacy)	Per 0.5mL liquid: vitamin A (100,000IU), vitamin E (20IU); per 1mL: vitamin A (200,000IU), vitamin E (40IU);<br>children <1 years of age received 0.5mL;<br>children ≥1 years of age received 1mL	1 dose at randomization	Placebo	
**Ghosh, 2019**	cRCT	Africa	6 months	970 (38 clusters)	MNP (Efficacy)	1 sachet: vitamin A (200.1ug RE), folic acid (45ug), niacin (3.1mg), riboflavin (0.3mg), thiamin (0.3mg), vit B6 (0.3mg), vit B12 (0.5ug), vit C (30.4mg), Ca (220.3mg), Fe (7mg), P (154.4mg), Zn (2.4mg), choline (62.5mg), vit D (2.5ug), vit E (2.7mg), iodide (0mg), vit K (11ug)	Daily for 12 months	No intervention	

**Giovanni, 2006**<br><br>**Associated refs: Agnostoni, 2007**	RCT	Cambodia	6 months	204	MNP (Efficacy)	1 sachet: fe (fumarate) (12.5mg), Zn (5mg), vitamin C (50mg), vitamin A (300mcg), vitamin D3 (7.5mcg), folic acid (150mcg)	Daily for 12 months	Placebo	
**Glinz, 2015**<br><br>**Associated refs: Glinz, 2017**	cRCT	Cote d'Ivoire	12-36 months	629 (40 clusters)	Iron fortified maize and soy-based porridge 1) with ferrous fumarate<br>2) ferrous pyrophosphate; We combined groups 1 and 2 (Targeted fortification – efficacy)	25g dry weight: iron as NaFeDTA (2mg), iron as ferrous fumarate or ferric pyrophosphate (3.8mg), and native iron (0.6mg)	6 days a week for 9 months	No intervention	
**Gokay, 2012**	RCT	India	6 months	120	Iron supplementation (Efficacy)	1mg/kg	Daily for 6 months	No intervention	
**Gupta, 2003**	RCT		6-41 months	280	Zinc supplementation (Efficacy)	5mL syrup: elemental zinc (10mg)	5 days a week for 16 weeks	Placebo	
**Gupta, 2007**	RCT	India	6-48 months	1878	Zinc supplementation (Efficacy)	5mL syrup: zinc (25mg)	Weekly for 6 months	Placebo	
**Hamadani, 2001**<br><br>**Associated refs: Osendarp, 2002**	RCT	Bangladesh	1 month	301	Zinc supplementation (Efficacy)	Syrup: elemental zinc (5mg)	Daily for 5 months	Placebo	
**Hess, 2015a**<br><br>**Associated refs:**	cRCT	Burkina Faso	8.8-9.9 months	3220 (2390 concessions/ clusters)	1) LNS 5mg zinc + placebo (LNS-Zn5)	1-2) 20g: energy (118kcal), protein (2.6g), fat (9.6g), vit A (400mg), B1 (0.3mg), B2 (0.4mg), B3 (4mg), B5	Daily for 9 months	No intervention	

**Abbeddou, 2015; Hess 2015b; Prado 2016c; Some, 2015**					2) LNS 10mg zinc + placebo (LNS-Zn10) We combined groups 1 and 2 (Efficacy)	(1.8mg), B6 (0.3mg), B12 (0.5mg), folic acid (80mg), vit C (30mg), vit D (5mg), vit E (6mg), vit K (30mg), Ca (280mg), copper (0.34mg), I (90mg), Fe (6mg), Mg (40mg), Mn (1.2mg), P (190mg), K (200mg), Se (20mcg), Zinc (5, or 10mg)			
**Huybregts, 2012**	cRCT	Chad	6-36 months	1038 (14 clusters)	LNS (Plumpy'Doz) (Efficacy and effectiveness)	46g (247 kcal/day)	Daily for 4 months	No intervention	
**Jack, 2012**	cRCT	Cambodia	6-7 months	3112 (20 clusters); 1350 recruited to subsample	MNP (Efficacy)	1 sachet: Iron (12.5mg), zinc gluconate (10mg), vit A (300mcg), iodine (90mcg), vit B1 (0.5mg), vit B2 (0.5mg), B6 (0.5mg), B12 (0.9mcg), niacin (6mg), folic acid (160mcg), vit C (30mg), copper (0.3mg), vit D (5mcg), vit E (6IU)	Daily for 6 months	No intervention	
**Kemmer, 2012**	RCT	Honduras	6-60 months	199	MNP (Efficacy)	1 sachet: iron (12.5mg), zinc (5mg), folic acid (150ug), vitamin A (1600IU), vitamin C (50mg), and vitamin D (300IU)	Daily for 4 months	No intervention	
**Kikafunda, 1998**	RCT	Uganda	30-89 months	155	Zinc supplementation (Efficacy)	1 tablet: zinc (10mg)	5 days a week (Monday to Friday) for 6 months (2 supplementation phases lasting 3 months each with a 2 month break in between due to school break)	Placebo	

**Kounnavong, 2011**	RCT	Lao People's Democratic Republic (Lao PDR)	6-52 months	336	MNP (Efficacy)	1 sachet: vitamin A (RE 400ug), vitamin D3 (5ug), vitamin E (TE 5mg), vitamin B1, B2, B6 each (0.5mg), folic acid (150ug), niacin (6mg), vitamin B12 (0.9ug), vitamin C (30mg), iron (10mg), zinc (4.1mg), selenium (17ug), copper (0.56mg), and iodine (90ug)	Daily for 24 weeks	Standard of care	
**Larson, 2018**	cRCT	India	6-18 months	10,000 (90clusters)	MNP (Efficacy and effectiveness)	1 sachet: iron (Ferrous Fumarate) 12.5 mg Zinc (Zinc Gluconate) 5 mg Folic Acid 0.160 mg Vitamin-A (Vit-A Acetate) 0.30 mg Vitamin-C (Ascorbic Acid) 30 mg Vitamin-B12 0.9 mcg Iodine 90 mcg	Daily; baseline and endline surveys conducted on August to September 2014 and from February to March 2016, respectively	No intervention	
**Lartey, 1999**	RCT	Ghana	<1 month (supplementation given at 6 months of age)	208	MMN fortified Weanimix (cereal-legume blend of maize, soybeans and groundnuts) (Targeted fortification – efficacy)	High: consuming > 60g or low: consuming ≤ 60g: energy (4350kcal), protein (150g), fat (114g), calcium (high 17360mg, low 8950mg), iron (high 366mg, low183mg), zinc (high 171mg, low 86mg), copper (high 25mg, low 13mg), magnesium (1400mg), potassium (high 18960mg, low 12310mg), sodium (30mg), phosphorus (high 17900mg, low 9400mg), ascorbic acid (high 781mg, low 391mg), niacin (high 29mg, low 149mg), pyridoxine (high 31.3mg, low 17.4mg), riboflavin (high 19.5mg, low 9.8mg), thiamine (high 22.1mg, low 13.5mg), vitamin B-12 (high 7ug, low 35ug), folic acid (high 5470ug, low 3070ug), vitamin A (high 18360 RE, low 9360 RE))	Daily for 6 months	Unfortified Weanimix (cereal-legume blend of maize, soybeans and groundnuts)	

**Le, 2005**	RCT	Vietnam	6-12 months	346	1) MMN supplementation (Efficacy)<br>2) Iron supplementation (Efficacy)	1) 1 tablet: vitamin A (375ug RE/1250IU), vitamin D (5ug), vitamin E (6mg), vitamin K (10ug), vitamin C (35mg), vitamin B1 (0.5mg), vitamin B2 (0.5mg), vitamin B6 (0.5mg), vitamin B12 (0.9ug), niacin (6mg), folate (150ug), iron (10mg), zinc (5mg), copper (0.6mg), iodine (59ug)<br>2) 1 tablet: iron (10mg)	Daily for 6 months (both intervention arms)	Placebo	
**Le Port 2017**	cRCT	Senegal	24-59 months	462 children from 310 households (247 concessions)	MMN fortified yoghurt (traditional Senegalese yoghurt mixed with millet) (LSFF – efficacy)	80g sachet: iron-EDTA (2.1mg), zinc (2.25mg), iodine (24ug), vitamin A (120ug)	Daily for 11 months	No intervention	
**Lind, 2003**<br><br>**Associated refs: Lind, 2004; Lind, 2008**	RCT	Indonesia	≤6 months	680	1) Iron supplementation (Efficacy)<br>2) Zinc supplementation (Efficacy)	1) 1.6mL of syrup: iron (10mg) as ferrous sulphate<br>2) 1.6 mL of syrup: zinc (10mg) as zinc sulphate	Daily for 6 months	Placebo	
**Lo, 2011**	RCT	Senegal	9-17 months	137	Iron fortified maize and millet-based porridge and MMN supplement (Targeted fortification – efficacy)	25g: per kg dry weight of complementary food: iron as ferrous fumarate (60mg), zinc as zinc oxide (240mg); Per 5mL of MMN supplement: thiamine (0.2mg), riboflavin (0.2mg), niacin (2mg), vitamin B6 (0.2mg), vitamin B12 (0.3ug), pantothenic acid (0.7mg), biotin (2.6mg), vitamin C (5mg)	Daily for 15 days	Iron fortified maize and millet-based porridge, and MMN (without zinc) supplement	25kg: per kg dry weight of complementary food: iron as ferrous fumarate (60mg); Per 5mL of MMN supplement: thiamine (0.2mg), riboflavin (0.2mg), niacin (2mg), vitamin B6 (0.2mg), vitamin B12 (0.3ug), pantothenic acid (0.7mg), biotin (2.6mg), vitamin C (5mg)
**Long, 2006**<br><br>**Associated refs: Rosado, 2009**	RCT	Mexico	6-15 months	786	1) Vitamin A supplementation (Efficacy)<br>2) Zinc supplementation (Efficacy)	1) 5mL of liquid: retinol (20,000IU) for children ≤1 years of age or 45,000IU for children >1 years of age<br>2) 5mL of liquid: zinc as zinc methionine (20mg)	1) Every 2 months for a year<br>2) Daily for a year	Placebo	
**Long, 2007**	RCT	Mexico	6-15 months	195	Vitamin A supplementation (Efficacy)	5 mL of liquid: retinol (20,000IU) for children ≤1 years of age or 45,000IU for children >1 years of age	Every 2 months for 15 months	Placebo	

**Lopez, 2005**	RCT	Peru	6-12 months	313	1) MMN supplementation (Efficacy)<br>2) Iron supplementation (Efficacy)	1) 1 foodlet: 1 RDA: vitamin A as retinyl acetate (375ug RE), vitamin D (5ug), vitamin E (6mg), vitamin K (10ug), vitamin C (35mg), vitamin B1 (0.5mg), vitamin B2 (0.5mg), vitamin B6 (0.5mg), vitamin B-12 (0.9ug) , niacin (6mg), folate (150ug), iron as ferrous fumarate (10mg), zinc as zinc gluconate (5mg), copper as cupric gluconate (0.6mg), and iodine as potassium iodide (50ug)<br>3) 1 foodlet: iron as ferrous sulfate (10mg)	Daily for 6 months (both intervention arms)	Placebo	
**Lopez, 2014**	Controlled before-after study	Nicaragua	Anemia sample: 0-60 months; cognitive sample: 30-60 months	57 in the anemia sample and 1047 in the cognitive assessment sample	MNP (Effectiveness)	1 sachet: six micronutrients (Sprinkles) were administered as per World Health Organization (WHO)/Sprinkles Global Health Initiative (SPGH) protocol	Daily for 2 months	No intervention	
**Lozoff, 1996**	RCT	Costa Rica	12-23 months	87 (32 ID anemic infants, 54 non-anemic infants)	Iron supplementation (Efficacy)	Drops: 3mg/kg	Twice daily for 6 months	Placebo	
**Lozoff, 2016**<br><br>**Associated refs: Angulo-Barroso, 2016**	RCT	China	At birth (supplementation at 6 weeks of age)	1482 infants; 2371 mothers	Iron supplementation (Efficacy)	Liquid: ~1mg/kg of elemental iron. Each mL contained 2.7 mg elemental iron. To simplify dosing, the volume was 2 mL/d for infants aged <6 mos. and 3 mL/d for infants aged ≥6 mos.	Daily for ~7.5 months	Placebo	
**Luby, 2018**<br><br>**Associated refs: Tofail, 2018**	cRCT	Bangladesh	Supplementation given at 6 months	720 clusters	LNS (Efficacy and effectiveness)	2 x 10 g sachets: 118 Kcal, 9.6 g of fat, 2.6 g of protein, 12 vitamins and 10 minerals	Daily for 18 months	No intervention	
**Lucia, 2017**	cRCT	Brazil	2-6 years	112	MMN fortified rice (LSFF – efficacy)	50g rice portion containing 0.5 g of UR: iron (4.2mg), zinc (2.1mg), thiamine (vitamin B1) (0.36mg), folic acid (72mg).	5 days a week for 4 months	Unfortified rice	

**Lundeen, 2010**	cRCT	Kyrgyz Republic (Kyrgyzstan)	6-36 months	2193 (24 clusters)	MNP (Efficacy)	1 sachet: elemental iron as microencapsulated ferrous fumarate (12.5mg), vitamin A as retinol acetate (300ug), zinc as zinc gluconate (5mg), vitamin C as ascorbic acid (30mg), and folic acid (160ug)	Daily for 2 months	No intervention	
**Luo, 2017**	cRCT	China	6-11 months	1802 (351 clusters)	MNP (Efficacy and effectiveness)	1 sachet: Iron (ferrous lactate) (6.0mg), zinc (zinc sulfate) (4.80mg), vitamin A (200µgRE), vitamin C (50.0mg), vitamin D (5.0µg), vitamin E (1.55mg), vitamin B1 (0.30mg), vitamin B2 (0.50mg), vitamin B6 (0.30mg), vitamin B12 (0.5µg), folic acid (66µg), niacin (3.0mg)	Daily for 18 months	No intervention	
**Ma, 2016**<br><br>**Associated refs: Sheng, 2019**	cRCT	China	6 months	1465	MMN fortified rice cereal (Targeted fortification – efficacy)	20g: iron (1.10 mg), zinc and vitamin B 12	Daily for 12 months	Unfortified local rice cereal	20g
**Macharia-Mutie, 2012**	RCT	Kenya	12-59 months	279	MNP (Efficacy)	1 sachet: retinyl palmitate (100µg RE), cholecalciferol (5µg), 1-α tocopheryl acetate (5mg TE), phylloquinone (30µg), thiamin (0.5mg), riboflavin (0.5mg), pyridoxine (0.5mg), folic acid (90µg), niacin (6mg), vitamin B12 (0.9µg), vitamin C (60mg), iron as NaFeDTA (2.5mg), zinc (2.5mg), selenium (17µg), copper (0.34mg), iodine (30µg)	Daily for 16 weeks	Unfortified maize porridge	
**Maleta, 2015**<br><br>**Associated refs: Bendadenda 2016; Prado, 2016d**	RCT	Malawi	5.5-6.5 months	1932	LNS (Efficacy)	20g: energy (490kJ), protein (2.5g), fat (9.5g), linoleic acid (4.44g), alpha-linolenic acid (0.58g), phytate (56mg), vitamin A (400ug RE), vitamin C (30mg), thiamin (0.3mg), riboflavin (0.4mg), niacin (4mg), folic acid (80ug), pantothenic acid (1.8mg), vitamin B6 (0.3mg), vitamin B12 (0.5ug), vitamin D (5ug), vitamin E (6mg), vitamin K (30ug), Fe (6mg),	Daily for 12 months	No intervention	

						Zn (8mg), Cu (0.34mg), Ca (240mg), P (208mg), K (265mg), Mg (50mg), Se (20ug), iodine (90ug), Mn (1.2mg)			
**Malik, 2013**<br><br>**Associated refs: Malik, 2014**	RCT	India	6-11 months	272	Zinc supplementation (Efficacy)	5ml of syrup: zinc as zinc sulfate (20mg)	Daily for 2 weeks	Placebo	
**Mangani, 2014**<br><br>**Associated refs: Mangani, 2014b; Mangani, 2015**	RCT	Malawi	5.5-6.5 months	840	LNS (with milk protein base) (Efficacy)	54g: energy (285kcal), protein (8.2g), fat (17.9g), retinol (400ug RE), folate (160ug), niacin (6mg), pantothenic acid (2mg), riboflavin (0.5mg), thiamin (0.5mg), vitamin B6 (0.5mg), vitamin B12 (0.9ug), vitamin C (30mg), vitamin D (5ug), calcium (366mg), copper (0.4mg), iodine (90ug), iron (6mg), magnesium (78.5mg), selenium (20ug), zinc (6.0mg), phosphorus (186mg), potassium (319mg), manganese (0.6mg)	Daily for 12 months	No intervention	
**Massaga, 2003**	RCT	Tanzania	12-16 weeks	291	Iron supplementation (Efficacy)	2.5mL of liquid: iron as ferric ammonium citrate mixture (3mg elemental iron/mL)	Daily for 6 months	Placebo	
**Mazariegos, 2010**	RCT	Guatemala	6-11 months	412	Zinc supplementation (Efficacy)	Half a tablet: zinc (5mg)	Daily for 6 months	Placebo	
**McDonald, 2015**<br><br>**Associated refs: Carter, 2018; Locks, 2016**	RCT	Tanzania	5-7 weeks	2400	1) MMN supplementation (Efficacy)<br>2) Zinc supplementation (Efficacy)	1 capsule: vitamin C (60mg), vitamin E (8mg), vitamin B1 (0.5mg), vitamin B2 (0.6mg), niacin (4mg), vitamin B6 (0.6mg), folate (130ug), vitamin B12 (1ug), zinc (5mg)<br>2)1 capsule: zinc (5mg)<br>1 capsule through 6 months of age, and 2 capsules from 7 months of age to end of follow-up	Daily for 18 months	Placebo	

**Menendez, 1997**<br><br>**Associated refs: Menendez, 2004**	RCT	Tanzania	8 weeks	832	Iron supplementation (Efficacy)	2mg/kg; we assumed average body weight of 6kg, therefore children received 2.4 mL of ferrous glycine sulphate containing 5 mg of ferrous sulfate per 5 mL	Daily for 4 months	Placebo	
**Menon, 2007**	cRCT	Haiti	9-24 months	425 (10 clusters)	MNP (to be added in wheat soy blend) (Efficacy and effectiveness)	1 sachet MMN: iron (12.5mg), zinc (5mg) , vitamin A (400ug), folic acid (160ug) and vitamin C (30mg)	Daily for 2 months	Fortified wheat soy blend	
**Monteiro, 2001**	Non-RCT	Brazil	6-59 months	1134	Iron supplementation (Efficacy)	Children 6-9 months old (30mg of elemental iron), children 10 to 17 months old (40mg), children 18 to 29 months old (50mg), children 30 to 35 months old (60mg), children 36 to 59 months old (80mg).	Weekly for ~7 months	No intervention	
**Muller, 2001**<br><br>**Associated refs: Garenne, 2007; Muller, 2003**	RCT	Burkina Faso	6-31 months	709	Zinc supplementation (Efficacy)	1 tablet: zinc (12.5mg)	6 days a week for 6 months	Placebo	
**Nagpal, 2004**	RCT	India	4-6 months	100	Iron supplementation (Efficacy)	Drops: 2mg/kg	Daily for 2 months	Placebo	
**Nestel, 2004**	cRCT	Sri Lanka	9\-71 months	3228 in total: 745 preschoolers (9-71 months old)	1) Reduced iron fortified wheat flour<br>2) Electrolytic iron fortified wheat flour;<br>We combined groups 1 and 2 (LSFF – efficacy)	1) 66ppm; analysis of iron content: 76 ± 6.6mg/kg<br>2) 66ppm; analysis of iron content: 78 ± 15.2mg/kg	24 months	Unfortified wheat flour	
**Nguyen, 2002**	cRCT	Vietnam	5-12 months	280	Iron supplementation (Efficacy)	2.5mL of syrup: elemental iron (15mg)	Daily for 3 months	Placebo	

**Northrop-Clewes, 1996**	RCT	Pakistan	<2 years	268	Iron supplementation (Efficacy)	Liquid: iron (15mg) as ferrous sulfate	Daily for 12 weeks	Placebo	
**Null, 2018**	cRCT	Kenya	Supplementation given at 6 months of age	762 clusters	LNS (Efficacy and effectiveness)	2 x 10 g sachets of SQ LNS (to be mixed with food): provided 118 kcal per day and 12 essential vitamins and 10 minerals	Daily for 18 months	No intervention	
**Oelofse, 2003**	RCT	South Africa	6-12 months	60	MMN fortified cereal (porridge) (Targeted fortification – efficacy)	60g of dry cereal: energy (1304 kJ), protein (12g), fat (6g), carbohydrates (54.8g), vitamin A (1200IU), vitamin C (40mg), vitamin B1 (0.64mg), vitamin B2, (0.24mg), niacin (3.2mg), calcium (368mg), iron (8mg), vitamin D (160IU), vitamin E (4 IU), biotin (20ug), folic acid (17.6ug), pantothenic acid (0.6mg), vitamin B12 (0.6ug), vitamin B6 (0.24mg), phosphorous (232mg), iodine (26ug), zinc (5.6mg), potassium (632mg), sodium (272mg), chloride (440mg)	Daily for 6 months	Normal diet	
**Ogunlade, 2011**	RCT	South Africa	36-79 months	151	MNP in stiff maize porridge (Efficacy)	35g porridge and MNP: iron (2.86mg), zinc (2.86mg), iodine (34.3ug), calcium (457mg), vitamin A (457 RE), vitamin C (68.6mg), vitamin E (5.71mg), vitamin B12 (1.03ug), thiamine (0.57mg), niacin (6.86mg), riboflavin (0.57mg), folate (103ug), and vitamin B6 (0.57mg).	5 days a week for 11 weeks	Soft maize-meal porridge plus placebo (maize maltodextrin)	28g
**Osei, 2015**	cRCT	Nepal	6-9 months	335	MNP and enhanced homestead food production program (Efficacy)	10 mg iron (encapsulated ferrous fumarate), 4.1 mg zinc (zinc gluconate), 90 g iodine (potassium iodide), 400 g vitamin A (vitamin A acetate), 150 g folic acid, 0.5 mg vitamin B1 (thiamine mononitrate), 0.5 mg vitamin B2 (riboflavin), 0.5 mg vitamin B6 (pyridoxine), 0.9 g vitamin B12	60 sachets were provided within a 6-month period (twice, for a total of 11 months) and participants were instructed to feed MNP on any day of	Enhanced homestead food production program	

						(cyanocobalamin), 30 mg vitamin C (ascorbic acid), 5 g vitamin D3 (cholecalciferol), 5 mg vitamin E (vitamin E acetate), 6 mg niacin (niacinamide) and 0.6 mg copper (cupric gluconate)	their preference, but to ensure not to feed more than one sachet per day.		
**Owino, 2007**	RCT	Zambia	6 or 8 months of age	185	Fortified blend of maize, beans, Bambara nuts, and groundnuts (Targeted fortification – efficacy)	Vitamin A 700 ug, vitamin C 200 mg, vitamin D 10 ug, thiamine 0.9 mg, riboflavin 1.0 mg, pyridoxine 860 ug, folate 220 ug, pantothenic acid 4 mg, calcium 700 mg, phosphorus 550 mg, magnesium 100 mg, electrolytic iron 41.8 mg, zinc oxide 400 mg, copper 400 ug, manganese 1200 ug, selenium 20 ug	Daily for 3 months	No intervention	
**Palupi, 1997**	RCT	Indonesia	2-5 years	299	Iron supplementation (Efficacy)	30g	Weekly for 9 weeks	Placebo	
**Phu, 2010**<br><br>**Associated refs:**<br>**Phu, 2012**	cRCT	Vietnam	5 months	426	1) Fortified instant flour made into energy-dense gruel<br>2) Fortified food complement made into energy-dense gruel<br>We combined groups 1 and 2 (Targeted fortification – efficacy)	1) Raw protein (3.9g), digestible protein (3.6g), lipids (2.24g), vitamin A (38ug RE), ascorbic acid (20mg), thiamin (68ug), riboflavin (95ug), nicotinamide (368ug), folic acid (12ug), pantothenic acid (330ug), vitamin B12 (0.06ug), vitamin B6 (53ug), vitamin K1 (8.4ug), vitamin D (117IU), sodium (73mg), potassium (143mg), chlorine (121mg), calcium (138mg), phosphorus (117mg), magnesium (21.5mg), iron (7.6mg), zinc (1.7mg), iodine (5.1ug), copper (92ug), manganese (319ug), selenium (2.1ug)<br>2) raw protein (9.7g), digestible protein (8.8g), lipids (5.3g), vitamin A (154ug RE), ascorbic acid (87mg), thiamin (229ug), riboflavin (309ug),	Daily for 6 months	Usual care (traditional gruel made at home by parents)	

						nicotinamide (535ug), folic acid (46ug), pantothenic acid (904ug), vitamin B12 (0.16ug), vitamin B6 (98ug), vitamin K1 (38ug), vitamin D (516IU), sodium (343mg), potassium (512mg), chlorine (553mg), calcium (710mg), phosphorus (473mg), magnesium (61mg), iron (33mg), zinc (5.9mg), iodine (22ug), copper (284ug), manganese (942ug), selenium (4.5ug)			
**Radhakrishna, 2013**	RCT	India	4 months	324	Zinc (and riboflavin) supplementation (Efficacy)	0.5mL of syrup: zinc (5mg) as zinc sulfate, riboflavin (0.5mg)	Daily for 14 months (effective duration 12 months)	Placebo (and riboflavin)	0.5mL of syrup: riboflavin (0.5mg)
**Rahman, 1996**<br><br>**Associated refs: Mahalanabis, 1997; Rahman, 1998**	RCT	Bangladesh	6-17 weeks	165	Vitamin A supplementation (Efficacy)	15mg	3 doses at DPT/oral polio (OPV) immunizations (baseline, 4 and 8 weeks)	Placebo	
**Rahman, 2001**<br><br>**Associated refs: Rahman, 2002a; Rahman, 2002b**	RCT	Bangladesh	12-35 months	800	Zinc supplementation and 1 placebo capsule on day 14 (Efficacy)	5mL of syrup: (20mg) elemental zinc	Daily for 14 days	Placebo	
**Rim, 2008**	RCT	North Korea	6-12 month	234	MNP with iron (to be used for home fortification in rice porridge) (Efficacy)	Elemental iron (10mg)	6 days per week for 6 months	Placebo fortified rice porridge	
**Rivera, 2001**<br><br>**Associated refs:**	RCT	Mexico	8-14 months	337	MMN supplementation (Efficacy)	25mL of liquid: 1 Recommended Dietary Allowance (RDA) for children age 1-3 y of vitamins D, E, K, niacin, B-1, B-6,	6 days a week for 12 months	Placebo	

**Rivera, 2001; Rosado, 1999**						folic acid, pantothenic acid, iodine, copper, manganese fluoride and selenium; 1.2 RDA of vitamin A; and 1.5 RDA of ascorbic acid, riboflavin, vitamin B-12, iron and zinc			
**Rivera, 2010**	cRCT	Mexico	12-30 months	795 (12 clusters)	Iron fortified milk (LSFF – efficacy)	Per unit/400mL reconstituted milk (48g powdered milk in 400mL of water): energy (236.8kcal), protein (12.4g), fat (12.4g), carbohydrates (18.6g), sodium (178mg), iron (5.28mg), zinc (5.28mg) vitamin A (216ug), vitamin D (1.8ug), vitamin C (48.0mg), vitamin B12 (0.44ug), folic acid (32.1ug), riboflavin (0.52mg).	Daily for 12 months	Unfortified whole milk (as milk powder)	
**Rosado, 1997**<br><br>**Associated refs:**<br>**Allen, 2000;**<br>**Munoz, 2000;**<br>**Rosado 1999**	RCT	Mexico	18-36 months	219	1) Iron supplementation (Efficacy)<br>2) Zinc supplementation (Efficacy)	1) 20mL of syrup: 20mg<br>2) 20mL of syrup: 20mg	6 days per week for 12 months	Placebo	
**Rosado, 2011**	RCT	Mexico	12-24 months	224	LNS (Oportunidades food supplement) (Efficacy and effectiveness)	44g: energy (194kcal), protein (5.8g), fat (6.6g), carbohydrates (27.9g), Na (24.5mg), Fe (10mg), zinc (10mg), vitamin A (400ug), vitamin E (6mg), vitamin C (40mg), vitamin B12 (0.7ug), folic acid (50ug), riboflavin (0.8mg)	Daily for 6 months	Placebo	
**Ross, 1995**<br><br>**Associated refs:**<br>**Binka, 1995**<br>**Dollimore, 1997;**<br>**Kirkwood, 1996**	Survival study: cRCT	Ghana	Survival study: 6-90 months	Survival study: 21,906 (185 clusters)	Vitamin A supplementation (Efficacy)	30mg retinol equivalents (RE) for children 6-11 mos, (100,000IU); 60mg RE for children aged 12+ mos. (200,000IU)	Survival study: every 4 months for 2 years	Placebo	

**Samuel, 2018**	Controlled before-after study	Ethiopia	6-11 months	2356 (9 districts)	MNP (to be used with complementary food) (Efficacy and effectiveness)	1 sachet: vit A (400mcg), vit D (5mcg), vit E (5mg TE), vit B1 (0.5mg), vit B2 (0.5mg), vit B6 (0.5mg), vit B12 (0.9mcg), niacin (6mg), folate (150mcg), vit C (30mg), iron (6mg), zinc (4.1mg), copper (0.56 mg), selenium (17mcg), iodine (90mcg)	Every other day for 37 weeks	No intervention	
**Sazawal, 2006**<br><br>**Associated refs:**<br>**Olney, 2006; Sazawal, 2007b**	RCT	Tanzania	1-35 months	32,155	1) MMN supplementation (Efficacy)<br>2) IFA supplementation (Efficacy)<br>3) Zinc supplementation (Efficacy)	1) 1 tablet: iron (12.5mg), folic acid (50ug), and zinc (10mg). months received half a tablet.<br>2) 1 tablet: iron (12.5mg), folic acid (50ug<br>3) 1 tablet: zinc (10mg)<br>Children aged 12 months or older received 1 tablet daily while children under 2	Daily until stopping of the trial (max duration of 18 months)	Placebo	
**Sazawal, 2007a**<br><br>**Associated refs:**<br>**Sazawal, 2010**	RCT	India	1-3 years	663	MMN fortified powdered milk (LSFF – efficacy)	Per 3 servings: energy (1890kJ), protein (20.1g), taurine (48mg), carbohydrates (48.9g), fat (18.9g), vitamin A (330ug), vitamin D3 (3.6ug), vitamin E (8.1mg), vitamin C (48mg), thiamin (0.6mg), riboflavin (1.8mg), niacin (4.5mg), vitamin B6 (0.6mg), pantothenic acid (2.7mg), folate (114ug), vitamin B12 (2.7ug), biotin (24.9ug), choline (114mg), calcium (720mg), phosphorus (600mg), magnesium (84mg), iron (9.6mg), zinc (9.6mg), iodine (36ug), selenium (6.6ug), copper (0.3mg), sodium (360mg), potassium (1260mg), chloride (900mg)	3 sachets daily for 12 months	Unfortified powdered milk	
**Sazawal, 2014**	cRCT	India	6-24 months	292 (12 clusters)	1) MNP (Efficacy)<br>2) Rice-based fortified complementary food (Targeted fortification – efficacy)	1) 1 sachet (1.5g) for children >1 year and half a sachet for children <1 year. Per 1.5g sachet: vitamin A (978.26ug), thiamine (0.98mg), riboflavin (1.11mg), niacin (13.04mg), pyridoxine (1.3mg),	Daily for 6 months	No intervention	

						cyanocobalamin (3.91ug), biotin (19.57ug), folic acid (260.87ug), vitamin C (39.13mg), vitamin D (6.52ug), vitamin E (9.78aug), calcium (494.02mg), magnesium (69.13mg), phosphorus (81.52mg), iron (12.5mg), zinc (10mg), copper (1.3mg), manganese (2.58mg), pantothenic acid (6.52mg) 2) 20g sachet for children <1 year and 40g sachet for children >1 year of age, to be reconstituted in water or milk to make 100 or 200ml, in additional to normal diet. Per 20g (100mL mix): energy (80.4kcal), protein (3.0g), fat (1.5g), linoleate (0.18g), vitamin A (1800IU), thiamine (0.51mg), riboflavin (0.58mg), niacin (6.6mg), pyridoxine (0.83mg), cyanocobalamin (0.21mg), biotin (0.11mg), folic acid (0.144mg), vitamin C (23.4mg), vitamin D (145IU), vitamin E (10.5IU), calcium (138.7mg), magnesium (20mg), phosphorus (64mg), iron (7.9mg), zinc (6.5mg), copper (1.0mg), manganese (0.16mg)			
**Semba, 2000**	RCT	Indonesia	3-6 years	236	Vitamin A supplementation (Efficacy)	60mg RE	1 dose	Placebo	
**Sempertegui, 1999**	RCT	Ecuador	6-36 months	400	Vitamin A supplementation (Efficacy)	0.2mL of syrup: 10,000 IU of vitamin A (3000ug of retinol)	Weekly for 40 weeks	Placebo	
**Shamah-Levy, 2008**	RCT	Mexico	12-30 months	486	1) Fortified complementary food based on powdered milk (Nutrisano) with iron as ferrous sulphate	Per portion (44g powder): energy (194kcal), protein (5.8g), carbohydrates (27.9g), fat (6.6g), iron as ferrous sulphate or gluconate (10mg), zinc as zinc oxide (10mg), vitamin A as retinol palmitate	Daily for 6 months	Complementary food without iron	Per portion (44g powder): energy (194kcal), protein (5.8g), carbohydrates (27.9g), fat (6.6g), zinc as zinc oxide (10mg), vitamin A as retinol palmitate (400ug RE), vitamin E as

					2) fortified complementary food as above, but with iron as ferrous gluconate<br>We combined groups 1 and 2 (Targeted fortification – efficacy)	(400ug RE), vitamin E as tocopherol acetate (6mg), vitamin C as ascorbic acid (49.9mg), riboflavin (0.8mg), vitamin B12 as cyanocobalamin (0.7ug), folic acid (0.05mg)			tocopherol acetate (6mg), vitamin C as ascorbic acid (49.9mg), riboflavin (0.8mg), vitamin B12 as cyanocobalamin (0.7ug), folic acid (0.05mg)
**Shankar, 1999**	RCT	Papua New Guinea	6-60 months	480	Vitamin A (and vitamin E) supplementation (Efficacy)	1 capsule for children aged 12 months and above and half a capsule for children below 12 months of age. 1 capsule: vitamin A (200,000IU), vitamin E (10ug)	Every 3 months for 13 months	Placebo	
**Sharieff, 2006**	cRCT	China	3-6 years	415 (16 clusters)	MNP (Efficacy)	1 sachet: iron as encapsulated ferrous fumarate (30mg), zinc gluconate (5mg), vitamin C (50mg), vitamin A (300ug), vitamin D3 (7.5ug) and folic acid (150ug) mixed with semi-solid meal of rice porridge or congee	Daily for 13 weeks	No intervention (children were served congee)	
**Sharma, 2011**	cRCT	India	6-36 months	158 (5 clusters)	IFA supplementation (Efficacy)	1 tablet: elemental iron (19.8mg)	Daily for 60 days	No intervention	
**Siega-Riz, 2014**	cRCT	Honduras	6-18 months	300 mother-child pairs (18 clusters)	LNS (Plumpy'Doz) (Efficacy)	46.3g: energy (247kcal), protein (5.9g), fat (16g), vitamin A (400ug), vitamin B12 (0.9ug), iron (9mg), zinc (9mg)	Daily for 12 months	No intervention	
**Silva, 2006**	RCT	Brazil	12-59 months	60	Zinc supplementation (Efficacy)	5mL of syrup: zinc (10mg) as zinc sulfate	Daily for 4 months	Placebo	
**Smuts, 2005**	RCT	South Africa	6-12 months	265	1) MMN supplementation (Efficacy)<br>2) Iron supplementation (Efficacy)	1) 1 tablet: 1recommended daily allowance (RDA): vitamin A (400ug RE), folic acid (150ug), vitamin D3 (5ug), iodine (90ug), Se (17ug), vitamin B12 (0.9ug), niacin (6mg), Fe (10mg), Zn (4.1mg), Cu (0.56mg), thiamin (0.5mg), riboflavin (0.5mg), vitamin C (30mg),	Daily for 6 months	Placebo	

						vitamin B6 (0.5mg), vitamin E (5mg)<br>2) 1 tablet: iron (10mg)			
**Smuts, 2019**	RCT	South Africa	6 months	750	LNS (Efficacy)	20g: energy (114kcal), protein (3g), fat (8g), linoleic acid (1.5g), alpha-linolenic acid (265mg), linoleic acid: alpha linolenic acid (5.7g), vitamin A (200ug), vitamin D 92.5ug), vitamin E (2.5mg), vitamin K (7.5ug), thiamine (0.25mg), riboflavin (0.25mg), niacin (3mg), pantothenate (1mg), vitamin B6 (0.25mg), biotin (4ug), folate (80ug), vitamin B12 (0.45ug), vitamin C (23.3mg), calcium (250mg), iodine (45ug), iron (5.8mg), zinc (6.2mg), copper (0.28mg), selenium (8.5ug), choline (2.0mg)	Daily for 6 months	No intervention	
**Somasse, 2018**	cRCT	Mali	6-23 months	722 (40 clusters)	MNP (Efficacy and effectiveness)	1 sachet: vitamin A (400ug RE), folic acid (150ug), vitamin D3 (5ug), iodine (90ug), Se (17ug), vitamin B12 (0.9ug), niacin (6mg), Fe (10mg), Zn (4.1mg), Cu (0.56mg), thiamin (0.5mg), riboflavin (0.5mg), vitamin C (30mg), vitamin B6 (0.5mg), vitamin E (5mg)	Daily for 3 months	No intervention	
**Soofi, 2013**<br><br>**Associated refs:**<br>**Ariff, 2014**	cRCT	Pakistan	<5 months	2746 (256 clusters)	MNP (Efficacy)	1 sachet: iron (12.5mg), vitamin C (50mg), vitamin A (300ug), vitamin D (5ug), and folic acid (150ug) and zinc (10mg)	Daily for 12 months	No intervention	
**Stoltzfus, 2001**<br><br>**Associated refs:**<br>**Stoltzfus, 2004**	RCT	Tanzania	5-59 months	684	Iron supplementation (Efficacy)	0.mL of syrup: 10mg iron as ferrous sulphate	Daily for 12 months	Placebo	

**Suchdev, 2012**	cRCT	Kenya	6-35 months	1063	MNP (Efficacy and effectiveness)	1 sachet: 12.5 mg Fe as microencapsulated ferrous fumarate, 375 ug vitamin A, 5 mg Zn, 150 ug folic acid; 35 mg vitamin C, 5 ug vitamin D3, 6 mg vitamin E, 6 mg niacin; 0.6 mg Cu, 50 ug iodine, 0.5 mg thiamine, riboflavin, and vitamin B-6, and 0.9 mg vitamin B-12	12 months	Placebo	
**Surono, 2014**	RCT	Indonesia	12-24 months	48	Zinc supplementation (Efficacy)	20mg as zinc sulfate monohydrate= 8mg zinc elemental	Daily for 90 days	Placebo	
**Thu, 1999**	RCT	Vietnam	6-24 months	168	MMN supplementation (Efficacy)	1mL of syrup: retinol as retinyl acetate (333ug), iron (8mg), zinc (5mg), vitamin C (20mg)	5 days per week for 12 weeks	Placebo	
**Tielsch, 2006**<br><br>**Associated refs: Christian, 2011; Katz, 2010; Murray-Kolb, 2012; Surkan, 2012; Surkan, 2013 Tielsch, 2007**	cRCT	Nepal	1-35 months	43,124	1) IFA supplementation (Efficacy)<br>2) MMN supplementation (Efficacy)<br>3) Zinc supplementation (efficacy)	1) 1 tablet: iron (12.5mg), folic acid (50ug) (half a tablet if <1-year-old)<br>2) 1 tablet: iron (12.5mg), folic acid (50ug), zinc (10mg) (half a tablet if <1-year-old)<br>3) 1 tablet: zinc (10mg) tablet (half a tablet if <1-year-old)	Enrollment until 36 months of age	Placebo	
**Umeta, 2000**<br><br>**Associated refs: Umeta, 2003**	RCT	Ethiopia	6-12 months	200	Zinc supplementation (Efficacy)	3mL of syrup: zinc as zinc sulphate (10mg)	6 days a week for 6 months	Placebo	
**Untoro, 2005**<br><br>**Associated refs: Wijaya-Erhardt, 2007**	RCT	Indonesia	6-12 months	284	1) MMN supplementation (Efficacy)<br>2) Iron supplementation (Efficacy)	1) 1 foodlet: 1 RDA: vitamin A as retinyl acetate (375ug RE), vitamin D (5ug), vitamin E (6mg α-tocopherol equivalents), vitamin K (10ug), vitamin C (35mg), thiamine (0.5mg), riboflavin (0.5mg), vitamin B-6 (0.5mg), vitamin B-12 (0.9ug) , niacin as niacinamide (6mg),folate	Daily for 23 weeks	Placebo	

						(150ug), iron as ferrous fumarate (10mg), zinc as zinc gluconate (5mg), copper as cupric gluconate (0.6mg), and iodine as potassium iodide (59ug)<br>2) Iron as ferrous sulfate (10mg)			
**Varma, 2007**	cRCT	India	36-66 months	684 (30 clusters)	MNP (to be mixed with local khichdi) (Efficacy)	200g of khichdi: ferrous fumarate (14mg), vitamin A retinyl acetate (500IU), folic acid (0.05mg)	6 days a week for 24 weeks	Unfortified khichdi with placebo premix	
**Vijay, 2014**	cRCT	India	6-36 months	1294	MNP (to be mixed with supplementary baby mix) (Efficacy)	1 sachet: 12mg ferrous fumarate, 300ug vitamin A (retinyl acetate), 5mg zinc oxide, 30mg vitamin C, 50ug folic acid and dextrose	5 days a week for 6 months	Supplementary baby mix	
**Villalpando, 2006**	RCT	Mexico	10-30 months	130	MMN fortified milk (LSFF – efficacy)	400 mL per day. Per unit/400mL reconstituted milk (48g powdered milk in 400mL of water): energy (992kJ), protein (12.5g), fat (12.5g), carbohydrates (18.7g), iron as ferrous gluconate (5.28mg), zinc as zinc oxide (5.28mg), retinol palmitate (21.6ug), vitamin C as sodium ascorbate (48mg), folic acid (32.1ug)	Daily for 6 months	Unfortified cow's whole milk (as milk powder)	
**Wasantwisut, 2006**<br><br>**Associated refs: Pongcharoen, 2011**	RCT	Thailand	4-6 months	674	1) Iron supplementation (Efficacy)<br>2) Zinc supplementation (Efficacy)	1) 2mL of syrup: zinc (10mg)<br>2) 2mL of syrup: zinc (10mg)	Daily for 6 months	Placebo	
**Wessells, 2012**	RCT	Burkina Faso	6-23 months	451	1) Zinc (liquid) supplementation<br>2) Zinc (tablet) supplementation<br>We combined groups 1 and 2 (Efficacy)	1) 5 mL: zinc as zinc sulfate (5mg)<br>2) 1 tablet: zinc as zinc sulfate (5mg)	Daily for 3 weeks	Placebo	

**West, 1995**<br><br>**Associated refs:**<br>**Bishai, 2005;**<br>**Katz, 1995**	cRCT	Nepal	6-72 months	11918 (261 wards)	Vitamin A (and vitamin E) supplementation (Efficacy)	30000ug RE (100,000IU) for children 6-11 months old and 60000ug RE (200,000IU) for children 12 months or older; vitamin E (40IU) as antioxidant	Every 4 months for 12 months	Placebo	
**Wieringa, 2003**	RCT	Indonesia	4 months	387	1) Iron supplementation (Efficacy)<br>2) Zinc supplementation (Efficacy)	1) 2mL of syrup: 10mg iron (ferrous sulfate<br>2) 12mL of syrup: 10mg zinc (zinc sulfate)	5 days a week for 6 months	Placebo	
**Yurdakok, 2004**	RCT	Turkey	4 months	79	Iron supplementation (Efficacy)	1 mg/kg iron - adjusted monthly according to infant weight	Daily for 3 months	No intervention	
**Zlotkin, 2003**	RCT	Ghana	8-20 months	437	MNP (with iron only) (Efficacy)	40mg	Daily for 6 months	Placebo	

Appendix 4 – Additional Forest Plots

<u>Comparison 1: Vitamin A Supplementation vs. Placebo/No Intervention (Efficacy)</u>

All-cause mortality (incidence)

Study or Subgroup	log[Rate Ratio]	SE	Weight	Rate Ratio IV, Random, 95% CI
Anonymous 1998	-0.0408	0.1397	39.2%	0.96 [0.73, 1.26]
Fisker 2013	-0.0943	0.2211	15.7%	0.91 [0.59, 1.40]
West 1995	0.1044	0.1302	45.1%	1.11 [0.86, 1.43]
**Total (95% CI)**			**100.0%**	**1.02 [0.86, 1.21]**

Heterogeneity: Tau² = 0.00; Chi² = 0.88, df = 2 (P = 0.65); I² = 0%
Test for overall effect: Z = 0.19 (P = 0.85)

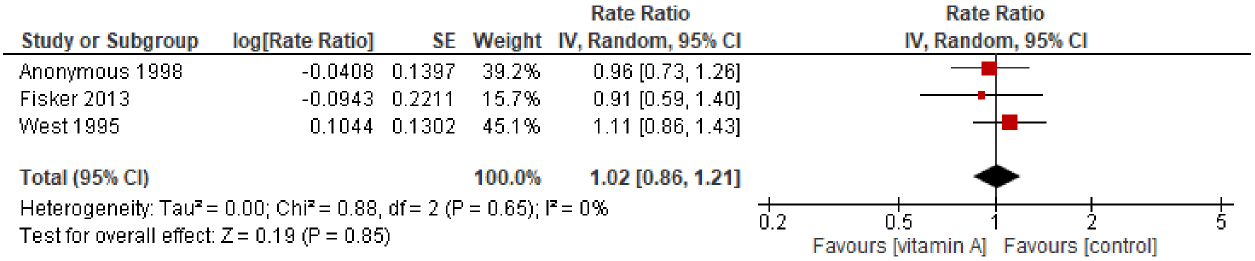

All-cause mortality (cumulative incidence)

Study or Subgroup	log[Risk Ratio]	SE	Weight	Risk Ratio IV, Random, 95% CI
Agarwal 1995	-0.1363	0.4988	1.6%	0.87 [0.33, 2.32]
Awasthi 2013	-0.0408	0.0386	64.9%	0.96 [0.89, 1.04]
Ayah 2007	-0.5114	0.445	2.0%	0.60 [0.25, 1.43]
Ross 1995	-0.2107	0.0893	31.6%	0.81 [0.68, 0.96]
**Total (95% CI)**			**100.0%**	**0.90 [0.80, 1.02]**

Heterogeneity: Tau² = 0.00; Chi² = 4.06, df = 3 (P = 0.26); I² = 26%
Test for overall effect: Z = 1.67 (P = 0.10)

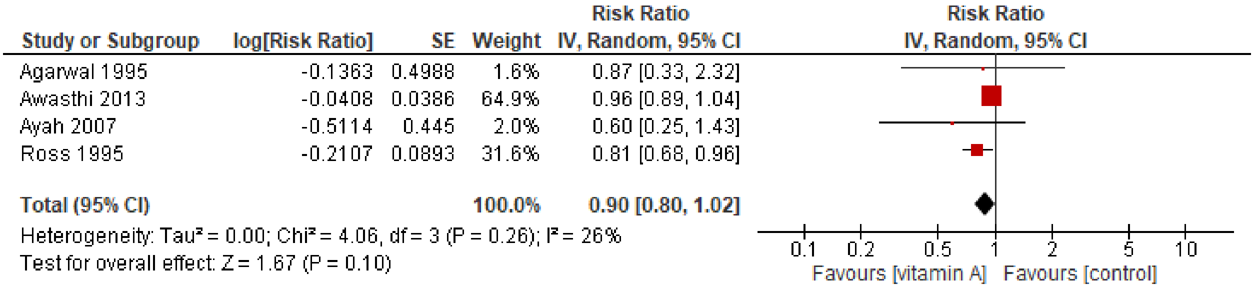

Serum/plasma retinol (μmol/L)

Study or Subgroup	Vitamin A supplementation Mean	SD	Total	Control Mean	SD	Total	Weight	Mean Difference IV, Random, 95% CI
Drammeh 2002	0.68	0.0656	43	0.65	0.0632	40	34.0%	0.03 [0.00, 0.06]
Semba 2000	1.67	0.46	59	0.77	0.23	58	32.3%	0.90 [0.77, 1.03]
Shankar 1999	0.76	0.25	125	0.67	0.27	140	33.7%	0.09 [0.03, 0.15]
**Total (95% CI)**			**227**			**238**	**100.0%**	**0.33 [0.01, 0.65]**

Heterogeneity: Tau² = 0.08; Chi² = 161.53, df = 2 (P < 0.00001); I² = 99%
Test for overall effect: Z = 2.01 (P = 0.04)

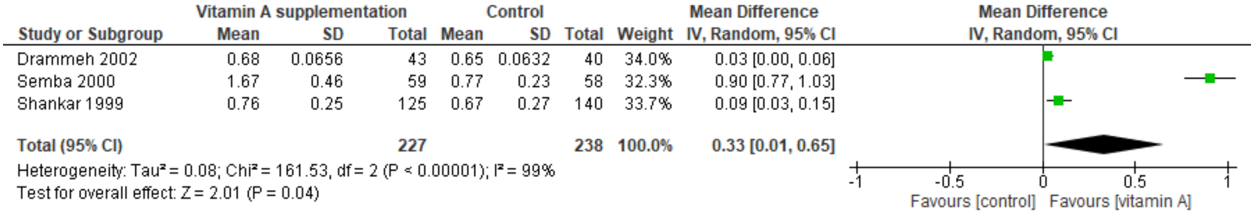

Diarrhea (incidence)

Study or Subgroup	log[Rate Ratio]	SE	Weight	Rate Ratio IV, Random, 95% CI
Chen 2013	-0.6931	0.212	8.3%	0.50 [0.33, 0.76]
Dibley 1996	0.0583	0.0723	17.6%	1.06 [0.92, 1.22]
Fisker 2013	-0.0202	0.1433	12.3%	0.98 [0.74, 1.30]
Long 2006	0.239	0.0733	17.5%	1.27 [1.10, 1.47]
Long 2007	-0.2357	0.1404	12.5%	0.79 [0.60, 1.04]
Rahman 1996	-0.0202	0.108	14.9%	0.98 [0.79, 1.21]
Sempértegui 1999	0.077	0.0818	16.9%	1.08 [0.92, 1.27]
**Total (95% CI)**			**100.0%**	**0.97 [0.83, 1.14]**

Heterogeneity: Tau² = 0.03; Chi² = 23.86, df = 6 (P = 0.0006); I² = 75%
Test for overall effect: Z = 0.35 (P = 0.73)

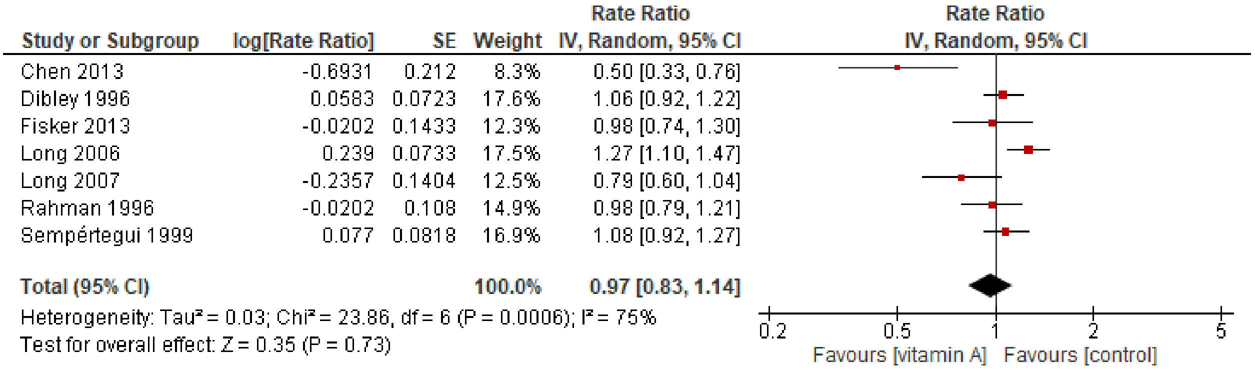

Lower respiratory tract infection (incidence)

Study or Subgroup	log[Rate Ratio]	SE	Weight	Rate Ratio IV, Random, 95% CI
Dibley 1996	0.3293	0.1665	24.7%	1.39 [1.00, 1.93]
Fisker 2013	-0.0619	0.0635	43.7%	0.94 [0.83, 1.06]
Long 2006	0.6831	0.5605	4.0%	1.98 [0.66, 5.94]
Long 2007	0.0296	0.379	7.9%	1.03 [0.49, 2.17]
Sempértegui 1999	0.1906	0.2048	19.6%	1.21 [0.81, 1.81]
**Total (95% CI)**			**100.0%**	**1.13 [0.90, 1.42]**

Heterogeneity: Tau² = 0.03; Chi² = 7.18, df = 4 (P = 0.13); I² = 44%
Test for overall effect: Z = 1.04 (P = 0.30)

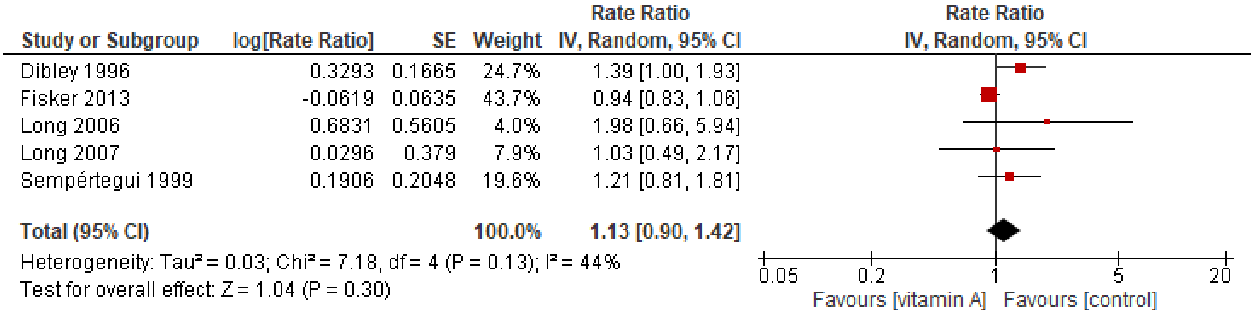

<u>Comparison 2: Zinc Supplementation vs. Placebo/No Intervention (Efficacy)</u>

All-cause mortality (incidence)

Study or Subgroup	log[Rate Ratio]	SE	Weight	Rate Ratio IV, Random, 95% CI
Brooks 2005	-1.8971	0.8212	28.5%	0.15 [0.03, 0.75]
Sazawal 2006	-0.0726	0.0705	35.8%	0.93 [0.81, 1.07]
Tielsch 2006	-2.3862	0.0871	35.7%	0.09 [0.08, 0.11]
**Total (95% CI)**			**100.0%**	**0.24 [0.04, 1.62]**

Heterogeneity: Tau² = 2.63; Chi² = 427.51, df = 2 (P < 0.00001); I² = 100%
Test for overall effect: Z = 1.46 (P = 0.14)

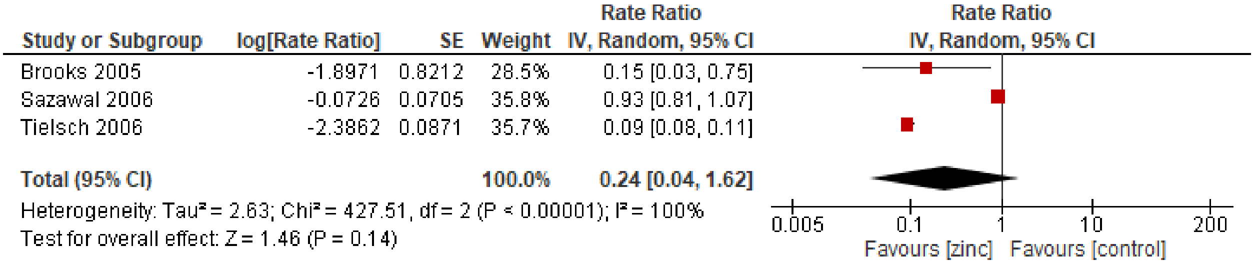

Anemia

Study or Subgroup	Experimental Events	Experimental Total	Control Events	Control Total	Weight	Risk Ratio M-H, Random, 95% CI
Barffour 2019	324	722	313	726	15.9%	1.04 [0.93, 1.17]
Berger 2006	59	191	59	195	2.4%	1.02 [0.76, 1.38]
Chang 2010	78	85	77	89	19.9%	1.06 [0.96, 1.18]
Dijkhuizen 2001	60	97	57	87	4.5%	0.94 [0.76, 1.17]
Fahmida 2007	140	153	138	150	47.1%	0.99 [0.93, 1.06]
Lind 2003	48	134	63	143	2.5%	0.81 [0.61, 1.09]
Sazawal 2006 (1)	29	44	44	58	3.2%	0.87 [0.67, 1.12]
Silva 2006	6	28	3	30	0.1%	2.14 [0.59, 7.76]
Wasantwisut 2006	40	58	40	66	3.2%	1.14 [0.88, 1.48]
Wieringa 2003	24	48	21	43	1.2%	1.02 [0.67, 1.55]
**Total (95% CI)**		**1560**		**1587**	**100.0%**	**1.01 [0.96, 1.06]**
Total events	808		815			

Heterogeneity: Tau² = 0.00; Chi² = 7.23, df = 9 (P = 0.61); I² = 0%
Test for overall effect: Z = 0.38 (P = 0.70)

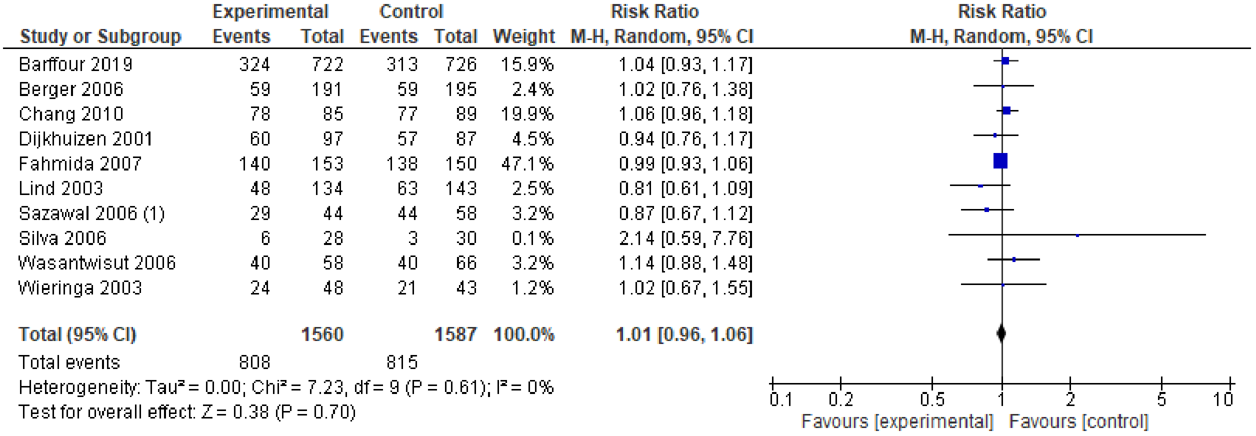

<u>Footnotes</u>
(1) hemoglobin concentration <100 g/L

Stunting

Study or Subgroup	Zinc supplementation Events	Zinc supplementation Total	Control Events	Control Total	Weight	Risk Ratio M-H, Random, 95% CI
Barffour 2019	355	739	317	740	43.4%	1.12 [1.00, 1.25]
Berger 2006	39	191	50	195	9.6%	0.80 [0.55, 1.15]
Dijkhuizen 2001	18	98	22	90	4.6%	0.75 [0.43, 1.31]
Fahmida 2007	46	189	54	192	11.1%	0.87 [0.62, 1.21]
Lind 2003	17	162	14	164	3.2%	1.23 [0.63, 2.41]
Radhakrishna 2013	95	154	91	147	28.1%	1.00 [0.83, 1.19]
**Total (95% CI)**		**1533**		**1528**	**100.0%**	**1.00 [0.89, 1.14]**
Total events	570		548			

Heterogeneity: Tau² = 0.01; Chi² = 6.72, df = 5 (P = 0.24); I² = 26%
Test for overall effect: Z = 0.07 (P = 0.95)

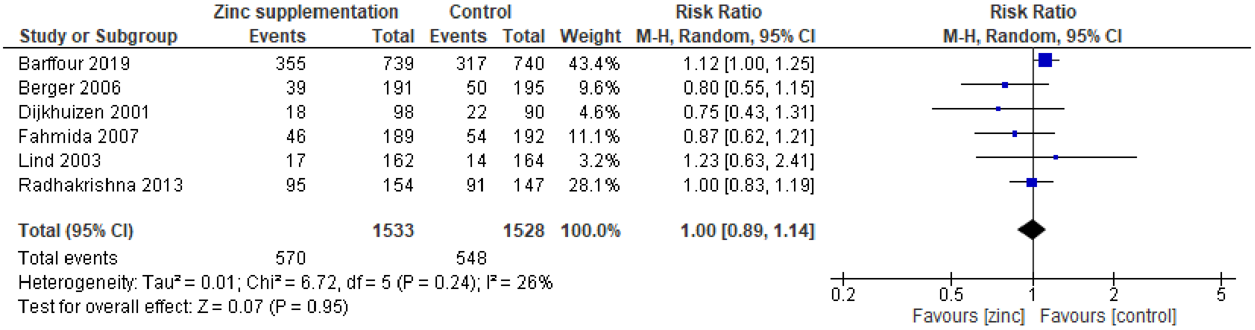

Wasting

Study or Subgroup	Experimental Events	Experimental Total	Control Events	Control Total	Weight	Risk Ratio M-H, Random, 95% CI
Barffour 2019	38	739	35	740	34.8%	1.09 [0.69, 1.70]
Berger 2006	6	191	8	195	6.5%	0.77 [0.27, 2.17]
Dijkhuizen 2001	1	98	0	90	0.7%	2.76 [0.11, 66.84]
Fahmida 2007	19	189	26	192	22.5%	0.74 [0.43, 1.29]
Lind 2003	18	162	30	164	23.7%	0.61 [0.35, 1.04]
Radhakrishna 2013	10	154	15	147	11.8%	0.64 [0.30, 1.37]
**Total (95% CI)**		**1533**		**1528**	**100.0%**	**0.80 [0.62, 1.05]**
Total events	92		114			

Heterogeneity: Tau² = 0.00; Chi² = 3.79, df = 5 (P = 0.58); I² = 0%
Test for overall effect: Z = 1.63 (P = 0.10)

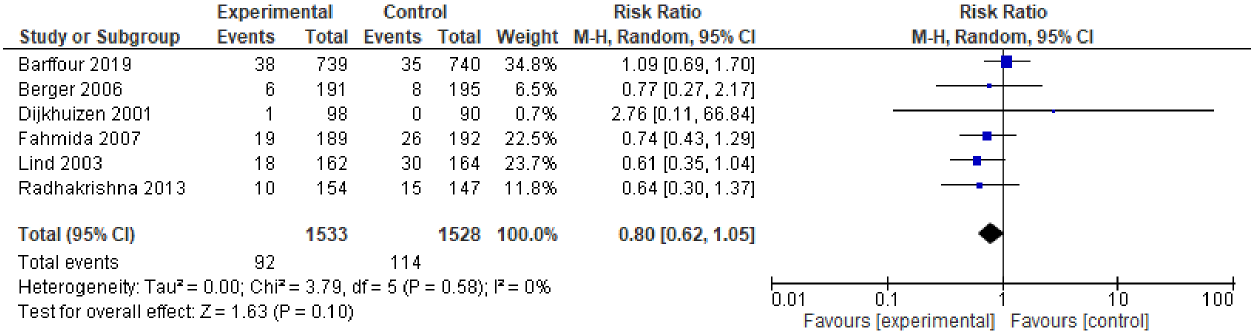

Underweight

Study or Subgroup	Zinc supplementation Events	Zinc supplementation Total	Control Events	Control Total	Weight	Risk Ratio M-H, Random, 95% CI
Barffour 2019	217	739	203	740	48.3%	1.07 [0.91, 1.26]
Fahmida 2007	74	189	80	192	21.3%	0.94 [0.74, 1.20]
Lind 2003	53	162	65	164	15.0%	0.83 [0.62, 1.10]
Radhakrishna 2013	59	154	56	147	15.4%	1.01 [0.75, 1.34]
**Total (95% CI)**		**1244**		**1243**	**100.0%**	**0.99 [0.89, 1.11]**
Total events	403		404			

Heterogeneity: Tau² = 0.00; Chi² = 2.59, df = 3 (P = 0.46); I² = 0%
Test for overall effect: Z = 0.14 (P = 0.89)

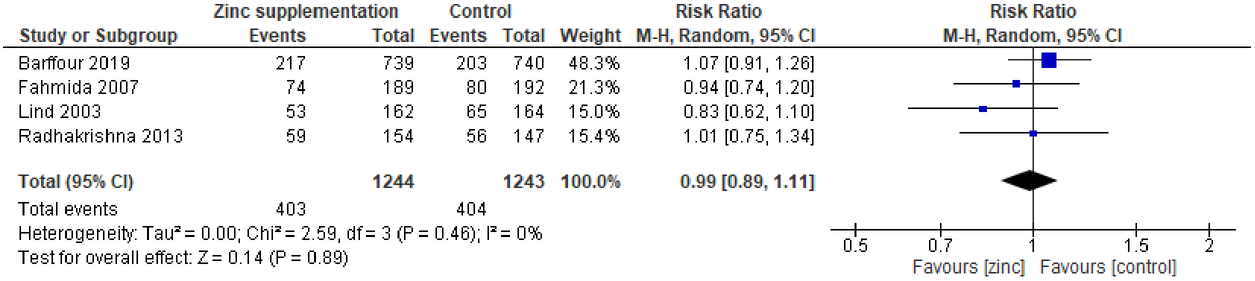

Height (cm)

Study or Subgroup	Zinc supplementation Mean	Zinc supplementation SD	Zinc supplementation Total	Control Mean	Control SD	Control Total	Weight	Mean Difference IV, Random, 95% CI
Baqui 2003	70.9	2.8	141	71	2.6	140	4.5%	-0.10 [-0.73, 0.53]
Barffour 2019	79	4.8	739	79.3	4.9	740	6.2%	-0.30 [-0.79, 0.19]
Becquey 2016	9.6	2.9661	261	9.8	4.0321	641	6.5%	-0.20 [-0.68, 0.28]
Bentley 1997	6.1	2	45	5.7	2	44	2.9%	0.40 [-0.43, 1.23]
Berger 2006	71.15	2.53	191	71.01	2.41	195	6.2%	0.14 [-0.35, 0.63]
Bhandari 2002	3.43	1.58	1093	3.55	1.74	1133	14.1%	-0.12 [-0.26, 0.02]
Brooks 2005	75.2	3.5	329	74.3	4	309	5.0%	0.90 [0.32, 1.48]
Dijkhuizen 2001	69.4	2.3	98	69.1	2.6	90	3.8%	0.30 [-0.40, 1.00]
Hamadani 2001	11.5	1.9	138	11.4	2.1	133	6.5%	0.10 [-0.38, 0.58]
Kikafunda 1998	108.1	5.5	59	107.95	5.4	54	0.6%	0.15 [-1.86, 2.16]
Lind 2003	72.4	2.8	134	72.4	2.8	143	4.2%	0.00 [-0.66, 0.66]
Mazariegos 2010	68.2	2.6	188	68.7	2.5	196	6.0%	-0.50 [-1.01, 0.01]
Muller 2001	5	2.2	332	4.9	2.1	329	9.4%	0.10 [-0.23, 0.43]
Radhakrishna 2013	77.4	2.82	154	77.5	3.53	147	3.7%	-0.10 [-0.82, 0.62]
Rahman 2001	4.29	1.54	165	4.25	1.4	160	9.6%	0.04 [-0.28, 0.36]
Rosado 1997	9.3	1.4549	48	9.1	1.5082	47	4.9%	0.20 [-0.40, 0.80]
Umeta 2000	6.7957	6.4073	92	3.9728	5.8253	92	0.8%	2.82 [1.05, 4.59]
Wasantwisut 2006	70.6	2.5	151	70.6	2.4	153	5.4%	0.00 [-0.55, 0.55]
**Total (95% CI)**			**4358**			**4746**	**100.0%**	**0.04 [-0.12, 0.20]**

Heterogeneity: Tau² = 0.04; Chi² = 30.33, df = 17 (P = 0.02); I² = 44%
Test for overall effect: Z = 0.52 (P = 0.60)

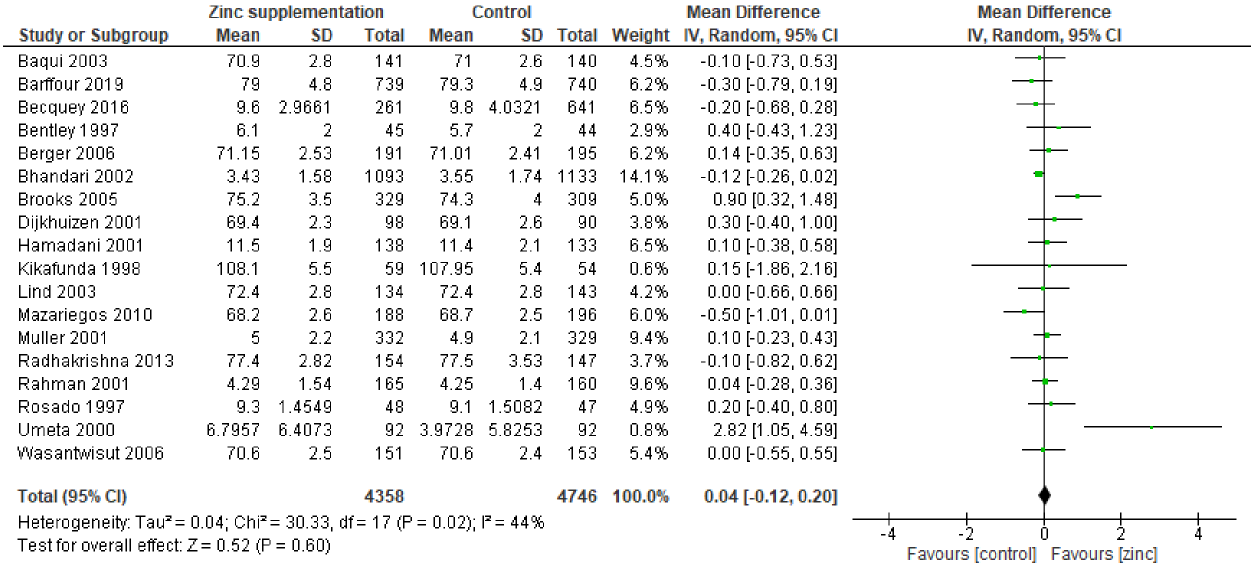

Weight (kg)

Study or Subgroup	Zinc supplementation Mean	SD	Total	Control Mean	SD	Total	Weight	Mean Difference IV, Random, 95% CI
Baqui 2003	8.08	1.07	141	8.16	1.01	140	2.8%	-0.08 [-0.32, 0.16]
Barffour 2019	9.52	1.25	739	9.63	1.32	740	7.7%	-0.11 [-0.24, 0.02]
Becquey 2016	2.1	0.8241	261	2.1	0.9305	641	8.3%	0.00 [-0.12, 0.12]
Bentley 1997	1.03	0.54	45	0.97	0.51	44	3.4%	0.06 [-0.16, 0.28]
Berger 2006	8.19	0.92	191	8.05	0.8	195	5.1%	0.14 [-0.03, 0.31]
Bhandari 2002	0.67	0.47	1093	0.68	0.49	1133	21.5%	-0.01 [-0.05, 0.03]
Brooks 2005	8.7	1.1	329	8.5	1.2	309	4.7%	0.20 [0.02, 0.38]
Dijkhuizen 2001	8	0.9	98	7.8	1	90	2.3%	0.20 [-0.07, 0.47]
Hamadani 2001	2.85	0.73	138	2.79	0.72	133	5.0%	0.06 [-0.11, 0.23]
Kikafunda 1998	18.06	2.1	59	17.95	2.1	54	0.3%	0.11 [-0.67, 0.89]
Lind 2003	8.48	0.97	134	8.31	0.94	143	3.2%	0.17 [-0.06, 0.40]
Mazariegos 2010	8	1	188	8.1	1	196	3.9%	-0.10 [-0.30, 0.10]
Muller 2001	1	0.7	332	0.9	0.7	329	10.1%	0.10 [-0.01, 0.21]
Radhakrishna 2013	8.85	1.005	154	8.79	0.988	147	3.2%	0.06 [-0.17, 0.29]
Rahman 2001	0.747	0.485	165	0.781	0.461	160	10.5%	-0.03 [-0.14, 0.07]
Rosado 1997	2.4	0.485	48	2.4	0.4799	47	4.1%	0.00 [-0.19, 0.19]
Umeta 2000	1.4541	2.6451	92	0.9858	2.3968	92	0.3%	0.47 [-0.26, 1.20]
Wasantwisut 2006	8	0.9	151	8.1	1	153	3.5%	-0.10 [-0.31, 0.11]
**Total (95% CI)**			**4358**			**4746**	**100.0%**	**0.02 [-0.02, 0.06]**

Heterogeneity: Tau² = 0.00; Chi² = 22.96, df = 17 (P = 0.15); I² = 26%
Test for overall effect: Z = 0.99 (P = 0.32)

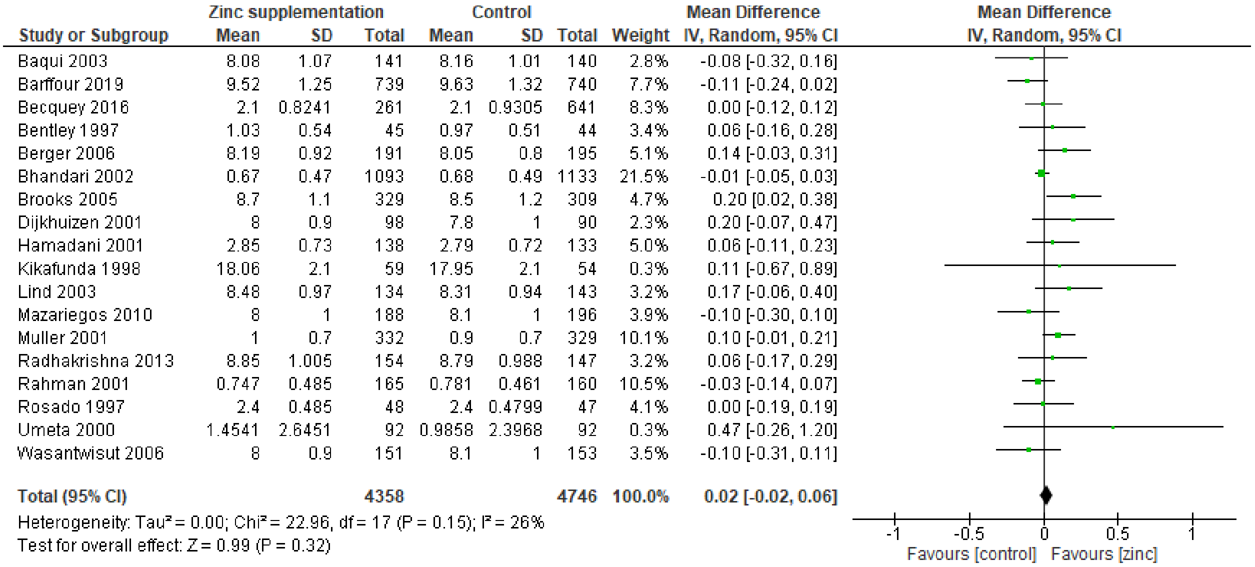

Head circumference (cm)

Study or Subgroup	Zinc supplementation Mean	SD	Total	Control Mean	SD	Total	Weight	Mean Difference IV, Random, 95% CI
Bentley 1997	2	2	45	1.9	0.8	44	7.9%	0.10 [-0.53, 0.73]
Hamadani 2001	5.1	1.1	138	5.2	1.1	133	45.7%	-0.10 [-0.36, 0.16]
Mazariegos 2010	44.3	1.3	188	44.5	1.3	196	46.4%	-0.20 [-0.46, 0.06]
**Total (95% CI)**			**371**			**373**	**100.0%**	**-0.13 [-0.31, 0.05]**

Heterogeneity: Tau² = 0.00; Chi² = 0.84, df = 2 (P = 0.66); I² = 0%
Test for overall effect: Z = 1.44 (P = 0.15)

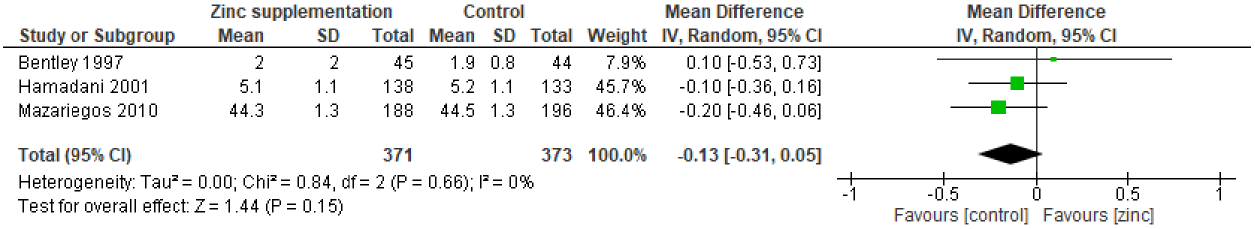

Mid-upper arm circumference (cm)

Study or Subgroup	Zinc supplementation Mean	SD	Total	Control Mean	SD	Total	Weight	Mean Difference IV, Random, 95% CI
Adu-Afarwuah 2007	0	0	0	0	0	0		Not estimable
Baqui 2003	13.62	1.02	141	13.67	1.03	140	9.8%	-0.05 [-0.29, 0.19]
Barffour 2019	14	0.9	739	14	0.9	740	29.5%	0.00 [-0.09, 0.09]
Bentley 1997	0.2	0.8	45	0	1	44	4.6%	0.20 [-0.18, 0.58]
Hamadani 2001	2.5	1.1	138	2.6	1	133	9.2%	-0.10 [-0.35, 0.15]
Kikafunda 1998	16.51	1.26	59	16.56	1.12	54	3.4%	-0.05 [-0.49, 0.39]
Lind 2003	14.8	1.14	162	14.7	1.18	164	9.1%	0.10 [-0.15, 0.35]
Muller 2001	-0.3	1	332	-0.4	1.1	329	17.3%	0.10 [-0.06, 0.26]
Rosado 1997	0.93	0.1371	47	0.67	0.5485	47	17.1%	0.26 [0.10, 0.42]
Umeta 2000	0.2467	8.4278	92	0.1467	8.4495	92	0.1%	0.10 [-2.34, 2.54]
**Total (95% CI)**			**1755**			**1743**	**100.0%**	**0.06 [-0.02, 0.15]**

Heterogeneity: Tau² = 0.00; Chi² = 10.99, df = 8 (P = 0.20); I² = 27%
Test for overall effect: Z = 1.48 (P = 0.14)

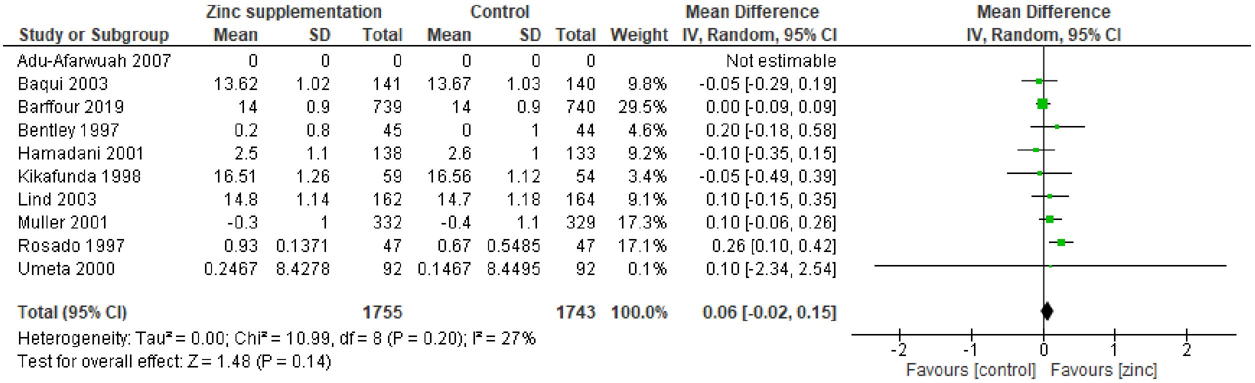

Length-for-age (z-score)

Study or Subgroup	Zinc supplementation Mean	SD	Total	Control Mean	SD	Total	Weight	Mean Difference IV, Random, 95% CI
Baqui 2003	-1.6	0.9	49	-1.7	1	45	0.4%	0.10 [-0.29, 0.49]
Barffour 2019	-1.93	0.97	739	-1.93	1	740	6.4%	0.00 [-0.10, 0.10]
Becquey 2016	-0.4	0.8898	261	-0.32	1.2406	641	3.1%	-0.08 [-0.22, 0.06]
Bentley 1997	-0.74	0.68	45	-0.89	0.74	44	0.7%	0.15 [-0.15, 0.45]
Berger 2006	-0.37	0.39	191	-0.42	0.46	195	8.9%	0.05 [-0.04, 0.14]
Bhandari 2002	-0.14	0.44	1093	-0.12	0.43	1133	49.3%	-0.02 [-0.06, 0.02]
Dijkhuizen 2001	-1.29	0.79	98	-1.3	0.88	90	1.1%	0.01 [-0.23, 0.25]
Fahmida 2007	-1.39	0.96	189	-1.47	0.94	192	1.8%	0.08 [-0.11, 0.27]
Hamadani 2001	-2.3	1	97	-2.4	1	101	0.8%	0.10 [-0.18, 0.38]
Kikafunda 1998	-0.5	0.92	59	-0.48	0.95	54	0.5%	-0.02 [-0.37, 0.33]
Lind 2003	-0.77	0.92	162	-0.81	0.86	164	1.7%	0.04 [-0.15, 0.23]
Mazariegos 2010	-2.57	1.11	188	-2.52	1.13	196	1.3%	-0.05 [-0.27, 0.17]
McDonald 2015	-0.93	1.2062	582	-0.93	1.2083	584	3.4%	0.00 [-0.14, 0.14]
Muller 2001	0.06	0.8	332	-0.02	0.8	329	4.3%	0.08 [-0.04, 0.20]
Rahman 2001	0.1	0.51	165	0.06	0.5	160	5.3%	0.04 [-0.07, 0.15]
Rosado 1997	0.16	0.3464	48	0.13	0.4113	47	2.8%	0.03 [-0.12, 0.18]
Sazawal 2006	-0.43	0.26	44	-0.35	0.26	58	6.2%	-0.08 [-0.18, 0.02]
Silva 2006	-1.7	2.6	28	-1.6	1.6	30	0.1%	-0.10 [-1.22, 1.02]
Umeta 2000	-0.0235	2.6584	92	-0.9846	3.0805	92	0.1%	0.96 [0.13, 1.79]
Wasantwisut 2006	-1	0.9	151	-0.99	0.86	153	1.6%	-0.01 [-0.21, 0.19]
**Total (95% CI)**			**4613**			**5048**	**100.0%**	**-0.00 [-0.03, 0.02]**

Heterogeneity: Tau² = 0.00; Chi² = 16.31, df = 19 (P = 0.64); I² = 0%
Test for overall effect: Z = 0.15 (P = 0.88)

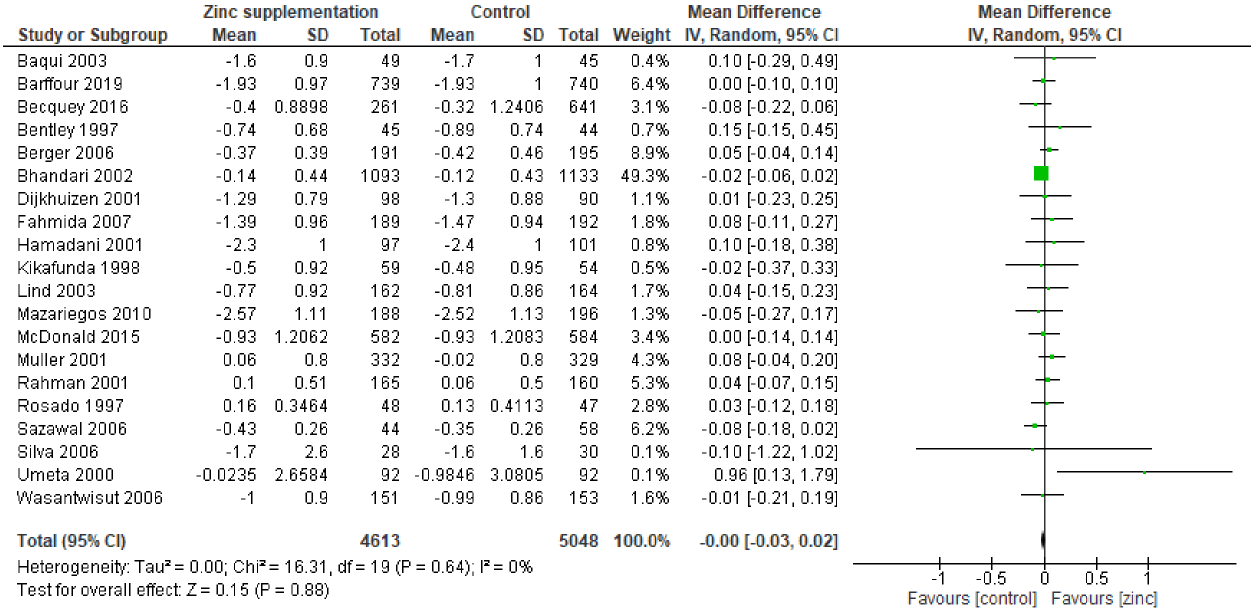

Weight-for-age (z-score)

Study or Subgroup	Zinc supplementation Mean	SD	Total	Control Mean	SD	Total	Weight	Mean Difference IV, Random, 95% CI
Baqui 2003	-2	1.2	49	-2	1.2	45	4.2%	0.00 [-0.49, 0.49]
Barffour 2019	-1.51	0.88	739	-1.52	0.9	740	5.7%	0.01 [-0.08, 0.10]
Becquey 2016	0.01	0.5932	261	0.08	0.6203	641	5.7%	-0.07 [-0.16, 0.02]
Bentley 1997	-0.87	0.55	45	-0.96	0.56	44	5.3%	0.09 [-0.14, 0.32]
Berger 2006	-0.97	0.41	191	-1.13	0.46	195	5.7%	0.16 [0.07, 0.25]
Bhandari 2002	-0.04	0.51	1093	-0.06	0.49	1133	5.8%	0.02 [-0.02, 0.06]
Dijkhuizen 2001	-1.26	0.85	98	-1.37	0.91	90	5.2%	0.11 [-0.14, 0.36]
Fahmida 2007	-1.71	0.89	189	-1.86	0.93	192	5.5%	0.15 [-0.03, 0.33]
Hamadani 2001	-2.4	0.9	97	-2.5	0.9	101	5.2%	0.10 [-0.15, 0.35]
Kikafunda 1998	-0.27	0.88	59	-0.27	0.7	54	5.1%	0.00 [-0.29, 0.29]
Lind 2003	-1.46	1.08	162	1.72	1	164	5.3%	-3.18 [-3.41, -2.95]
Mazariegos 2010	-1.4	1.12	188	-1.3	1.08	196	5.4%	-0.10 [-0.32, 0.12]
McDonald 2015	-0.61	0.965	582	-0.5	0.9666	584	5.7%	-0.11 [-0.22, 0.00]
Muller 2001	-0.002	0.6	332	-0.08	0.6	329	5.7%	0.08 [-0.01, 0.17]
Rahman 2001	-0.03	0.37	165	-0.03	0.35	160	5.7%	0.00 [-0.08, 0.08]
Rosado 1997	0.26	0.3464	48	0.25	0.3428	47	5.6%	0.01 [-0.13, 0.15]
Sazawal 2006	-0.22	0.46	44	-0.15	0.44	58	5.5%	-0.07 [-0.25, 0.11]
Umeta 2000	0.0177	3.3247	92	-0.372	3.7455	92	2.1%	0.39 [-0.63, 1.41]
Wasantwisut 2006	-1.3	0.9	151	-1.3	0.9	153	5.4%	0.00 [-0.20, 0.20]
**Total (95% CI)**			**4585**			**5018**	**100.0%**	**-0.14 [-0.33, 0.04]**

Heterogeneity: Tau² = 0.15; Chi² = 787.64, df = 18 (P < 0.00001); I² = 98%
Test for overall effect: Z = 1.49 (P = 0.14)

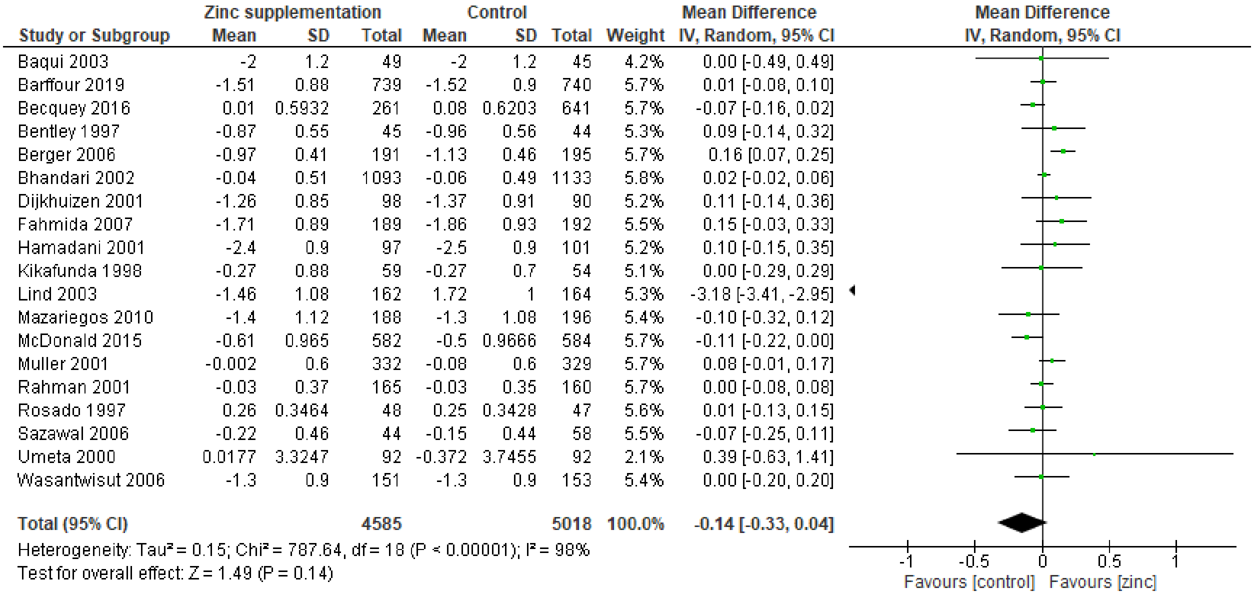

Weight-for-height (z-score)

Study or Subgroup	Zinc supplementation Mean	SD	Total	Control Mean	SD	Total	Weight	Mean Difference IV, Random, 95% CI
Baqui 2003	-0.8	0.8	49	-0.7	0.9	45	1.5%	-0.10 [-0.45, 0.25]
Barffour 2019	-0.72	0.79	739	-0.74	0.82	740	12.4%	0.02 [-0.06, 0.10]
Becquey 2016	0.27	0.7514	261	0.3	0.8374	641	9.1%	-0.03 [-0.14, 0.08]
Bentley 1997	-0.91	0.78	45	-0.84	0.88	44	1.5%	-0.07 [-0.42, 0.28]
Berger 2006	-0.96	0.51	191	-1.13	0.57	195	9.5%	0.17 [0.06, 0.28]
Bhandari 2002	-0.02	0.64	1093	-0.07	0.67	1133	16.3%	0.05 [-0.00, 0.10]
Dijkhuizen 2001	-0.36	0.8	98	-0.44	0.82	90	3.1%	0.08 [-0.15, 0.31]
Fahmida 2007	-0.88	0.97	189	-1	0.93	192	4.3%	0.12 [-0.07, 0.31]
Hamadani 2001	-1.2	0.6	97	-1.3	0.8	101	4.1%	0.10 [-0.10, 0.30]
Lind 2003	-0.7	1.06	162	-1.01	1.16	164	2.9%	0.31 [0.07, 0.55]
Mazariegos 2010	0.05	0.91	188	0.1	1.1	196	4.0%	-0.05 [-0.25, 0.15]
McDonald 2015	-0.57	1.6887	582	-0.35	1.6916	584	4.2%	-0.22 [-0.41, -0.03]
Muller 2001	-0.09	0.8	332	-0.1	0.7	329	8.9%	0.01 [-0.10, 0.12]
Rahman 2001	-0.16	0.59	165	-0.13	0.57	160	7.9%	-0.03 [-0.16, 0.10]
Rosado 1997	0.25	0.4157	48	0.29	0.4113	47	5.4%	-0.04 [-0.21, 0.13]
Silva 2006	0.7	1.5	28	0.6	1.6	30	0.3%	0.10 [-0.70, 0.90]
Umeta 2000	-0.3028	3.5423	92	-0.214	3.4029	92	0.2%	-0.09 [-1.09, 0.91]
Wasantwisut 2006	-1.2	0.6	97	-1.3	0.8	101	4.1%	0.10 [-0.10, 0.30]
**Total (95% CI)**			**4456**			**4884**	**100.0%**	**0.03 [-0.01, 0.08]**

Heterogeneity: Tau² = 0.00; Chi² = 24.80, df = 17 (P = 0.10); I² = 31%
Test for overall effect: Z = 1.40 (P = 0.16)

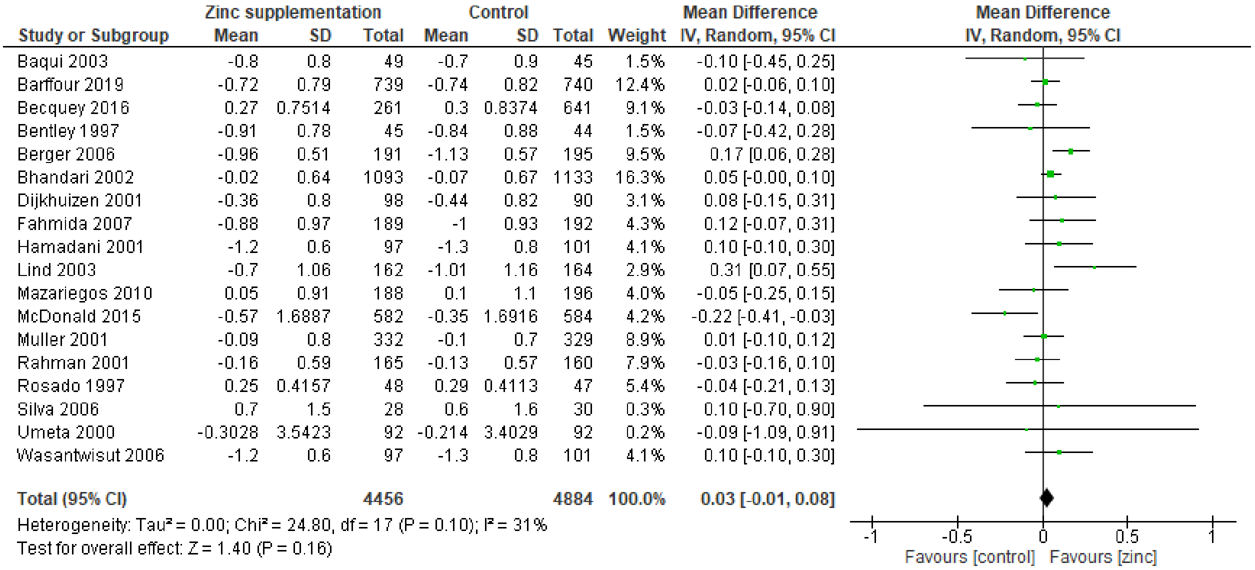

Hemoglobin (g/L)

Study or Subgroup	Zinc supplementation Mean	SD	Total	Control Mean	SD	Total	Weight	Mean Difference IV, Random, 95% CI
Baqui 2003	-3.9	12.8	64	-1.61	13.9	60	4.6%	-2.29 [-7.00, 2.42]
Barffour 2019	110.4	0.4	722	110.6	0.4	726	16.9%	-0.20 [-0.24, -0.16]
Berger 2006	6.4	20.6	191	9.8	19.3	195	5.8%	-3.40 [-7.38, 0.58]
Brooks 2005	96	9.1	329	97	9.4	309	13.5%	-1.00 [-2.44, 0.44]
Chang 2010	0.9	14	85	2	13	89	5.8%	-1.10 [-5.12, 2.92]
Dijkhuizen 2001	106	11	97	106	11	87	7.6%	0.00 [-3.18, 3.18]
Fahmida 2007	-6.4	20.1	153	-5	20.8	150	4.8%	-1.40 [-6.01, 3.21]
Lind 2003	115.7	15.2	134	113.5	16	143	6.5%	2.20 [-1.47, 5.87]
McDonald 2015	96	12.7685	159	96	13.1304	168	8.7%	0.00 [-2.81, 2.81]
Radhakrishna 2013	84	14.1	34	86.3	15.6	34	2.4%	-2.30 [-9.37, 4.77]
Rosado 1997	8	12.9	49	8	16.3	50	3.4%	0.00 [-5.78, 5.78]
Sazawal 2006	3.4	11.5	44	-7.5	11.4	58	5.0%	10.90 [6.41, 15.39]
Silva 2006	7	8	28	5	9	30	5.1%	2.00 [-2.38, 6.38]
Wasantwisut 2006	101.6	13.2	58	106.3	13	66	4.8%	-4.70 [-9.32, -0.08]
Wieringa 2003	110	10	48	110	11	43	5.2%	0.00 [-4.34, 4.34]
**Total (95% CI)**			**2195**			**2208**	**100.0%**	**-0.10 [-1.29, 1.09]**

Heterogeneity: Tau² = 2.18; Chi² = 35.00, df = 14 (P = 0.001); I² = 60%
Test for overall effect: Z = 0.16 (P = 0.87)

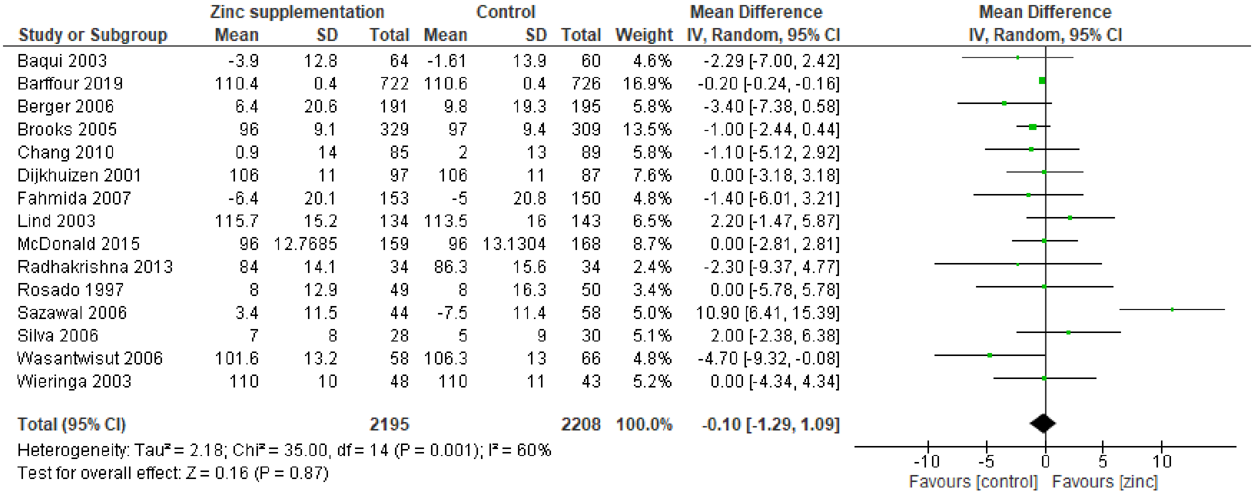

Plasma/serum ferritin (µg/L)

Study or Subgroup	Zinc supplementation Mean	SD	Total	Control Mean	SD	Total	Weight	Mean Difference IV, Random, 95% CI
Baqui 2003	-15.3	22.6	40	-21.7	22.7	37	6.9%	6.40 [-3.73, 16.53]
Berger 2006	-18.2	56.5	173	-16.9	42.6	176	6.5%	-1.30 [-11.81, 9.21]
Dijkhuizen 2001	13.07	11.26	97	15.9	12.74	87	36.4%	-2.83 [-6.32, 0.66]
Fahmida 2007	-21.7	20.64	29	-26.53	32.71	29	3.7%	4.83 [-9.25, 18.91]
McDonald 2015	51.5	47.2646	151	47.8	43.5332	151	6.8%	3.70 [-6.55, 13.95]
Rosado 1997	-2.3	15.6	48	-4.6	41.6	48	4.6%	2.30 [-10.27, 14.87]
Wasantwisut 2006	28.5	22.15	58	26.73	13.78	66	14.7%	1.77 [-4.83, 8.37]
Wieringa 2003	11.6	9.99	48	17.17	15.23	43	20.5%	-5.57 [-10.93, -0.21]
**Total (95% CI)**			**644**			**637**	**100.0%**	**-1.02 [-3.79, 1.76]**

Heterogeneity: Tau² = 2.34; Chi² = 8.17, df = 7 (P = 0.32); I² = 14%
Test for overall effect: Z = 0.72 (P = 0.47)

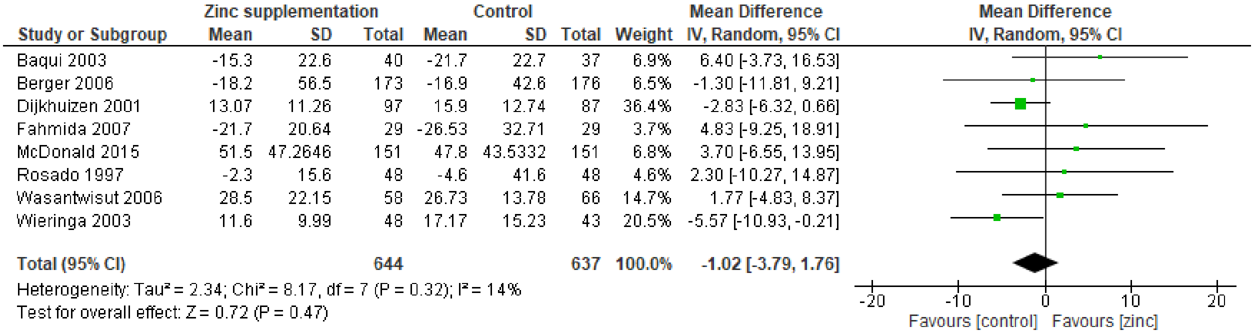

Plasma/serum TfR (mg/L)

Study or Subgroup	Zinc supplementation Mean	SD	Total	Control Mean	SD	Total	Weight	Mean Difference IV, Random, 95% CI
Baqui 2003	-0.47	2.71	42	-1.69	3.22	38	18.9%	1.22 [-0.09, 2.53]
Chang 2010	-0.4	3.3	85	-1	3.7	89	25.1%	0.60 [-0.44, 1.64]
McDonald 2015	1.8	0.6219	151	1.8	0.6219	151	56.0%	0.00 [-0.14, 0.14]
**Total (95% CI)**			**278**			**278**	**100.0%**	**0.38 [-0.31, 1.08]**

Heterogeneity: Tau² = 0.22; Chi² = 4.48, df = 2 (P = 0.11); I² = 55%
Test for overall effect: Z = 1.07 (P = 0.28)

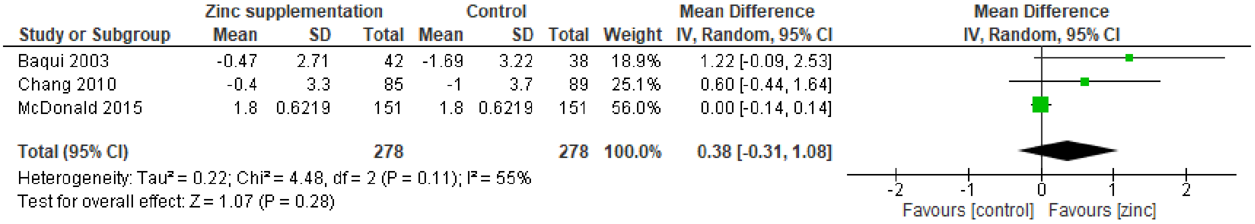

Plasma/serum retinol (μmol/L)

Study or Subgroup	Zinc supplementation Mean	SD	Total	Control Mean	SD	Total	Weight	Mean Difference IV, Random, 95% CI
Fahmida 2007	0.03	0.22	26	0.05	0.22	37	25.7%	-0.02 [-0.13, 0.09]
Rahman 2001	0.07	0.3133	84	0.06	0.3226	84	33.8%	0.01 [-0.09, 0.11]
Rosado 1997	0.08	0.4	37	-0.05	0.3	38	12.2%	0.13 [-0.03, 0.29]
Umeta 2000	0.7972	0.2633	69	0.8384	0.2954	0		Not estimable
Wieringa 2003	0.71	0.25	48	0.74	0.26	43	28.3%	-0.03 [-0.14, 0.08]
**Total (95% CI)**			**264**			**202**	**100.0%**	**0.01 [-0.05, 0.06]**

Heterogeneity: Tau² = 0.00; Chi² = 2.97, df = 3 (P = 0.40); I² = 0%
Test for overall effect: Z = 0.19 (P = 0.85)

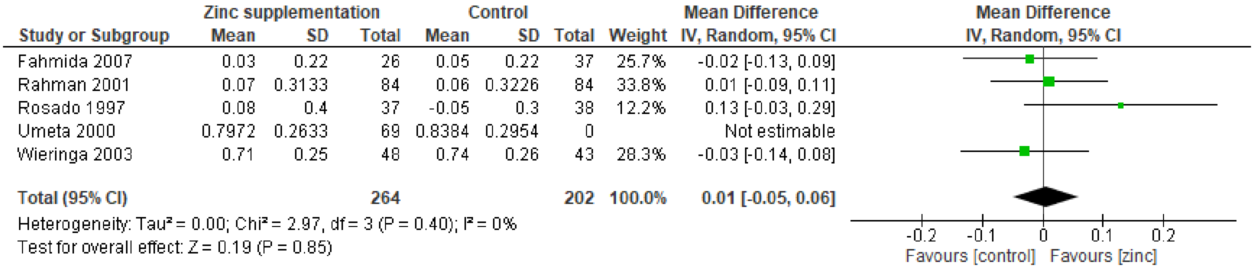

Plasma/serum zinc (μmol/L)

Study or Subgroup	Mean Difference	SE	Weight	Mean Difference IV, Random, 95% CI
Baqui 2003	0.68	0.5247	5.4%	0.68 [-0.35, 1.71]
Becquey 2016	0.78	0.2441	5.5%	0.78 [0.30, 1.26]
Berger 2006	8.7	0.7217	5.3%	8.70 [7.29, 10.11]
Bhandari 2002	10.44	0.4872	5.4%	10.44 [9.49, 11.39]
Brooks 2005	1.1	0.1668	5.6%	1.10 [0.77, 1.43]
Chang 2010	0.25	0.1592	5.6%	0.25 [-0.06, 0.56]
Dijkhuizen 2001	3.6	0.6347	5.3%	3.60 [2.36, 4.84]
Mazariegos 2010	2.5	1.4509	4.5%	2.50 [-0.34, 5.34]
Muller 2001	2.9	1.3295	4.7%	2.90 [0.29, 5.51]
Radhakrishna 2013	0.5	0.6024	5.4%	0.50 [-0.68, 1.68]
Rahman 2001	1.7	0.3927	5.5%	1.70 [0.93, 2.47]
Rosado 1997	3.45	0.8122	5.2%	3.45 [1.86, 5.04]
Silva 2006	5.31	0.1152	5.6%	5.31 [5.08, 5.54]
Surono 2014	1.23	0.8619	5.2%	1.23 [-0.46, 2.92]
Tielsch 2006	0.8	0.3674	5.5%	0.80 [0.08, 1.52]
Umeta 2000	4.1	0.7366	5.3%	4.10 [2.66, 5.54]
Wasantwisut 2006	6.9	0.7217	5.3%	6.90 [5.49, 8.31]
Wessells 2012	16.5527	1.2163	4.8%	16.55 [14.17, 18.94]
Wieringa 2003	3.07	0.9296	5.1%	3.07 [1.25, 4.89]
**Total (95% CI)**			**100.0%**	**3.85 [2.48, 5.23]**

Heterogeneity: Tau² = 8.83; Chi² = 1451.40, df = 18 (P < 0.00001); I² = 99%
Test for overall effect: Z = 5.49 (P < 0.00001)

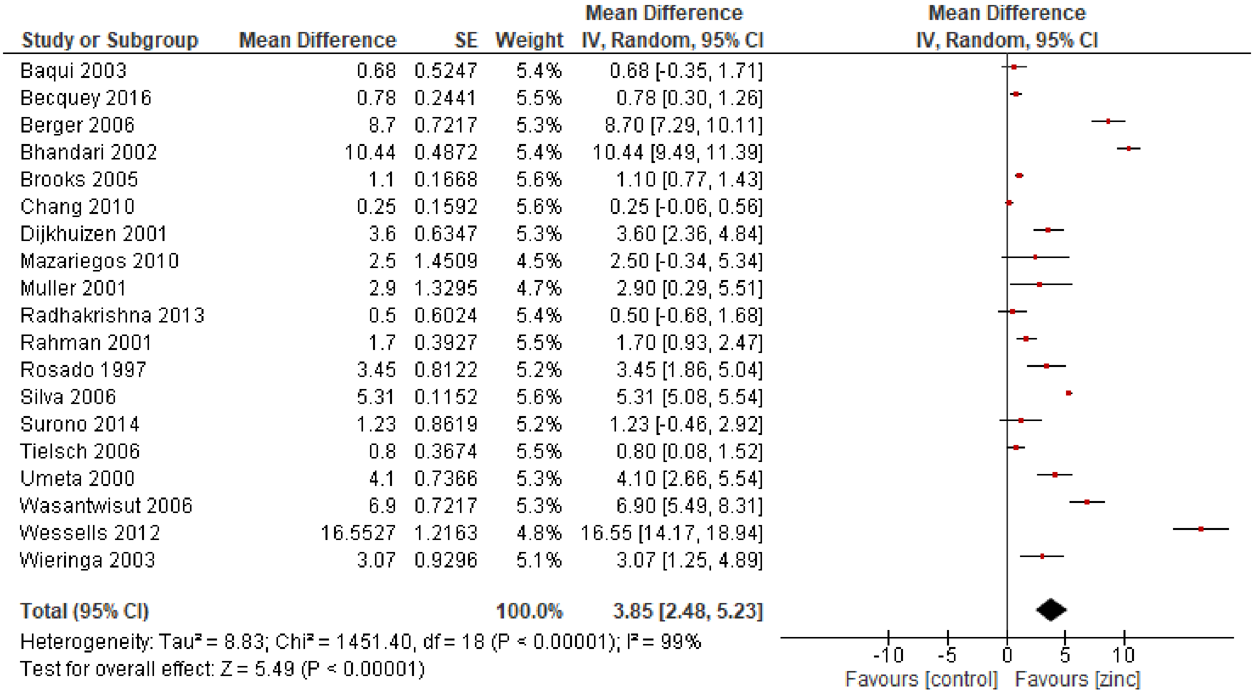

Iron deficiency (serum/plasma ferritin <12µg/L)

Study or Subgroup	Zinc supplementation Events	Zinc supplementation Total	Control Events	Control Total	Weight	Risk Ratio M-H, Random, 95% CI
Barffour 2019	16	145	40	140	15.3%	0.39 [0.23, 0.66]
Berger 2006	56	173	59	176	21.6%	0.97 [0.72, 1.30]
Lind 2003	47	134	54	143	21.2%	0.93 [0.68, 1.27]
McDonald 2015	23	151	13	151	12.7%	1.77 [0.93, 3.36]
Sazawal 2006 (1)	26	35	35	43	23.1%	0.91 [0.72, 1.16]
Wasantwisut 2006	9	58	4	66	6.1%	2.56 [0.83, 7.88]
**Total (95% CI)**		**696**		**719**	**100.0%**	**0.94 [0.69, 1.29]**
Total events	177		205			

Heterogeneity: Tau² = 0.10; Chi² = 17.54, df = 5 (P = 0.004); I² = 71%
Test for overall effect: Z = 0.37 (P = 0.71)

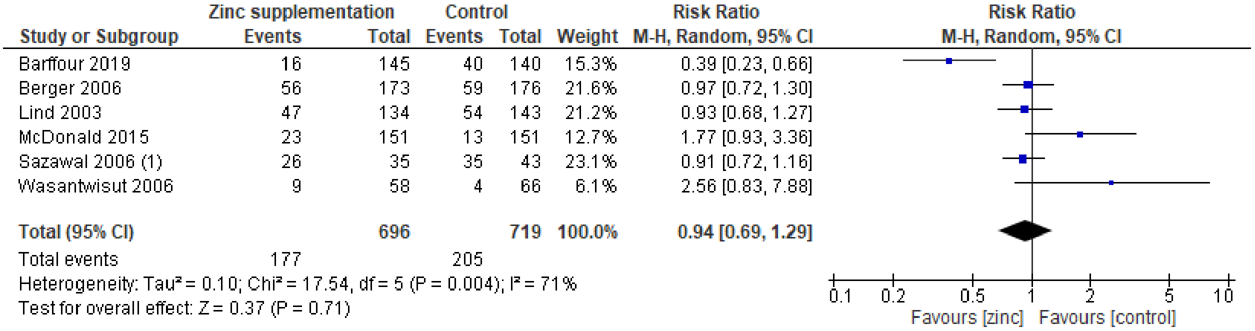

Footnotes
(1) Zinc protoporphyrin >/=90umol/mol heme

Zinc deficiency (serum/plasma zinc <10.7µmol/L)

Study or Subgroup	Zinc supplementation Events	Zinc supplementation Total	Control Events	Control Total	Weight	Risk Ratio M-H, Random, 95% CI
Barffour 2019 (1)	74	145	111	140	12.8%	0.64 [0.54, 0.77]
Berger 2006 (2)	0	161	1	155	2.2%	0.32 [0.01, 7.82]
Bhandari 2002 (3)	21	450	256	499	11.9%	0.09 [0.06, 0.14]
Dijkhuizen 2001	9	97	21	87	10.3%	0.38 [0.19, 0.79]
Lind 2003	63	134	111	143	12.7%	0.61 [0.50, 0.74]
Radhakrishna 2013 (4)	9	34	15	34	10.6%	0.60 [0.31, 1.18]
Tielsch 2006	47	124	57	119	12.4%	0.79 [0.59, 1.06]
Umeta 2000	0	38	8	40	2.6%	0.06 [0.00, 1.04]
Wasantwisut 2006	1	58	44	66	4.5%	0.03 [0.00, 0.18]
Wessells 2012 (5)	36	279	59	146	12.2%	0.32 [0.22, 0.46]
Wieringa 2003	5	48	5	43	7.8%	0.90 [0.28, 2.88]
**Total (95% CI)**		**1568**		**1472**	**100.0%**	**0.37 [0.22, 0.62]**
Total events	265		688			

Heterogeneity: Tau² = 0.53; Chi² = 141.51, df = 10 (P < 0.00001); I² = 93%
Test for overall effect: Z = 3.81 (P = 0.0001)

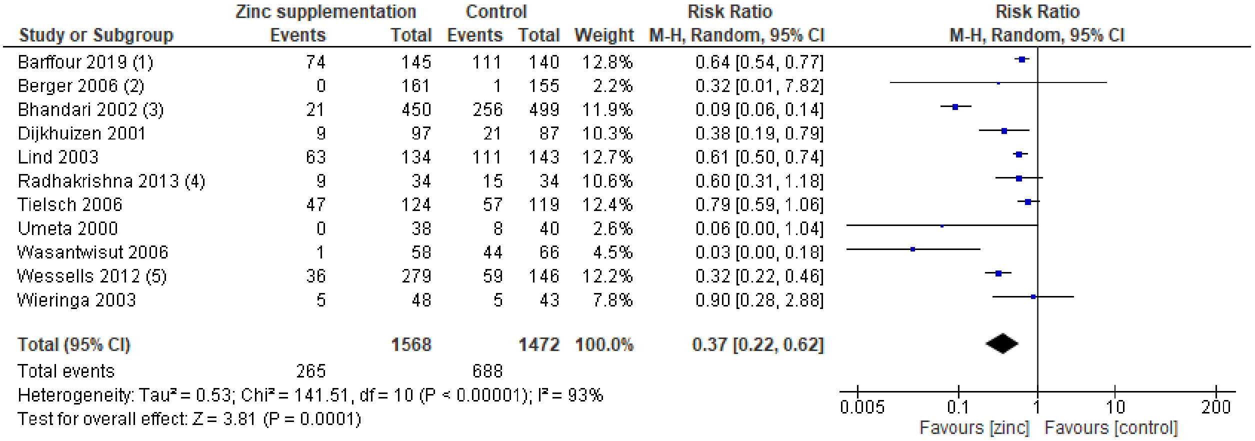

Footnotes
(1) Plasma zinc <9.94umol/L
(2) Serum zinc <9.9umol/L
(3) Plasma zinc <9.17umol/L
(4) Serum zinc <9.17umol/L
(5) Plasma zinc <9.94umol/L

Diarrhea (incidence)

Study or Subgroup	log[Rate Ratio]	SE	Weight	Rate Ratio IV, Random, 95% CI
Baqui 2003	-0.0202	0.0607	10.1%	0.98 [0.87, 1.10]
Bhandari 2002	-0.1071	0.026	12.0%	0.90 [0.85, 0.95]
Brooks 2005	-0.0619	0.0337	11.7%	0.94 [0.88, 1.00]
Chang 2010	-0.0187	0.0934	8.0%	0.98 [0.82, 1.18]
Gupta 2003	-0.8916	0.2732	2.1%	0.41 [0.24, 0.70]
Gupta 2007	-0.3011	0.0741	9.3%	0.74 [0.64, 0.86]
Hamadani 2001	0.1222	0.0939	8.0%	1.13 [0.94, 1.36]
Long 2006	0.1222	0.0727	9.4%	1.13 [0.98, 1.30]
Malik 2013	-0.4943	0.0717	9.4%	0.61 [0.53, 0.70]
McDonald 2015	-0.1863	0.0943	8.0%	0.83 [0.69, 1.00]
Tielsch 2006	-0.0408	0.0273	12.0%	0.96 [0.91, 1.01]
**Total (95% CI)**			**100.0%**	**0.89 [0.82, 0.97]**

Heterogeneity: Tau² = 0.02; Chi² = 69.40, df = 10 (P < 0.00001); I² = 86%
Test for overall effect: Z = 2.64 (P = 0.008)

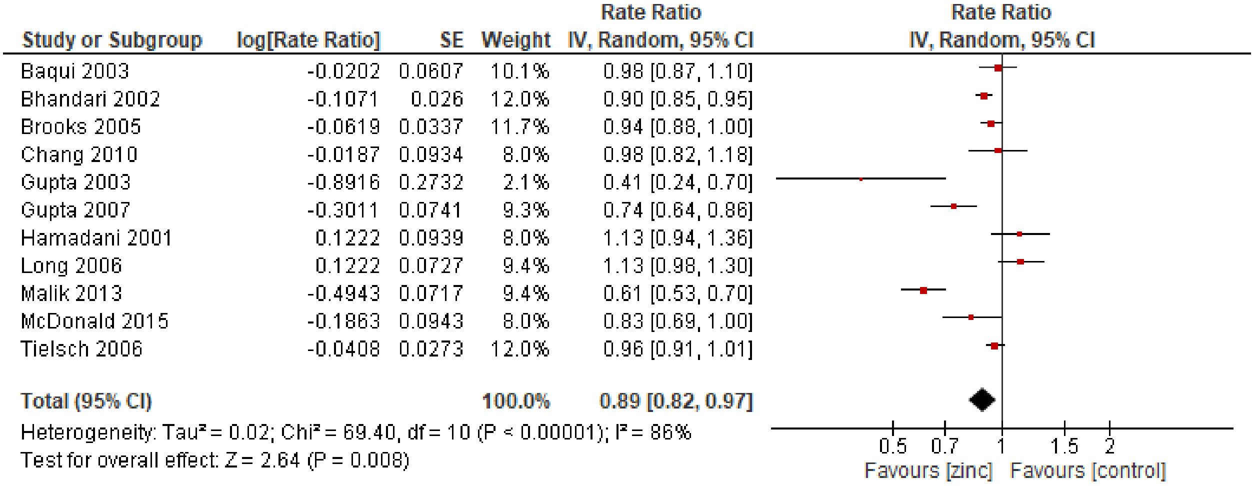

Lower respiratory tract infection (incidence)

Study or Subgroup	log[Rate Ratio]	SE	Weight	Rate Ratio IV, Random, 95% CI
Baqui 2003	-0.0513	0.1072	18.6%	0.95 [0.77, 1.17]
Bhandari 2002	-0.0041	0.0583	19.1%	1.00 [0.89, 1.12]
Hamadani 2001	-0.0101	0.1696	17.6%	0.99 [0.71, 1.38]
Long 2006	-0.2231	0.7611	6.5%	0.80 [0.18, 3.56]
Malik 2013	-1.204	0.073	19.0%	0.30 [0.26, 0.35]
Tielsch 2006	0.01	0.0476	19.2%	1.01 [0.92, 1.11]
**Total (95% CI)**			**100.0%**	**0.78 [0.49, 1.24]**

Heterogeneity: Tau² = 0.30; Chi² = 222.76, df = 5 (P < 0.00001); I² = 98%
Test for overall effect: Z = 1.06 (P = 0.29)

Rate Ratio
IV, Random, 95% CI

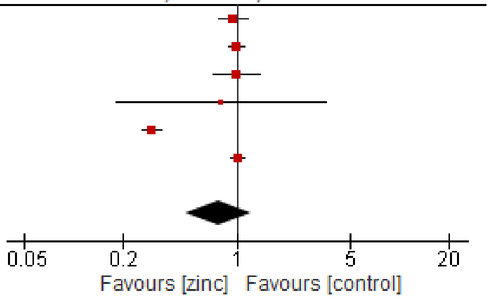

Mental development

Study or Subgroup	Std. Mean Difference	SE	Weight	Std. Mean Difference IV, Random, 95% CI
Baqui 2003 (1)	2	2.3358	1.1%	2.00 [-2.58, 6.58]
Bhandari 2002 (2)	-0.7	0.7143	9.3%	-0.70 [-2.10, 0.70]
Hamadani 2001 (3)	-3.3	1.4504	2.7%	-3.30 [-6.14, -0.46]
Lind 2003 (4)	2	1.0694	4.7%	2.00 [-0.10, 4.10]
Tielsch 2006 (5)	0.281	0.1253	41.0%	0.28 [0.04, 0.53]
Wasantwisut 2006 (6)	-0.0498	0.12	41.4%	-0.05 [-0.28, 0.19]
**Total (95% CI)**			**100.0%**	**0.06 [-0.42, 0.53]**

Heterogeneity: Tau² = 0.13; Chi² = 14.21, df = 5 (P = 0.01); I² = 65%
Test for overall effect: Z = 0.23 (P = 0.82)

Std. Mean Difference
IV, Random, 95% CI

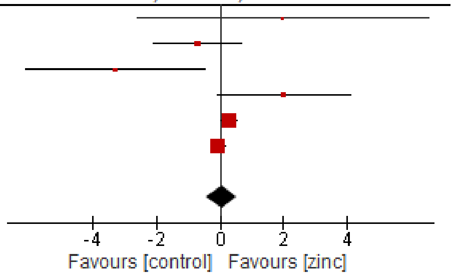

Footnotes
(1) Mental Development Index (Bayley Scales of Infant Development)
(2) Mental Development Index (Bayley Scales of Infant Development)
(3) Mental Development Index (Bayley Scales of Infant Development)
(4) Mental Development Index (Bayley Scales of Infant Development)
(5) Universal Test of Nonverbal Intelligence
(6) Full IQ (Wechsler Intelligence Scale for Children 3rd edition)

Mental development (Mental Development Index of Bayley Scales of Infant Development)

Study or Subgroup	Mean Difference	SE	Weight	Mean Difference IV, Random, 95% CI
Baqui 2003	2	2.3358	14.7%	2.00 [-2.58, 6.58]
Bhandari 2002	-0.7	0.7143	33.2%	-0.70 [-2.10, 0.70]
Hamadani 2001	-3.3	1.4504	23.6%	-3.30 [-6.14, -0.46]
Lind 2003	2	1.0694	28.6%	2.00 [-0.10, 4.10]
**Total (95% CI)**			**100.0%**	**-0.15 [-2.38, 2.09]**

Heterogeneity: Tau² = 3.42; Chi² = 10.17, df = 3 (P = 0.02); I² = 71%
Test for overall effect: Z = 0.13 (P = 0.90)

Mean Difference
IV, Random, 95% CI

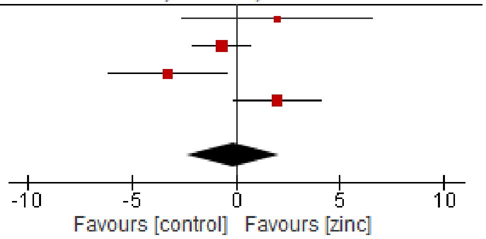

Motor development

Study or Subgroup	Std. Mean Difference	SE	Weight	Std. Mean Difference IV, Random, 95% CI
Baqui 2003 (1)	5.8	3.3953	5.6%	5.80 [-0.85, 12.45]
Bhandari 2002 (2)	1.7	0.9712	25.9%	1.70 [-0.20, 3.60]
Hamadani 2001 (3)	-2.6	2.6869	8.3%	-2.60 [-7.87, 2.67]
Lind 2003 (4)	2	1.1834	22.4%	2.00 [-0.32, 4.32]
Tielsch 2006 (5)	-0.312	0.1254	37.8%	-0.31 [-0.56, -0.07]
**Total (95% CI)**			**100.0%**	**0.88 [-0.83, 2.59]**

Heterogeneity: Tau² = 2.00; Chi² = 11.83, df = 4 (P = 0.02); I² = 66%
Test for overall effect: Z = 1.01 (P = 0.31)

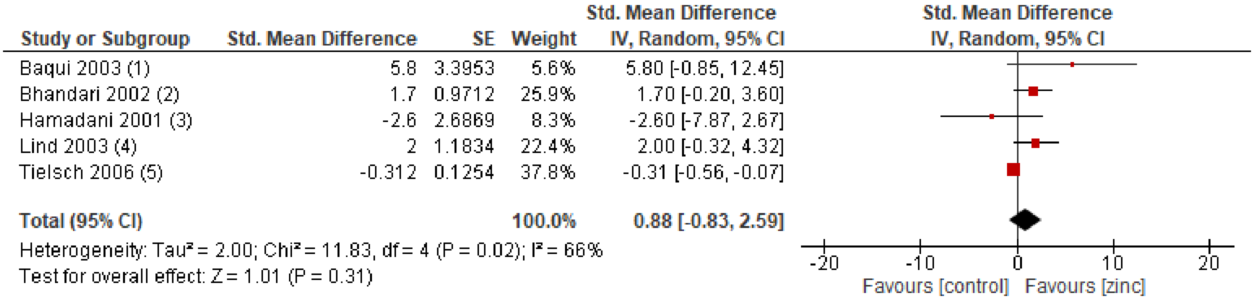

Footnotes
(1) Psychomotor Development Index (Bayley Scales of Infant Development)
(2) Psychomotor Development Index (Bayley Scales of Infant Development)
(3) Psychomotor Development Index (Bayley Scales of Infant Development)
(4) Psychomotor Development Index (Bayley Scales of Infant Development)
(5) Movement Assessment Battery for Children

Motor development (Psychomotor Development Index of Bayley Scales of Infant Development)

Study or Subgroup	Mean Difference	SE	Weight	Mean Difference IV, Random, 95% CI
Baqui 2003	5.8	3.3953	6.9%	5.80 [-0.85, 12.45]
Bhandari 2002	1.7	0.9712	45.8%	1.70 [-0.20, 3.60]
Hamadani 2001	-2.6	2.6869	10.5%	-2.60 [-7.87, 2.67]
Lind 2003	2	1.1834	36.8%	2.00 [-0.32, 4.32]
**Total (95% CI)**			**100.0%**	**1.64 [-0.17, 3.45]**

Heterogeneity: Tau² = 0.93; Chi² = 4.08, df = 3 (P = 0.25); I² = 27%
Test for overall effect: Z = 1.77 (P = 0.08)

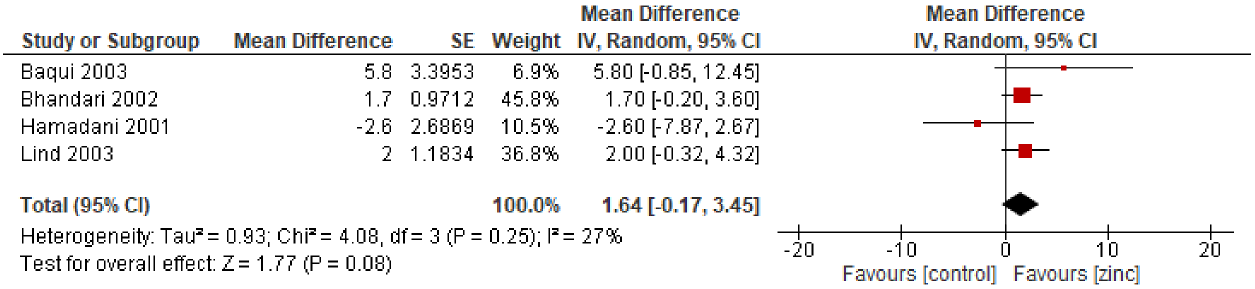

Comparison 3: Iron Supplementation vs. Placebo/No Intervention (Efficacy)

Stunting

Study or Subgroup	Iron supplementation Events	Total	Control Events	Total	Weight	Risk Ratio M-H, Random, 95% CI
Berger 2006	52	197	50	195	40.8%	1.03 [0.74, 1.44]
Dijkhuizen 2001	18	94	22	90	15.0%	0.78 [0.45, 1.36]
Lind 2003	12	163	14	164	8.3%	0.86 [0.41, 1.81]
Stoltzfus 2001	39	116	41	119	35.9%	0.98 [0.68, 1.39]
**Total (95% CI)**		**570**		**568**	**100.0%**	**0.96 [0.77, 1.18]**
Total events	121		127			

Heterogeneity: Tau² = 0.00; Chi² = 0.78, df = 3 (P = 0.85); I² = 0%
Test for overall effect: Z = 0.42 (P = 0.67)

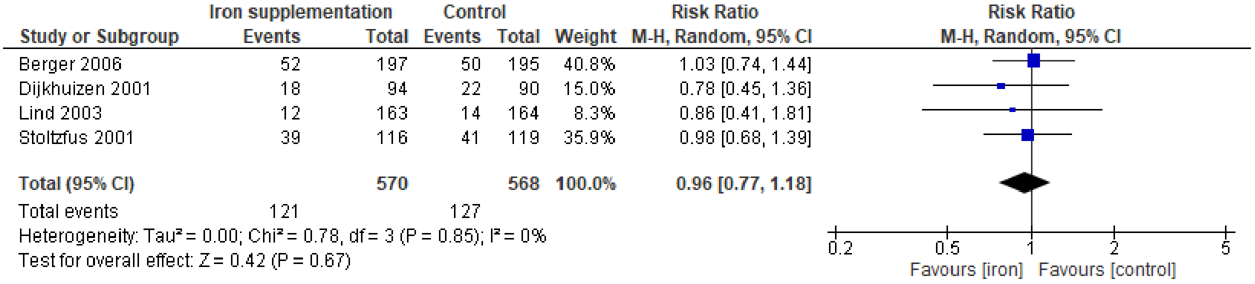

Wasting

Study or Subgroup	Iron supplementation Events	Total	Control Events	Total	Weight	Risk Ratio M-H, Random, 95% CI
Berger 2006	11	197	8	195	19.5%	1.36 [0.56, 3.31]
Dijkhuizen 2001	0	94	0	90		Not estimable
Lind 2003	35	163	30	164	80.5%	1.17 [0.76, 1.82]
**Total (95% CI)**		**454**		**449**	**100.0%**	**1.21 [0.82, 1.79]**
Total events	46		38			

Heterogeneity: Tau² = 0.00; Chi² = 0.09, df = 1 (P = 0.77); I² = 0%
Test for overall effect: Z = 0.95 (P = 0.34)

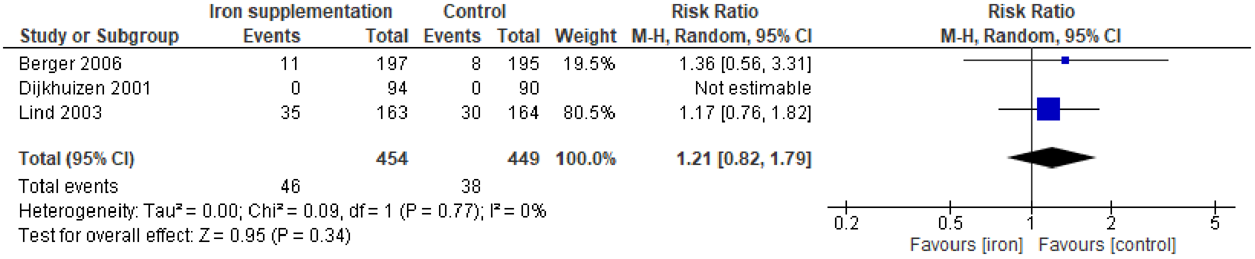

Height (cm)

Study or Subgroup	Iron supplementation Mean	SD	Total	Control Mean	SD	Total	Weight	Mean Difference IV, Random, 95% CI
Baqui 2003	70.9	2.7	150	71	2.6	140	6.1%	-0.10 [-0.71, 0.51]
Berger 2006	70.99	2.51	197	71.01	2.41	195	8.8%	-0.02 [-0.51, 0.47]
Dijkhuizen 2001	69.5	2.5	94	69.1	2.7	90	4.2%	0.40 [-0.35, 1.15]
Domellof 2001	6.52	1.1384	40	6.83	1.1665	42	8.5%	-0.31 [-0.81, 0.19]
Dossa 2001	1.9	0.6	36	2.1	0.9	32	13.1%	-0.20 [-0.57, 0.17]
Lind 2003	72.5	3	136	72.4	2.8	143	5.0%	0.10 [-0.58, 0.78]
Lopez 2005	6.67	1.7	74	7.08	1.3	72	8.7%	-0.41 [-0.90, 0.08]
Lozoff 2016	6.67	1.7	74	7.08	1.3	72	8.7%	-0.41 [-0.90, 0.08]
Nagpal 2004	3.08	1.52	19	3.26	1.54	24	2.9%	-0.18 [-1.10, 0.74]
Palupi 1997	1.4	1	96	1.2	1	98	18.1%	0.20 [-0.08, 0.48]
Rosado 1997	9	1.3435	50	9.1	1.5082	47	6.8%	-0.10 [-0.67, 0.47]
Smuts 2005	77.9	3	49	76.6	3.3	50	1.7%	1.30 [0.06, 2.54]
Wasantwisut 2006	70.6	2.5	153	70.6	2.4	153	7.3%	0.00 [-0.55, 0.55]
**Total (95% CI)**			**1168**			**1158**	**100.0%**	**-0.06 [-0.23, 0.10]**

Heterogeneity: Tau² = 0.02; Chi² = 15.13, df = 12 (P = 0.23); I² = 21%
Test for overall effect: Z = 0.76 (P = 0.45)

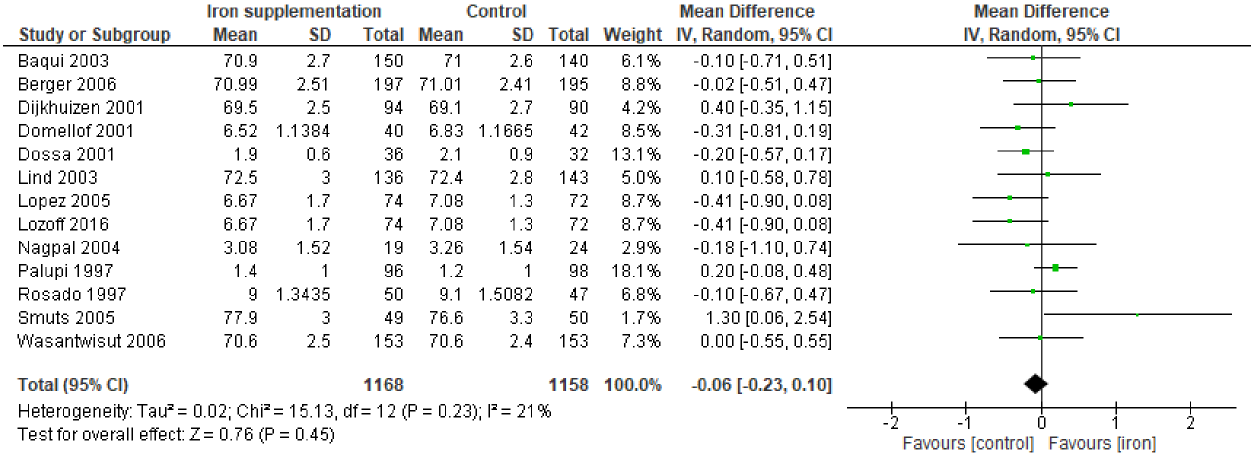

Weight (kg)

Study or Subgroup	Iron supplementation Mean	SD	Total	Control Mean	SD	Total	Weight	Mean Difference IV, Random, 95% CI
Baqui 2003	8.09	1.09	150	8.16	1.01	140	6.4%	-0.07 [-0.31, 0.17]
Berger 2006	7.98	0.9	197	8.05	0.8	195	11.7%	-0.07 [-0.24, 0.10]
Dijkhuizen 2001	8.1	1	94	7.8	1	90	4.7%	0.30 [0.01, 0.59]
Domellof 2001	1.548	0.5439	40	1.515	0.5444	42	6.7%	0.03 [-0.20, 0.27]
Dossa 2001	0.3	0.4	36	0.5	0.6	32	6.3%	-0.20 [-0.45, 0.05]
Ermis 2002	9	1	28	9.1	1	23	1.4%	-0.10 [-0.65, 0.45]
Lind 2003	8.33	1.02	136	8.31	0.94	143	7.0%	0.02 [-0.21, 0.25]
Lopez 2005	1.24	0.6	74	1.32	0.6	72	9.3%	-0.08 [-0.27, 0.11]
Lozoff 2016	6.161	1.111	321	6.27	1.1132	312	11.2%	-0.11 [-0.28, 0.06]
Nagpal 2004	0.778	0.532	19	0.703	0.341	24	5.1%	0.08 [-0.20, 0.35]
Palupi 1997	0.45	0.58	96	0.34	0.63	98	11.5%	0.11 [-0.06, 0.28]
Rosado 1997	2.4	0.495	50	2.4	0.4799	47	9.3%	0.00 [-0.19, 0.19]
Smuts 2005	11	1.3	49	10.6	1.3	50	1.6%	0.40 [-0.11, 0.91]
Wasantwisut 2006	8.2	0.9	153	8.1	1	153	8.0%	0.10 [-0.11, 0.31]
**Total (95% CI)**			**1443**			**1421**	**100.0%**	**0.00 [-0.06, 0.07]**

Heterogeneity: Tau² = 0.00; Chi² = 15.15, df = 13 (P = 0.30); I² = 14%
Test for overall effect: Z = 0.07 (P = 0.95)

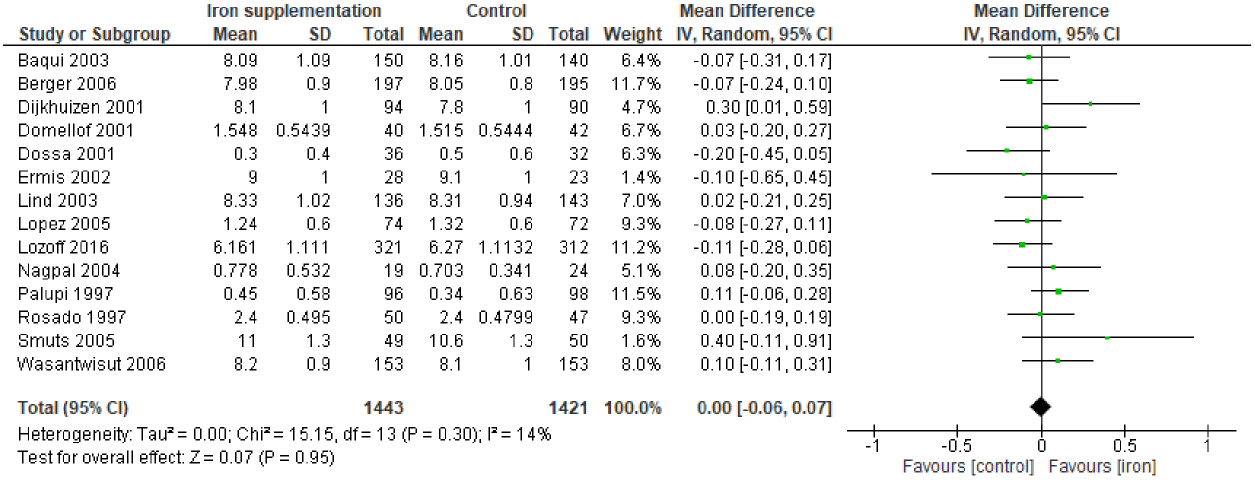

Length-for-age (z-score)

Study or Subgroup	Iron supplementation Mean	SD	Total	Control Mean	SD	Total	Weight	Mean Difference IV, Random, 95% CI
Baqui 2003	-1.6	0.9	49	-1.7	1	45	1.4%	0.10 [-0.29, 0.49]
Berger 2006	-0.38	0.39	197	-0.42	0.46	195	16.4%	0.04 [-0.04, 0.12]
Dijkhuizen 2001	-1.24	0.87	94	-1.3	0.88	90	3.1%	0.06 [-0.19, 0.31]
Domellof 2001	-1.2	1.02	40	-1.28	0.99	42	1.1%	0.08 [-0.36, 0.52]
Dossa 2001	-0.02	0.14	36	0.02	0.22	32	15.5%	-0.04 [-0.13, 0.05]
Le 2005	-0.43	0.433	75	-0.49	0.4272	73	8.6%	0.06 [-0.08, 0.20]
Lind 2003	-0.66	0.91	163	-0.81	0.86	164	5.0%	0.15 [-0.04, 0.34]
Lopez 2005	-0.27	0.5	74	-0.09	0.5	72	6.7%	-0.18 [-0.34, -0.02]
Lozoff 2016	0.24	1.0017	321	0.33	1.0773	312	6.7%	-0.09 [-0.25, 0.07]
Palupi 1997	0.07	0.27	96	0.03	0.26	98	18.6%	0.04 [-0.03, 0.11]
Rosado 1997	0.02	0.4243	50	0.13	0.4113	47	6.4%	-0.11 [-0.28, 0.06]
Smuts 2005	-0.34	0.63	49	-0.3	0.65	50	3.1%	-0.04 [-0.29, 0.21]
Untoro 2005	-1.51	0.87	69	-1.77	0.96	65	2.1%	0.26 [-0.05, 0.57]
Wasantwisut 2006	-1	0.8	153	-0.99	0.86	153	5.3%	-0.01 [-0.20, 0.18]
**Total (95% CI)**			**1466**			**1438**	**100.0%**	**0.00 [-0.04, 0.05]**

Heterogeneity: Tau² = 0.00; Chi² = 16.70, df = 13 (P = 0.21); I² = 22%
Test for overall effect: Z = 0.14 (P = 0.89)

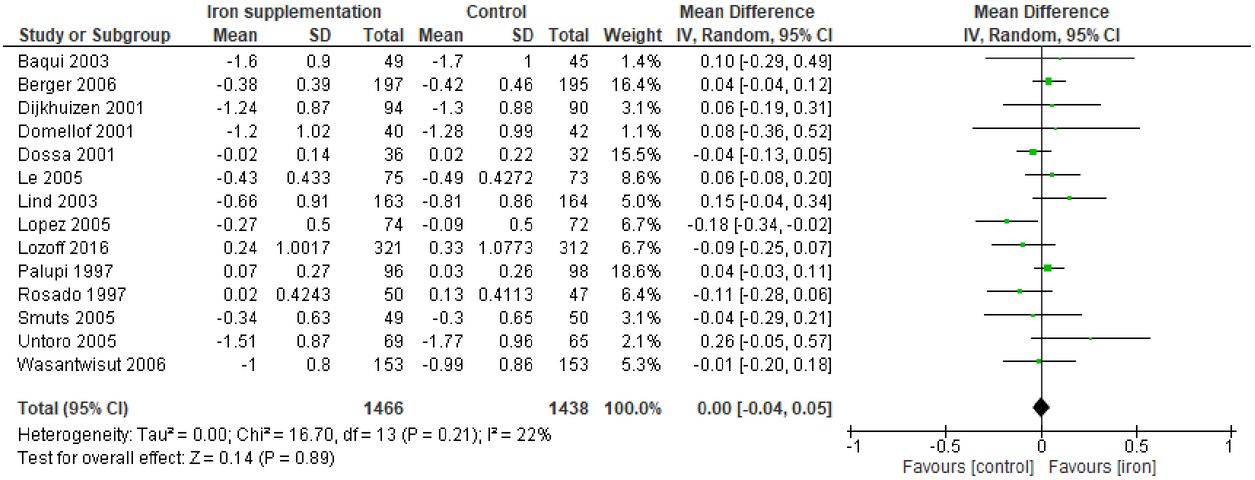

Weight-for-age (z-score)

Study or Subgroup	Iron supplementation Mean	SD	Total	Control Mean	SD	Total	Weight	Mean Difference IV, Random, 95% CI
Baqui 2003	-2.2	1	49	-2	1.2	45	1.1%	-0.20 [-0.65, 0.25]
Berger 2006	-1.13	0.4	197	-1.13	0.46	195	29.1%	0.00 [-0.09, 0.09]
Dijkhuizen 2001	-1.19	0.95	94	-1.37	0.91	90	2.9%	0.18 [-0.09, 0.45]
Domellof 2001	-0.79	1.1	40	-0.9	0.86	42	1.2%	0.11 [-0.32, 0.54]
Le 2005	-0.64	0.433	75	-0.66	0.4272	73	11.0%	0.02 [-0.12, 0.16]
Lind 2003	-1.65	1.08	163	-1.72	1	164	4.2%	0.07 [-0.16, 0.30]
Lopez 2005	-0.49	0.6	74	-0.33	0.6	72	5.6%	-0.16 [-0.35, 0.03]
Lozoff 2016	0.8	1.0017	321	0.89	1.0773	312	8.1%	-0.09 [-0.25, 0.07]
Palupi 1997	0.14	0.36	96	0.06	0.41	98	18.0%	0.08 [-0.03, 0.19]
Rosado 1997	0.28	0.4243	50	0.25	0.3428	47	9.0%	0.03 [-0.12, 0.18]
Smuts 2005	-0.61	0.66	49	-0.5	0.91	50	2.2%	-0.11 [-0.42, 0.20]
Untoro 2005	-1.49	0.8	69	-1.54	0.93	65	2.4%	0.05 [-0.24, 0.34]
Wasantwisut 2006	-1.2	0.9	153	-1.3	0.9	153	5.2%	0.10 [-0.10, 0.30]
**Total (95% CI)**			**1430**			**1406**	**100.0%**	**0.01 [-0.03, 0.06]**

Heterogeneity: Tau² = 0.00; Chi² = 10.35, df = 12 (P = 0.59); I² = 0%
Test for overall effect: Z = 0.62 (P = 0.54)

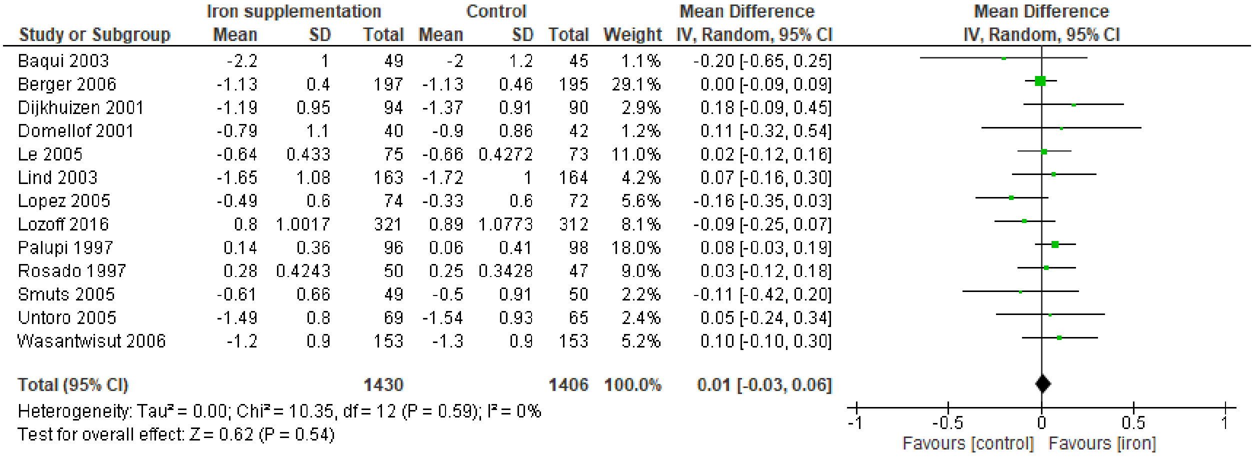

Weight-for-height (z-score)

Study or Subgroup	Iron supplementation Mean	SD	Total	Control Mean	SD	Total	Weight	Mean Difference IV, Random, 95% CI
Baqui 2003	-0.9	0.9	49	-0.7	0.9	45	2.6%	-0.20 [-0.56, 0.16]
Berger 2006	-1.18	0.51	197	-1.13	0.57	195	17.5%	-0.05 [-0.16, 0.06]
Dijkhuizen 2001	-0.3	0.81	94	-0.44	0.82	90	5.6%	0.14 [-0.10, 0.38]
Dossa 2001	-0.05	0.37	36	0.06	0.51	32	6.6%	-0.11 [-0.32, 0.10]
Le 2005	-0.63	0.5196	75	-0.63	0.5126	73	9.8%	0.00 [-0.17, 0.17]
Lind 2003	-1.07	1.23	163	-1.01	1.16	164	4.7%	-0.06 [-0.32, 0.20]
Lopez 2005	-0.62	0.8	74	-0.53	0.8	72	4.7%	-0.09 [-0.35, 0.17]
Lozoff 2016	0.96	1.0928	321	1.02	1.0773	312	9.6%	-0.06 [-0.23, 0.11]
Palupi 1997	0.12	0.49	96	0.03	0.56	98	11.6%	0.09 [-0.06, 0.24]
Rosado 1997	0.36	0.4243	50	0.29	0.4113	47	9.8%	0.07 [-0.10, 0.24]
Smuts 2005	-0.62	0.88	49	-0.55	1.05	50	2.3%	-0.07 [-0.45, 0.31]
Untoro 2005	-0.72	0.79	69	-0.57	0.83	65	4.3%	-0.15 [-0.42, 0.12]
Wasantwisut 2006	-0.5	0.7	153	-0.7	0.7	153	10.7%	0.20 [0.04, 0.36]
**Total (95% CI)**			**1426**			**1396**	**100.0%**	**0.00 [-0.06, 0.06]**

Heterogeneity: Tau² = 0.00; Chi² = 15.09, df = 12 (P = 0.24); I² = 20%
Test for overall effect: Z = 0.15 (P = 0.88)

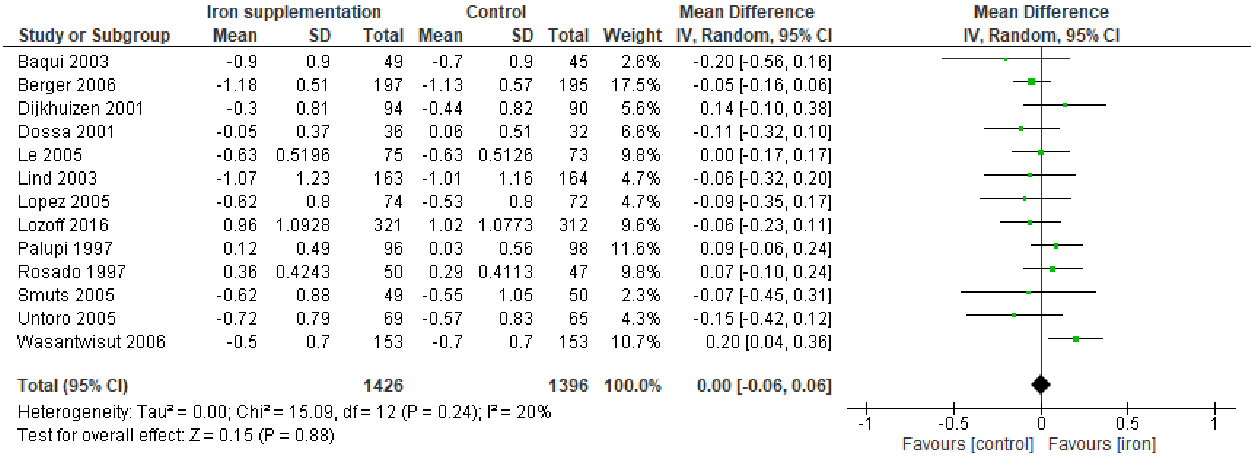

Mid-upper arm circumference (cm)

Study or Subgroup	Iron supplementation Mean	SD	Total	Control Mean	SD	Total	Weight	Mean Difference IV, Random, 95% CI
Baqui 2003	13.71	1.1	150	13.67	1.03	140	27.0%	0.04 [-0.21, 0.29]
Dossa 2001	-0.2	0.6	36	0	0.8	32	14.1%	-0.20 [-0.54, 0.14]
Lind 2003	14.7	1.12	163	14.7	1.18	164	26.1%	0.00 [-0.25, 0.25]
Rosado 1997	0.73	0.5426	46	0.67	0.5426	46	32.9%	0.06 [-0.16, 0.28]
**Total (95% CI)**			**395**			**382**	**100.0%**	**0.00 [-0.12, 0.13]**

Heterogeneity: Tau² = 0.00; Chi² = 1.72, df = 3 (P = 0.63); I² = 0%
Test for overall effect: Z = 0.04 (P = 0.97)

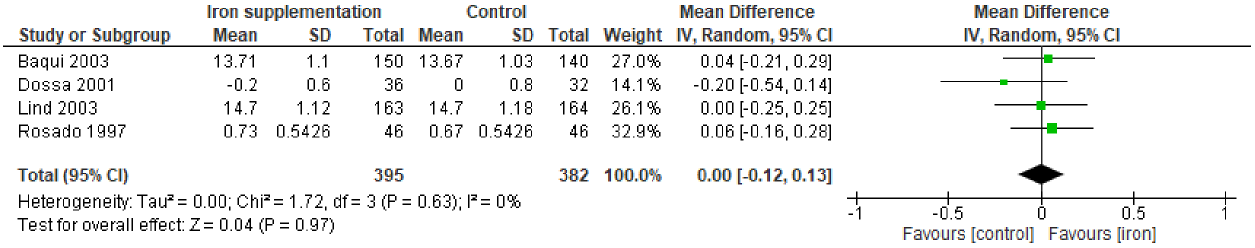

Hemoglobin (g/L)

Study or Subgroup	Iron supplementation Mean	SD	Total	Control Mean	SD	Total	Weight	Mean Difference IV, Random, 95% CI
Baqui 2003	1.6	12.8	68	-1.61	13.9	60	3.8%	3.21 [-1.44, 7.86]
Berger 2000	105.4	12	68	105.3	13	60	3.9%	0.10 [-4.25, 4.45]
Berger 2006	22.6	19.4	197	9.8	19.3	195	4.1%	12.80 [8.97, 16.63]
Chen 2013	125.1	12.7	98	114.9	13.7	104	4.2%	10.20 [6.56, 13.84]
Dijkhuizen 2001	115	10	90	106	11	87	4.4%	9.00 [5.90, 12.10]
Domellof 2001	115.9	10.9	30	108.7	8.8	35	3.7%	7.20 [2.33, 12.07]
Dossa 2001	7	14	34	4	10	32	3.2%	3.00 [-2.84, 8.84]
Ermis 2002	11.6491	0.3018	55	11.3	0.4	23	5.1%	0.35 [0.17, 0.53]
Gokcay 2012	114	9	51	112	8	54	4.3%	2.00 [-1.26, 5.26]
Le 2005	12.9	16.6458	55	8.6	13.4428	56	3.3%	4.30 [-1.33, 9.93]
Lind 2003	119.4	15.3	136	113.5	16	143	4.2%	5.90 [2.23, 9.57]
Lopez 2005	16	4	74	4	3	72	5.0%	12.00 [10.86, 13.14]
Lozoff 2016	113	8.9626	311	111	8.875	305	5.0%	2.00 [0.59, 3.41]
Monteiro 2001	10	13.6938	293	4.5	18.6011	346	4.6%	5.50 [2.99, 8.01]
Nagpal 2004	3	13	19	-3	4	24	3.2%	6.00 [-0.06, 12.06]
Nguyen 2002	21.6	12.3	37	8	11.1	36	3.4%	13.60 [8.23, 18.97]
Northrop-Clewes 1996	8.67	13.33	95	-1.93	10.22	96	4.3%	10.60 [7.23, 13.97]
Palupi 1997	6.4	10.6	96	1.9	8	98	4.6%	4.50 [1.85, 7.15]
Rosado 1997	14	14	52	8	16.3	50	3.2%	6.00 [0.09, 11.91]
Smuts 2005	2.47	1.49	49	-0.56	1.81	50	5.1%	3.03 [2.38, 3.68]
Untoro 2005	5.6	2.6	58	1.5	1.8	65	5.1%	4.10 [3.30, 4.90]
Wasantwisut 2006	118	12.2	65	106.3	13	66	3.9%	11.70 [7.38, 16.02]
Wieringa 2003	118	10	49	110	11	43	3.9%	8.00 [3.68, 12.32]
Yurdakök 2004	114	5	18	112	3	16	4.5%	2.00 [-0.74, 4.74]
**Total (95% CI)**			**2098**			**2116**	**100.0%**	**6.02 [4.28, 7.76]**

Heterogeneity: $Tau^2 = 15.38$; $Chi^2 = 710.67$, df = 23 ($P < 0.00001$); $I^2 = 97\%$
Test for overall effect: $Z = 6.78$ ($P < 0.00001$)

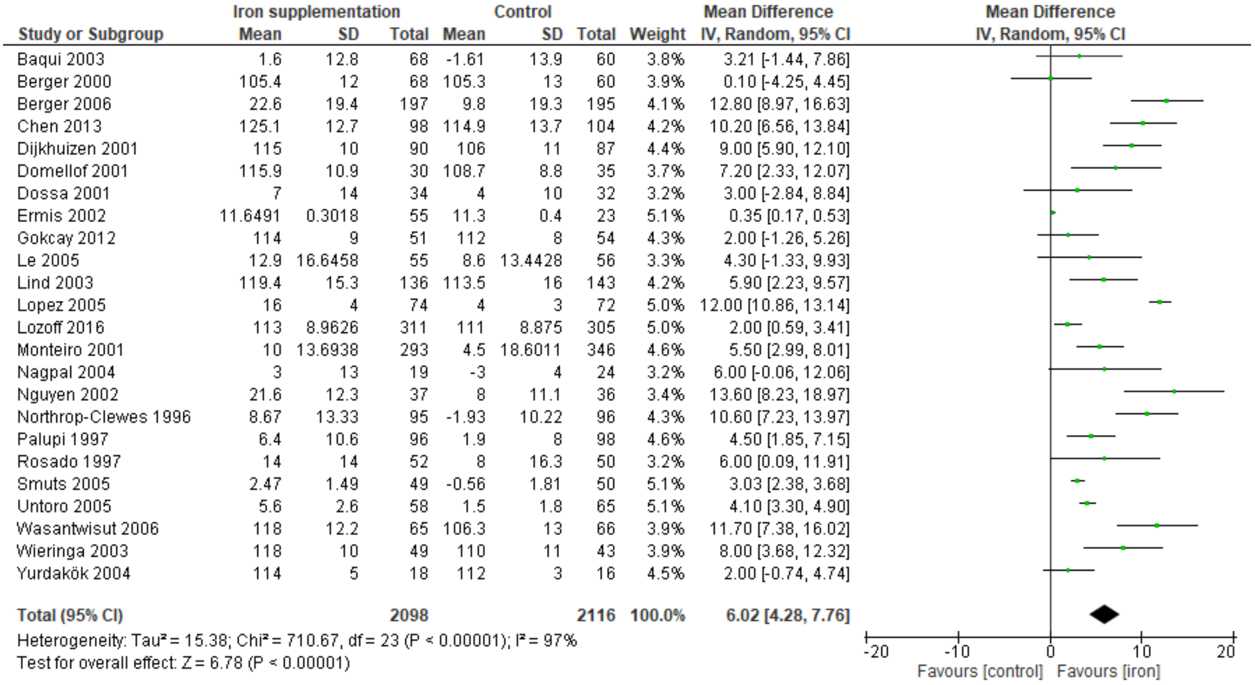

Plasma/serum ferritin (μg/L)

Study or Subgroup	Iron supplementation Mean	SD	Total	Control Mean	SD	Total	Weight	Mean Difference IV, Random, 95% CI
Baqui 2003	-18	26.8	38	-21.7	27.7	37	6.1%	3.70 [-8.64, 16.04]
Berger 2000	126.2	52.6	84	75.4	59.9	79	5.2%	50.80 [33.45, 68.15]
Berger 2006	36	47.1	195	-16.9	42.6	176	6.7%	52.90 [43.77, 62.03]
Chen 2013	41.3	12.4	98	33.1	10.9	104	7.4%	8.20 [4.97, 11.43]
Dijkhuizen 2001	39.03	31.39	90	15.9	12.74	87	7.0%	23.13 [16.11, 30.15]
Ermis 2002	45.3236	10.4401	55	17.2	4.6	23	7.4%	28.12 [24.78, 31.46]
Gokcay 2012	31.2	24.9	37	20.6	16.8	43	6.6%	10.60 [1.14, 20.06]
Lopez 2005	22.71	22.99	74	10.33	7.35	72	7.2%	12.38 [6.87, 17.89]
Nagpal 2004	0.33	3.71	19	-1.89	3.47	24	7.5%	2.22 [0.05, 4.39]
Northrop-Clewes 1996	6.07	14	95	-2.67	10.27	96	7.4%	8.74 [5.25, 12.23]
Rosado 1997	16	38.7	49	-4.6	41.6	48	5.4%	20.60 [4.60, 36.60]
Smuts 2005	46.53	62.96	38	22.07	20.2	35	4.5%	24.46 [3.35, 45.57]
Untoro 2005	35.68	75.63	65	38	69.88	60	3.8%	-2.32 [-27.83, 23.19]
Wasantwisut 2006	77.37	50.59	65	26.8	13.78	66	6.1%	50.57 [37.83, 63.31]
Wieringa 2003	41.43	38.22	49	17.17	15.23	43	6.3%	24.26 [12.63, 35.89]
Yurdakök 2004	55.7	26.4	18	43.9	23.7	16	5.3%	11.80 [-5.04, 28.64]
**Total (95% CI)**			**1069**			**1009**	**100.0%**	**20.48 [13.41, 27.55]**

Heterogeneity: $Tau^2 = 172.43$; $Chi^2 = 327.20$, df = 15 ($P < 0.00001$); $I^2 = 95\%$
Test for overall effect: $Z = 5.68$ ($P < 0.00001$)

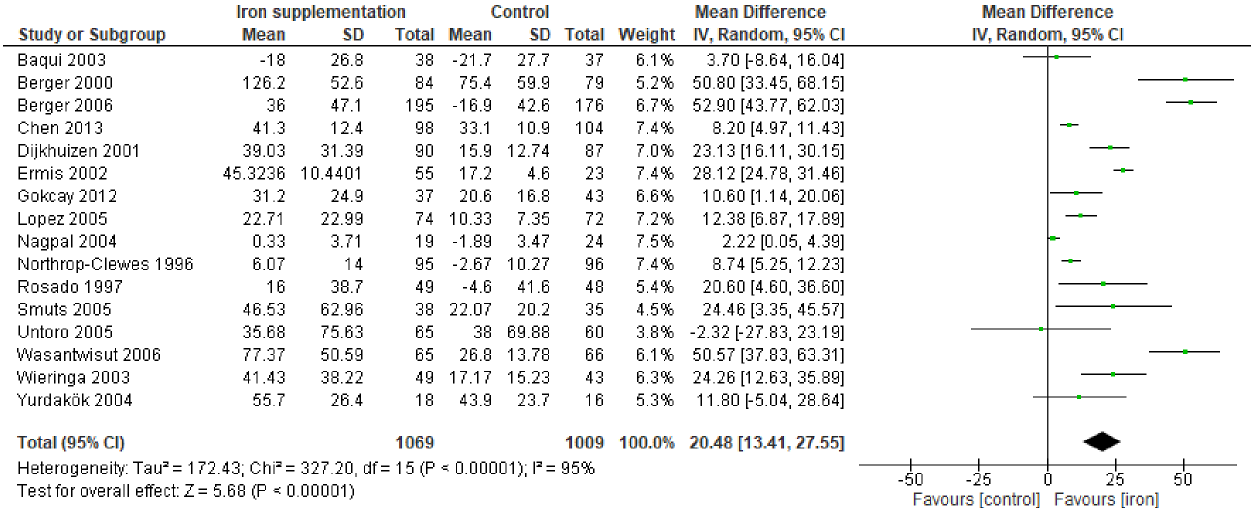

Plasma/serum ferritin (geometric means) (μg/L)

Study or Subgroup	Iron supplementation Mean	SD	Total	Control Mean	SD	Total	Weight	Mean Difference IV, Random, 95% CI
Domellof 2001	42.9	38.94	30	15.8	17.72	35	18.6%	27.10 [11.98, 42.22]
Le 2005	48.2	34.4667	49	14	10.1964	44	19.8%	34.20 [24.09, 44.31]
Lind 2003	46.5	2	136	12.9	3.7	143	20.9%	33.60 [32.91, 34.29]
Lozoff 2016	12.2	9.8588	311	11.2	9.7625	305	20.8%	1.00 [-0.55, 2.55]
Menendez 1997	65.6	54.5	166	35.1	36	164	19.8%	30.50 [20.55, 40.45]
**Total (95% CI)**			**692**			**691**	**100.0%**	**25.10 [5.07, 45.13]**

Heterogeneity: $Tau^2 = 500.54$; $Chi^2 = 1418.32$, df = 4 ($P < 0.00001$); $I^2 = 100\%$
Test for overall effect: $Z = 2.46$ ($P = 0.01$)

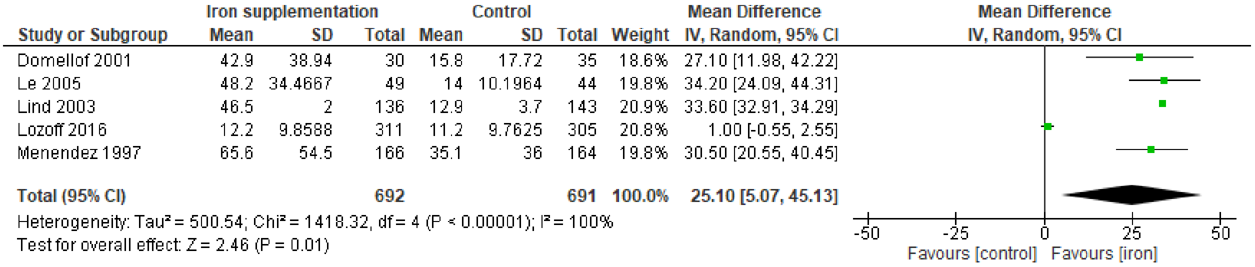

Plasma/serum retinol (μmol/L)

Study or Subgroup	Iron supplementation Mean	SD	Total	Control Mean	SD	Total	Weight	Mean Difference IV, Random, 95% CI
Chen 2013	1.15	0.24	98	1.12	0.22	104	11.1%	0.03 [-0.03, 0.09]
Le 2005	0.28	0.4178	49	0.15	0.3947	44	2.6%	0.13 [-0.04, 0.30]
Lopez 2005	-0.05	0.07	74	-0.03	0.06	72	22.4%	-0.02 [-0.04, 0.00]
Northrop-Clewes 1996	0.12	0.33	95	0.12	0.27	96	7.6%	0.00 [-0.09, 0.09]
Rosado 1997	0.27	0.5	43	-0.05	0.3	38	2.3%	0.32 [0.14, 0.50]
Smuts 2005	-0.05	0.05	38	-0.02	0.05	35	21.9%	-0.03 [-0.05, -0.01]
Untoro 2005	0.09	0.03	64	0.09	0.02	57	25.0%	0.00 [-0.01, 0.01]
Wieringa 2003	0.62	0.17	49	0.74	0.26	43	7.0%	-0.12 [-0.21, -0.03]
**Total (95% CI)**			**510**			**489**	**100.0%**	**-0.01 [-0.03, 0.02]**

Heterogeneity: Tau² = 0.00; Chi² = 30.43, df = 7 (P < 0.0001); I² = 77%
Test for overall effect: Z = 0.37 (P = 0.71)

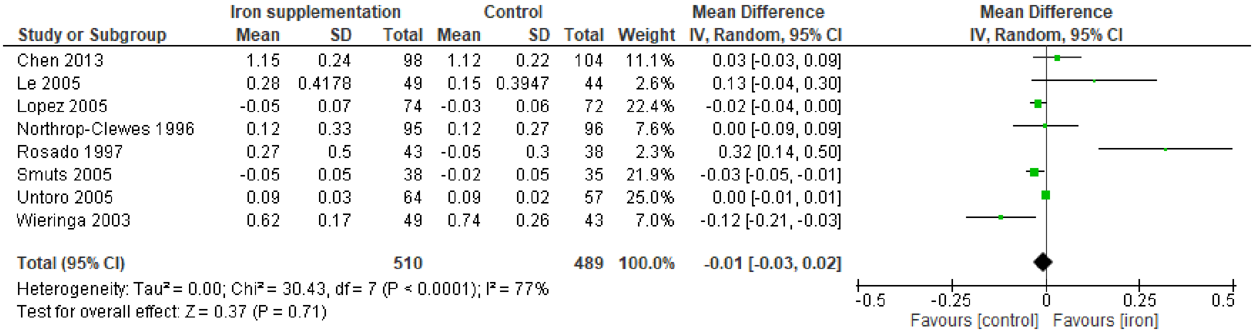

Plasma/serum zinc (μmol/L)

Study or Subgroup	Iron supplementation Mean	SD	Total	Control Mean	SD	Total	Weight	Mean Difference IV, Random, 95% CI
Baqui 2003	0.74	2	42	0.59	1.54	38	11.4%	0.15 [-0.63, 0.93]
Berger 2006	1.2	3	135	1.6	4	155	11.2%	-0.40 [-1.21, 0.41]
Dijkhuizen 2001	13.4	2.89	90	13	3.41	87	10.3%	0.40 [-0.53, 1.33]
Le 2005	-0.6	3.1333	49	-1	4.1717	42	6.6%	0.40 [-1.14, 1.94]
Lopez 2005	-0.1	0.8	74	0.5	0.7	72	14.9%	-0.60 [-0.84, -0.36]
Rosado 1997	0.28	3.4	45	0.18	3.2	54	7.8%	0.10 [-1.21, 1.41]
Smuts 2005	0.08	0.33	38	-0.31	0.43	35	15.1%	0.39 [0.21, 0.57]
Untoro 2005	-8.2	9.15	65	-5.03	6.78	60	2.8%	-3.17 [-5.98, -0.36]
Wasantwisut 2006	11.5	2.5	65	9.8	1.9	66	11.6%	1.70 [0.94, 2.46]
Wieringa 2003	13.33	2.82	49	14	3.06	43	8.4%	-0.67 [-1.88, 0.54]
**Total (95% CI)**			**652**			**652**	**100.0%**	**0.07 [-0.45, 0.59]**

Heterogeneity: Tau² = 0.46; Chi² = 67.74, df = 9 (P < 0.00001); I² = 87%
Test for overall effect: Z = 0.26 (P = 0.80)

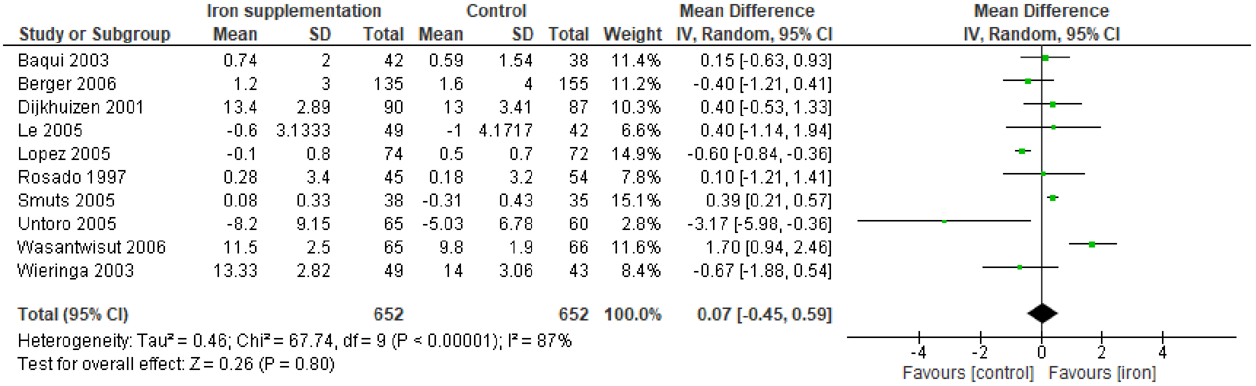

Iron deficiency (serum/plasma ferritin <12μg/L)

Study or Subgroup	Iron supplementation Events	Total	Control Events	Total	Weight	Risk Ratio M-H, Random, 95% CI
Berger 2000	9	84	40	79	11.2%	0.21 [0.11, 0.41]
Berger 2006	2	195	59	176	7.7%	0.03 [0.01, 0.12]
Chen 2013	10	98	31	104	11.2%	0.34 [0.18, 0.66]
Le 2005	0	49	22	44	3.5%	0.02 [0.00, 0.32]
Lind 2003	7	136	54	143	10.8%	0.14 [0.06, 0.29]
Lopez 2005 (1)	51	74	63	72	12.8%	0.79 [0.66, 0.94]
Lozoff 1996	5	27	16	27	10.3%	0.31 [0.13, 0.73]
Lozoff 2016	188	311	208	305	12.9%	0.89 [0.79, 1.00]
Menendez 1997	2	170	20	166	7.5%	0.10 [0.02, 0.41]
Untoro 2005	3	65	34	60	8.9%	0.08 [0.03, 0.25]
Wasantwisut 2006	0	65	4	66	3.3%	0.11 [0.01, 2.05]
**Total (95% CI)**		**1274**		**1242**	**100.0%**	**0.21 [0.12, 0.39]**
Total events	277		551			

Heterogeneity: Tau² = 0.74; Chi² = 178.57, df = 10 (P < 0.00001); I² = 94%
Test for overall effect: Z = 5.01 (P < 0.00001)

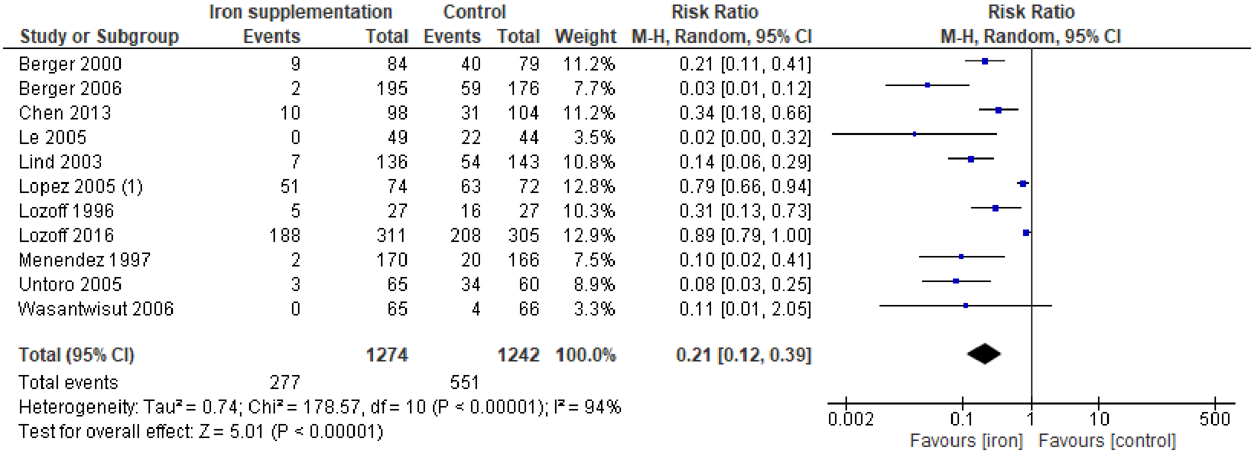

Footnotes
(1) Plasma ferritin <20ug/L

Zinc deficiency (serum/plasma retinol <10.7μmol/L)

Study or Subgroup	Iron supplementation Events	Iron supplementation Total	Control Events	Control Total	Weight	Risk Ratio M-H, Random, 95% CI
Berger 2006 (1)	4	135	1	155	1.4%	4.59 [0.52, 40.59]
Dijkhuizen 2001	15	90	21	87	11.6%	0.69 [0.38, 1.25]
Le 2005	13	49	13	42	10.5%	0.86 [0.45, 1.64]
Lind 2003	118	136	111	143	26.2%	1.12 [1.00, 1.25]
Lopez 2005	29	74	24	72	15.9%	1.18 [0.76, 1.81]
Untoro 2005	21	65	9	60	9.5%	2.15 [1.07, 4.33]
Wasantwisut 2006	29	65	44	66	19.7%	0.67 [0.49, 0.92]
Wieringa 2003	7	49	5	43	5.1%	1.23 [0.42, 3.59]
**Total (95% CI)**		**663**		**668**	**100.0%**	**1.02 [0.78, 1.33]**
Total events	236		228			

Heterogeneity: Tau² = 0.07; Chi² = 17.46, df = 7 (P = 0.01); I² = 60%
Test for overall effect: Z = 0.16 (P = 0.87)

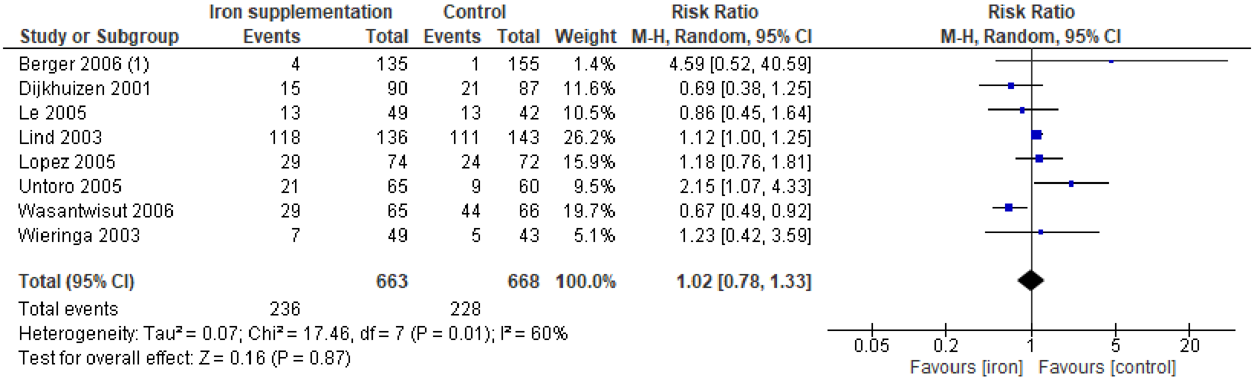

Footnotes
(1) Serum zinc <9.9umol/L

Vitamin A deficiency (serum/plasma retinol <0.7μmol/L)

Study or Subgroup	Iron supplementation Events	Iron supplementation Total	Control Events	Control Total	Weight	Risk Ratio M-H, Random, 95% CI
Chen 2013	10	98	9	104	10.9%	1.18 [0.50, 2.78]
Le 2005	4	49	3	44	3.9%	1.20 [0.28, 5.06]
Lopez 2005	5	74	4	72	4.9%	1.22 [0.34, 4.35]
Untoro 2005	10	64	6	57	8.9%	1.48 [0.58, 3.83]
Wieringa 2003	34	49	23	43	71.4%	1.30 [0.93, 1.81]
**Total (95% CI)**		**334**		**320**	**100.0%**	**1.29 [0.97, 1.71]**
Total events	63		45			

Heterogeneity: Tau² = 0.00; Chi² = 0.15, df = 4 (P = 1.00); I² = 0%
Test for overall effect: Z = 1.77 (P = 0.08)

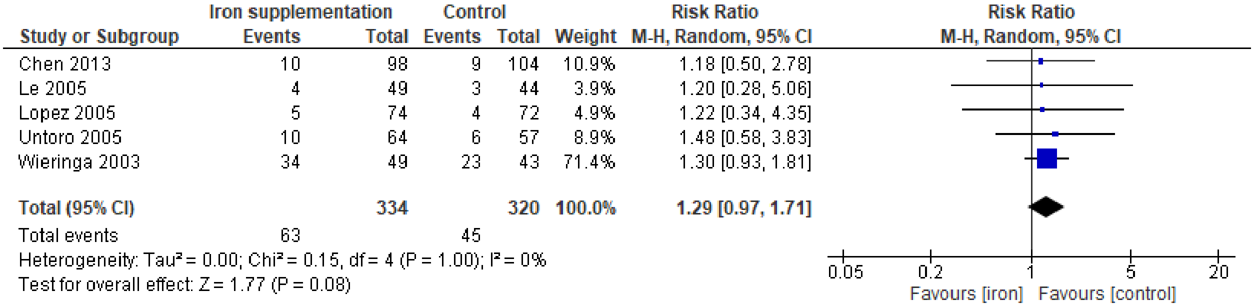

Iron-deficiency anemia

Study or Subgroup	Iron supplementation Events	Iron supplementation Total	Control Events	Control Total	Weight	Risk Ratio M-H, Random, 95% CI
Berger 2006	1	195	29	176	14.5%	0.03 [0.00, 0.23]
Dijkhuizen 2001	3	90	26	87	18.4%	0.11 [0.04, 0.36]
Lind 2003	3	136	30	143	18.3%	0.11 [0.03, 0.34]
Lozoff 2016	87	311	117	305	21.2%	0.73 [0.58, 0.92]
Wasantwisut 2006	0	65	4	66	10.6%	0.11 [0.01, 2.05]
Wieringa 2003	2	49	12	43	17.1%	0.15 [0.03, 0.62]
**Total (95% CI)**		**846**		**820**	**100.0%**	**0.14 [0.04, 0.54]**
Total events	96		218			

Heterogeneity: Tau² = 2.15; Chi² = 41.03, df = 5 (P < 0.00001); I² = 88%
Test for overall effect: Z = 2.87 (P = 0.004)

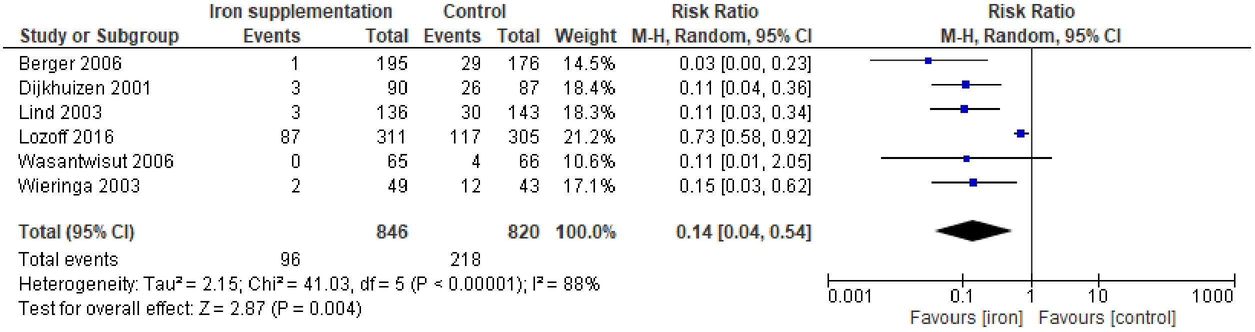

Diarrhea (cumulative incidence)

Study or Subgroup	Iron supplementation Events	Iron supplementation Total	Control Events	Control Total	Weight	Risk Ratio M-H, Random, 95% CI
Berger 2006	46	200	51	197	47.5%	0.89 [0.63, 1.26]
Domellof 2001	23	40	25	42	42.8%	0.97 [0.67, 1.39]
Nagpal 2004	1	19	2	24	1.1%	0.63 [0.06, 6.45]
Smuts 2005	9	49	10	50	8.7%	0.92 [0.41, 2.06]
**Total (95% CI)**		**308**		**313**	**100.0%**	**0.92 [0.72, 1.17]**
Total events	79		88			

Heterogeneity: Tau² = 0.00; Chi² = 0.22, df = 3 (P = 0.97); I² = 0%
Test for overall effect: Z = 0.68 (P = 0.49)

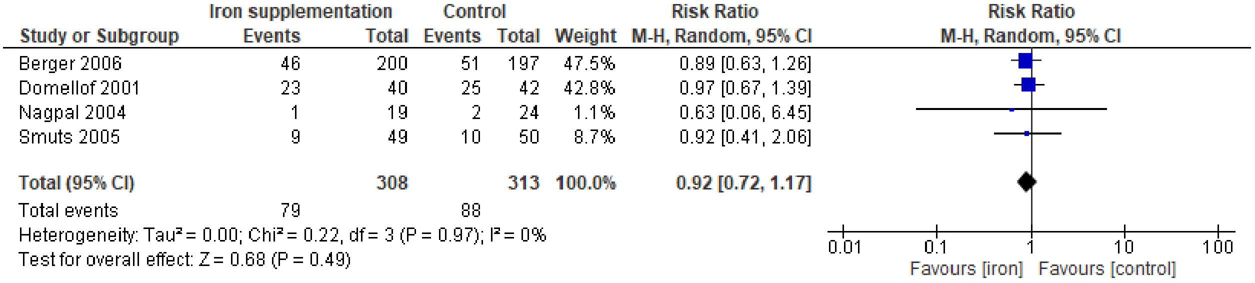

Mental development

Study or Subgroup	Iron supplementation Mean	SD	Total	Control Mean	SD	Total	Weight	Std. Mean Difference IV, Random, 95% CI	Std. Mean Difference IV, Random, 95% CI
Baqui 2003 (1)	104.3	9.5	49	102.7	13.5	45	10.9%	0.14 [-0.27, 0.54]	
Lind 2003 (2)	101	9.7	163	99	10	164	36.6%	0.20 [-0.01, 0.42]	
Stoltzfus 2001 (3)	14	3.9059	94	13.1	1.8545	85	20.3%	0.29 [-0.01, 0.58]	
Wasantwisut 2006 (4)	93.3	9.5	147	93.5	11.7	139	32.3%	-0.02 [-0.25, 0.21]	
**Total (95% CI)**			**453**			**433**	**100.0%**	**0.14 [0.01, 0.28]**	

Heterogeneity: Tau² = 0.00; Chi² = 3.09, df = 3 (P = 0.38); I² = 3%
Test for overall effect: Z = 2.06 (P = 0.04)

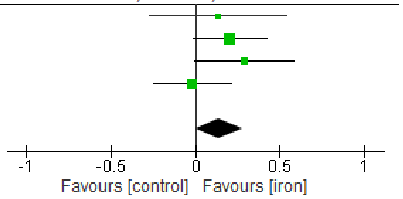

Footnotes
(1) Mental Development Index (Bayley Scales of Infant Development)
(2) Mental Development Index (Bayley Scales of Infant Development)
(3) Language (Griffiths and McCarthy scales of mental development)
(4) Full IQ (Wechsler Intelligence Scale for Children 3rd edition)

Motor development

Study or Subgroup	Iron supplementation Mean	SD	Total	Control Mean	SD	Total	Weight	Std. Mean Difference IV, Random, 95% CI	Std. Mean Difference IV, Random, 95% CI
Baqui 2003 (1)	99.5	16.6	49	95.4	16.3	45	9.3%	0.25 [-0.16, 0.65]	
Lind 2003 (2)	106	11	163	103	10.8	165	32.3%	0.27 [0.06, 0.49]	
Lozoff 2016 (3)	90	8.875	305	87.5	8.6221	288	58.4%	0.29 [0.12, 0.45]	
**Total (95% CI)**			**517**			**498**	**100.0%**	**0.28 [0.15, 0.40]**	

Heterogeneity: Tau² = 0.00; Chi² = 0.03, df = 2 (P = 0.98); I² = 0%
Test for overall effect: Z = 4.41 (P < 0.0001)

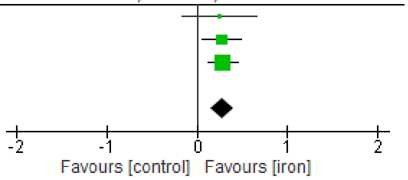

Footnotes
(1) Psychomotor Development Index (Bayley Scales of Infant Development)
(2) Psychomotor Development Index (Bayley Scales of Infant Development)
(3) Gross motor score (Peabody Developmental Motor Scale 2nd Edition)

Comparison 3: Subgroup Analyses for Iron Supplementation vs. Placebo/No Intervention (Efficacy)

Anemia: WHO region

	Iron supplementation		Control			Risk Ratio
Study or Subgroup	Events	Total	Events	Total	Weight	M-H, Random, 95% CI
**13.1.1 Africa**						
Berger 2000	46	84	50	79	10.9%	0.87 [0.67, 1.12]
Massaga 2003	12	74	26	72	6.8%	0.45 [0.25, 0.82]
**Subtotal (95% CI)**		**158**		**151**	**17.7%**	**0.66 [0.34, 1.28]**
Total events	58		76			
Heterogeneity: Tau² = 0.18; Chi² = 4.26, df = 1 (P = 0.04); I² = 77%						
Test for overall effect: Z = 1.23 (P = 0.22)						
**13.1.2 Americas**						
Lopez 2005	17	74	32	72	8.0%	0.52 [0.32, 0.84]
**Subtotal (95% CI)**		**74**		**72**	**8.0%**	**0.52 [0.32, 0.84]**
Total events	17		32			
Heterogeneity: Not applicable						
Test for overall effect: Z = 2.64 (P = 0.008)						
**13.1.3 South-east Asia**						
Dijkhuizen 2001	25	90	57	87	9.6%	0.42 [0.29, 0.61]
Lind 2003	34	136	63	143	9.8%	0.57 [0.40, 0.80]
Palupi 1997	17	96	26	98	7.4%	0.67 [0.39, 1.15]
Untoro 2005	21	58	34	65	9.0%	0.69 [0.46, 1.05]
Wasantwisut 2006	11	65	40	66	7.1%	0.28 [0.16, 0.50]
Wieringa 2003	10	49	21	43	6.5%	0.42 [0.22, 0.79]
**Subtotal (95% CI)**		**494**		**502**	**49.3%**	**0.50 [0.39, 0.64]**
Total events	118		241			
Heterogeneity: Tau² = 0.04; Chi² = 9.07, df = 5 (P = 0.11); I² = 45%						
Test for overall effect: Z = 5.40 (P < 0.00001)						
**13.1.4 Western Pacific**						
Berger 2006	16	197	59	195	7.7%	0.27 [0.16, 0.45]
Chen 2013	9	98	27	104	5.8%	0.35 [0.18, 0.71]
Lozoff 2016	108	311	138	305	11.6%	0.77 [0.63, 0.93]
**Subtotal (95% CI)**		**606**		**604**	**25.1%**	**0.43 [0.20, 0.95]**
Total events	133		224			
Heterogeneity: Tau² = 0.42; Chi² = 17.99, df = 2 (P = 0.0001); I² = 89%						
Test for overall effect: Z = 2.09 (P = 0.04)						
**Total (95% CI)**		**1332**		**1329**	**100.0%**	**0.52 [0.41, 0.65]**
Total events	326		573			
Heterogeneity: Tau² = 0.11; Chi² = 40.68, df = 11 (P < 0.0001); I² = 73%						
Test for overall effect: Z = 5.65 (P < 0.00001)						
Test for subgroup differences: Chi² = 0.76, df = 3 (P = 0.86), I² = 0%						

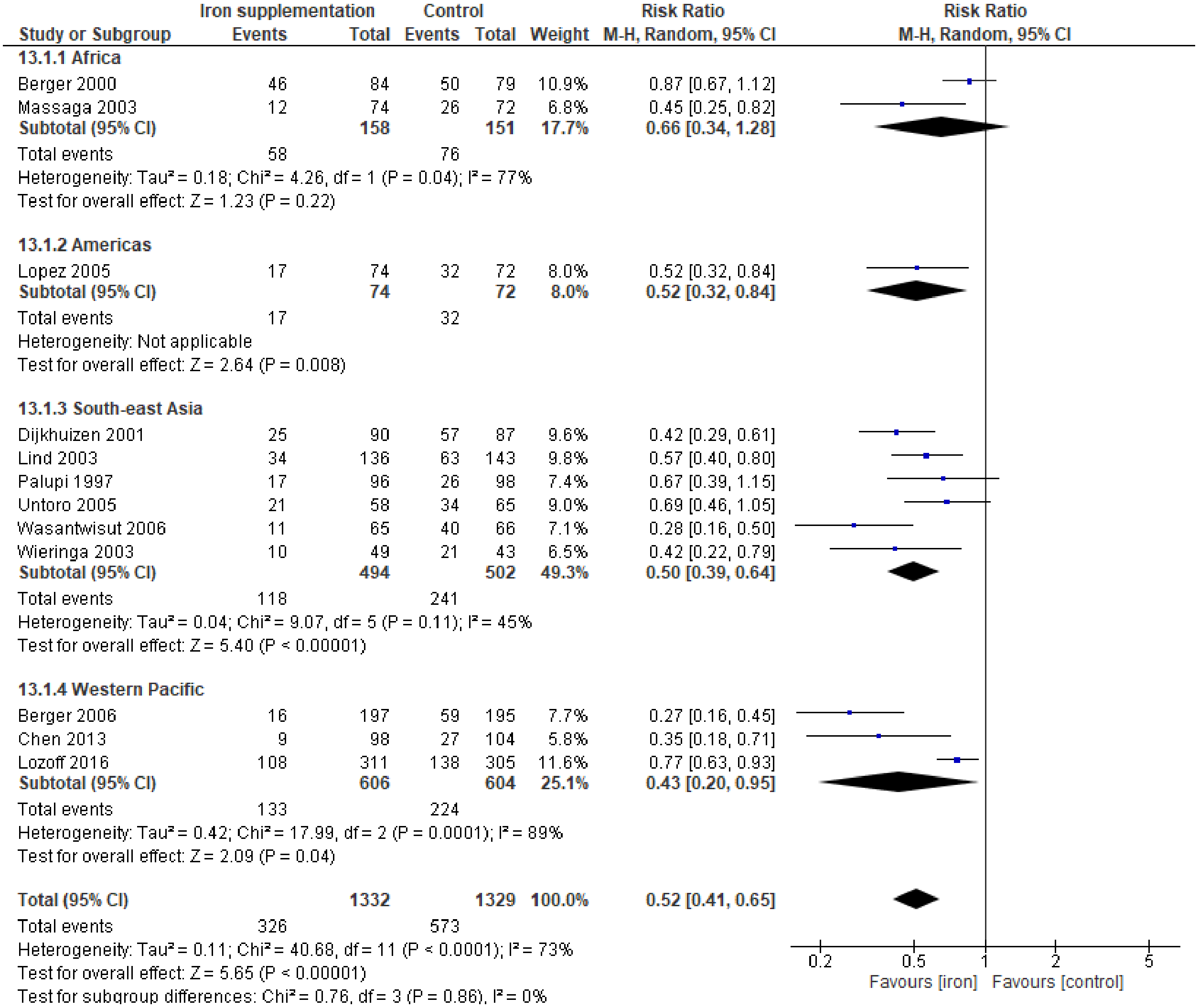

Anemia: mean age at baseline

	Iron supplementation		Control			Risk Ratio
Study or Subgroup	Events	Total	Events	Total	Weight	M-H, Random, 95% CI
**13.2.1 1-5 months**						
Berger 2006	16	197	59	195	8.6%	0.27 [0.16, 0.45]
Chen 2013	9	98	27	104	6.3%	0.35 [0.18, 0.71]
Dijkhuizen 2001	25	90	57	87	10.8%	0.42 [0.29, 0.61]
Lozoff 2016	108	311	138	305	13.4%	0.77 [0.63, 0.93]
Massaga 2003	12	74	26	72	7.5%	0.45 [0.25, 0.82]
Wasantwisut 2006	11	65	40	66	7.8%	0.28 [0.16, 0.50]
Wieringa 2003	10	49	21	43	7.1%	0.42 [0.22, 0.79]
**Subtotal (95% CI)**		**884**		**872**	**61.5%**	**0.41 [0.28, 0.60]**
Total events	191		368			
Heterogeneity: Tau² = 0.20; Chi² = 29.51, df = 6 (P < 0.0001); I² = 80%						
Test for overall effect: Z = 4.55 (P < 0.00001)						
**13.2.2 6-11 months**						
Lind 2003	34	136	63	143	11.2%	0.57 [0.40, 0.80]
Lopez 2005	17	74	32	72	8.9%	0.52 [0.32, 0.84]
Untoro 2005	21	58	34	65	10.1%	0.69 [0.46, 1.05]
**Subtotal (95% CI)**		**268**		**280**	**30.2%**	**0.59 [0.47, 0.75]**
Total events	72		129			
Heterogeneity: Tau² = 0.00; Chi² = 0.91, df = 2 (P = 0.63); I² = 0%						
Test for overall effect: Z = 4.41 (P < 0.0001)						
**13.2.3 24-59 months**						
Palupi 1997	17	96	26	98	8.2%	0.67 [0.39, 1.15]
**Subtotal (95% CI)**		**96**		**98**	**8.2%**	**0.67 [0.39, 1.15]**
Total events	17		26			
Heterogeneity: Not applicable						
Test for overall effect: Z = 1.46 (P = 0.14)						
**Total (95% CI)**		**1248**		**1250**	**100.0%**	**0.49 [0.38, 0.61]**
Total events	280		523			
Heterogeneity: Tau² = 0.10; Chi² = 30.86, df = 10 (P = 0.0006); I² = 68%						
Test for overall effect: Z = 6.05 (P < 0.00001)						
Test for subgroup differences: Chi² = 3.06, df = 2 (P = 0.22), I² = 34.7%						

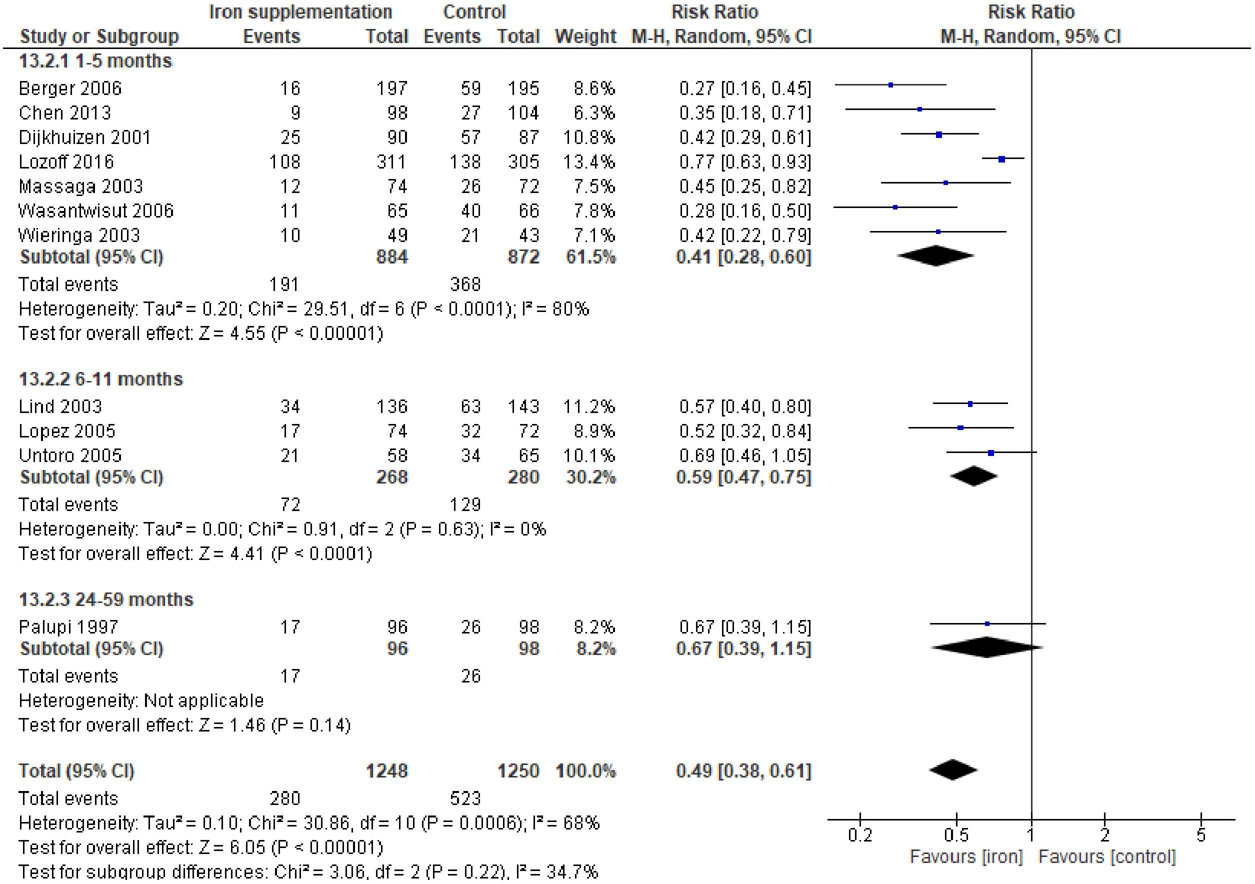

Anemia: intervention duration

Study or Subgroup	Iron supplementation Events	Iron supplementation Total	Control Events	Control Total	Weight	Risk Ratio M-H, Random, 95% CI
**13.3.1 3-5 months**						
Berger 2000	46	84	50	79	10.9%	0.87 [0.67, 1.12]
Berger 2006	16	197	59	195	7.7%	0.27 [0.16, 0.45]
Chen 2013	9	98	27	104	5.8%	0.35 [0.18, 0.71]
Dijkhuizen 2001	25	90	57	87	9.6%	0.42 [0.29, 0.61]
Lind 2003	34	136	63	143	9.8%	0.57 [0.40, 0.80]
Lopez 2005	17	74	32	72	8.0%	0.52 [0.32, 0.84]
Massaga 2003	12	74	26	72	6.8%	0.45 [0.25, 0.82]
Palupi 1997	17	96	26	98	7.4%	0.67 [0.39, 1.15]
Untoro 2005	21	58	34	65	9.0%	0.69 [0.46, 1.05]
Wasantwisut 2006	11	65	40	66	7.1%	0.28 [0.16, 0.50]
Wieringa 2003	10	49	21	43	6.5%	0.42 [0.22, 0.79]
**Subtotal (95% CI)**		**1021**		**1024**	**88.4%**	**0.49 [0.38, 0.63]**
Total events	218		435			
Heterogeneity: Tau² = 0.12; Chi² = 33.21, df = 10 (P = 0.0003); I² = 70%						
Test for overall effect: Z = 5.58 (P < 0.00001)						
**13.3.2 6-12 months**						
Lozoff 2016	108	311	138	305	11.6%	0.77 [0.63, 0.93]
**Subtotal (95% CI)**		**311**		**305**	**11.6%**	**0.77 [0.63, 0.93]**
Total events	108		138			
Heterogeneity: Not applicable						
Test for overall effect: Z = 2.64 (P = 0.008)						
**Total (95% CI)**		**1332**		**1329**	**100.0%**	**0.52 [0.41, 0.65]**
Total events	326		573			
Heterogeneity: Tau² = 0.11; Chi² = 40.68, df = 11 (P < 0.0001); I² = 73%						
Test for overall effect: Z = 5.65 (P < 0.00001)						
Test for subgroup differences: Chi² = 7.70, df = 1 (P = 0.006), I² = 87.0%						

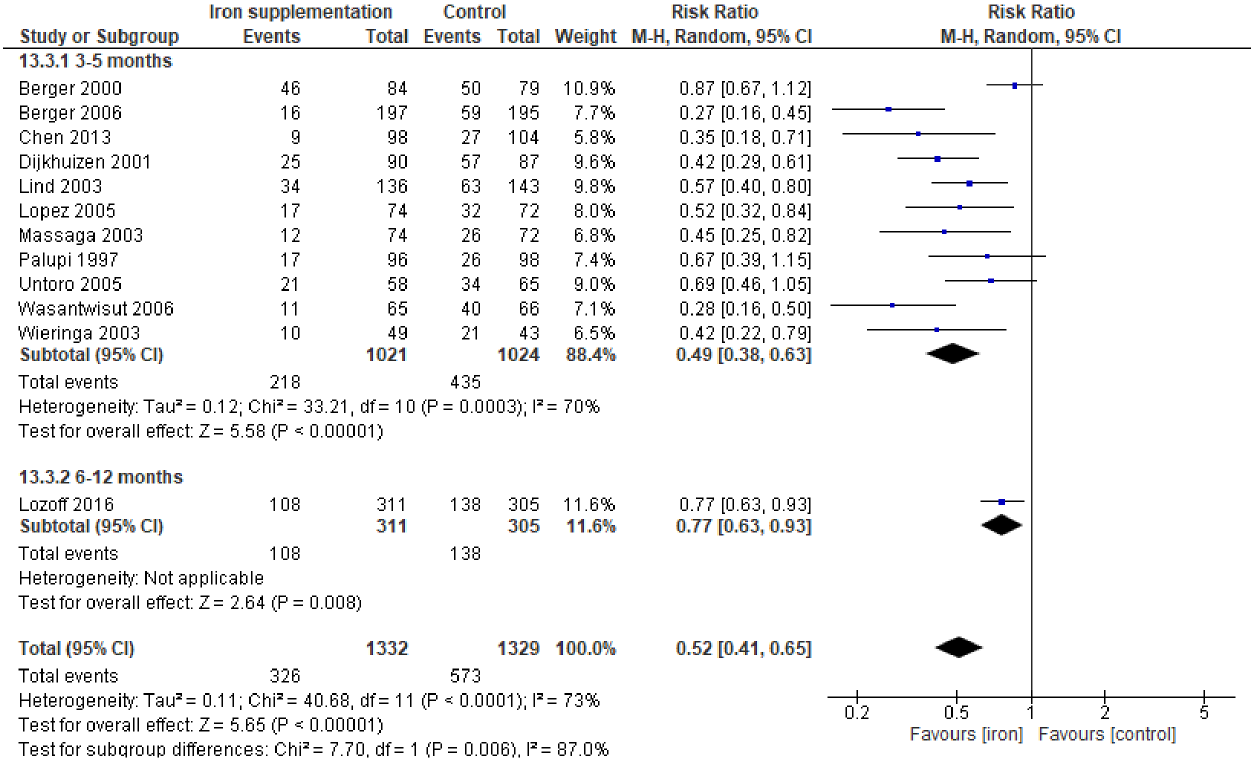

Anemia: intervention frequency

Study or Subgroup	Iron supplementation Events	Iron supplementation Total	Control Events	Control Total	Weight	Risk Ratio M-H, Random, 95% CI
**13.4.1 Daily**						
Berger 2000	46	84	50	79	10.9%	0.87 [0.67, 1.12]
Berger 2006	16	197	59	195	7.7%	0.27 [0.16, 0.45]
Chen 2013	9	98	27	104	5.8%	0.35 [0.18, 0.71]
Dijkhuizen 2001	25	90	57	87	9.6%	0.42 [0.29, 0.61]
Lind 2003	34	136	63	143	9.8%	0.57 [0.40, 0.80]
Lopez 2005	17	74	32	72	8.0%	0.52 [0.32, 0.84]
Lozoff 2016	108	311	138	305	11.6%	0.77 [0.63, 0.93]
Massaga 2003	12	74	26	72	6.8%	0.45 [0.25, 0.82]
Untoro 2005	21	58	34	65	9.0%	0.69 [0.46, 1.05]
Wasantwisut 2006	11	65	40	66	7.1%	0.28 [0.16, 0.50]
Wieringa 2003	10	49	21	43	6.5%	0.42 [0.22, 0.79]
**Subtotal (95% CI)**		**1236**		**1231**	**92.6%**	**0.50 [0.39, 0.64]**
Total events	309		547			
Heterogeneity: Tau² = 0.12; Chi² = 40.73, df = 10 (P < 0.0001); I² = 75%						
Test for overall effect: Z = 5.46 (P < 0.00001)						
**13.4.2 Intermittent**						
Palupi 1997	17	96	26	98	7.4%	0.67 [0.39, 1.15]
**Subtotal (95% CI)**		**96**		**98**	**7.4%**	**0.67 [0.39, 1.15]**
Total events	17		26			
Heterogeneity: Not applicable						
Test for overall effect: Z = 1.46 (P = 0.14)						
**Total (95% CI)**		**1332**		**1329**	**100.0%**	**0.52 [0.41, 0.65]**
Total events	326		573			
Heterogeneity: Tau² = 0.11; Chi² = 40.68, df = 11 (P < 0.0001); I² = 73%						
Test for overall effect: Z = 5.65 (P < 0.00001)						
Test for subgroup differences: Chi² = 0.86, df = 1 (P = 0.35), I² = 0%						

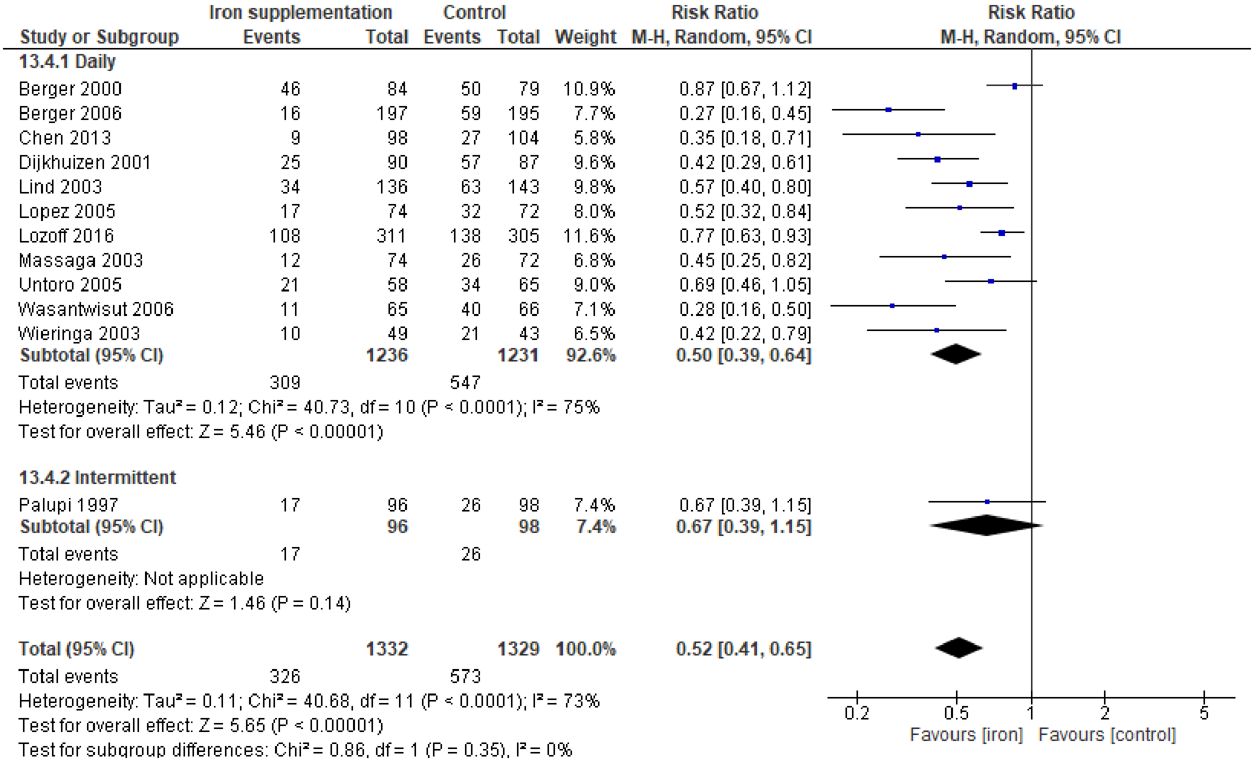

Anemia: nutritional status at baseline (anemic vs. non-anemic)

Study or Subgroup	Iron supplementation Events	Iron supplementation Total	Control Events	Control Total	Weight	Risk Ratio M-H, Random, 95% CI
**13.5.1 Anemic**						
Berger 2000	46	84	50	79	19.1%	0.87 [0.67, 1.12]
Lopez 2005	17	74	32	72	13.8%	0.52 [0.32, 0.84]
Untoro 2005	21	58	34	65	15.5%	0.69 [0.46, 1.05]
**Subtotal (95% CI)**		**216**		**216**	**48.4%**	**0.72 [0.53, 0.96]**
Total events	84		116			
Heterogeneity: Tau² = 0.03; Chi² = 3.75, df = 2 (P = 0.15); I² = 47%						
Test for overall effect: Z = 2.21 (P = 0.03)						
**13.5.2 Non-anemic**						
Chen 2013	9	98	27	104	9.8%	0.35 [0.18, 0.71]
Lind 2003	34	136	63	143	17.1%	0.57 [0.40, 0.80]
Palupi 1997	17	96	26	98	12.7%	0.67 [0.39, 1.15]
Wasantwisut 2006	11	65	40	66	12.1%	0.28 [0.16, 0.50]
**Subtotal (95% CI)**		**395**		**411**	**51.6%**	**0.46 [0.31, 0.68]**
Total events	71		156			
Heterogeneity: Tau² = 0.08; Chi² = 6.53, df = 3 (P = 0.09); I² = 54%						
Test for overall effect: Z = 3.96 (P < 0.0001)						
**Total (95% CI)**		**611**		**627**	**100.0%**	**0.56 [0.42, 0.75]**
Total events	155		272			
Heterogeneity: Tau² = 0.10; Chi² = 18.69, df = 6 (P = 0.005); I² = 68%						
Test for overall effect: Z = 3.87 (P = 0.0001)						
Test for subgroup differences: Chi² = 3.18, df = 1 (P = 0.07), I² = 68.5%						

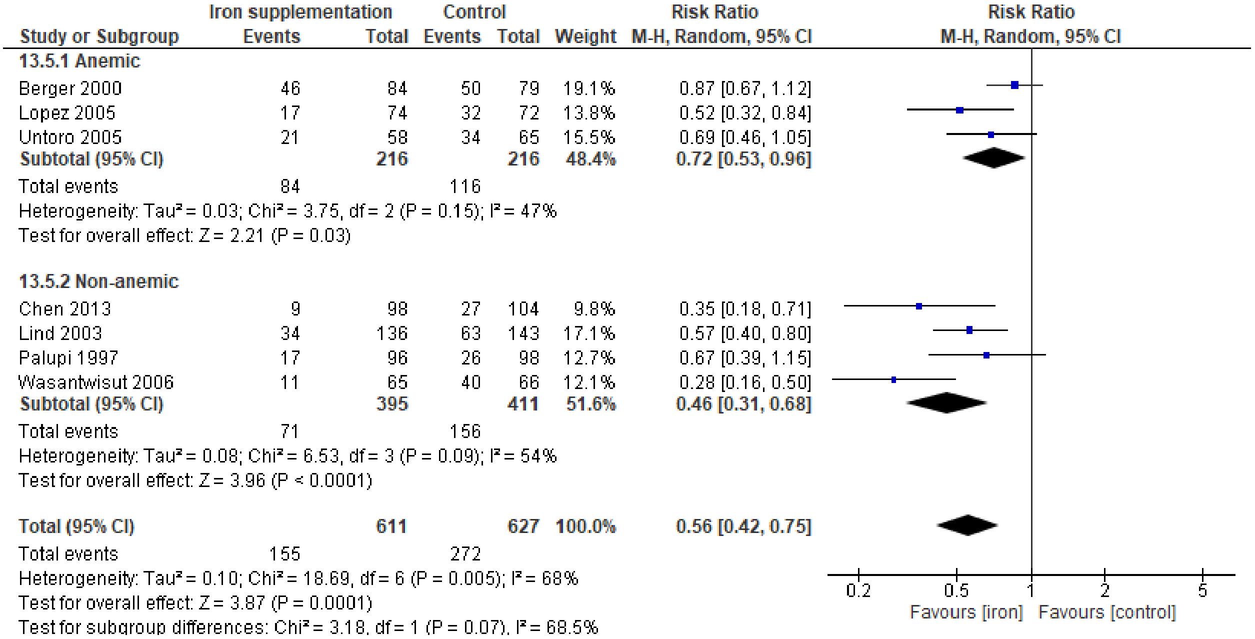

Comparison 4: Iron-folic Acid Supplementation vs. Placebo/No intervention (Efficacy)

Hemoglobin (g/L)

Study or Subgroup	Mean Difference	SE	Weight	Mean Difference IV, Random, 95% CI
Chang 2010	4	2.1567	13.3%	4.00 [-0.23, 8.23]
Sazawal 2006	11.7	2.2052	12.9%	11.70 [7.38, 16.02]
Sharma 2011	2.09	0.3397	36.4%	2.09 [1.42, 2.76]
Tielsch 2006	0.71	0.1888	37.5%	0.71 [0.34, 1.08]
**Total (95% CI)**			**100.0%**	**3.06 [1.16, 4.97]**
Heterogeneity: Tau² = 2.49; Chi² = 37.67, df = 3 (P < 0.00001); I² = 92%				
Test for overall effect: Z = 3.15 (P = 0.002)				

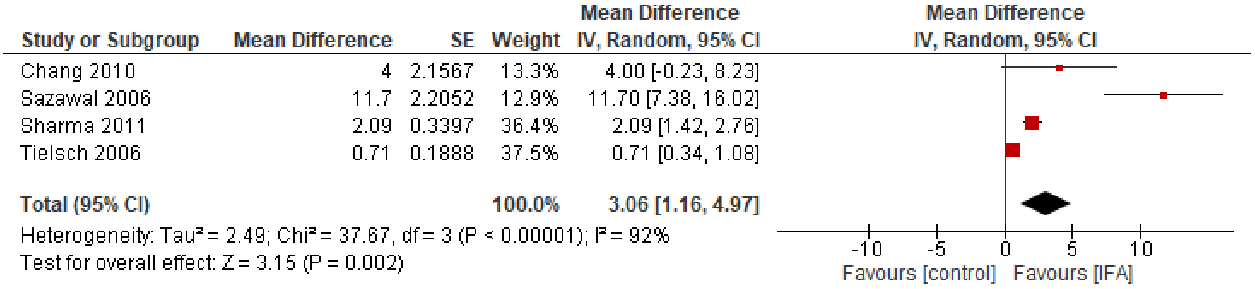

Diarrhea (incidence)

Study or Subgroup	log[Rate Ratio]	SE	Weight	Rate Ratio IV, Random, 95% CI
Chang 2010	-0.0117	0.0386	66.0%	0.99 [0.92, 1.07]
Sazawal 2006	-0.0834	0.1542	4.1%	0.92 [0.68, 1.24]
Tielsch 2006	-0.0619	0.0574	29.9%	0.94 [0.84, 1.05]
**Total (95% CI)**			**100.0%**	**0.97 [0.91, 1.03]**
Heterogeneity: Tau² = 0.00; Chi² = 0.65, df = 2 (P = 0.72); I² = 0%				
Test for overall effect: Z = 0.95 (P = 0.34)				

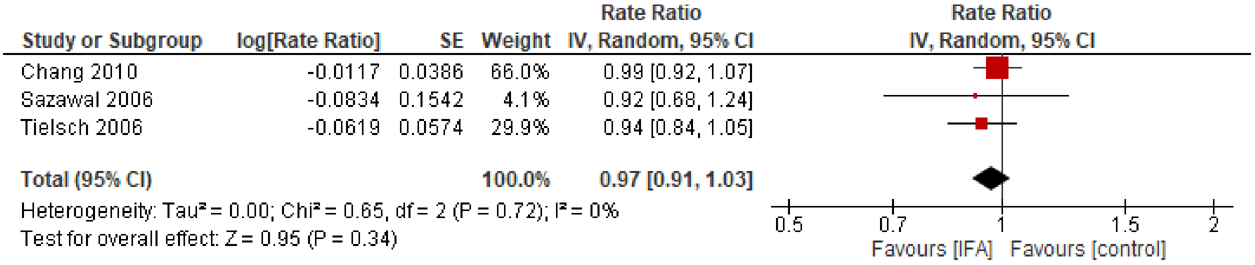

Comparison 4: Subgroup Analyses for Iron-folic Acid Supplementation vs. Placebo/No Intervention (Efficacy)

Anemia: WHO region

Study or Subgroup	IFA supplementation Events	IFA supplementation Total	Control Events	Control Total	Weight	Risk Ratio M-H, Random, 95% CI
**16.1.1 Africa**						
Sazawal 2006 (1)	36	54	44	58	25.0%	0.88 [0.69, 1.11]
**Subtotal (95% CI)**		**54**		**58**	**25.0%**	**0.88 [0.69, 1.11]**
Total events	36		44			
Heterogeneity: Not applicable						
Test for overall effect: Z = 1.06 (P = 0.29)						
**16.1.2 South-east Asia**						
Chang 2010	75	93	77	89	34.2%	0.93 [0.82, 1.06]
Sharma 2011	12	22	21	22	15.1%	0.57 [0.39, 0.85]
Tielsch 2006	56	124	94	152	25.6%	0.73 [0.58, 0.92]
**Subtotal (95% CI)**		**239**		**263**	**75.0%**	**0.76 [0.57, 1.01]**
Total events	143		192			
Heterogeneity: Tau² = 0.05; Chi² = 8.90, df = 2 (P = 0.01); I² = 78%						
Test for overall effect: Z = 1.89 (P = 0.06)						
**Total (95% CI)**		**293**		**321**	**100.0%**	**0.80 [0.66, 0.97]**
Total events	179		236			
Heterogeneity: Tau² = 0.02; Chi² = 8.64, df = 3 (P = 0.03); I² = 65%						
Test for overall effect: Z = 2.25 (P = 0.02)						
Test for subgroup differences: Chi² = 0.58, df = 1 (P = 0.45), I² = 0%						

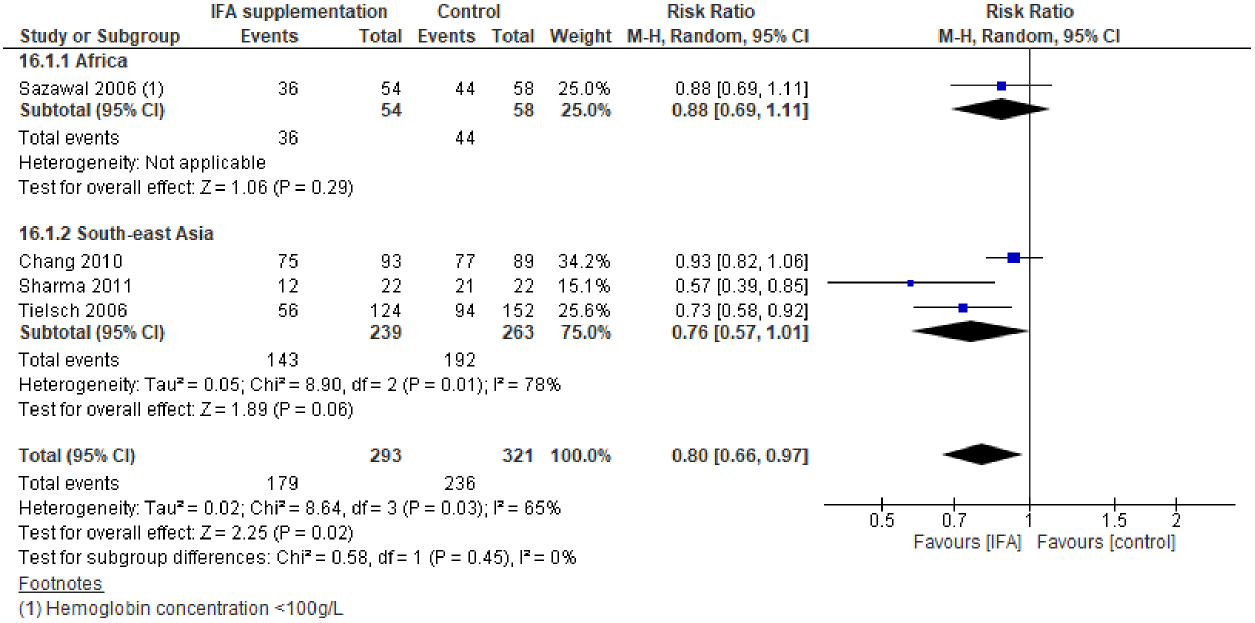

Footnotes

(1) Hemoglobin concentration <100g/L

Anemia: intervention frequency

Study or Subgroup	IFA supplementation Events	IFA supplementation Total	Control Events	Control Total	Weight	Risk Ratio M-H, Random, 95% CI
**16.2.1 Daily**						
Chang 2010	75	93	77	89	25.6%	0.93 [0.82, 1.06]
Sazawal 2006 (1)	36	54	44	58	21.9%	0.88 [0.69, 1.11]
Sharma 2011	12	22	21	22	16.1%	0.57 [0.39, 0.85]
Tielsch 2006	56	124	94	152	22.1%	0.73 [0.58, 0.92]
**Subtotal (95% CI)**		**293**		**321**	**85.7%**	**0.80 [0.66, 0.97]**
Total events	179		236			
Heterogeneity: Tau² = 0.02; Chi² = 8.64, df = 3 (P = 0.03); I² = 65%						
Test for overall effect: Z = 2.25 (P = 0.02)						
**16.2.2 Intermittent**						
Sharma 2011	12	30	29	31	14.3%	0.43 [0.27, 0.67]
**Subtotal (95% CI)**		**30**		**31**	**14.3%**	**0.43 [0.27, 0.67]**
Total events	12		29			
Heterogeneity: Not applicable						
Test for overall effect: Z = 3.72 (P = 0.0002)						
**Total (95% CI)**		**323**		**352**	**100.0%**	**0.72 [0.56, 0.92]**
Total events	191		265			
Heterogeneity: Tau² = 0.06; Chi² = 18.64, df = 4 (P = 0.0009); I² = 79%						
Test for overall effect: Z = 2.63 (P = 0.009)						
Test for subgroup differences: Chi² = 6.37, df = 1 (P = 0.01), I² = 84.3%						

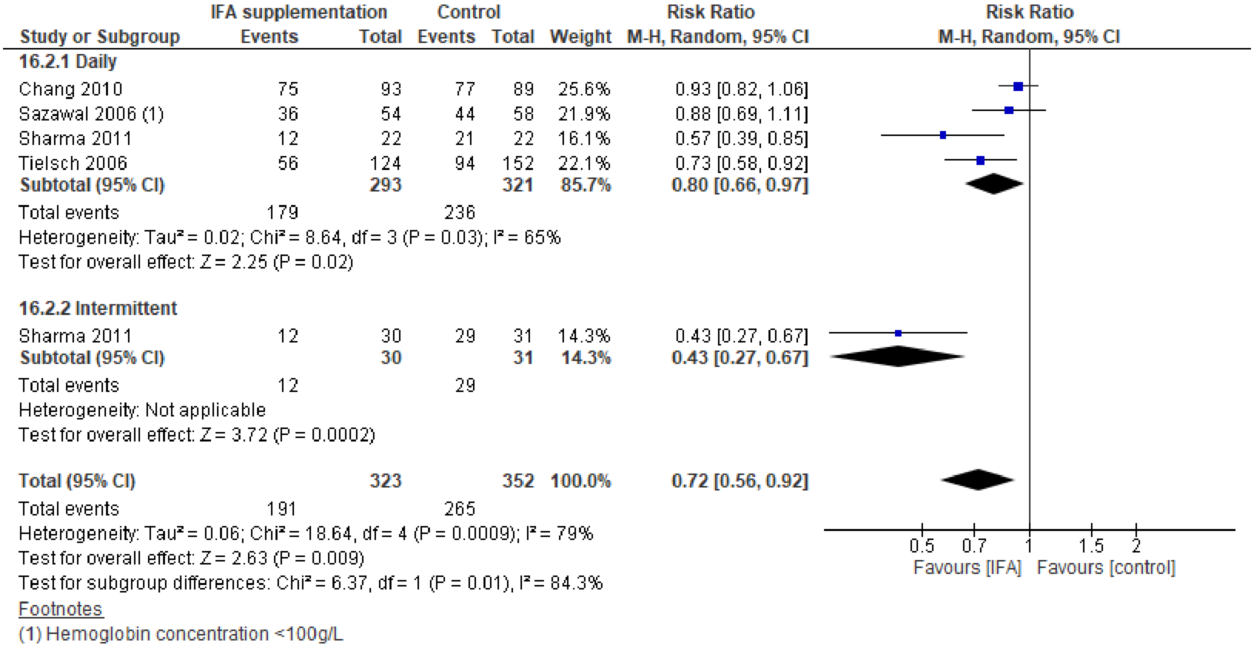

Footnotes

(1) Hemoglobin concentration <100g/L

Comparison 5: MMN Supplementation vs. Placebo/No Intervention (Efficacy)

Height (cm)

	MMN supplementation			Control				Mean Difference
Study or Subgroup	Mean	SD	Total	Mean	SD	Total	Weight	IV, Random, 95% CI
Lopez 2005	88.6	10.3	409	88.1	11	407	5.7%	0.50 [-0.96, 1.96]
Rivera 2001	10.7565	2.4157	161	10.3272	2.2166	158	47.0%	0.43 [-0.08, 0.94]
Smuts 2005	76.7	3.5	49	76.6	3.3	50	6.8%	0.10 [-1.24, 1.44]
Thu 1999	5.4	1.3	55	5.1	1.6	54	40.5%	0.30 [-0.25, 0.85]
**Total (95% CI)**			**674**			**669**	**100.0%**	**0.36 [0.01, 0.71]**

Heterogeneity: Tau² = 0.00; Chi² = 0.30, df = 3 (P = 0.96); I² = 0%
Test for overall effect: Z = 2.02 (P = 0.04)

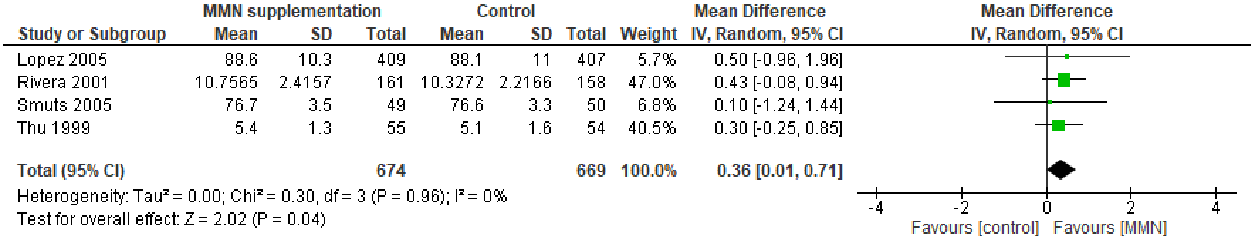

Weight (kg)

	MMN supplementation			Control				Mean Difference
Study or Subgroup	Mean	SD	Total	Mean	SD	Total	Weight	IV, Random, 95% CI
Lopez 2005	13.6	3.1	409	13.5	3.2	407	12.1%	0.10 [-0.33, 0.53]
Rivera 2001	2.0313	0.8017	161	2.1354	0.8066	158	72.9%	-0.10 [-0.28, 0.07]
Smuts 2005	10.6	1.5	49	10.6	1.3	50	7.4%	0.00 [-0.55, 0.55]
Thu 1999	5.4	1.3	55	5.1	1.6	54	7.6%	0.30 [-0.25, 0.85]
**Total (95% CI)**			**674**			**669**	**100.0%**	**-0.04 [-0.19, 0.11]**

Heterogeneity: Tau² = 0.00; Chi² = 2.41, df = 3 (P = 0.49); I² = 0%
Test for overall effect: Z = 0.53 (P = 0.59)

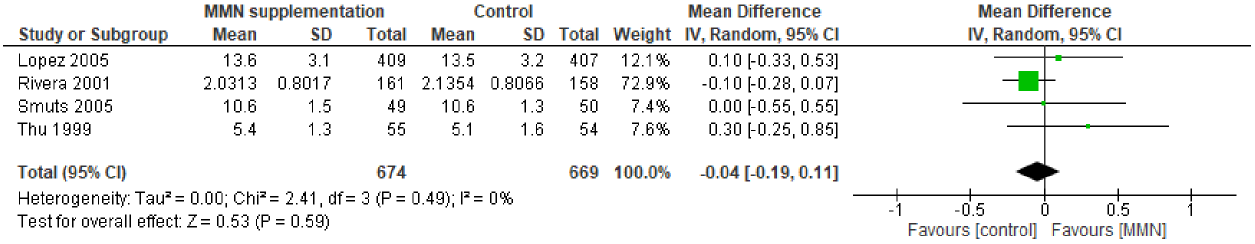

Length-for-age (z-score)

	MMN supplementation			Control				Mean Difference
Study or Subgroup	Mean	SD	Total	Mean	SD	Total	Weight	IV, Random, 95% CI
Adu-Afarwuah 2007	-0.44	1	101	-0.4	1	81	6.2%	-0.04 [-0.33, 0.25]
Baqui 2003	-1.7	0.8	35	-1.7	1	45	3.9%	0.00 [-0.39, 0.39]
Fahmida 2007	-1.48	0.94	194	-1.47	0.94	192	11.1%	-0.01 [-0.20, 0.18]
Le 2005	-0.32	0.4359	76	-0.49	0.4272	73	14.9%	0.17 [0.03, 0.31]
Lopez 2005	-0.18	0.5	63	-0.09	0.5	72	12.4%	-0.09 [-0.26, 0.08]
McDonald 2015	-0.79	1.2093	585	-0.93	1.2083	584	14.9%	0.14 [0.00, 0.28]
Rivera 2001	0.023	0.6338	161	-0.2504	0.7155	158	14.0%	0.27 [0.12, 0.42]
Smuts 2005	-0.3	0.65	49	-0.3	0.65	50	7.5%	0.00 [-0.26, 0.26]
Thu 1999	-0.12	0.63	55	-0.15	0.53	54	9.3%	0.03 [-0.19, 0.25]
Untoro 2005	-1.51	0.85	67	-1.77	0.96	65	5.7%	0.26 [-0.05, 0.57]
**Total (95% CI)**			**1386**			**1374**	**100.0%**	**0.09 [0.00, 0.17]**

Heterogeneity: Tau² = 0.01; Chi² = 15.85, df = 9 (P = 0.07); I² = 43%
Test for overall effect: Z = 2.03 (P = 0.04)

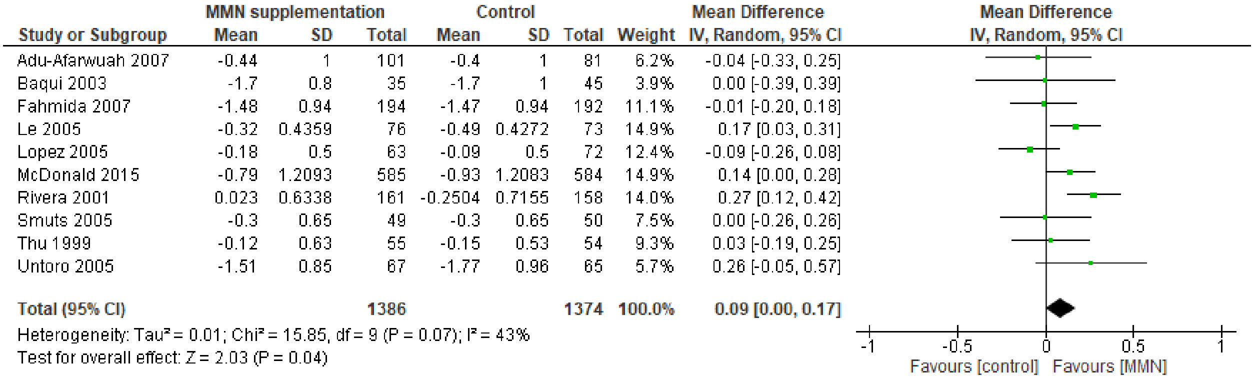

Weight-for-age (z-score)

	MMN supplementation			Control				Mean Difference
Study or Subgroup	Mean	SD	Total	Mean	SD	Total	Weight	IV, Random, 95% CI
Adu-Afarwuah 2007	-0.88	1.1	101	-0.74	1.1	81	3.5%	-0.14 [-0.46, 0.18]
Baqui 2003	-2.1	1	35	-2	1.2	45	1.6%	-0.10 [-0.58, 0.38]
Fahmida 2007	-1.86	0.85	194	-1.86	0.93	192	11.4%	0.00 [-0.18, 0.18]
Le 2005	-0.58	0.4359	76	-0.66	0.4272	73	18.4%	0.08 [-0.06, 0.22]
Lopez 2005	-0.35	0.6	63	-0.33	0.6	72	8.8%	-0.02 [-0.22, 0.18]
McDonald 2015	-0.36	0.9675	585	-0.5	0.9666	584	28.1%	0.14 [0.03, 0.25]
Rivera 2001	-0.1226	0.658	161	-0.0684	0.7247	158	15.4%	-0.05 [-0.21, 0.10]
Smuts 2005	-0.25	0.84	49	-0.5	0.91	50	3.1%	0.25 [-0.09, 0.59]
Thu 1999	0.06	0.74	55	-0.04	0.54	54	6.2%	0.10 [-0.14, 0.34]
Untoro 2005	-1.34	0.95	67	-1.54	0.93	65	3.6%	0.20 [-0.12, 0.52]
**Total (95% CI)**			**1386**			**1374**	**100.0%**	**0.06 [-0.00, 0.12]**

Heterogeneity: Tau² = 0.00; Chi² = 9.19, df = 9 (P = 0.42); I² = 2%
Test for overall effect: Z = 1.89 (P = 0.06)

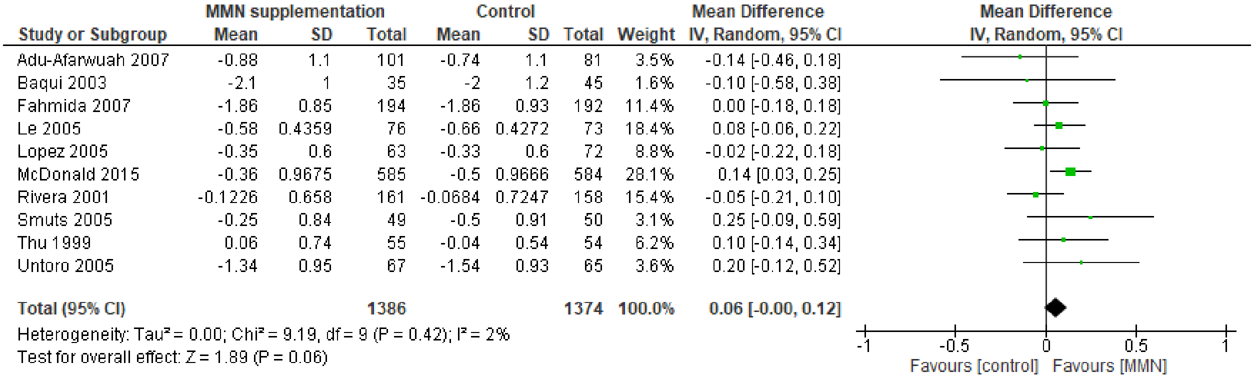

Weight-for-height (z-score)

Study or Subgroup	MMN supplementation Mean	SD	Total	Control Mean	SD	Total	Weight	Mean Difference IV, Random, 95% CI
Adu-Afarwuah 2007	-0.89	1.1	101	-0.74	1.1	81	5.2%	-0.15 [-0.47, 0.17]
Baqui 2003	-0.8	0.8	35	-0.7	0.9	45	3.9%	-0.10 [-0.47, 0.27]
Fahmida 2007	-0.98	0.93	194	-1	0.93	192	15.8%	0.02 [-0.17, 0.21]
Le 2005	-0.66	0.5231	76	-0.63	0.5126	73	19.6%	-0.03 [-0.20, 0.14]
Lopez 2005	-0.45	0.8	63	-0.53	0.8	72	7.4%	0.08 [-0.19, 0.35]
McDonald 2015	-0.31	1.6931	585	-0.35	1.6916	584	14.4%	0.04 [-0.15, 0.23]
Rivera 2001	-0.4152	0.7294	161	-0.2392	0.8015	158	19.2%	-0.18 [-0.34, -0.01]
Smuts 2005	-0.23	1.16	49	-0.55	1.05	50	2.9%	0.32 [-0.12, 0.76]
Thu 1999	0.09	0.9	55	-0.01	0.72	54	5.8%	0.10 [-0.21, 0.41]
Untoro 2005	-0.51	0.96	67	-0.57	0.83	65	5.8%	0.06 [-0.25, 0.37]
**Total (95% CI)**			**1386**			**1374**	**100.0%**	**-0.02 [-0.09, 0.06]**

Heterogeneity: Tau² = 0.00; Chi² = 8.38, df = 9 (P = 0.50); I² = 0%
Test for overall effect: Z = 0.48 (P = 0.63)

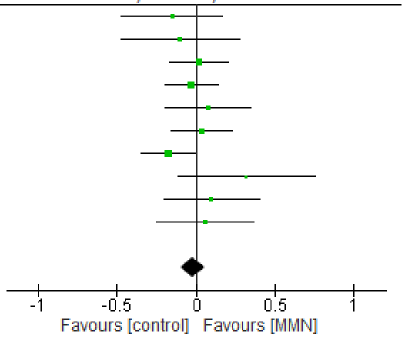

Hemoglobin (g/L)

Study or Subgroup	Mean Difference	SE	Weight	Mean Difference IV, Random, 95% CI
Adu-Afarwuah 2007	6.2	2.0334	6.6%	6.20 [2.21, 10.19]
Baqui 2003	0.8	3.0825	4.1%	0.80 [-5.24, 6.84]
Chang 2010	4	1.938	6.9%	4.00 [0.20, 7.80]
Ekwall 2000	8	2.1524	6.2%	8.00 [3.78, 12.22]
Fahmida 2007	0.66	0.2434	12.2%	0.66 [0.18, 1.14]
Le 2005	7.8	2.6827	4.9%	7.80 [2.54, 13.06]
Lopez 2005	1.1	0.0616	12.3%	1.10 [0.98, 1.22]
McDonald 2015	3	2.7276	4.8%	3.00 [-2.35, 8.35]
Smuts 2005	6.87	0.3578	12.0%	6.87 [6.17, 7.57]
Thu 1999	16	2.2021	6.1%	16.00 [11.68, 20.32]
Tielsch 2006	0.59	0.25	12.2%	0.59 [0.10, 1.08]
Untoro 2005	4.9	0.3778	12.0%	4.90 [4.16, 5.64]
**Total (95% CI)**			**100.0%**	**4.40 [2.91, 5.90]**

Heterogeneity: Tau² = 4.71; Chi² = 426.94, df = 11 (P < 0.00001); I² = 97%
Test for overall effect: Z = 5.78 (P < 0.00001)

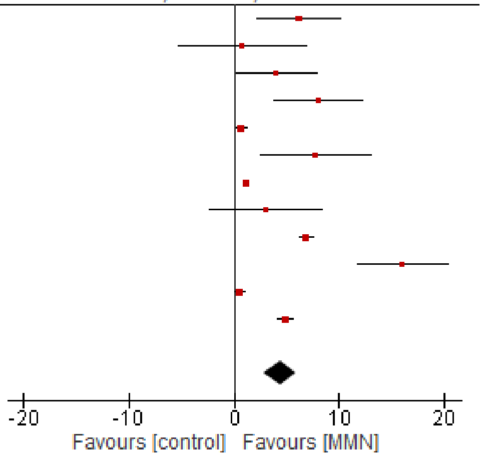

Plasma/serum ferritin (µg/L)

Study or Subgroup	MMN supplementation Mean	SD	Total	Control Mean	SD	Total	Weight	Mean Difference IV, Random, 95% CI
Baqui 2003	18.9	18.2	32	14.6	14.3	37	23.9%	4.30 [-3.51, 12.11]
Fahmida 2007	-15.9	56.24	37	-26.53	32.71	29	10.3%	10.63 [-11.05, 32.31]
Lopez 2005	39.66	54.51	63	10.33	7.35	72	17.1%	29.33 [15.76, 42.90]
McDonald 2015	51.3	43.7094	144	47.8	43.5332	151	21.3%	3.50 [-6.46, 13.46]
Smuts 2005	35.69	28.38	44	22.07	20.2	35	20.3%	13.62 [2.89, 24.35]
Untoro 2005	-30.7	87.95	62	-57.38	69.88	60	7.1%	26.68 [-1.46, 54.82]
**Total (95% CI)**			**382**			**384**	**100.0%**	**12.55 [3.93, 21.17]**

Heterogeneity: Tau² = 65.17; Chi² = 13.27, df = 5 (P = 0.02); I² = 62%
Test for overall effect: Z = 2.85 (P = 0.004)

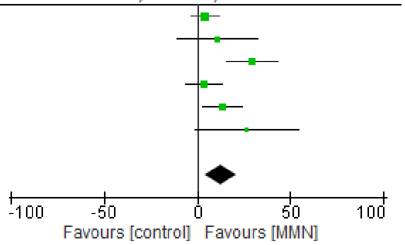

Plasma/serum retinol (µmol/L)

Study or Subgroup	MMN supplementation Mean	SD	Total	Control Mean	SD	Total	Weight	Mean Difference IV, Random, 95% CI
Fahmida 2007	0.11	0.35	42	0.05	0.22	37	7.4%	0.06 [-0.07, 0.19]
Le 2005	0.33	0.4993	45	0.15	0.3947	44	4.1%	0.18 [-0.01, 0.37]
Lopez 2005	0.07	0.1	63	-0.03	0.06	72	22.9%	0.10 [0.07, 0.13]
Smuts 2005	0.06	0.04	44	-0.02	0.05	35	24.2%	0.08 [0.06, 0.10]
Thu 1999	0.25	0.18	53	-0.01	0.13	53	16.7%	0.26 [0.20, 0.32]
Untoro 2005	0.16	0.06	61	0.09	0.02	57	24.7%	0.07 [0.05, 0.09]
**Total (95% CI)**			**308**			**298**	**100.0%**	**0.11 [0.07, 0.16]**

Heterogeneity: Tau² = 0.00; Chi² = 38.78, df = 5 (P < 0.00001); I² = 87%
Test for overall effect: Z = 5.45 (P < 0.00001)

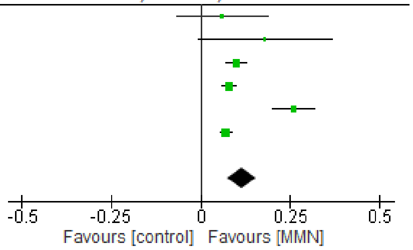

Plasma/serum zinc (µmol/L)

Study or Subgroup	MMN supplementation Mean	SD	Total	Control Mean	SD	Total	Weight	Mean Difference IV, Random, 95% CI
Baqui 2003	11.17	1.68	32	10.55	1.22	38	16.2%	0.62 [-0.08, 1.32]
Chang 2010	0.1	1	91	0.05	1.1	89	18.5%	0.05 [-0.26, 0.36]
Fahmida 2007	1.53	2.39	35	-0.13	2.63	34	12.6%	1.66 [0.47, 2.85]
Lopez 2005	0.6	1.1	63	0.5	0.7	72	18.5%	0.10 [-0.22, 0.42]
Smuts 2005	1.04	0.33	44	-0.31	0.43	35	18.9%	1.35 [1.18, 1.52]
Thu 1999	3.42	3.69	53	-0.09	3.29	53	11.6%	3.51 [2.18, 4.84]
Untoro 2005	-6.28	11.38	62	-5.03	6.78	60	3.8%	-1.25 [-4.56, 2.06]
**Total (95% CI)**			**380**			**381**	**100.0%**	**0.95 [0.23, 1.67]**

Heterogeneity: Tau² = 0.70; Chi² = 98.65, df = 6 (P < 0.00001); I² = 94%
Test for overall effect: Z = 2.59 (P = 0.010)

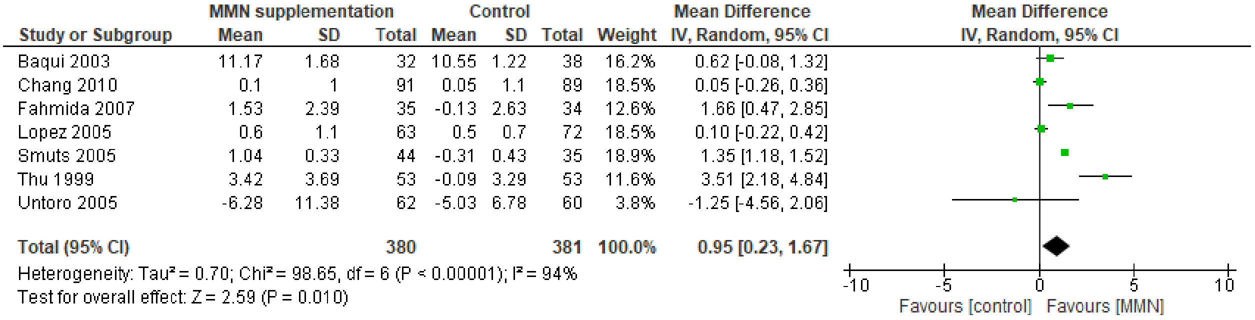

Plasma/serum TfR (log) (mg/L)

Study or Subgroup	MMN supplementation Mean	SD	Total	Control Mean	SD	Total	Weight	Mean Difference IV, Random, 95% CI
Adu-Afarwuah 2007	2.04	0.4297	90	2.36	0.4551	82	25.1%	-0.32 [-0.45, -0.19]
Chang 2010	1.54	0.43	91	1.67	0.49	89	24.8%	-0.13 [-0.26, 0.00]
McDonald 2015	1.8	1.2141	144	1.8	0.6219	151	14.4%	0.00 [-0.22, 0.22]
Untoro 2005	2.17	0.1614	65	2.4	0.2421	65	35.8%	-0.23 [-0.30, -0.16]
**Total (95% CI)**			**390**			**387**	**100.0%**	**-0.19 [-0.30, -0.09]**

Heterogeneity: Tau² = 0.01; Chi² = 7.72, df = 3 (P = 0.05); I² = 61%
Test for overall effect: Z = 3.71 (P = 0.0002)

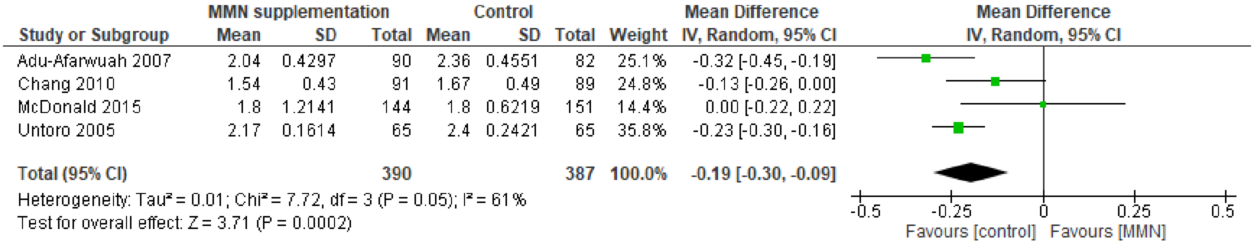

Iron deficiency (plasma/serum ferritin <12µg/L)

Study or Subgroup	log[Risk Ratio]	SE	Weight	Risk Ratio IV, Random, 95% CI
Adu-Afarwuah 2007	-0.8613	0.2288	22.7%	0.42 [0.27, 0.66]
Le 2005	-3.8286	1.4144	2.7%	0.02 [0.00, 0.35]
Lopez 2005 (1)	-0.5756	0.1355	26.1%	0.56 [0.43, 0.73]
McDonald 2015	-0.1196	0.3928	16.4%	0.89 [0.41, 1.92]
Tielsch 2006	-0.7133	0.2676	21.1%	0.49 [0.29, 0.83]
Untoro 2005	-2.4605	0.5744	11.0%	0.09 [0.03, 0.26]
**Total (95% CI)**			**100.0%**	**0.41 [0.25, 0.66]**

Heterogeneity: Tau² = 0.21; Chi² = 17.81, df = 5 (P = 0.003); I² = 72%
Test for overall effect: Z = 3.65 (P = 0.0003)

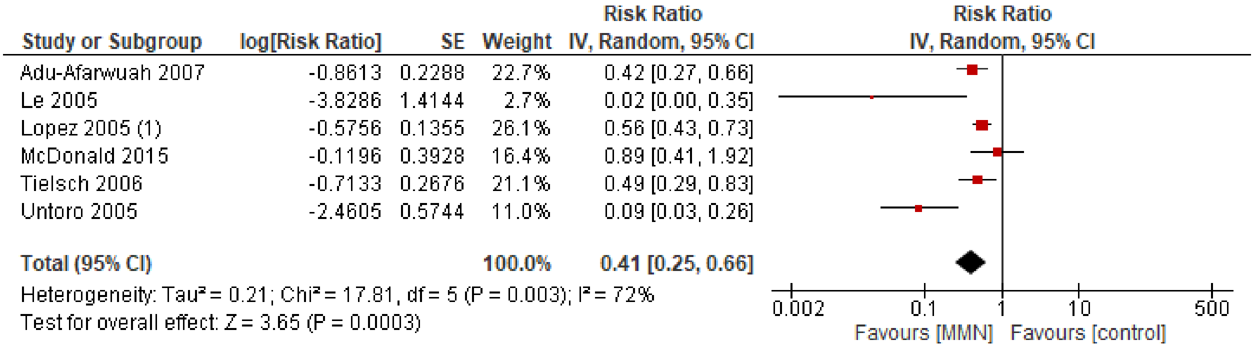

Footnotes
(1) Plasma ferritin <20 ug/L

Vitamin A deficiency (plasma/serum retinol <0.7µmol/L)

Study or Subgroup	MMN supplementation Events	Total	Control Events	Total	Weight	Risk Ratio M-H, Random, 95% CI
Le 2005	2	45	3	44	18.5%	0.65 [0.11, 3.71]
Lopez 2005	6	63	4	72	37.7%	1.71 [0.51, 5.80]
Untoro 2005	5	61	6	57	43.8%	0.78 [0.25, 2.41]
**Total (95% CI)**		**169**		**173**	**100.0%**	**1.01 [0.48, 2.14]**
Total events	13		13			

Heterogeneity: Tau² = 0.00; Chi² = 1.17, df = 2 (P = 0.56); I² = 0%
Test for overall effect: Z = 0.04 (P = 0.97)

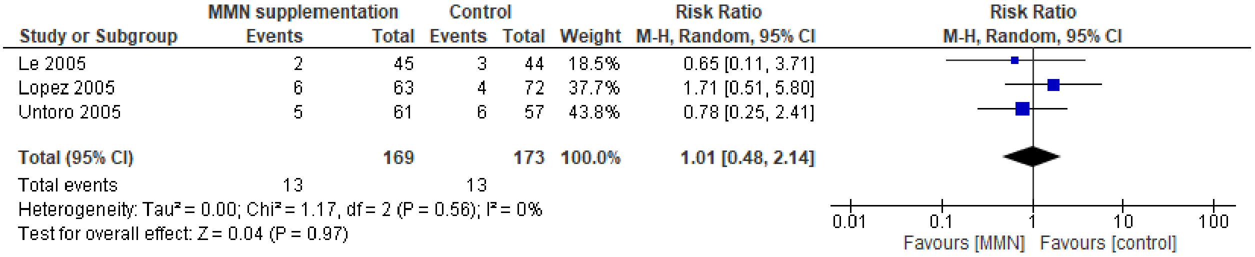

Zinc deficiency (plasma zinc <10.7µmol/L)

Study or Subgroup	MMN supplementation Events	MMN supplementation Total	Control Events	Control Total	Weight	Risk Ratio M-H, Random, 95% CI
Adu-Afarwuah 2007 (1)	41	62	47	69	39.8%	0.97 [0.76, 1.23]
Le 2005	8	44	13	42	24.0%	0.59 [0.27, 1.27]
Lopez 2005	13	63	24	72	29.6%	0.62 [0.35, 1.11]
Untoro 2005	1	62	9	60	6.6%	0.11 [0.01, 0.82]
**Total (95% CI)**		**231**		**243**	**100.0%**	**0.65 [0.37, 1.15]**
Total events	63		93			

Heterogeneity: Tau² = 0.20; Chi² = 9.15, df = 3 (P = 0.03); I² = 67%
Test for overall effect: Z = 1.48 (P = 0.14)

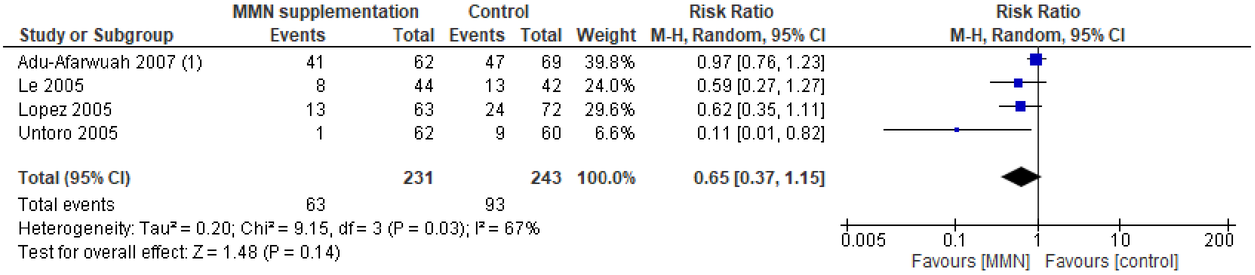

Footnotes
(1) plasma zinc <9.9umol/L

Diarrhea (incidence)

Study or Subgroup	log[Rate Ratio]	SE	Weight	Rate Ratio IV, Random, 95% CI
Baqui 2003	0.1398	0.0612	25.4%	1.15 [1.02, 1.30]
Chang 2010	-0.1382	0.0961	18.1%	0.87 [0.72, 1.05]
McDonald 2015	-0.0513	0.0877	19.7%	0.95 [0.80, 1.13]
Sazawal 2006	-0.0408	0.1468	11.0%	0.96 [0.72, 1.28]
Tielsch 2006	-0.0943	0.0594	25.8%	0.91 [0.81, 1.02]
**Total (95% CI)**			**100.0%**	**0.97 [0.87, 1.09]**

Heterogeneity: Tau² = 0.01; Chi² = 10.03, df = 4 (P = 0.04); I² = 60%
Test for overall effect: Z = 0.48 (P = 0.63)

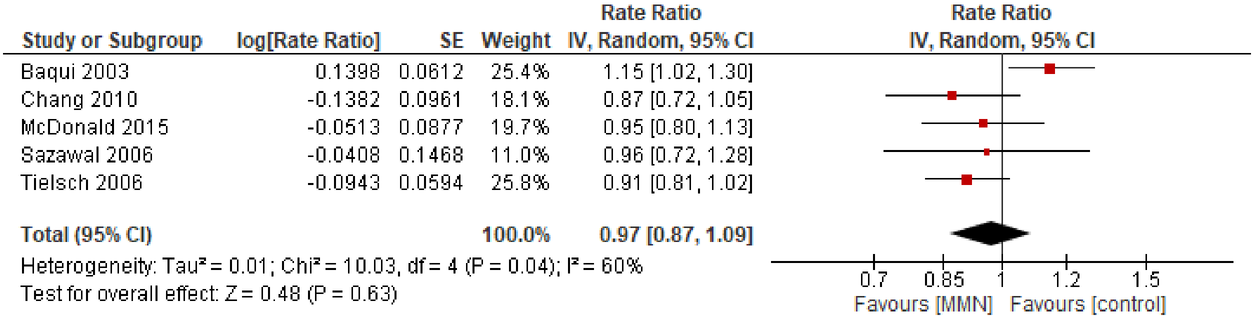

Comparison 5: Subgroup Analyses for MMN Supplementation vs. Placebo/No Intervention (Efficacy)

Anemia: WHO region

Study or Subgroup	MMN supplementation Events	MMN supplementation Total	Control Events	Control Total	Weight	Risk Ratio M-H, Random, 95% CI
**8.1.1 Africa**						
Adu-Afarwuah 2007 (1)	16	102	31	96	9.1%	0.49 [0.28, 0.83]
**Subtotal (95% CI)**		**102**		**96**	**9.1%**	**0.49 [0.28, 0.83]**
Total events	16		31			
Heterogeneity: Not applicable						
Test for overall effect: Z = 2.64 (P = 0.008)						
**8.1.2 Americas**						
Lopez 2005	10	63	32	72	7.5%	0.36 [0.19, 0.67]
**Subtotal (95% CI)**		**63**		**72**	**7.5%**	**0.36 [0.19, 0.67]**
Total events	10		32			
Heterogeneity: Not applicable						
Test for overall effect: Z = 3.23 (P = 0.001)						
**8.1.3 South-east Asia**						
Chang 2010	69	91	77	89	21.2%	0.88 [0.76, 1.01]
Fahmida 2007 (2)	122	152	138	150	22.5%	0.87 [0.80, 0.96]
Tielsch 2006	71	148	94	152	19.0%	0.78 [0.63, 0.96]
Untoro 2005	21	62	34	65	12.0%	0.65 [0.43, 0.98]
**Subtotal (95% CI)**		**453**		**456**	**74.7%**	**0.85 [0.78, 0.92]**
Total events	283		343			
Heterogeneity: Tau² = 0.00; Chi² = 3.72, df = 3 (P = 0.29); I² = 19%						
Test for overall effect: Z = 3.70 (P = 0.0002)						
**8.1.4 Western Pacific**						
Le 2005	12	52	29	56	8.7%	0.45 [0.26, 0.78]
**Subtotal (95% CI)**		**52**		**56**	**8.7%**	**0.45 [0.26, 0.78]**
Total events	12		29			
Heterogeneity: Not applicable						
Test for overall effect: Z = 2.84 (P = 0.004)						
**Total (95% CI)**		**670**		**680**	**100.0%**	**0.69 [0.56, 0.85]**
Total events	321		435			
Heterogeneity: Tau² = 0.05; Chi² = 28.35, df = 6 (P < 0.0001); I² = 79%						
Test for overall effect: Z = 3.53 (P = 0.0004)						
Test for subgroup differences: Chi² = 15.54, df = 3 (P = 0.001), I² = 80.7%						

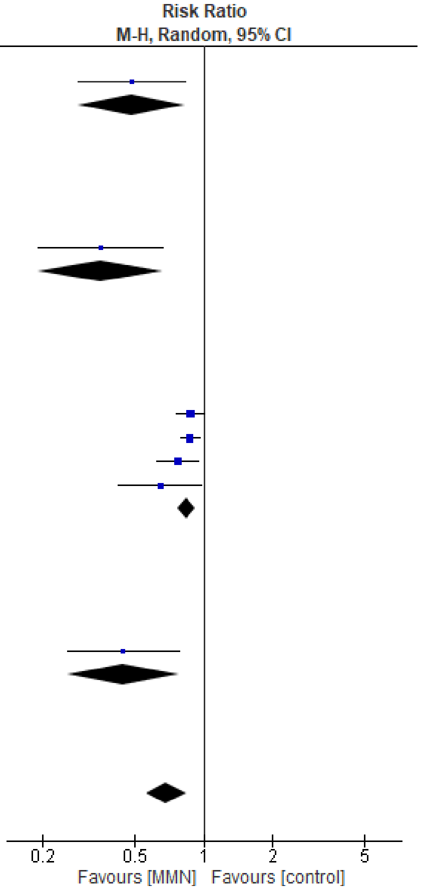

Footnotes
(1) Hemoglobin concentration <100g/L
(2) Hemoglobin concentration <100g/L

Anemia: mean age at baseline

	MMN supplementation		Control			Risk Ratio
**Study or Subgroup**	**Events**	**Total**	**Events**	**Total**	**Weight**	**M-H, Random, 95% CI**
**8.2.1 1-5 months of age**						
Fahmida 2007 (1)	122	152	138	150	22.5%	0.87 [0.80, 0.96]
**Subtotal (95% CI)**		**152**		**150**	**22.5%**	**0.87 [0.80, 0.96]**
Total events	122		138			
Heterogeneity: Not applicable						
Test for overall effect: Z = 2.91 (P = 0.004)						
**8.2.2 6-11 months of age**						
Adu-Afarwuah 2007 (2)	16	102	31	96	9.1%	0.49 [0.28, 0.83]
Chang 2010	69	91	77	89	21.2%	0.88 [0.76, 1.01]
Le 2005	12	52	29	56	8.7%	0.45 [0.26, 0.78]
Lopez 2005	10	63	32	72	7.5%	0.36 [0.19, 0.67]
Tielsch 2006	71	148	94	152	19.0%	0.78 [0.63, 0.96]
Untoro 2005	21	62	34	65	12.0%	0.65 [0.43, 0.98]
**Subtotal (95% CI)**		**518**		**530**	**77.5%**	**0.62 [0.47, 0.82]**
Total events	199		297			
Heterogeneity: Tau² = 0.08; Chi² = 21.35, df = 5 (P = 0.0007); I² = 77%						
Test for overall effect: Z = 3.31 (P = 0.0009)						
**Total (95% CI)**		**670**		**680**	**100.0%**	**0.69 [0.56, 0.85]**
Total events	321		435			
Heterogeneity: Tau² = 0.05; Chi² = 28.35, df = 6 (P < 0.0001); I² = 79%						
Test for overall effect: Z = 3.53 (P = 0.0004)						
Test for subgroup differences: Chi² = 5.02, df = 1 (P = 0.03), I² = 80.1%						

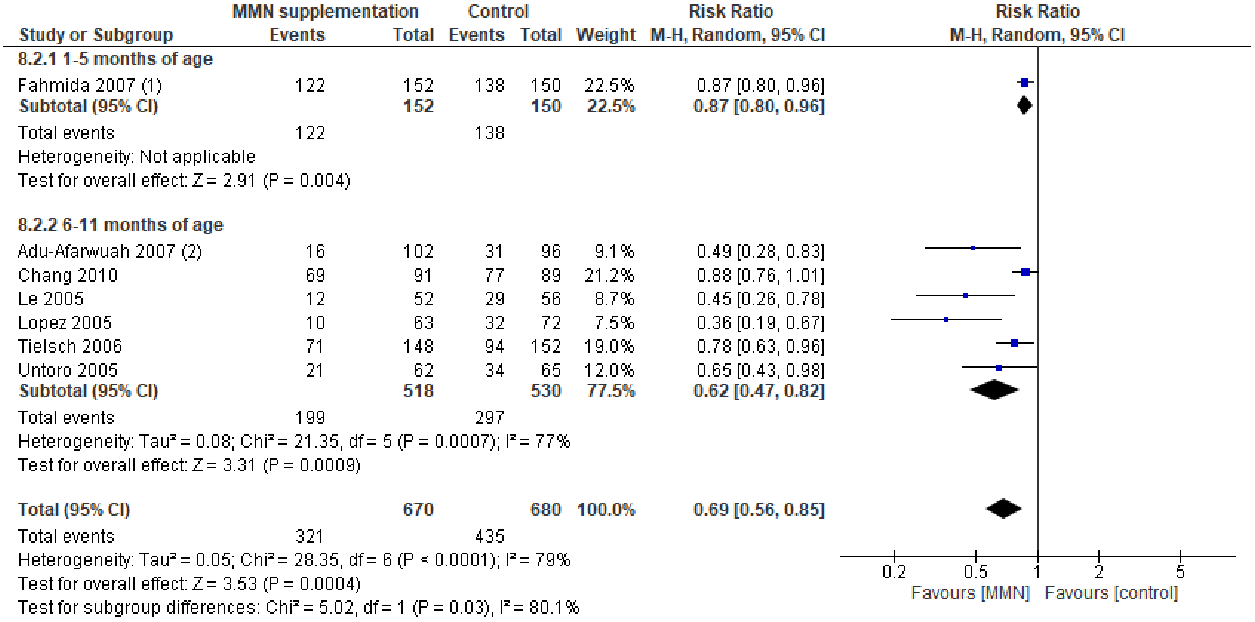

Footnotes
(1) Hemoglobin concentration <100g/L
(2) Hemoglobin concentration <100g/L

Anemia: intervention duration

	MMN supplementation		Control			Risk Ratio
**Study or Subgroup**	**Events**	**Total**	**Events**	**Total**	**Weight**	**M-H, Random, 95% CI**
**8.3.1 3-5 months**						
Untoro 2005	21	62	34	65	15.7%	0.65 [0.43, 0.98]
**Subtotal (95% CI)**		**62**		**65**	**15.7%**	**0.65 [0.43, 0.98]**
Total events	21		34			
Heterogeneity: Not applicable						
Test for overall effect: Z = 2.04 (P = 0.04)						
**8.3.2 6-12 months**						
Adu-Afarwuah 2007 (1)	16	102	31	96	12.6%	0.49 [0.28, 0.83]
Chang 2010	69	91	77	89	24.0%	0.88 [0.76, 1.01]
Fahmida 2007 (2)	122	152	138	150	25.0%	0.87 [0.80, 0.96]
Le 2005	12	52	29	56	12.1%	0.45 [0.26, 0.78]
Lopez 2005	10	63	32	72	10.6%	0.36 [0.19, 0.67]
**Subtotal (95% CI)**		**460**		**463**	**84.3%**	**0.65 [0.48, 0.87]**
Total events	229		307			
Heterogeneity: Tau² = 0.07; Chi² = 27.63, df = 4 (P < 0.0001); I² = 86%						
Test for overall effect: Z = 2.91 (P = 0.004)						
**Total (95% CI)**		**522**		**528**	**100.0%**	**0.65 [0.50, 0.85]**
Total events	250		341			
Heterogeneity: Tau² = 0.07; Chi² = 30.24, df = 5 (P < 0.0001); I² = 83%						
Test for overall effect: Z = 3.19 (P = 0.001)						
Test for subgroup differences: Chi² = 0.00, df = 1 (P = 1.00), I² = 0%						

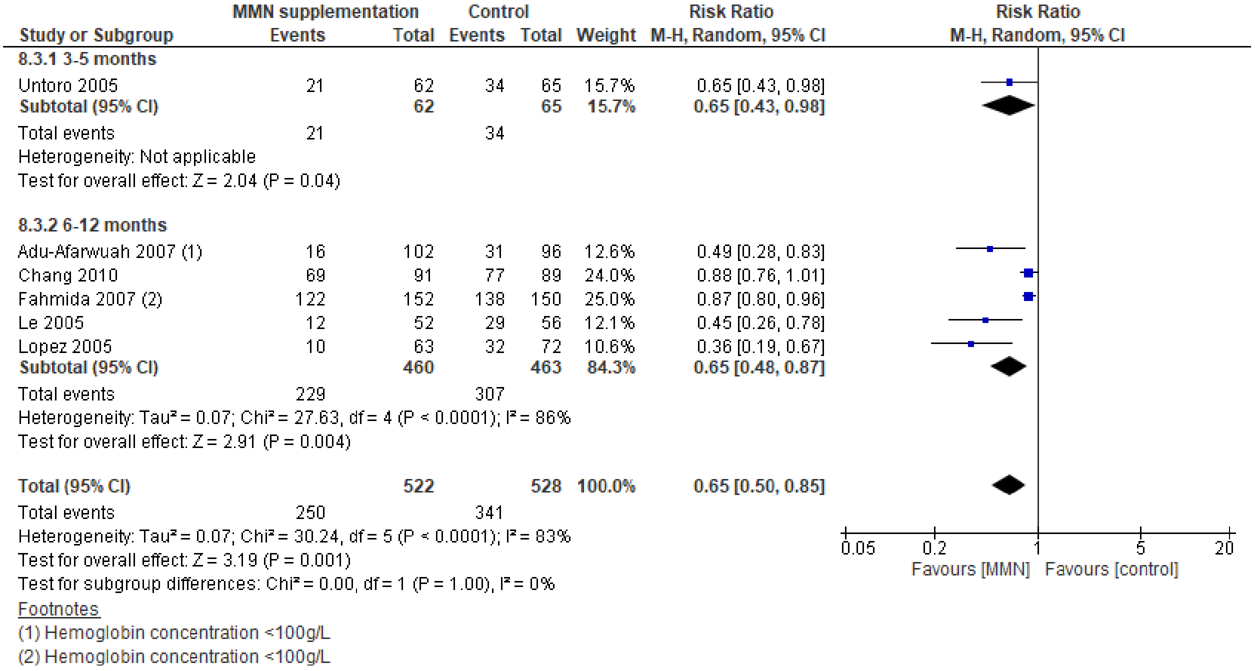

Footnotes
(1) Hemoglobin concentration <100g/L
(2) Hemoglobin concentration <100g/L

Anemia: intervention frequency

Study or Subgroup	MMN supplementation Events	MMN supplementation Total	Control Events	Control Total	Weight	Risk Ratio M-H, Random, 95% CI
**8.4.1 Daily**						
Adu-Afarwuah 2007 (1)	16	102	31	96	7.9%	0.49 [0.28, 0.83]
Fahmida 2007 (2)	122	152	138	150	20.7%	0.87 [0.80, 0.96]
Le 2005	12	52	29	56	7.5%	0.45 [0.26, 0.78]
Lopez 2005	10	63	32	72	6.4%	0.36 [0.19, 0.67]
Tielsch 2006	71	148	94	152	17.2%	0.78 [0.63, 0.96]
Untoro 2005	21	62	34	65	10.5%	0.65 [0.43, 0.98]
**Subtotal (95% CI)**		**579**		**591**	**70.2%**	**0.62 [0.46, 0.83]**
Total events	252		358			
Heterogeneity: Tau² = 0.10; Chi² = 28.51, df = 5 (P < 0.0001); I² = 82%						
Test for overall effect: Z = 3.16 (P = 0.002)						
**8.4.2 Intermittent**						
Chang 2010	69	91	77	89	19.3%	0.88 [0.76, 1.01]
Le 2005	21	55	29	56	10.5%	0.74 [0.48, 1.12]
**Subtotal (95% CI)**		**146**		**145**	**29.8%**	**0.86 [0.75, 0.99]**
Total events	90		106			
Heterogeneity: Tau² = 0.00; Chi² = 0.73, df = 1 (P = 0.39); I² = 0%						
Test for overall effect: Z = 2.18 (P = 0.03)						
**Total (95% CI)**		**725**		**736**	**100.0%**	**0.70 [0.58, 0.84]**
Total events	342		464			
Heterogeneity: Tau² = 0.04; Chi² = 28.44, df = 7 (P = 0.0002); I² = 75%						
Test for overall effect: Z = 3.73 (P = 0.0002)						
Test for subgroup differences: Chi² = 3.97, df = 1 (P = 0.05), I² = 74.8%						

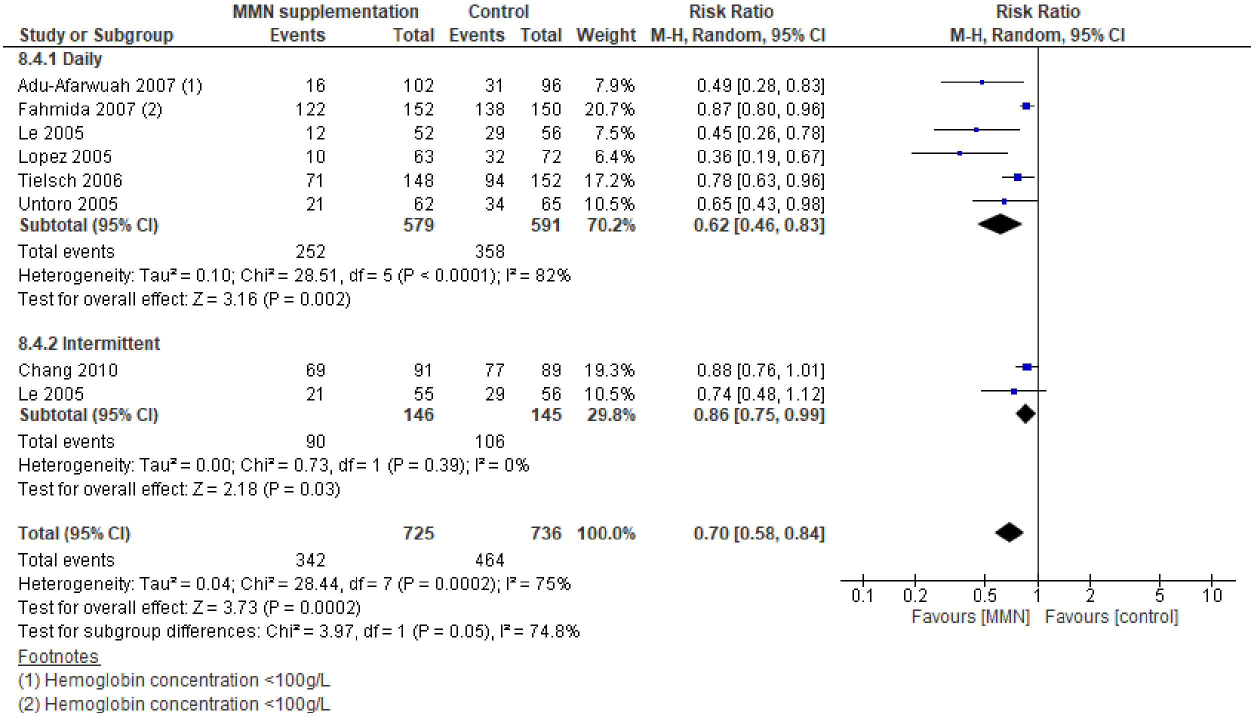

Footnotes
(1) Hemoglobin concentration <100g/L
(2) Hemoglobin concentration <100g/L

Comparison 6: Micronutrient powders vs. Placebo/No Intervention (Efficacy)

Stunting

Study or Subgroup	MNP Events	MNP Total	Control Events	Control Total	Weight	Risk Ratio M-H, Random, 95% CI
Barffour 2019	337	701	317	740	25.6%	1.12 [1.00, 1.26]
Dewey 2017	378	877	336	800	26.0%	1.03 [0.92, 1.15]
Jack 2012	26	145	27	143	2.9%	0.95 [0.58, 1.54]
Kemmer 2012	53	114	50	85	8.4%	0.79 [0.61, 1.03]
Osei 2015	27	50	24	50	4.4%	1.13 [0.77, 1.65]
Soofi 2013	254	458	264	473	25.3%	0.99 [0.89, 1.11]
Vijay 2014	72	119	33	71	7.3%	1.30 [0.98, 1.74]
**Total (95% CI)**		**2464**		**2362**	**100.0%**	**1.04 [0.95, 1.13]**
Total events	1147		1051			
Heterogeneity: Tau² = 0.00; Chi² = 9.13, df = 6 (P = 0.17); I² = 34%						
Test for overall effect: Z = 0.87 (P = 0.38)						

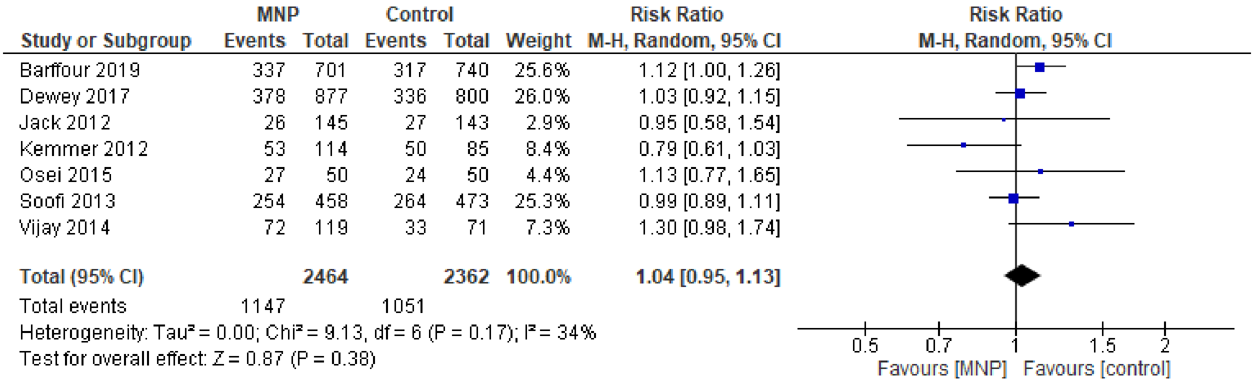

Underweight

Study or Subgroup	MNP Events	MNP Total	Control Events	Control Total	Weight	Risk Ratio M-H, Random, 95% CI
Barffour 2019	197	701	203	740	18.8%	1.02 [0.87, 1.21]
Dewey 2017	367	877	344	800	41.8%	0.97 [0.87, 1.09]
Jack 2012	24	145	23	143	1.9%	1.03 [0.61, 1.74]
Kemmer 2012	17	114	8	85	0.8%	1.58 [0.72, 3.50]
Osei 2015	16	50	17	51	1.7%	0.96 [0.55, 1.68]
Soofi 2013	230	490	216	502	27.4%	1.09 [0.95, 1.25]
Vijay 2014	65	119	40	71	7.6%	0.97 [0.75, 1.26]
**Total (95% CI)**		**2496**		**2392**	**100.0%**	**1.02 [0.95, 1.09]**
Total events	916		851			
Heterogeneity: Tau² = 0.00; Chi² = 2.98, df = 6 (P = 0.81); I² = 0%						
Test for overall effect: Z = 0.50 (P = 0.62)						

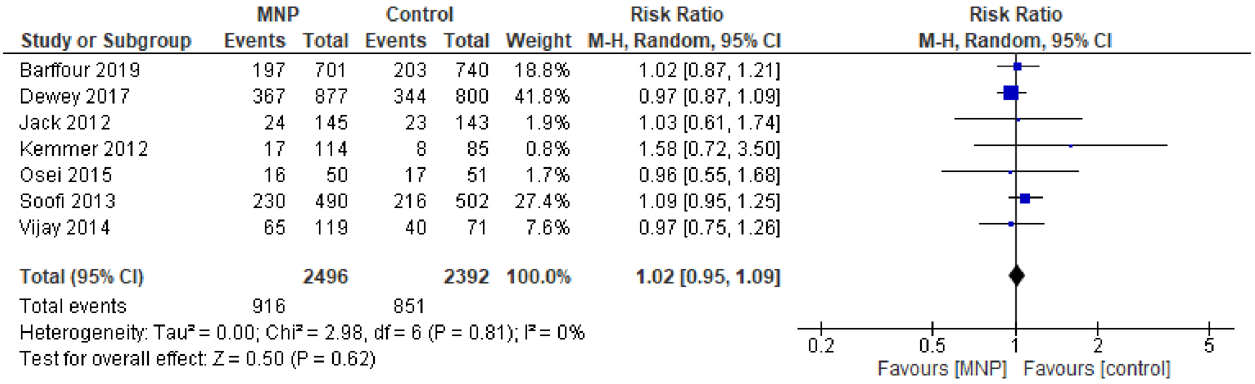

Wasting

Study or Subgroup	MNP Events	MNP Total	Control Events	Control Total	Weight	Risk Ratio M-H, Random, 95% CI
Barffour 2019	39	701	35	740	5.4%	1.18 [0.75, 1.83]
Dewey 2017	340	877	303	800	72.3%	1.02 [0.91, 1.16]
Jack 2012	10	145	11	143	1.6%	0.90 [0.39, 2.05]
Kemmer 2012	0	114	1	85	0.1%	0.25 [0.01, 6.04]
Osei 2015	3	50	6	50	0.6%	0.50 [0.13, 1.89]
Soofi 2013	80	458	77	473	13.1%	1.07 [0.81, 1.43]
Vijay 2014	44	119	25	71	6.9%	1.05 [0.71, 1.56]
**Total (95% CI)**		**2464**		**2362**	**100.0%**	**1.03 [0.93, 1.14]**
Total events	516		458			

Heterogeneity: Tau² = 0.00; Chi² = 2.45, df = 6 (P = 0.87); I² = 0%
Test for overall effect: Z = 0.59 (P = 0.56)

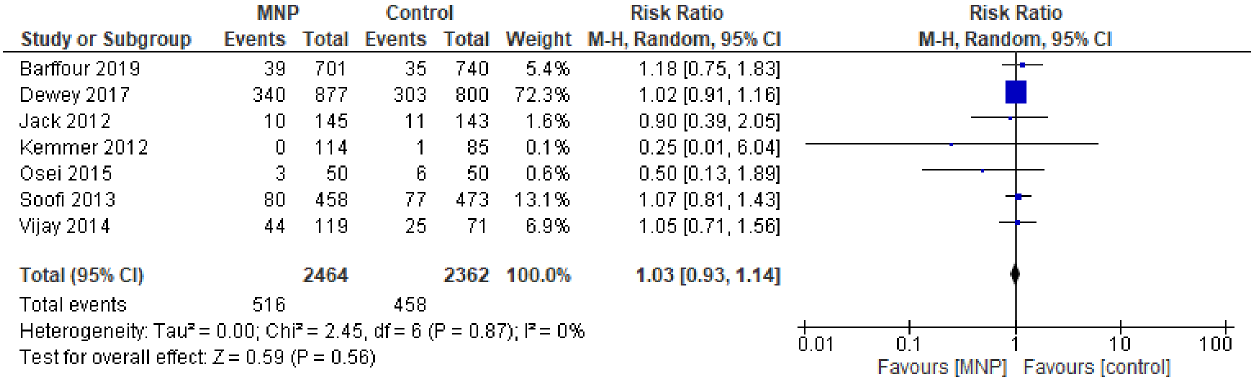

Weight (kg)

Study or Subgroup	MNP Mean	MNP SD	MNP Total	Control Mean	Control SD	Control Total	Weight	Mean Difference IV, Random, 95% CI
Attanasio 2014	14.19	1.8	199	14.04	1.9	205	5.4%	0.15 [-0.21, 0.51]
Barffour 2019	9.58	1.32	701	9.63	1.32	740	37.6%	-0.05 [-0.19, 0.09]
Esamai 2014	8.17	1.0119	10	8.37	1.08	9	0.8%	-0.20 [-1.14, 0.74]
Ogunlade 2011	0.22	0.4368	63	0.34	0.4544	68	30.0%	-0.12 [-0.27, 0.03]
Somasse 2018	0.76	0.75	221	0.74	0.94	183	24.7%	0.02 [-0.15, 0.19]
Vijay 2014	9.9869	2.7761	119	9.8402	1.8138	71	1.6%	0.15 [-0.51, 0.80]
**Total (95% CI)**			**1313**			**1276**	**100.0%**	**-0.04 [-0.12, 0.04]**

Heterogeneity: Tau² = 0.00; Chi² = 3.05, df = 5 (P = 0.69); I² = 0%
Test for overall effect: Z = 0.96 (P = 0.34)

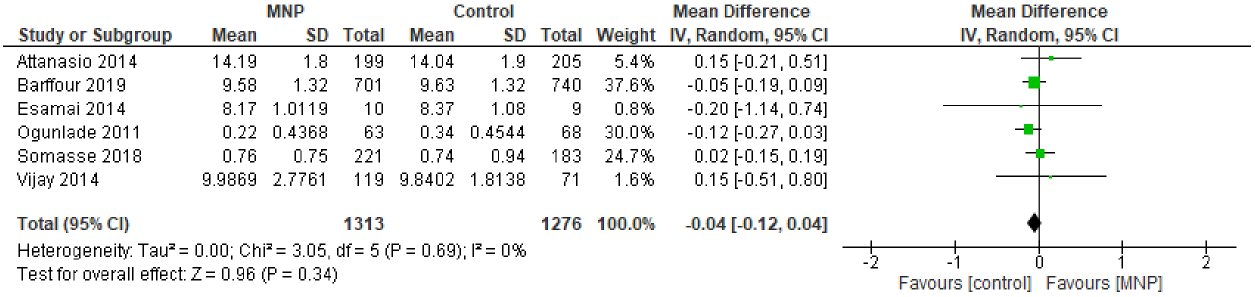

Height (cm)

Study or Subgroup	MNP Mean	MNP SD	MNP Total	Control Mean	Control SD	Control Total	Weight	Mean Difference IV, Random, 95% CI
Attanasio 2014	93.24	4.37	199	92.79	4.2	205	13.7%	0.45 [-0.39, 1.29]
Barffour 2019	79.2	4.8	701	79.3	4.9	740	23.1%	-0.10 [-0.60, 0.40]
Esamai 2014	67.9	3.7947	10	69.5	6	9	0.7%	-1.60 [-6.17, 2.97]
Ogunlade 2011	1.6	0.5162	63	1.65	0.5784	68	34.5%	-0.05 [-0.24, 0.14]
Somasse 2018	2.83	1.85	221	2.1	2.59	183	25.1%	0.73 [0.28, 1.18]
Vijay 2014	82.9706	7.3037	119	82.2764	7.5138	71	2.9%	0.69 [-1.49, 2.88]
**Total (95% CI)**			**1313**			**1276**	**100.0%**	**0.21 [-0.18, 0.60]**

Heterogeneity: Tau² = 0.10; Chi² = 11.99, df = 5 (P = 0.03); I² = 58%
Test for overall effect: Z = 1.07 (P = 0.28)

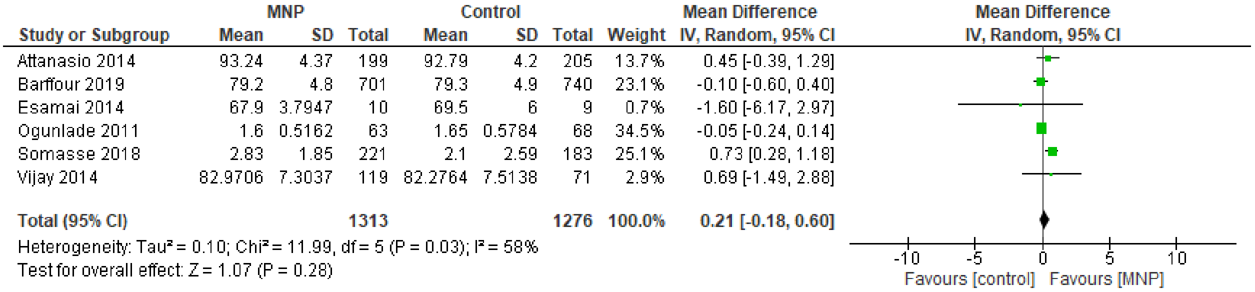

Length-for-age (z-score)

Study or Subgroup	MNP Mean	MNP SD	MNP Total	Control Mean	Control SD	Control Total	Weight	Mean Difference IV, Random, 95% CI
Aboud 2011	-1.89	1.1	85	-1.99	1.1	73	1.0%	0.10 [-0.24, 0.44]
Adu-Afarwuah 2007	-0.4	1	96	-0.4	1	81	1.3%	0.00 [-0.30, 0.30]
Barffour 2019	-1.94	1	701	-1.93	1	740	11.0%	-0.01 [-0.11, 0.09]
Cardoso 2016	-0.02	2.7942	462	-0.27	2.7391	521	1.0%	0.25 [-0.10, 0.60]
Dewey 2017	-1.85	0.95	877	-1.8	0.94	800	14.4%	-0.05 [-0.14, 0.04]
Esamai 2014	-1.16	1.2333	10	-0.21	2.49	9	0.0%	-0.95 [-2.75, 0.85]
Ghosh 2019	-1.177	0.383	163	-1.233	0.3771	158	17.0%	0.06 [-0.03, 0.14]
Giovannini 2006	-1.56	0.7668	65	-1.48	0.7482	62	1.7%	-0.08 [-0.34, 0.18]
Kounnavong 2011	-1	1.49	110	-0.89	1.56	110	0.7%	-0.11 [-0.51, 0.29]
Larson 2018	-1.36	1.2	1009	-1.38	1.2	1015	10.8%	0.02 [-0.08, 0.12]
Ogunlade 2011	0.08	0.1588	63	0.11	0.1653	68	38.2%	-0.03 [-0.09, 0.03]
Osei 2015	-2.03	1.194	50	-2.01	1	50	0.6%	-0.02 [-0.45, 0.41]
Sazawal 2014	-0.22	0.46	29	-0.21	0.47	31	2.1%	-0.01 [-0.25, 0.23]
**Total (95% CI)**			**3720**			**3718**	**100.0%**	**-0.01 [-0.04, 0.03]**

Heterogeneity: Tau² = 0.00; Chi² = 8.08, df = 12 (P = 0.78); I² = 0%
Test for overall effect: Z = 0.43 (P = 0.67)

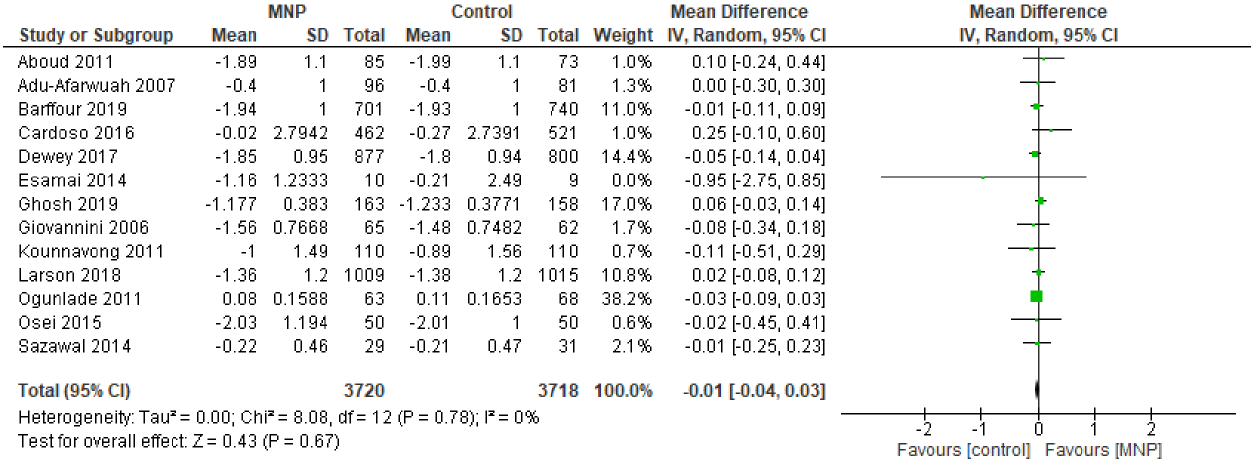

Weight-for-age (z-score)

Study or Subgroup	MNP Mean	MNP SD	MNP Total	Control Mean	Control SD	Control Total	Weight	Mean Difference IV, Random, 95% CI
Aboud 2011	-1.87	1	85	-2.03	1	73	1.9%	0.16 [-0.15, 0.47]
Adu-Afarwuah 2007	-0.53	1.1	96	-0.74	1.1	81	1.8%	0.21 [-0.12, 0.54]
Barffour 2019	-1.51	0.93	701	-1.52	0.9	740	16.9%	0.01 [-0.08, 0.10]
Dewey 2017	-1.77	0.94	877	-1.75	0.93	800	18.4%	-0.02 [-0.11, 0.07]
Esamai 2014	-0.36	1.0119	10	-0.38	1.23	9	0.2%	0.02 [-1.00, 1.04]
Ghosh 2019	-0.899	0.3192	163	-0.994	0.5154	158	17.0%	0.09 [0.00, 0.19]
Giovannini 2006	-1.25	0.6457	65	-1.08	0.6694	62	3.5%	-0.17 [-0.40, 0.06]
Kounnavong 2011	-1.23	0.93	110	-1.27	1.08	110	2.6%	0.04 [-0.23, 0.31]
Larson 2018	-1.32	1.1	1009	-1.4	1.1	1015	16.5%	0.08 [-0.02, 0.18]
Ogunlade 2011	-0.14	0.26	63	-0.09	0.26	68	18.5%	-0.05 [-0.14, 0.04]
Osei 2015	-1.6	0.995	50	-1.57	0.9045	51	1.4%	-0.03 [-0.40, 0.34]
Sazawal 2014	0.65	0.79	29	0.52	0.67	31	1.4%	0.13 [-0.24, 0.50]
**Total (95% CI)**			**3258**			**3198**	**100.0%**	**0.02 [-0.02, 0.07]**

Heterogeneity: Tau² = 0.00; Chi² = 12.28, df = 11 (P = 0.34); I² = 10%
Test for overall effect: Z = 0.96 (P = 0.34)

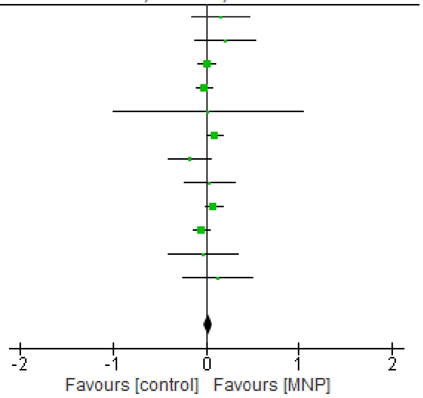

Weight-for-height (z-score)

Study or Subgroup	MNP Mean	MNP SD	MNP Total	Control Mean	Control SD	Control Total	Weight	Mean Difference IV, Random, 95% CI
Adu-Afarwuah 2007	-0.45	1.1	96	-0.74	1.1	81	2.1%	0.29 [-0.04, 0.62]
Barffour 2019	-0.72	0.85	701	-0.74	0.82	740	26.1%	0.02 [-0.07, 0.11]
Dewey 2017	-1.11	0.89	877	-1.11	0.89	800	26.7%	0.00 [-0.09, 0.09]
Ghosh 2019	-0.434	0.3192	163	-0.532	0.817	158	11.4%	0.10 [-0.04, 0.23]
Giovannini 2006	-0.46	0.8071	65	-0.26	0.8269	62	2.8%	-0.20 [-0.48, 0.08]
Kounnavong 2011	-1.09	1.08	110	-1.15	1.04	110	2.8%	0.06 [-0.22, 0.34]
Larson 2018	-0.83	1	1009	-0.91	1	1015	25.7%	0.08 [-0.01, 0.17]
Osei 2015	-0.73	0.995	50	-0.71	1.1	50	1.3%	-0.02 [-0.43, 0.39]
Sazawal 2014	0.91	0.99	29	0.66	0.81	31	1.1%	0.25 [-0.21, 0.71]
**Total (95% CI)**			**3100**			**3047**	**100.0%**	**0.04 [-0.01, 0.09]**

Heterogeneity: Tau² = 0.00; Chi² = 8.46, df = 8 (P = 0.39); I² = 5%
Test for overall effect: Z = 1.72 (P = 0.09)

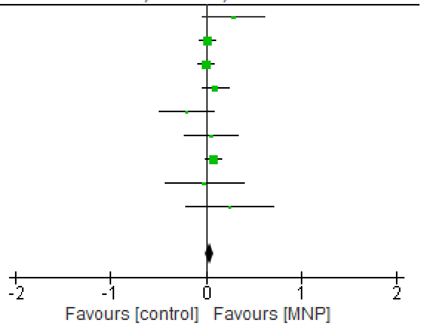

Hemoglobin (g/L)

Study or Subgroup	MNP Mean	MNP SD	MNP Total	Control Mean	Control SD	Control Total	Weight	Mean Difference IV, Random, 95% CI
Adu-Afarwuah 2007	110.1	14.3	98	105.8	14.3	96	1.9%	4.30 [0.28, 8.32]
Arcanjo 2019	119	9	41	122	9.2	39	2.0%	-3.00 [-6.99, 0.99]
Attanasio 2014	120.45	11.87	204	120.94	12.07	207	4.4%	-0.49 [-2.80, 1.82]
Barffour 2019	111.6	0.4	689	110.6	0.4	726	11.3%	1.00 [0.96, 1.04]
Begin 2008 (1)	-0.06	1	37	-0.26	0.88	39	10.7%	0.20 [-0.22, 0.62]
Cardoso 2016	120.8	32.2413	462	117.6	34.2381	521	1.8%	3.20 [-0.96, 7.36]
Chen 2008 (2)	9.93	9.26	94	10.3	7.67	61	3.6%	-0.37 [-3.05, 2.31]
Dewey 2017	115.2	9.6652	252	112.4	13.4033	229	4.9%	2.80 [0.69, 4.91]
Esamai 2014	120	12.65	10	128	14.14	8	0.2%	-8.00 [-20.55, 4.55]
Ghosh 2019	107.012	33.6797	163	108.664	10.2193	158	1.2%	-1.65 [-7.06, 3.76]
Giovannini 2006	107.6	11	65	99.7	10.3	60	2.2%	7.90 [4.17, 11.63]
Jack 2012	105.8	8.8	127	100	10	139	4.5%	5.80 [3.54, 8.06]
Larson 2018	104	14	1009	104	14	1015	7.8%	0.00 [-1.22, 1.22]
Lundeen 2010	7	13.5	243	-1.7	12.7	237	4.3%	8.70 [6.36, 11.04]
Macharia-Mutie 2012	117	9	93	115	11	93	3.2%	2.00 [-0.89, 4.89]
Menon 2007 (3)	104.4	10.1509	72	100.5	17.5311	113	2.0%	3.90 [-0.09, 7.89]
Ogunlade 2011	3.8	9.53	63	5.7	9.089	68	2.8%	-1.90 [-5.09, 1.29]
Osei 2015	18.4	16.9148	50	17.9	19	50	0.7%	0.50 [-6.55, 7.55]
Rim 2008 (4)	118	11.7	107	110	11.1	104	3.0%	8.00 [4.92, 11.08]
Sazawal 2014	3.7	11	36	2.3	13	28	1.0%	1.40 [-4.61, 7.41]
Sharieff 2006	128	10	109	128	9	108	3.9%	0.00 [-2.53, 2.53]
Somasse 2018	0.5	1.22	158	0.09	1.21	152	11.0%	0.41 [0.14, 0.68]
Soofi 2013	101	14.7018	161	92	22.1057	162	1.9%	9.00 [4.91, 13.09]
Suchdev 2012	112	21.026	285	108	20.525	271	2.5%	4.00 [0.55, 7.45]
Varma 2007	128	11	122	124	14	135	3.0%	4.00 [0.94, 7.06]
Zlotkin 2003 (5)	112.5088	9.1256	159	112.8	8.5	80	4.3%	-0.29 [-2.63, 2.05]
**Total (95% CI)**			**4909**			**4899**	**100.0%**	**1.85 [1.24, 2.47]**

Heterogeneity: Tau² = 0.88; Chi² = 170.29, df = 25 (P < 0.00001); I² = 85%
Test for overall effect: Z = 5.89 (P < 0.00001)

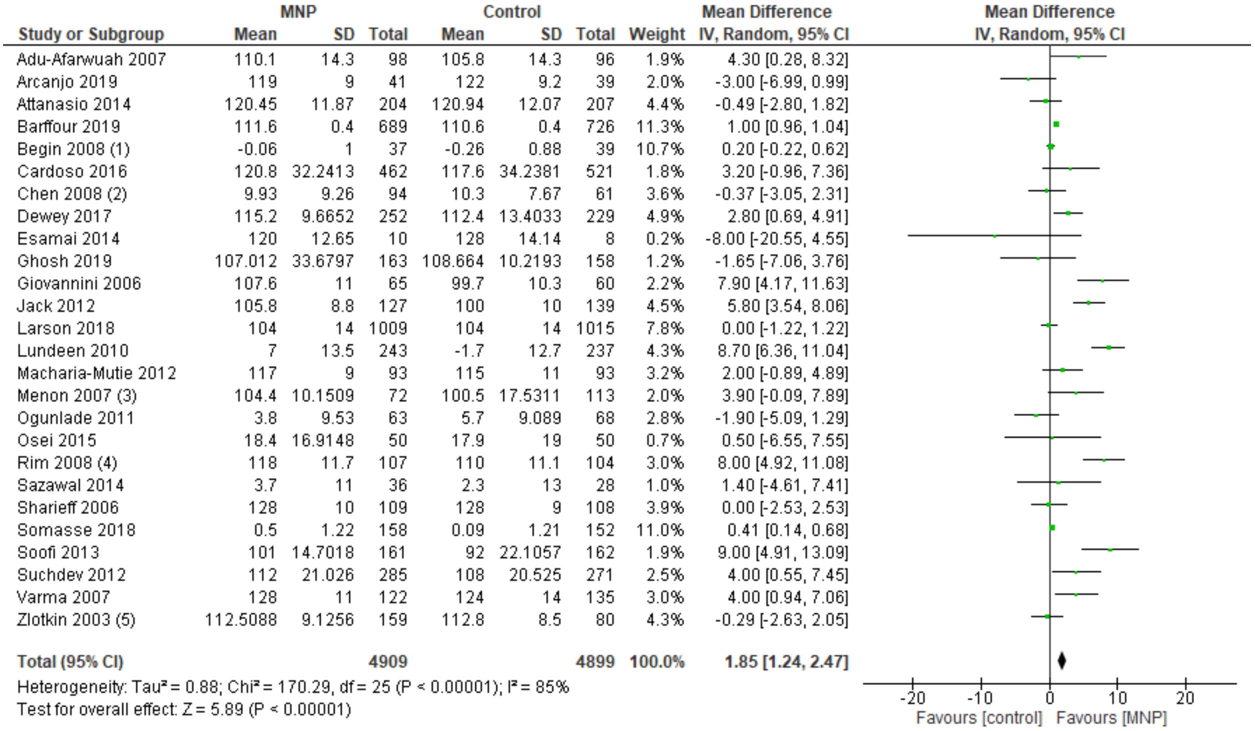

Footnotes
(1) Both groups received whey protein concentrate.
(2) MMN (with vitamin A) versus vitamin A-fortified seasoning powder
(3) Both groups received iron-fortified wheat-soy blend.
(4) MNP contains iron only
(5) MNP contains iron alone or iron + vitamin A

Serum/plasma ferritin (µg/L)

Study or Subgroup	MNP Mean	MNP SD	MNP Total	Control Mean	Control SD	Control Total	Weight	Mean Difference IV, Fixed, 95% CI
Adu-Afarwuah 2007	20.8	26.6482	67	7.9	11.0644	72	0.5%	12.90 [6.03, 19.77]
Begin 2008 (1)	-19.4	32.9	36	-33.9	44.7	33	0.1%	14.50 [-4.16, 33.16]
Cardoso 2016	22.9	15.78	462	22.03	15.41	521	6.5%	0.87 [-1.08, 2.82]
Chen 2008	-9.83	12	94	-3.02	7.51	61	2.6%	-6.81 [-9.88, -3.74]
Dewey 2017	33.4	20.2091	299	22.8	14.241	272	3.0%	10.60 [7.75, 13.45]
Esamai 2014	49.9	35.921	8	23.7	33.3	9	0.0%	26.20 [-6.86, 59.26]
Ghosh 2019	45.457	44.9404	163	39.705	35.3714	158	0.3%	5.75 [-3.08, 14.58]
Giovannini 2006	41.6	30.7	65	14.4	13.5	60	0.4%	27.20 [18.99, 35.41]
Jack 2012	29	21.2	142	19.1	17.3	155	1.3%	9.90 [5.48, 14.32]
Macharia-Mutie 2012	36	32.047	93	25.5	25.2491	93	0.4%	10.50 [2.21, 18.79]
Rim 2008 (2)	40.7	24	96	26.8	21.4	91	0.6%	13.90 [7.39, 20.41]
Sharieff 2006	115.25	44.17	115	110.48	53.35	111	0.2%	4.77 [-8.02, 17.56]
Soofi 2013	16	14.6369	121	9.6	8.6779	101	2.6%	6.40 [3.29, 9.51]
Varma 2007	35.5	2.1	122	22.9	2.4	135	81.6%	12.60 [12.05, 13.15]
Zlotkin 2003 (3)	53.2	56.25	83	72.7	59.5	80	0.1%	-19.50 [-37.29, -1.71]
**Total (95% CI)**			**1966**			**1952**	**100.0%**	**11.08 [10.58, 11.58]**

Heterogeneity: Chi² = 303.92, df = 14 (P < 0.00001); I² = 95%
Test for overall effect: Z = 43.70 (P < 0.00001)

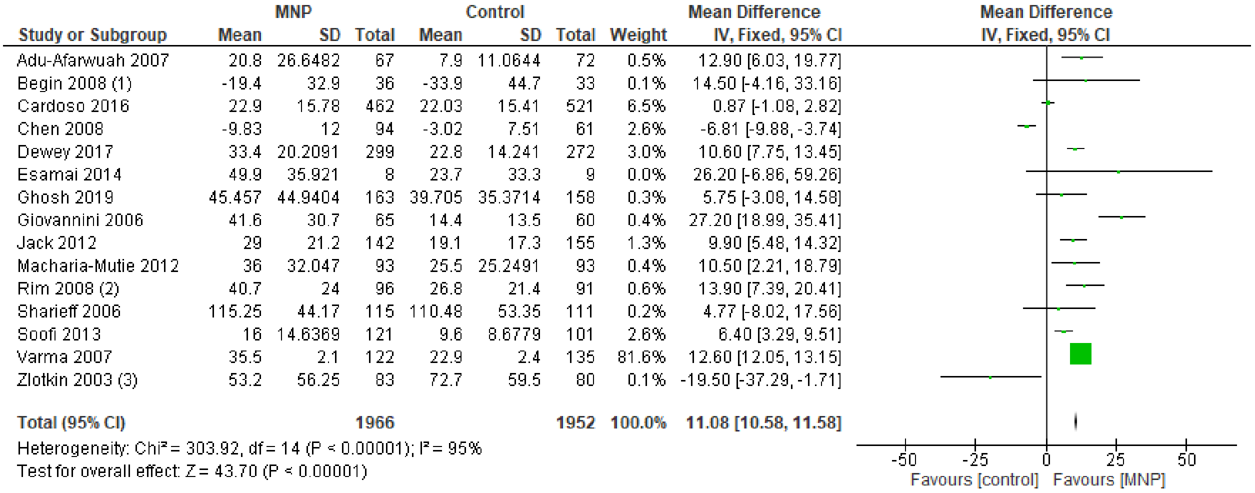

Footnotes
(1) Both groups received whey protein concentrate.
(2) MNP fortified with iron only (intervention)
(3) MNP fortified with iron and vitamin A only

Plasma/serum TfR (mg/L)

Study or Subgroup	MNP Mean	MNP SD	MNP Total	Control Mean	Control SD	Control Total	Weight	Mean Difference IV, Random, 95% CI
Adu-Afarwuah 2007	8.3	3.3989	93	10.6	4.5512	82	11.5%	-2.30 [-3.50, -1.10]
Cardoso 2016	3.04	3.1	462	4.27	5.24	521	18.2%	-1.23 [-1.76, -0.70]
Dewey 2017	8.5	2.636	299	9.4	3.3508	272	18.6%	-0.90 [-1.40, -0.40]
Esamai 2014	2.8	0.9487	10	4	1.1314	8	13.6%	-1.20 [-2.18, -0.22]
Kemmer 2012	7.19	1.64	114	7.02	1.18	85	19.5%	0.17 [-0.22, 0.56]
Macharia-Mutie 2012	4.4	1.4567	93	4.8	1.9422	93	18.6%	-0.40 [-0.89, 0.09]
**Total (95% CI)**			**1071**			**1061**	**100.0%**	**-0.86 [-1.46, -0.26]**

Heterogeneity: Tau² = 0.44; Chi² = 31.30, df = 5 (P < 0.00001); I² = 84%
Test for overall effect: Z = 2.80 (P = 0.005)

Mean Difference IV, Random, 95% CI

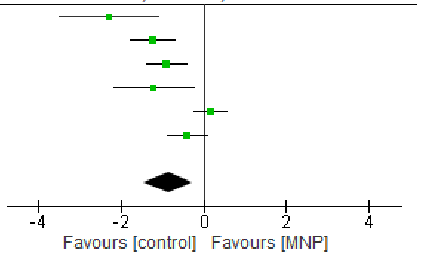

Serum/plasma zinc (μg/dL)

Study or Subgroup	MNP Mean	MNP SD	MNP Total	Control Mean	Control SD	Control Total	Weight	Mean Difference IV, Random, 95% CI
Adu-Afarwuah 2007	60.8	16.345	56	62.11	16.345	69	10.4%	-1.31 [-7.07, 4.45]
Begin 2008 (1)	-0.6	20.1	29	-2	24.2	26	2.5%	1.40 [-10.43, 13.23]
Esamai 2014	70.2	15.8392	8	69.8	16.2	9	1.5%	0.40 [-14.85, 15.65]
Ghosh 2019	57.7	8.5913	61	58.2	7.8102	61	40.8%	-0.50 [-3.41, 2.41]
Jack 2012	66	15.7	142	62.7	11.1	155	35.7%	3.30 [0.18, 6.42]
Soofi 2013	67.1	28.9035	156	69.4	26.4678	153	9.1%	-2.30 [-8.48, 3.88]
**Total (95% CI)**			**452**			**473**	**100.0%**	**0.67 [-1.19, 2.53]**

Heterogeneity: Tau² = 0.00; Chi² = 4.71, df = 5 (P = 0.45); I² = 0%
Test for overall effect: Z = 0.70 (P = 0.48)

Mean Difference IV, Random, 95% CI

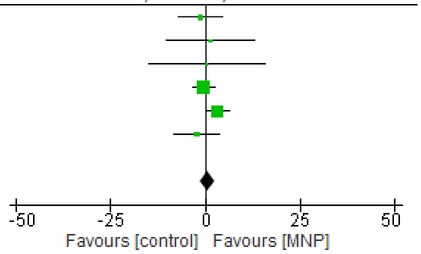

Footnotes
(1) Both groups received whey protein concentrate.

Serum retinol (μg/dL)

Study or Subgroup	MNP Mean	MNP SD	MNP Total	Control Mean	Control SD	Control Total	Weight	Mean Difference IV, Random, 95% CI
Begin 2008 (1)	5.8	8.1	29	6.4	11.1	25	18.1%	-0.60 [-5.86, 4.66]
Cardoso 2016	45.85	51.5155	399	40.11	56.9407	395	13.1%	5.74 [-1.82, 13.30]
Chen 2008	5.92	11.88	94	1.05	7.42	61	23.7%	4.87 [1.83, 7.91]
Soofi 2013	27.22	18.0285	146	31.81	16.3269	151	21.5%	-4.59 [-8.51, -0.67]
Varma 2007	39.2	11.2	122	39.2	14	135	23.6%	0.00 [-3.09, 3.09]
**Total (95% CI)**			**790**			**767**	**100.0%**	**0.81 [-2.94, 4.56]**

Heterogeneity: Tau² = 13.04; Chi² = 16.32, df = 4 (P = 0.003); I² = 75%
Test for overall effect: Z = 0.42 (P = 0.67)

Mean Difference IV, Random, 95% CI

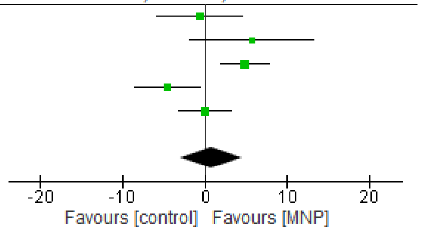

Footnotes
(1) Both groups received whey protein concentrate.

Iron deficiency

Study or Subgroup	MNP Events	MNP Total	Control Events	Control Total	Weight	Risk Ratio M-H, Random, 95% CI
Adu-Afarwuah 2007	24	87	39	72	9.5%	0.51 [0.34, 0.76]
Barffour 2019	16	138	40	140	7.8%	0.41 [0.24, 0.69]
Begin 2008 (1)	10	36	17	33	6.8%	0.54 [0.29, 1.00]
Cardoso 2016	128	462	188	521	12.1%	0.77 [0.64, 0.93]
Dewey 2017	144	299	149	272	12.4%	0.88 [0.75, 1.03]
Giovannini 2006	9	65	31	60	6.5%	0.27 [0.14, 0.52]
Jack 2012	13	70	34	79	7.6%	0.43 [0.25, 0.75]
Macharia-Mutie 2012	5	93	16	93	4.1%	0.31 [0.12, 0.82]
Rim 2008 (2)	6	96	26	91	4.8%	0.22 [0.09, 0.51]
Soofi 2013	42	121	65	101	11.0%	0.54 [0.41, 0.72]
Suchdev 2012	38	169	63	164	10.3%	0.59 [0.42, 0.82]
Varma 2007	13	122	41	135	7.3%	0.35 [0.20, 0.62]
**Total (95% CI)**		**1758**		**1761**	**100.0%**	**0.50 [0.40, 0.63]**
Total events	448		709			

Heterogeneity: Tau² = 0.11; Chi² = 47.59, df = 11 (P < 0.00001); I² = 77%
Test for overall effect: Z = 5.83 (P < 0.00001)

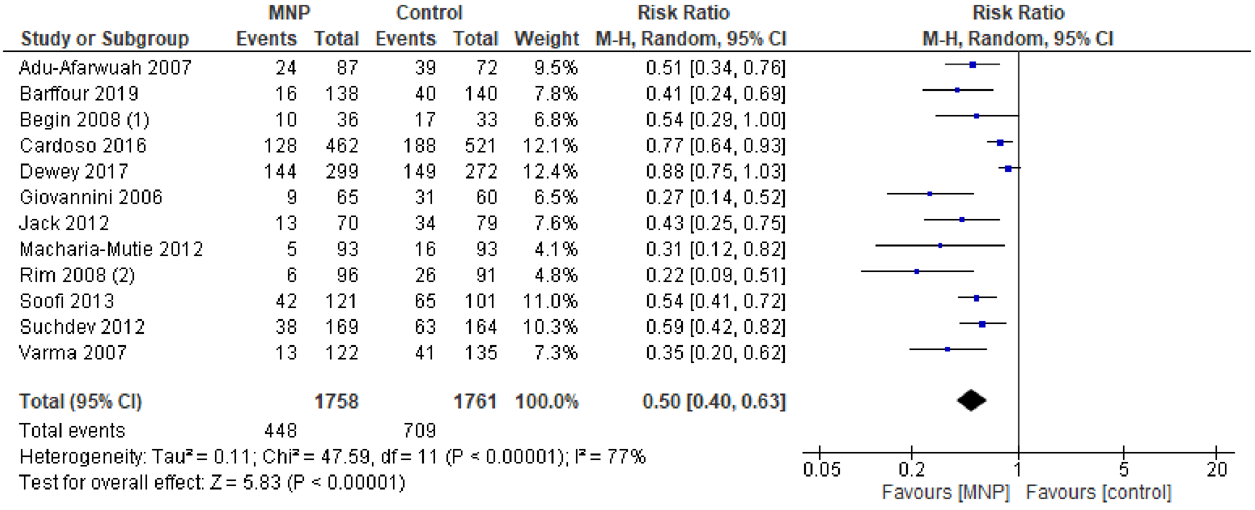

Footnotes
(1) Both groups received whey protein concentrate.
(2) MNP contains iron only

Iron-deficiency anemia

Study or Subgroup	MNP Events	MNP Total	Control Events	Control Total	Weight	Risk Ratio M-H, Random, 95% CI
Adu-Afarwuah 2007	15	94	25	82	16.1%	0.52 [0.30, 0.92]
Cardoso 2016	21	462	52	521	19.8%	0.46 [0.28, 0.74]
Dewey 2017	19	117	31	106	19.0%	0.56 [0.33, 0.92]
Macharia-Mutie 2012	4	93	11	93	5.2%	0.36 [0.12, 1.10]
Rim 2008	3	96	20	91	4.6%	0.14 [0.04, 0.46]
Soofi 2013	38	142	71	126	33.7%	0.47 [0.35, 0.65]
Varma 2007	1	122	13	135	1.6%	0.09 [0.01, 0.64]
**Total (95% CI)**		**1126**		**1154**	**100.0%**	**0.45 [0.34, 0.58]**
Total events	101		223			

Heterogeneity: Tau² = 0.03; Chi² = 7.79, df = 6 (P = 0.25); I² = 23%
Test for overall effect: Z = 6.01 (P < 0.00001)

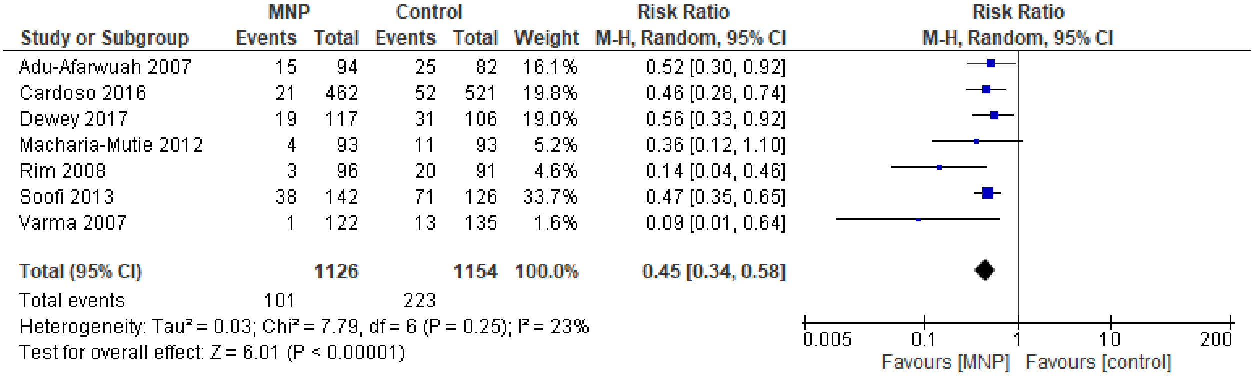

Zinc deficiency

Study or Subgroup	MNP Events	MNP Total	Control Events	Control Total	Weight	Risk Ratio M-H, Random, 95% CI
Adu-Afarwuah 2007	43	56	47	69	23.3%	1.13 [0.91, 1.40]
Barffour 2019	81	138	111	140	26.4%	0.74 [0.63, 0.87]
Begin 2008 (1)	11	29	12	26	7.8%	0.82 [0.44, 1.53]
Jack 2012	54	98	68	110	22.4%	0.89 [0.71, 1.12]
Soofi 2013	67	156	58	153	20.1%	1.13 [0.86, 1.49]
**Total (95% CI)**		**477**		**498**	**100.0%**	**0.93 [0.77, 1.14]**
Total events	256		296			

Heterogeneity: Tau² = 0.03; Chi² = 12.55, df = 4 (P = 0.01); I² = 68%
Test for overall effect: Z = 0.66 (P = 0.51)

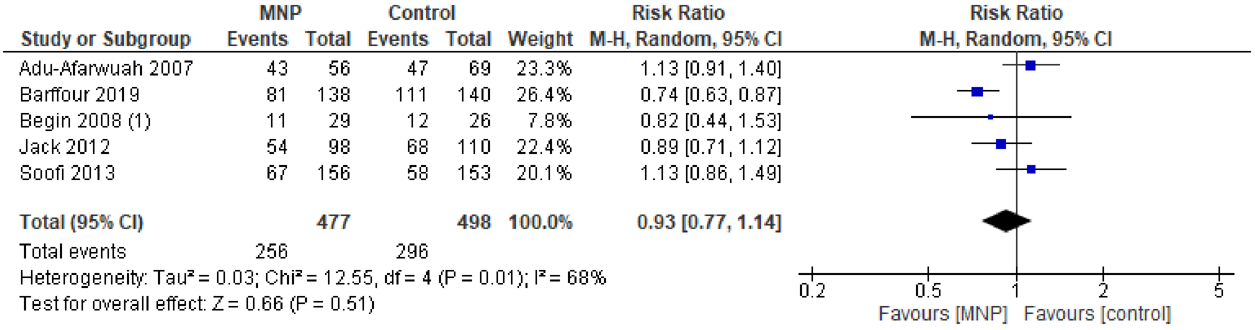

Footnotes
(1) Both groups received whey protein concentrate.

Vitamin A deficiency

Study or Subgroup	log[Risk Ratio]	SE	Weight	Risk Ratio IV, Random, 95% CI
Barffour 2019	0.5534	0.4602	14.6%	1.74 [0.71, 4.29]
Begin 2008	0.7679	0.7909	8.7%	2.16 [0.46, 10.16]
Cardoso 2016	-0.8377	0.1945	20.4%	0.43 [0.30, 0.63]
Jack 2012	-0.2614	1.6915	2.7%	0.77 [0.03, 21.20]
Soofi 2013	0.4746	0.1903	20.4%	1.61 [1.11, 2.33]
Suchdev 2012	-0.4115	0.2919	18.4%	0.66 [0.37, 1.17]
Varma 2007	0.2601	0.4494	14.8%	1.30 [0.54, 3.13]
**Total (95% CI)**			**100.0%**	**1.03 [0.57, 1.85]**

Heterogeneity: $Tau^2 = 0.40$; $Chi^2 = 28.50$, df = 6 (P < 0.0001); $I^2 = 79\%$
Test for overall effect: Z = 0.10 (P = 0.92)

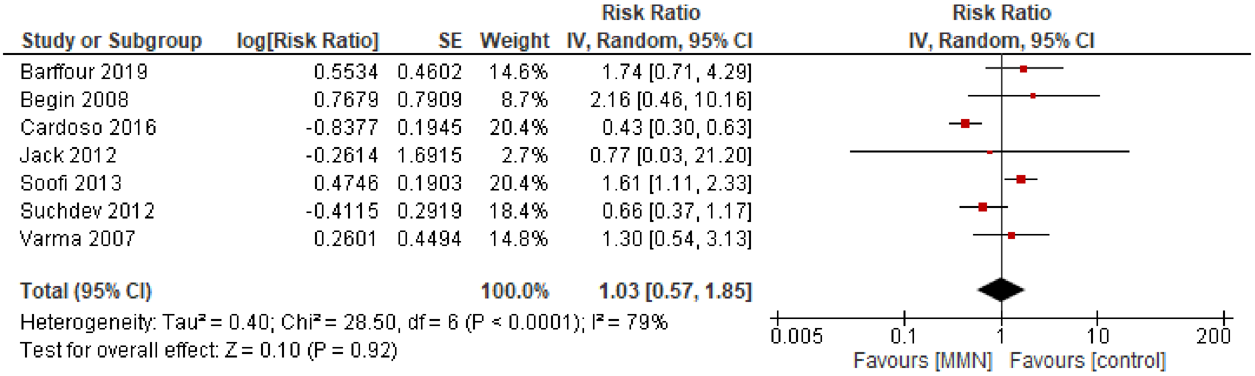

Diarrhea (prevalence)

Study or Subgroup	log[Risk Ratio]	SE	Weight	Risk Ratio IV, Random, 95% CI
Begin 2008	0.3646	0.5746	2.1%	1.44 [0.47, 4.44]
Osei 2015	0.0189	1.171	0.5%	1.02 [0.10, 10.11]
Somasse 2018	0.091	0.3908	4.5%	1.10 [0.51, 2.36]
Soofi 2013	0.27	0.086	92.9%	1.31 [1.11, 1.55]
**Total (95% CI)**			**100.0%**	**1.30 [1.11, 1.53]**

Heterogeneity: $Tau^2 = 0.00$; $Chi^2 = 0.28$, df = 3 (P = 0.96); $I^2 = 0\%$
Test for overall effect: Z = 3.17 (P = 0.002)

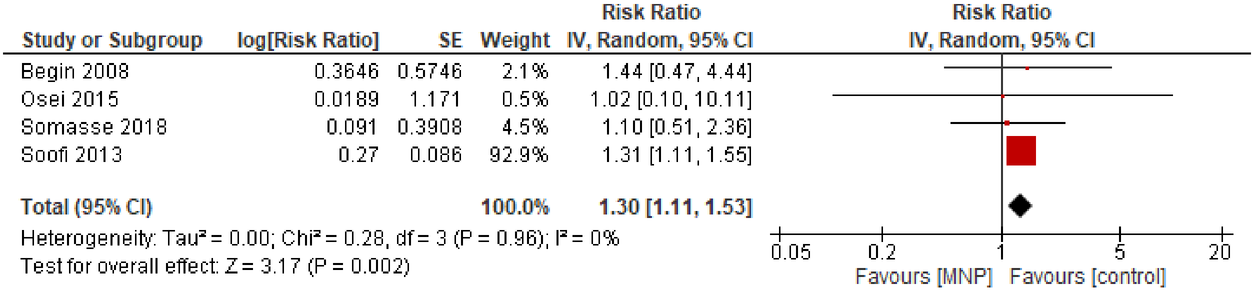

Cognitive function

Study or Subgroup	log[Odds Ratio]	SE	Weight	Odds Ratio IV, Random, 95% CI
Attanasio 2014	0.196	0.3113	70.8%	1.22 [0.66, 2.24]
Luo 2017	0.828	1.5375	14.4%	2.29 [0.11, 46.59]
Ogunlade 2011	2.73	1.5204	14.7%	15.33 [0.78, 301.85]
**Total (95% CI)**			**100.0%**	**1.94 [0.55, 6.80]**

Heterogeneity: $Tau^2 = 0.48$; $Chi^2 = 2.78$, df = 2 (P = 0.25); $I^2 = 28\%$
Test for overall effect: Z = 1.03 (P = 0.30)

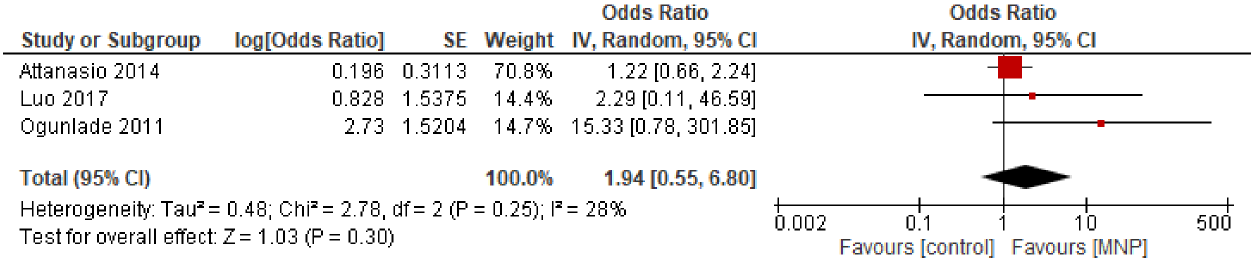

Comparison 6: Subgroup Analyses for Micronutrient Powders vs. Placebo/No Intervention (Efficacy)

Anemia: WHO region

Study or Subgroup	log[Risk Ratio]	SE	Weight	Risk Ratio IV, Random, 95% CI
**2.6.1 Africa**				
Adu-Afarwuah 2007	-0.5642	0.2592	2.8%	0.57 [0.34, 0.95]
Dewey 2017	-0.3077	0.2113	3.7%	0.74 [0.49, 1.11]
Macharia-Mutie 2012	-0.5108	0.2596	2.8%	0.60 [0.36, 1.00]
Somasse 2018	-0.0136	0.0716	8.4%	0.99 [0.86, 1.14]
Suchdev 2012	-0.1839	0.0977	7.3%	0.83 [0.69, 1.01]
Varma 2007	-1.6215	0.4692	1.0%	0.20 [0.08, 0.50]
Zlotkin 2003 (1)	0.5386	0.3861	1.5%	1.71 [0.80, 3.65]
**Subtotal (95% CI)**			**27.4%**	**0.75 [0.58, 0.98]**
Heterogeneity: Tau² = 0.07; Chi² = 21.71, df = 6 (P = 0.001); I² = 72%				
Test for overall effect: Z = 2.14 (P = 0.03)				
**2.6.2 Americas**				
Arcanjo 2019	0.1616	0.8236	0.4%	1.18 [0.23, 5.91]
Attanasio 2014	-0.2792	0.1812	4.4%	0.76 [0.53, 1.08]
Begin 2008 (2)	-0.507	0.3792	1.5%	0.60 [0.29, 1.27]
Cardoso 2016	-0.4777	0.1393	5.7%	0.62 [0.47, 0.81]
Menon 2007 (3)	-0.6079	0.2377	3.1%	0.54 [0.34, 0.87]
**Subtotal (95% CI)**			**15.1%**	**0.65 [0.54, 0.78]**
Heterogeneity: Tau² = 0.00; Chi² = 1.92, df = 4 (P = 0.75); I² = 0%				
Test for overall effect: Z = 4.54 (P < 0.00001)				
**2.6.4 Eastern Mediterranean**				
Soofi 2013	-0.2442	0.0543	9.0%	0.78 [0.70, 0.87]
**Subtotal (95% CI)**			**9.0%**	**0.78 [0.70, 0.87]**
Heterogeneity: Not applicable				
Test for overall effect: Z = 4.50 (P < 0.00001)				
**2.6.5 Europe**				
Lundeen 2010	-0.3626	0.0718	8.3%	0.70 [0.60, 0.80]
**Subtotal (95% CI)**			**8.3%**	**0.70 [0.60, 0.80]**
Heterogeneity: Not applicable				
Test for overall effect: Z = 5.05 (P < 0.00001)				
**2.6.6 South-East Asia**				
Larson 2018	-0.0198	0.0324	9.6%	0.98 [0.92, 1.04]
Osei 2015	0.2231	0.4301	1.2%	1.25 [0.54, 2.90]
Rim 2008 (4)	-0.6824	0.1987	4.0%	0.51 [0.34, 0.75]
**Subtotal (95% CI)**			**14.8%**	**0.81 [0.49, 1.36]**
Heterogeneity: Tau² = 0.15; Chi² = 11.20, df = 2 (P = 0.004); I² = 82%				
Test for overall effect: Z = 0.79 (P = 0.43)				
**2.6.7 Western Pacific**				
Barffour 2019	-0.0808	0.0634	8.7%	0.92 [0.81, 1.04]
Giovannini 2006	-0.6224	0.1766	4.5%	0.54 [0.38, 0.76]
Jack 2012	-0.2395	0.0388	9.5%	0.79 [0.73, 0.85]
Kounnavong 2011	-0.2624	0.2651	2.7%	0.77 [0.46, 1.29]
**Subtotal (95% CI)**			**25.3%**	**0.78 [0.66, 0.93]**
Heterogeneity: Tau² = 0.02; Chi² = 10.22, df = 3 (P = 0.02); I² = 71%				
Test for overall effect: Z = 2.84 (P = 0.004)				
**Total (95% CI)**			**100.0%**	**0.76 [0.69, 0.84]**
Heterogeneity: Tau² = 0.03; Chi² = 81.62, df = 20 (P < 0.00001); I² = 75%				
Test for overall effect: Z = 5.39 (P < 0.00001)				
Test for subgroup differences: Chi² = 4.40, df = 5 (P = 0.49), I² = 0%				

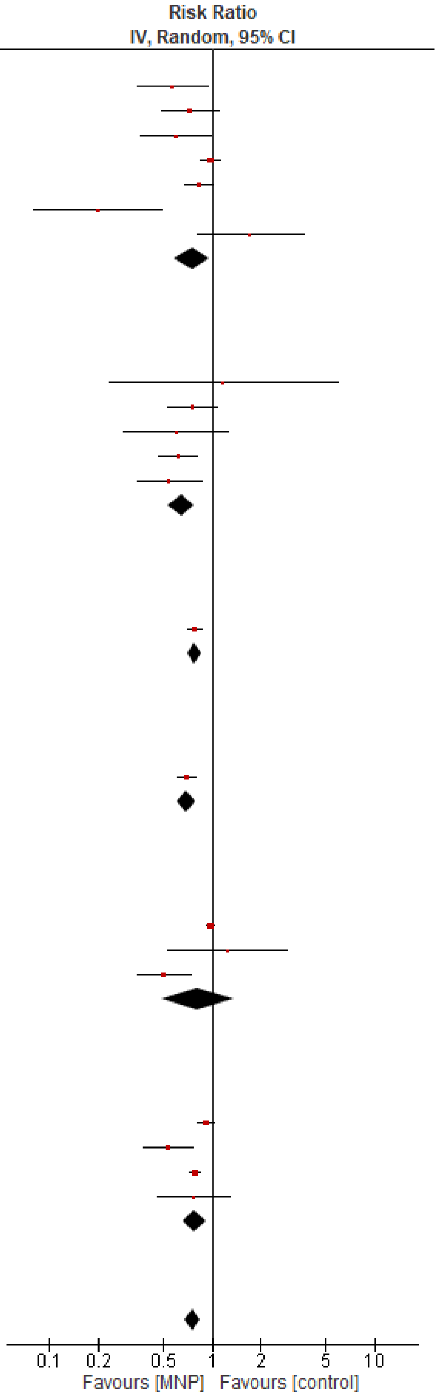

Footnotes
(1) MNP contains iron & vitamin A only
(2) Both groups received why protein concentrate
(3) Both groups received iron fortified wheat soy blend
(4) MNP contains iron only

Anemia: mean age at baseline

Study or Subgroup	log[Risk Ratio]	SE	Weight	Risk Ratio IV, Random, 95% CI
**2.7.1 6-11 months**				
Adu-Afarwuah 2007	-0.5642	0.2592	2.8%	0.57 [0.34, 0.95]
Begin 2008 (1)	-0.507	0.3792	1.5%	0.60 [0.29, 1.27]
Cardoso 2016	-0.4777	0.1393	6.2%	0.62 [0.47, 0.81]
Dewey 2017	-0.3077	0.2113	3.8%	0.74 [0.49, 1.11]
Giovannini 2006	-0.6224	0.1766	4.8%	0.54 [0.38, 0.76]
Jack 2012	-0.2395	0.0388	11.3%	0.79 [0.73, 0.85]
Osei 2015	0.2231	0.4301	1.2%	1.25 [0.54, 2.90]
Rim 2008 (2)	-0.6824	0.1987	4.1%	0.51 [0.34, 0.75]
Soofi 2013	-0.2442	0.0543	10.6%	0.78 [0.70, 0.87]
**Subtotal (95% CI)**			**46.2%**	**0.71 [0.63, 0.79]**
Heterogeneity: Tau² = 0.01; Chi² = 14.33, df = 8 (P = 0.07); I² = 44%				
Test for overall effect: Z = 5.99 (P < 0.00001)				
**2.7.2 12-23 months**				
Arcanjo 2019	0.1616	0.8236	0.3%	1.18 [0.23, 5.91]
Attanasio 2014	-0.2792	0.1812	4.6%	0.76 [0.53, 1.08]
Barffour 2019	-0.0808	0.0634	10.1%	0.92 [0.81, 1.04]
Lundeen 2010	-0.3626	0.0718	9.7%	0.70 [0.60, 0.80]
Menon 2007 (3)	-0.6079	0.2377	3.2%	0.54 [0.34, 0.87]
Somasse 2018	-0.0136	0.0716	9.7%	0.99 [0.86, 1.14]
Suchdev 2012	-0.1839	0.0977	8.2%	0.83 [0.69, 1.01]
Zlotkin 2003 (4)	0.5386	0.3861	1.4%	1.71 [0.80, 3.65]
**Subtotal (95% CI)**			**47.3%**	**0.84 [0.72, 0.97]**
Heterogeneity: Tau² = 0.02; Chi² = 21.17, df = 7 (P = 0.004); I² = 67%				
Test for overall effect: Z = 2.35 (P = 0.02)				
**2.7.3 24-59 months**				
Kounnavong 2011	-0.2624	0.2651	2.7%	0.77 [0.46, 1.29]
Macharia-Mutie 2012	-0.5108	0.2596	2.8%	0.60 [0.36, 1.00]
Varma 2007	-1.6215	0.4692	1.0%	0.20 [0.08, 0.50]
**Subtotal (95% CI)**			**6.5%**	**0.50 [0.26, 0.95]**
Heterogeneity: Tau² = 0.22; Chi² = 6.41, df = 2 (P = 0.04); I² = 69%				
Test for overall effect: Z = 2.11 (P = 0.03)				
**Total (95% CI)**			**100.0%**	**0.75 [0.68, 0.82]**
Heterogeneity: Tau² = 0.02; Chi² = 51.89, df = 19 (P < 0.0001); I² = 63%				
Test for overall effect: Z = 5.95 (P < 0.00001)				
Test for subgroup differences: Chi² = 4.71, df = 2 (P = 0.09), I² = 57.5%				

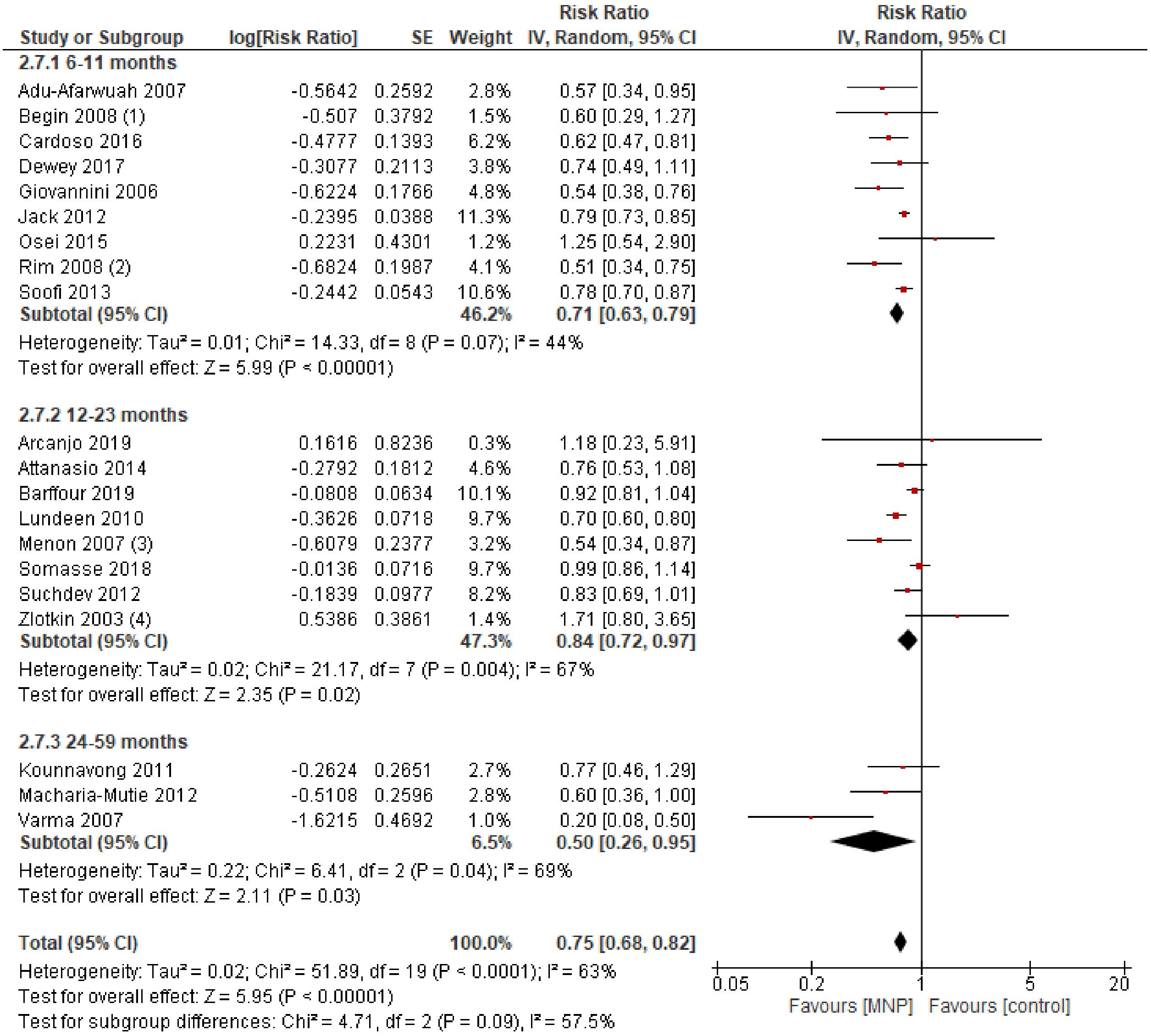

Footnotes
(1) Both groups received why protein concentrate
(2) MNP contains iron only
(3) Both groups received iron fortified wheat soy blend
(4) MNP contains iron & vitamin A only

Anemia: intervention duration

Study or Subgroup	log[Risk Ratio]	SE	Weight	Risk Ratio IV, Random, 95% CI
**2.8.1 Less than 3 months**				
Barffour 2019	-0.0808	0.0634	8.7%	0.92 [0.81, 1.04]
Cardoso 2016	-0.4777	0.1393	5.7%	0.62 [0.47, 0.81]
Lundeen 2010	-0.3626	0.0718	8.3%	0.70 [0.60, 0.80]
Menon 2007 (1)	-0.6079	0.2377	3.1%	0.54 [0.34, 0.87]
Osei 2015	0.2231	0.4301	1.2%	1.25 [0.54, 2.90]
**Subtotal (95% CI)**			**27.1%**	**0.74 [0.59, 0.92]**
Heterogeneity: Tau² = 0.04; Chi² = 15.69, df = 4 (P = 0.003); I² = 75%				
Test for overall effect: Z = 2.74 (P = 0.006)				
**2.8.2 3-5 months**				
Arcanjo 2019	0.1616	0.8236	0.4%	1.18 [0.23, 5.91]
Macharia-Mutie 2012	-0.5108	0.2596	2.8%	0.60 [0.36, 1.00]
Somasse 2018	-0.0136	0.0716	8.4%	0.99 [0.86, 1.14]
**Subtotal (95% CI)**			**11.5%**	**0.86 [0.59, 1.24]**
Heterogeneity: Tau² = 0.05; Chi² = 3.47, df = 2 (P = 0.18); I² = 42%				
Test for overall effect: Z = 0.83 (P = 0.41)				
**2.8.3 6-11 months**				
Adu-Afarwuah 2007	-0.5642	0.2592	2.8%	0.57 [0.34, 0.95]
Begin 2008 (2)	-0.507	0.3792	1.5%	0.60 [0.29, 1.27]
Jack 2012	-0.2395	0.0388	9.5%	0.79 [0.73, 0.85]
Kounnavong 2011	-0.2624	0.2651	2.7%	0.77 [0.46, 1.29]
Rim 2008 (3)	-0.6824	0.1987	4.0%	0.51 [0.34, 0.75]
Varma 2007	-1.6215	0.4692	1.0%	0.20 [0.08, 0.50]
Zlotkin 2003 (4)	0.5386	0.3861	1.5%	1.71 [0.80, 3.65]
**Subtotal (95% CI)**			**22.9%**	**0.65 [0.48, 0.89]**
Heterogeneity: Tau² = 0.10; Chi² = 19.27, df = 6 (P = 0.004); I² = 69%				
Test for overall effect: Z = 2.73 (P = 0.006)				
**2.8.4 12-17 months**				
Giovannini 2006	-0.6224	0.1766	4.5%	0.54 [0.38, 0.76]
Soofi 2013	-0.2442	0.0543	9.0%	0.78 [0.70, 0.87]
Suchdev 2012	-0.1839	0.0977	7.3%	0.83 [0.69, 1.01]
**Subtotal (95% CI)**			**20.8%**	**0.75 [0.63, 0.89]**
Heterogeneity: Tau² = 0.01; Chi² = 4.90, df = 2 (P = 0.09); I² = 59%				
Test for overall effect: Z = 3.25 (P = 0.001)				
**2.8.5 18 months and above**				
Attanasio 2014	-0.2792	0.1812	4.4%	0.76 [0.53, 1.08]
Dewey 2017	-0.3077	0.2113	3.7%	0.74 [0.49, 1.11]
Larson 2018	-0.0198	0.0324	9.6%	0.98 [0.92, 1.04]
**Subtotal (95% CI)**			**17.7%**	**0.88 [0.72, 1.08]**
Heterogeneity: Tau² = 0.02; Chi² = 3.70, df = 2 (P = 0.16); I² = 46%				
Test for overall effect: Z = 1.22 (P = 0.22)				
**Total (95% CI)**			**100.0%**	**0.76 [0.69, 0.84]**
Heterogeneity: Tau² = 0.03; Chi² = 81.62, df = 20 (P < 0.00001); I² = 75%				
Test for overall effect: Z = 5.39 (P < 0.00001)				
Test for subgroup differences: Chi² = 3.36, df = 4 (P = 0.50), I² = 0%				

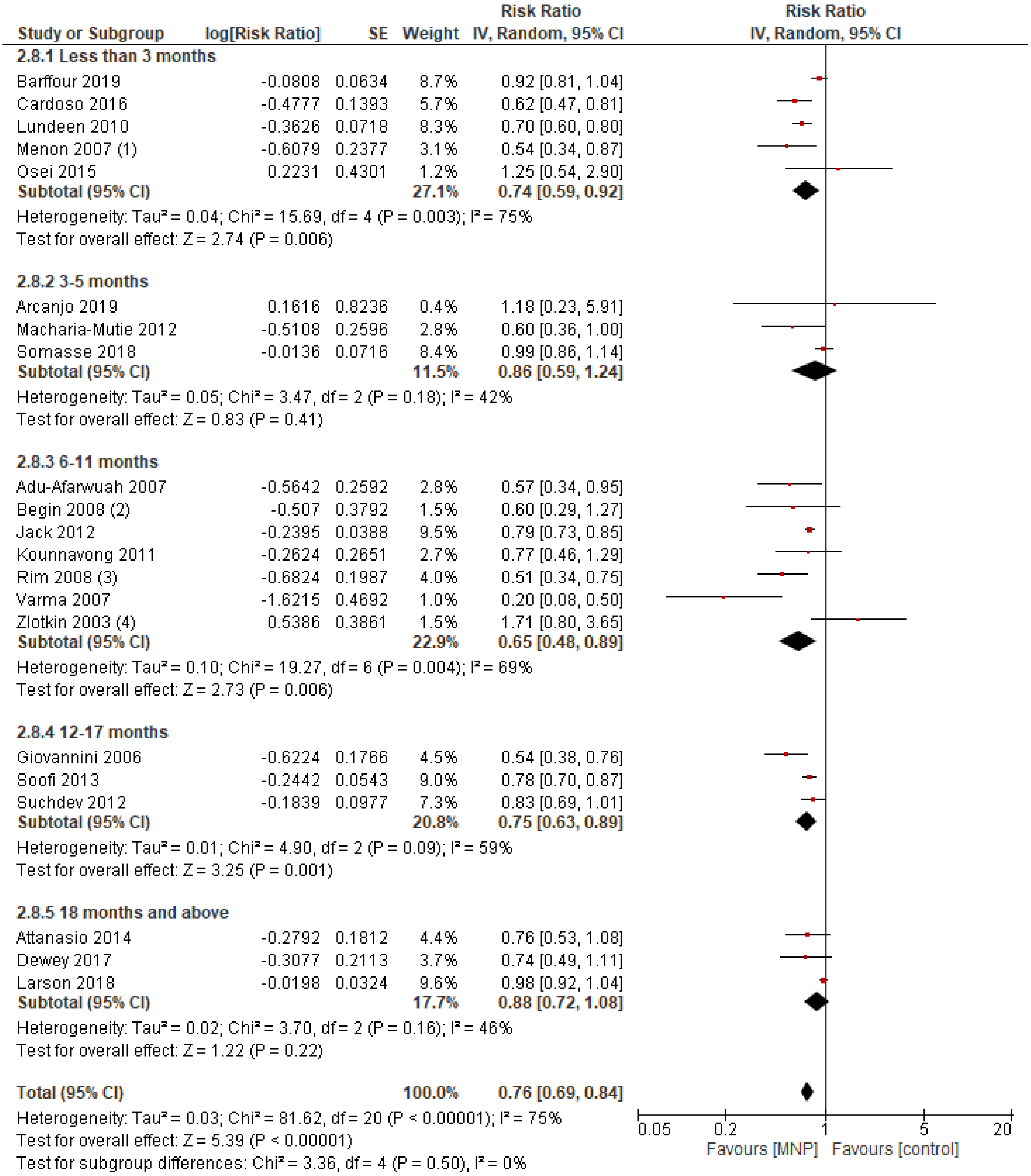

Footnotes
(1) Both groups received iron fortified wheat soy blend
(2) Both groups received why protein concentrate
(3) MNP contains iron only
(4) MNP contains iron & vitamin A only

Anemia: intervention frequency

Study or Subgroup	log[Risk Ratio]	SE	Weight	Risk Ratio IV, Random, 95% CI
**2.9.1 Daily**				
Adu-Afarwuah 2007	-0.5642	0.2592	2.8%	0.57 [0.34, 0.95]
Arcanjo 2019	0.1616	0.8236	0.4%	1.18 [0.23, 5.91]
Attanasio 2014	-0.2792	0.1812	4.4%	0.76 [0.53, 1.08]
Barffour 2019	-0.0808	0.0634	8.7%	0.92 [0.81, 1.04]
Begin 2008 (1)	-0.507	0.3792	1.5%	0.60 [0.29, 1.27]
Dewey 2017	-0.3077	0.2113	3.7%	0.74 [0.49, 1.11]
Giovannini 2006	-0.6224	0.1766	4.5%	0.54 [0.38, 0.76]
Jack 2012	-0.2395	0.0388	9.5%	0.79 [0.73, 0.85]
Larson 2018	-0.0198	0.0324	9.6%	0.98 [0.92, 1.04]
Lundeen 2010	-0.3626	0.0718	8.3%	0.70 [0.60, 0.80]
Macharia-Mutie 2012	-0.5108	0.2596	2.8%	0.60 [0.36, 1.00]
Menon 2007 (2)	-0.6079	0.2377	3.1%	0.54 [0.34, 0.87]
Rim 2008 (3)	-0.6824	0.1987	4.0%	0.51 [0.34, 0.75]
Somasse 2018	-0.0136	0.0716	8.4%	0.99 [0.86, 1.14]
Soofi 2013	-0.2442	0.0543	9.0%	0.78 [0.70, 0.87]
Varma 2007	-1.6215	0.4692	1.0%	0.20 [0.08, 0.50]
Zlotkin 2003 (4)	0.5386	0.3861	1.5%	1.71 [0.80, 3.65]
**Subtotal (95% CI)**			**83.1%**	**0.76 [0.68, 0.85]**
Heterogeneity: Tau² = 0.03; Chi² = 75.49, df = 16 (P < 0.00001); I² = 79%				
Test for overall effect: Z = 4.89 (P < 0.00001)				
**2.9.2 Intermittent**				
Cardoso 2016	-0.4777	0.1393	5.7%	0.62 [0.47, 0.81]
Kounnavong 2011	-0.2624	0.2651	2.7%	0.77 [0.46, 1.29]
Osei 2015	0.2231	0.4301	1.2%	1.25 [0.54, 2.90]
Suchdev 2012	-0.1839	0.0977	7.3%	0.83 [0.69, 1.01]
**Subtotal (95% CI)**			**16.9%**	**0.76 [0.62, 0.94]**
Heterogeneity: Tau² = 0.01; Chi² = 4.31, df = 3 (P = 0.23); I² = 30%				
Test for overall effect: Z = 2.57 (P = 0.01)				
**Total (95% CI)**			**100.0%**	**0.76 [0.69, 0.84]**
Heterogeneity: Tau² = 0.03; Chi² = 81.62, df = 20 (P < 0.00001); I² = 75%				
Test for overall effect: Z = 5.39 (P < 0.00001)				
Test for subgroup differences: Chi² = 0.00, df = 1 (P = 0.95), I² = 0%				

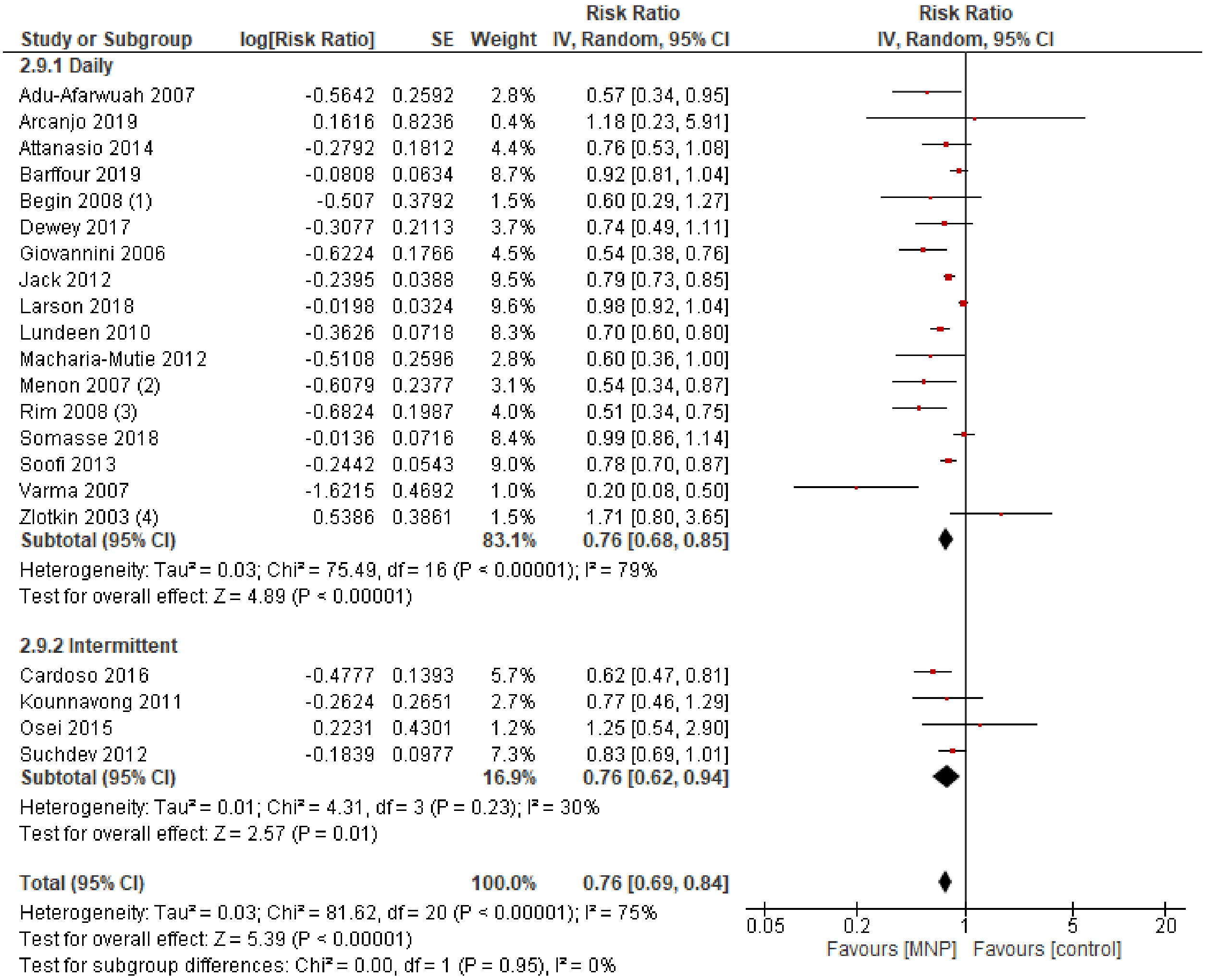

Footnotes
(1) Both groups received why protein concentrate
(2) Both groups received iron fortified wheat soy blend
(3) MNP contains iron only
(4) MNP contains iron & vitamin A only

Anemia: nutritional status at baseline (anemic vs. non-anemic)

Study or Subgroup	log[Risk Ratio]	SE	Weight	Risk Ratio IV, Random, 95% CI
**2.10.1 Non-anemic at baseline**				
Arcanjo 2019	0.1616	0.8236	0.4%	1.18 [0.23, 5.91]
Attanasio 2014	-0.2792	0.1812	4.6%	0.76 [0.53, 1.08]
Begin 2008 (1)	-0.507	0.3792	1.6%	0.60 [0.29, 1.27]
Cardoso 2016	-0.4777	0.1393	5.9%	0.62 [0.47, 0.81]
Macharia-Mutie 2012	-0.5108	0.2596	2.9%	0.60 [0.36, 1.00]
Rim 2008 (2)	-0.6824	0.1987	4.1%	0.51 [0.34, 0.75]
Soofi 2013	-0.2442	0.0543	9.3%	0.78 [0.70, 0.87]
Varma 2007	-1.6215	0.4692	1.1%	0.20 [0.08, 0.50]
Zlotkin 2003 (3)	0.5386	0.3861	1.5%	1.71 [0.80, 3.65]
**Subtotal (95% CI)**			**31.5%**	**0.67 [0.53, 0.83]**
Heterogeneity: Tau² = 0.05; Chi² = 20.30, df = 8 (P = 0.009); I² = 61%				
Test for overall effect: Z = 3.58 (P = 0.0003)				
**2.10.2 Anemic at baseline**				
Adu-Afarwuah 2007	-0.5642	0.2592	2.9%	0.57 [0.34, 0.95]
Barffour 2019	-0.0808	0.0634	9.0%	0.92 [0.81, 1.04]
Giovannini 2006	-0.6224	0.1766	4.7%	0.54 [0.38, 0.76]
Jack 2012	-0.2395	0.0388	9.8%	0.79 [0.73, 0.85]
Kounnavong 2011	-0.2624	0.2651	2.8%	0.77 [0.46, 1.29]
Larson 2018	-0.0198	0.0324	9.9%	0.98 [0.92, 1.04]
Lundeen 2010	-0.3626	0.0718	8.6%	0.70 [0.60, 0.80]
Menon 2007 (4)	-0.6079	0.2377	3.3%	0.54 [0.34, 0.87]
Osei 2015	0.2231	0.4301	1.3%	1.25 [0.54, 2.90]
Somasse 2018	-0.0136	0.0716	8.6%	0.99 [0.86, 1.14]
Suchdev 2012	-0.1839	0.0977	7.6%	0.83 [0.69, 1.01]
**Subtotal (95% CI)**			**68.5%**	**0.81 [0.72, 0.91]**
Heterogeneity: Tau² = 0.02; Chi² = 49.04, df = 10 (P < 0.00001); I² = 80%				
Test for overall effect: Z = 3.64 (P = 0.0003)				
**Total (95% CI)**			**100.0%**	**0.76 [0.69, 0.84]**
Heterogeneity: Tau² = 0.03; Chi² = 81.16, df = 19 (P < 0.00001); I² = 77%				
Test for overall effect: Z = 5.24 (P < 0.00001)				
Test for subgroup differences: Chi² = 2.31, df = 1 (P = 0.13), I² = 56.6%				

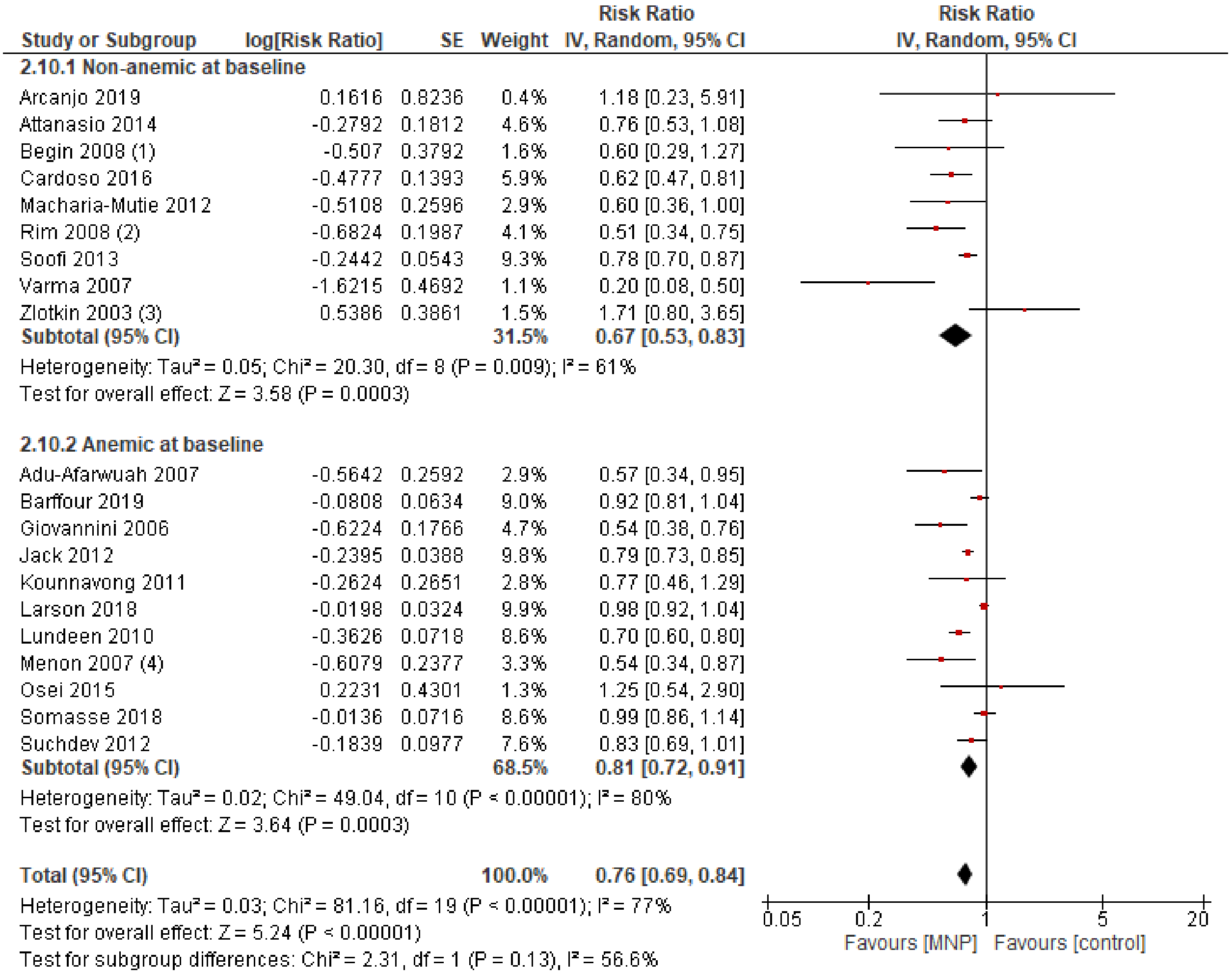

Footnotes
(1) Both groups received why protein concentrate
(2) MNP contains iron only
(3) MNP contains iron & vitamin A only
(4) Both groups received iron fortified wheat soy blend

Comparison 7: Lipid-based Nutrient Supplementation vs. Placebo/No Intervention (Efficacy)

Wasting

Study or Subgroup	LNS supplementation Events	LNS supplementation Total	Control Events	Control Total	Weight	Risk Ratio M-H, Random, 95% CI
Adu-Afarwuah 2016	22	347	31	337	2.8%	0.69 [0.41, 1.17]
Ashorn 2015	7	214	8	220	0.8%	0.90 [0.33, 2.44]
Christian 2015 (1)	123	896	138	843	11.7%	0.84 [0.67, 1.05]
Hess 2015 (2)	72	811	60	523	6.6%	0.77 [0.56, 1.07]
Luby 2018	567	699	1104	1302	38.5%	0.96 [0.92, 1.00]
Null 2018	695	811	1536	1864	39.6%	1.04 [1.00, 1.08]
**Total (95% CI)**		**3778**		**5089**	**100.0%**	**0.95 [0.87, 1.04]**
Total events	1486		2877			

Heterogeneity: Tau² = 0.01; Chi² = 18.77, df = 5 (P = 0.002); I² = 73%
Test for overall effect: Z = 1.09 (P = 0.28)

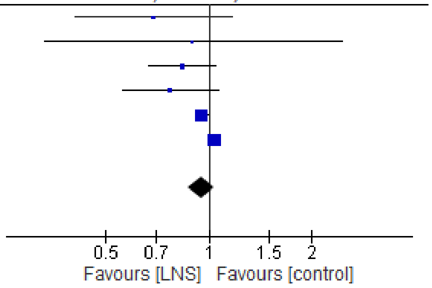

Footnotes
(1) Plumpy'doz group only
(2) LNS (Zn 5mg) and LNS (Zn 10mg) groups were combined into one group

Underweight

Study or Subgroup	LNS supplementation Events	LNS supplementation Total	Control Events	Control Total	Weight	Risk Ratio M-H, Random, 95% CI
Adu-Afarwuah 2016	37	347	55	337	6.3%	0.65 [0.44, 0.96]
Ashorn 2015	28	214	22	220	3.8%	1.31 [0.77, 2.21]
Christian 2015 (1)	292	896	330	843	20.7%	0.83 [0.73, 0.94]
Hess 2015 (2)	154	811	137	523	14.5%	0.72 [0.59, 0.89]
Luby 2018	573	699	1121	1302	27.2%	0.95 [0.91, 0.99]
Null 2018	698	811	1548	1864	27.5%	1.04 [1.00, 1.07]
**Total (95% CI)**		**3778**		**5089**	**100.0%**	**0.90 [0.81, 1.01]**
Total events	1782		3213			

Heterogeneity: Tau² = 0.01; Chi² = 41.29, df = 5 (P < 0.00001); I² = 88%
Test for overall effect: Z = 1.85 (P = 0.06)

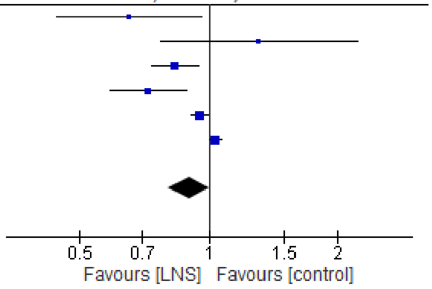

Footnotes
(1) Plumpy'doz group only
(2) LNS (Zn 5mg) and LNS (Zn 10mg) groups were combined into one group

Height (cm)

Study or Subgroup	Mean Difference	SE	Weight	Mean Difference IV, Random, 95% CI
Adu-Afarwuah 2016	0.8	0.2173	14.0%	0.80 [0.37, 1.23]
Ashorn 2015	-0.2	0.288	11.0%	-0.20 [-0.76, 0.36]
Christian 2015 (1)	0.3	0.0999	19.4%	0.30 [0.10, 0.50]
Hess 2015 (2)	0.7	0.1832	15.6%	0.70 [0.34, 1.06]
Maleta 2015 (3)	0.1	0.1885	15.3%	0.10 [-0.27, 0.47]
Mangani 2014 (4)	0.2	0.1917	15.2%	0.20 [-0.18, 0.58]
Smuts 2019 (5)	-0.5	0.3266	9.6%	-0.50 [-1.14, 0.14]
**Total (95% CI)**			**100.0%**	**0.25 [-0.01, 0.52]**

Heterogeneity: Tau² = 0.08; Chi² = 20.47, df = 6 (P = 0.002); I² = 71%
Test for overall effect: Z = 1.87 (P = 0.06)

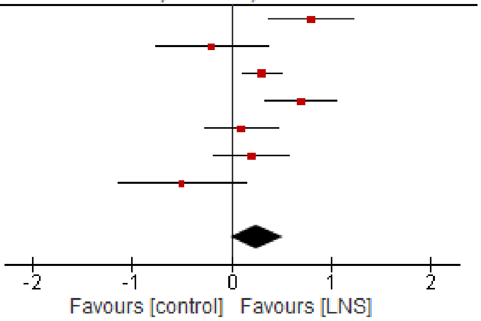

Footnotes
(1) Plumpy'doz group only
(2) LNS (Zn 5mg) and LNS (Zn 10mg) groups were combined into one group
(3) 20g LNS group only
(4) LNS (with milk) group only
(5) SQ LNS group only

Weight (kg)

Study or Subgroup	LNS supplementation Mean	SD	Total	Control Mean	SD	Total	Weight	Mean Difference IV, Random, 95% CI
Adu-Afarwuah 2016	9.87	1.23	348	9.58	1.17	340	18.4%	0.29 [0.11, 0.47]
Ashorn 2015	9.6	1.1	214	9.7	1.2	220	16.7%	-0.10 [-0.32, 0.12]
Hess 2015 (1)	9.295	1.0995	811	9.02	1.18	523	20.9%	0.28 [0.15, 0.40]
Maleta 2015 (2)	2.4	0.8	233	2.5	2.8	240	10.5%	-0.10 [-0.47, 0.27]
Mangani 2014 (3)	2.53	0.78	191	2.42	0.77	185	19.5%	0.11 [-0.05, 0.27]
Smuts 2019 (4)	8.7	1.3	151	8.9	1.3	201	14.1%	-0.20 [-0.47, 0.07]
**Total (95% CI)**			**1948**			**1709**	**100.0%**	**0.08 [-0.08, 0.24]**

Heterogeneity: Tau² = 0.03; Chi² = 19.46, df = 5 (P = 0.002); I² = 74%
Test for overall effect: Z = 0.94 (P = 0.35)

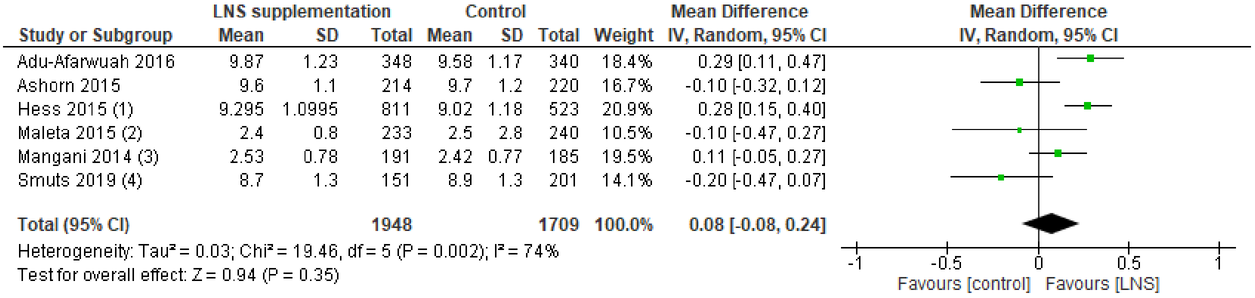

Footnotes
(1) LNS (Zn 5mg) and LNS (Zn 10mg) groups were combined into one group
(2) 20g LNS group only
(3) LNS (with milk) group only
(4) SQ LNS group only

Length-for-age (z-score)

Study or Subgroup	Mean Difference	SE	Weight	Mean Difference IV, Random, 95% CI
Adu-Afarwuah 2007	0.26	0.1505	3.0%	0.26 [-0.03, 0.55]
Adu-Afarwuah 2016	0.28	0.0757	6.8%	0.28 [0.13, 0.43]
Ashorn 2015	-0.06	0.0979	5.3%	-0.06 [-0.25, 0.13]
Batra 2016	0.02	0.017	11.3%	0.02 [-0.01, 0.05]
Christian 2015 (1)	0.11	0.0482	9.0%	0.11 [0.02, 0.20]
Dewey 2017	0.07	0.0477	9.1%	0.07 [-0.02, 0.16]
Hess 2015 (2)	0.29	0.064	7.7%	0.29 [0.16, 0.42]
Huybregts 2012	0.27	0.1181	4.2%	0.27 [0.04, 0.50]
Luby 2018	0.26	0.0597	8.1%	0.26 [0.14, 0.38]
Maleta 2015 (3)	0.01	0.0681	7.4%	0.01 [-0.12, 0.14]
Mangani 2014 (4)	0.08	0.0723	7.1%	0.08 [-0.06, 0.22]
Null 2018	0.1	0.0565	8.3%	0.10 [-0.01, 0.21]
Rosado 2011	0	0.1091	4.7%	0.00 [-0.21, 0.21]
Smuts 2019 (5)	0.02	0.0612	7.9%	0.02 [-0.10, 0.14]
**Total (95% CI)**			**100.0%**	**0.11 [0.05, 0.17]**

Heterogeneity: Tau² = 0.01; Chi² = 47.27, df = 13 (P < 0.00001); I² = 72%
Test for overall effect: Z = 3.72 (P = 0.0002)

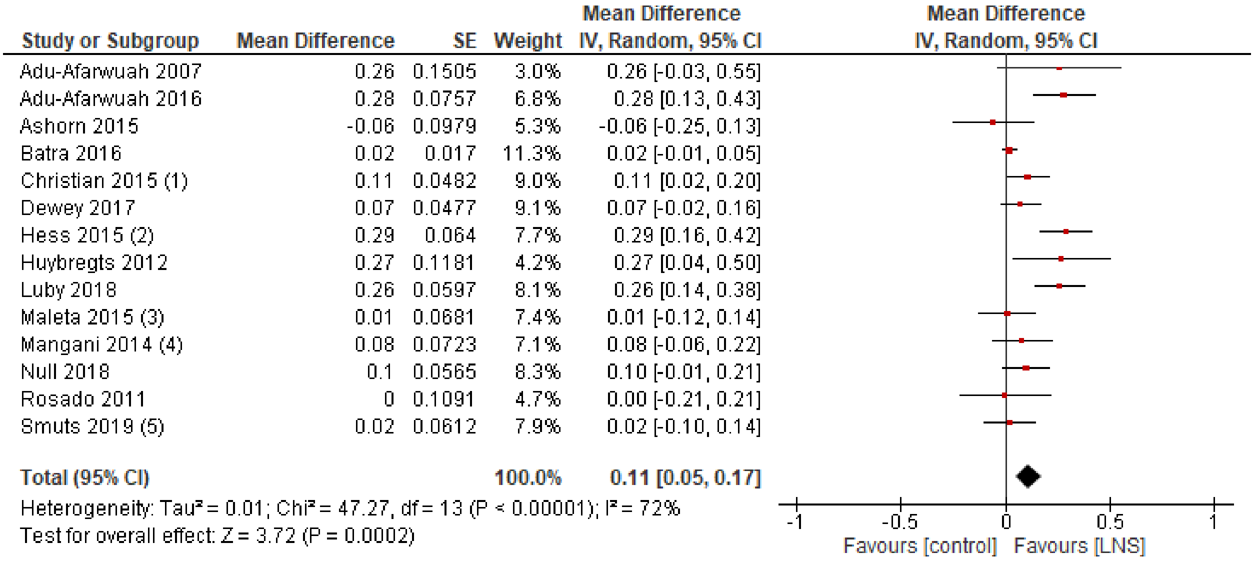

Footnotes
(1) Plumpy'doz group only
(2) LNS (Zn 5mg) and LNS (Zn 10mg) groups were combined into one group
(3) 20g LNS group only
(4) LNS (with milk) group only
(5) SQ LNS group only

Weight-for-age (z-score)

Study or Subgroup	Mean Difference	SE	Weight	Mean Difference IV, Random, 95% CI
Adu-Afarwuah 2007	0.34	0.1656	2.8%	0.34 [0.02, 0.66]
Adu-Afarwuah 2016	0.24	0.0795	7.5%	0.24 [0.08, 0.40]
Ashorn 2015	-0.12	0.0946	6.2%	-0.12 [-0.31, 0.07]
Batra 2016	0.11	0.017	14.3%	0.11 [0.08, 0.14]
Dewey 2017	0.1	0.0469	11.1%	0.10 [0.01, 0.19]
Hess 2015 (1)	0.27	0.0607	9.5%	0.27 [0.15, 0.39]
Luby 2018	0.25	0.0599	9.6%	0.25 [0.13, 0.37]
Maleta 2015 (2)	-0.12	0.133	4.0%	-0.12 [-0.38, 0.14]
Mangani 2014 (3)	0.09	0.0769	7.8%	0.09 [-0.06, 0.24]
Null 2018	0.07	0.0502	10.7%	0.07 [-0.03, 0.17]
Rosado 2011	-0.1	0.0922	6.4%	-0.10 [-0.28, 0.08]
Smuts 2019 (4)	0.02	0.0561	10.0%	0.02 [-0.09, 0.13]
**Total (95% CI)**			**100.0%**	**0.10 [0.04, 0.16]**

Heterogeneity: $Tau^2 = 0.01$; $Chi^2 = 34.42$, df = 11 (P = 0.0003); $I^2 = 68\%$
Test for overall effect: Z = 3.29 (P = 0.0010)

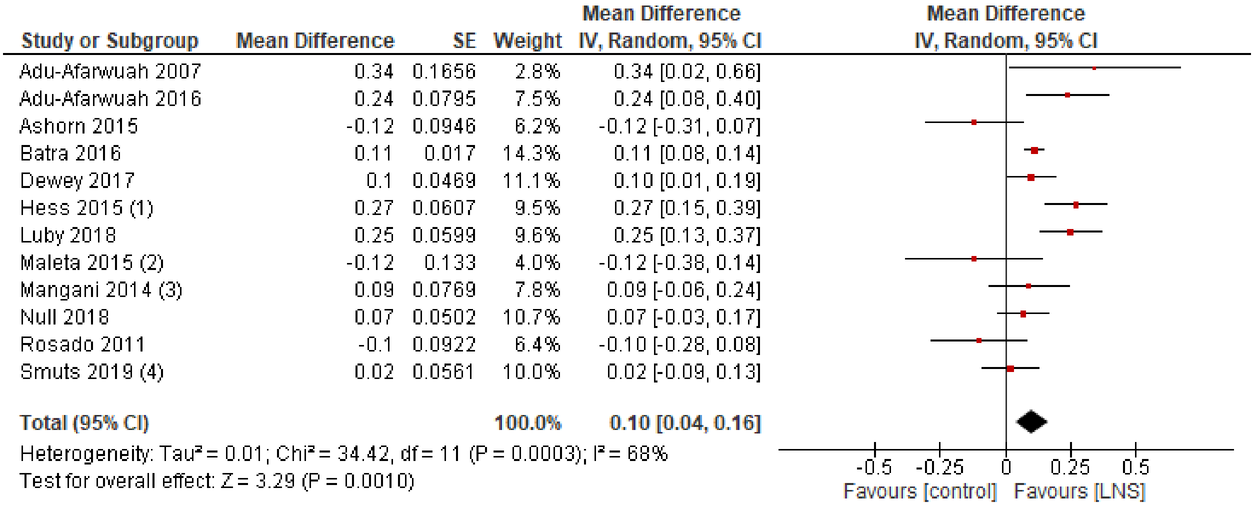

Footnotes
(1) LNS (Zn 5mg) and LNS (Zn 10mg) groups were combined into one group
(2) 20g LNS group only
(3) LNS (with milk) group only
(4) SQ LNS group only

Weight-for-height (z-score)

Study or Subgroup	LNS supplementation Mean	SD	Total	Control Mean	SD	Total	Weight	Mean Difference IV, Random, 95% CI
Adu-Afarwuah 2007	-0.43	1.1	97	-0.74	1.1	81	2.3%	0.31 [-0.01, 0.63]
Adu-Afarwuah 2016	-0.49	1.02	347	-0.64	1.02	337	7.2%	0.15 [-0.00, 0.30]
Ashorn 2015	-0.21	0.99	214	-0.08	1	220	5.5%	-0.13 [-0.32, 0.06]
Batra 2016	-0.16	0.1	82	-0.31	0.1	54	17.5%	0.15 [0.12, 0.18]
Dewey 2017	-1.03	0.87	772	-1.11	0.89	802	12.4%	0.08 [-0.01, 0.17]
Hess 2015 (1)	-0.7549	0.9699	811	-0.93	1.01	523	10.3%	0.18 [0.07, 0.28]
Huybregts 2012	-1.05	0.93	341	-1.09	0.95	251	7.2%	0.04 [-0.11, 0.19]
Luby 2018	-0.71	1	457	-0.88	0.93	890	10.3%	0.17 [0.06, 0.28]
Maleta 2015 (2)	-0.62	1.07	233	-0.61	0.92	240	5.8%	-0.01 [-0.19, 0.17]
Mangani 2014 (3)	-0.57	1.02	191	-0.66	0.93	185	5.1%	0.09 [-0.11, 0.29]
Null 2018	0.14	0.92	560	0.11	0.94	1239	11.9%	0.03 [-0.06, 0.12]
Rosado 2011	0	0.6	57	0.1	0.6	62	4.5%	-0.10 [-0.32, 0.12]
**Total (95% CI)**			**4162**			**4884**	**100.0%**	**0.09 [0.04, 0.14]**

Heterogeneity: $Tau^2 = 0.00$; $Chi^2 = 24.59$, df = 11 (P = 0.01); $I^2 = 55\%$
Test for overall effect: Z = 3.31 (P = 0.0009)

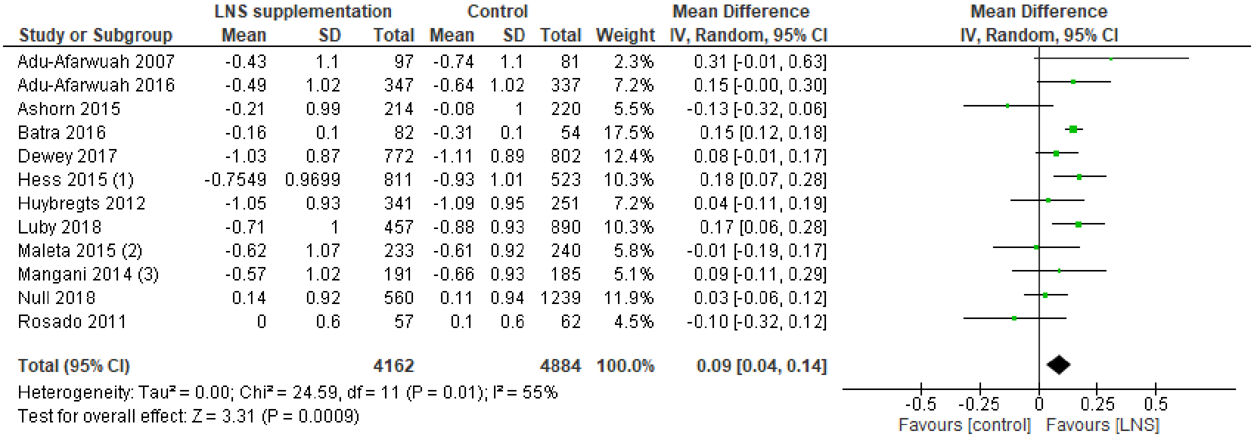

Footnotes
(1) LNS (Zn 5mg) and LNS (Zn 10mg) groups were combined into one group
(2) 20g LNS group only
(3) LNS (with milk) group only

Hemoglobin (g/L)

Study or Subgroup	LNS supplementation Mean	SD	Total	Control Mean	SD	Total	Weight	Mean Difference IV, Random, 95% CI
Adu-Afarwuah 2007	114.3	14.4	98	105.8	14.3	96	11.5%	8.50 [4.46, 12.54]
Batra 2016	-5	2	85	0.03	1	58	13.2%	-5.03 [-5.53, -4.53]
Dewey 2017	115.6	9.1702	151	112.4	10.0835	160	12.7%	3.20 [1.06, 5.34]
Hess 2015 (1)	97.4954	15.0007	987	88	16	664	12.9%	9.50 [7.96, 11.03]
Huybregts 2012	105.8	14.3	326	102.5	15.2	233	12.5%	3.30 [0.81, 5.79]
Rosado 2011	6	11	55	4	11	61	11.6%	2.00 [-2.01, 6.01]
Siega-Riz 2014	108.7	1.1	116	105.7	1.1	94	13.2%	3.00 [2.70, 3.30]
Smuts 2019 (2)	118.67	11.85	151	115.33	14.07	201	12.4%	3.34 [0.63, 6.05]
**Total (95% CI)**			**1969**			**1567**	**100.0%**	**3.41 [-0.48, 7.29]**

Heterogeneity: Tau² = 29.79; Chi² = 875.99, df = 7 (P < 0.00001); I² = 99%
Test for overall effect: Z = 1.72 (P = 0.09)

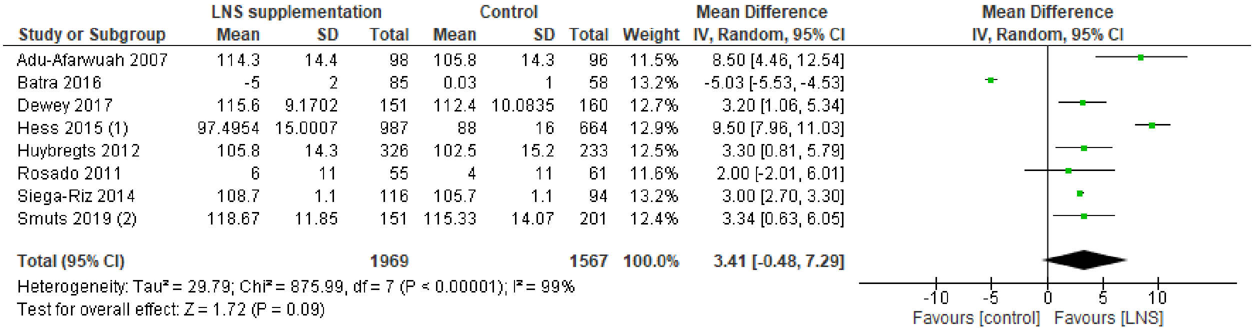

Footnotes
(1) LNS (Zn 5mg) and LNS (Zn 10mg) groups were combined into one group
(2) SQ LNS group only

Head circumference (cm)

Study or Subgroup	Mean Difference	SE	Weight	Mean Difference IV, Random, 95% CI
Adu-Afarwuah 2007	0.4	0.1806	12.3%	0.40 [0.05, 0.75]
Adu-Afarwuah 2016	0.2	0.1011	20.3%	0.20 [0.00, 0.40]
Ashorn 2015	-0.2	0.1392	16.1%	-0.20 [-0.47, 0.07]
Hess 2015 (1)	0.2	0.074	23.5%	0.20 [0.05, 0.35]
Smuts 2019 (2)	-0.03	0.0306	27.7%	-0.03 [-0.09, 0.03]
**Total (95% CI)**			**100.0%**	**0.10 [-0.07, 0.26]**

Heterogeneity: Tau² = 0.02; Chi² = 18.68, df = 4 (P = 0.0009); I² = 79%
Test for overall effect: Z = 1.15 (P = 0.25)

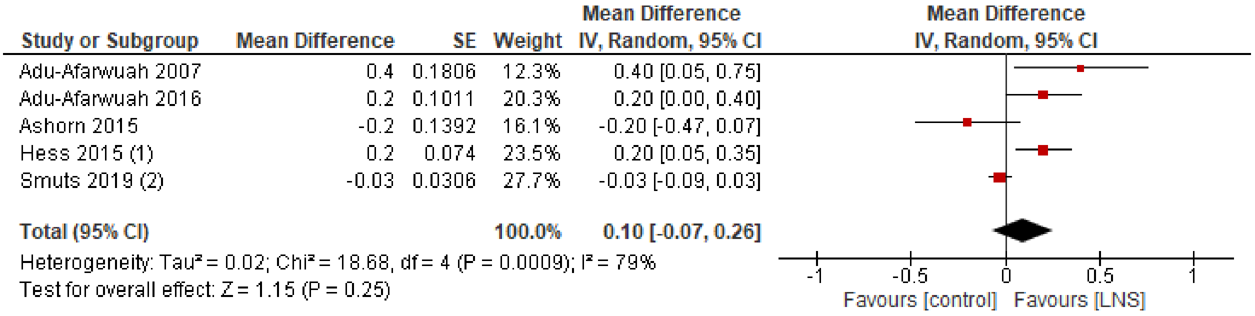

Footnotes
(1) LNS (Zn 5mg) and LNS (Zn 10mg) groups were combined into one group
(2) SQ LNS group only

Mid-upper arm circumference (cm)

Study or Subgroup	Mean Difference	SE	Weight	Mean Difference IV, Random, 95% CI
Adu-Afarwuah 2016	0.2	0.0801	19.5%	0.20 [0.04, 0.36]
Ashorn 2015	-0.1	0.1062	16.8%	-0.10 [-0.31, 0.11]
Hess 2015 (1)	0.3	0.0606	21.4%	0.30 [0.18, 0.42]
Maleta 2015 (2)	0.1	0.0876	18.7%	0.10 [-0.07, 0.27]
Smuts 2019 (3)	-0.02	0.0357	23.5%	-0.02 [-0.09, 0.05]
**Total (95% CI)**			**100.0%**	**0.10 [-0.05, 0.25]**

Heterogeneity: Tau² = 0.02; Chi² = 26.07, df = 4 (P < 0.0001); I² = 85%
Test for overall effect: Z = 1.30 (P = 0.19)

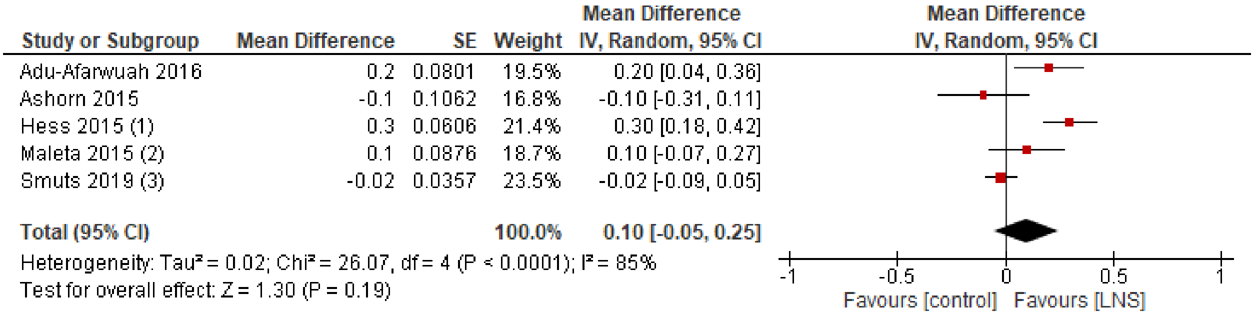

Footnotes
(1) LNS (Zn 5mg) and LNS (Zn 10mg) groups were combined into one group
(2) 20g LNS group only
(3) SQ LNS group only

Plasma/serum zinc (log) (µg/dL)

Study or Subgroup	LNS supplementation Mean	SD	Total	Control Mean	SD	Total	Weight	Mean Difference IV, Random, 95% CI
Adu-Afarwuah 2007	4.07	0.27	55	4.1	0.26	69	7.5%	-0.03 [-0.12, 0.06]
Hess 2015 (1)	4.17	0.0199	153	4.17	0.02	93	46.4%	0.00 [-0.01, 0.01]
Siega-Riz 2014	4.18	0.02	99	4.15	0.02	77	46.1%	0.03 [0.02, 0.04]
**Total (95% CI)**			**307**			**239**	**100.0%**	**0.01 [-0.02, 0.04]**

Heterogeneity: Tau² = 0.00; Chi² = 56.61, df = 2 (P < 0.00001); I² = 96%
Test for overall effect: Z = 0.81 (P = 0.42)

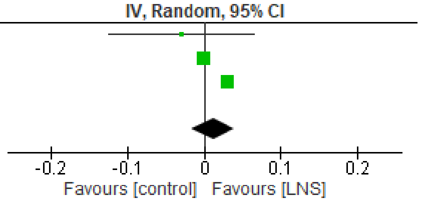

Footnotes
(1) LNS (Zn 5mg) and LNS (Zn 10mg) groups were combined into one group

Mental development (language)

Study or Subgroup	LNS supplementation Mean	SD	Total	Control Mean	SD	Total	Weight	Std. Mean Difference IV, Random, 95% CI
Adu-Afarwuah 2016 (1)	0.02	1.04	339	-0.05	0.95	338	21.6%	0.07 [-0.08, 0.22]
Ashorn 2015 (2)	0	0.99	225	-0.01	1	225	17.6%	0.01 [-0.17, 0.19]
Hess 2015 (3)	0.09	0.96	165	-0.23	1.01	251	16.3%	0.32 [0.12, 0.52]
Luby 2018 (4)	0.19	0.86	436	0	1	886	26.6%	0.20 [0.08, 0.31]
Maleta 2015 (5)	0.01	0.96	227	-0.02	1.01	230	17.8%	0.03 [-0.15, 0.21]
**Total (95% CI)**			**1392**			**1930**	**100.0%**	**0.13 [0.02, 0.23]**

Heterogeneity: Tau² = 0.01; Chi² = 8.35, df = 4 (P = 0.08); I² = 52%
Test for overall effect: Z = 2.39 (P = 0.02)

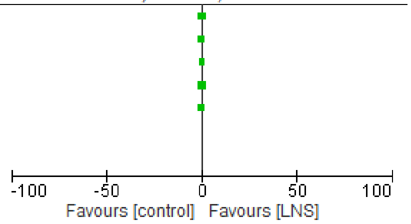

Footnotes
(1) Language (100-word vocabulary checklist based on the MacArthur-Bates Communicative Development Inventory)
(2) Language (100-word vocabulary checklist based on the MacArthur-Bates Communicative Development Inventory)
(3) Language (Developmental Milestones Checklist II)
(4) Extended Ages and Stages Questionnaire after 2 years of intervention
(5) Language (100-word vocabulary checklist based on the MacArthur-Bates Communicative Development Inventory)

Mental development (personal-social/socioemotional)

Study or Subgroup	LNS supplementation Mean	SD	Total	Control Mean	SD	Total	Weight	Std. Mean Difference IV, Random, 95% CI
Adu-Afarwuah 2016 (1)	0	1.06	340	0.01	0.93	338	21.3%	-0.01 [-0.16, 0.14]
Ashorn 2015 (2)	0.01	1.03	225	0	1.04	225	18.3%	0.01 [-0.18, 0.19]
Hess 2015 (3)	0.1	0.89	165	-0.22	1.08	251	17.3%	0.32 [0.12, 0.51]
Luby 2018 (4)	0.22	0.97	426	0	1	886	24.6%	0.22 [0.11, 0.34]
Maleta 2015 (5)	0.03	1.01	227	-0.03	0.99	230	18.5%	0.06 [-0.12, 0.24]
**Total (95% CI)**			**1383**			**1930**	**100.0%**	**0.12 [-0.00, 0.24]**

Heterogeneity: Tau² = 0.01; Chi² = 11.36, df = 4 (P = 0.02); I² = 65%
Test for overall effect: Z = 1.92 (P = 0.05)

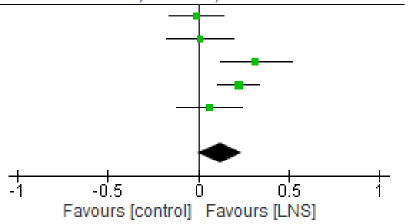

Footnotes
(1) Socioemotional (Profile of Social and Emotional Development)
(2) Socioemotional (Profile of Social and Emotional Development)
(3) Personal-social (Developmental Milestones Checklist II)
(4) Extended Ages and Stages Questionnaire after 2 years of intervention
(5) Socioemotional (Profile of Social and Emotional Development)

Mental development (executive function)

Study or Subgroup	LNS supplementation Mean	SD	Total	Control Mean	SD	Total	Weight	Mean Difference IV, Random, 95% CI
Adu-Afarwuah 2016 (1)	0.01	0.96	293	-0.01	1.03	287	45.7%	0.02 [-0.14, 0.18]
Ashorn 2015 (2)	-0.05	1	189	0.03	1	195	30.0%	-0.08 [-0.28, 0.12]
Maleta 2015 (3)	-0.02	1.08	160	0.05	0.97	168	24.3%	-0.07 [-0.29, 0.15]
**Total (95% CI)**			**642**			**650**	**100.0%**	**-0.03 [-0.14, 0.08]**

Heterogeneity: Tau² = 0.00; Chi² = 0.73, df = 2 (P = 0.69); I² = 0%
Test for overall effect: Z = 0.57 (P = 0.57)

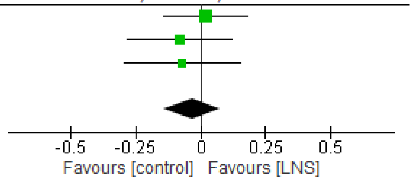

Footnotes
(1) A not B correct score
(2) A not B correct score
(3) A not B correct score

Motor development

Study or Subgroup	LNS supplementation Mean	SD	Total	Control Mean	SD	Total	Weight	Std. Mean Difference IV, Random, 95% CI
Adu-Afarwuah 2016 (1)	0.02	1.01	305	0.05	0.89	298	17.7%	-0.03 [-0.19, 0.13]
Ashorn 2015 (2)	0.01	0.99	223	0.03	0.99	222	16.0%	-0.02 [-0.21, 0.17]
Hess 2015 (3)	0.16	0.88	165	-0.27	1.15	251	15.2%	0.41 [0.21, 0.61]
Luby 2018 (4)	0.18	0.95	426	0	1	886	20.7%	0.18 [0.07, 0.30]
Maleta 2015 (5)	0.05	1.05	219	-0.02	0.99	226	16.0%	0.07 [-0.12, 0.25]
Smuts 2019 (6)	33	4.44	151	32.33	3.7	201	14.4%	0.17 [-0.05, 0.38]
**Total (95% CI)**			**1489**			**2084**	**100.0%**	**0.13 [0.00, 0.25]**

Heterogeneity: Tau² = 0.02; Chi² = 15.31, df = 5 (P = 0.009); I² = 67%
Test for overall effect: Z = 2.02 (P = 0.04)

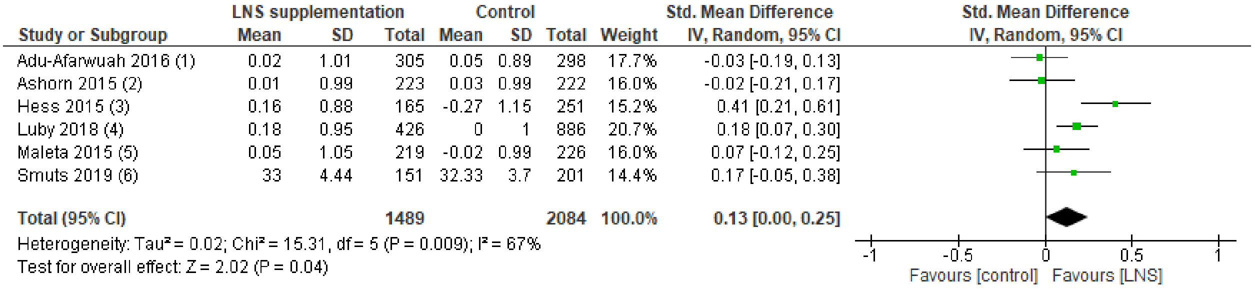

Footnotes
(1) Kilifi Developmental Inventory
(2) Kilifi Developmental Inventory
(3) Developmental Milestones Checklist II
(4) Extended Ages and Stages Questionnaire after 2 years of intervention
(5) Kilifi Developmental Inventory
(6) South African Parent Rating

Motor development (standing independently at 12 months of age)

Study or Subgroup	LNS supplementation Events	Total	Control Events	Total	Weight	Risk Ratio M-H, Random, 95% CI
Adu-Afarwuah 2007	62	94	48	88	10.4%	1.21 [0.95, 1.54]
Adu-Afarwuah 2016	246	325	242	322	26.7%	1.01 [0.92, 1.10]
Ashorn 2015	189	218	180	219	27.8%	1.05 [0.97, 1.14]
Luby 2018	469	473	940	948	35.1%	1.00 [0.99, 1.01]
**Total (95% CI)**		**1110**		**1577**	**100.0%**	**1.04 [0.95, 1.14]**
Total events	966		1410			

Heterogeneity: Tau² = 0.01; Chi² = 17.20, df = 3 (P = 0.0006); I² = 83%
Test for overall effect: Z = 0.78 (P = 0.44)

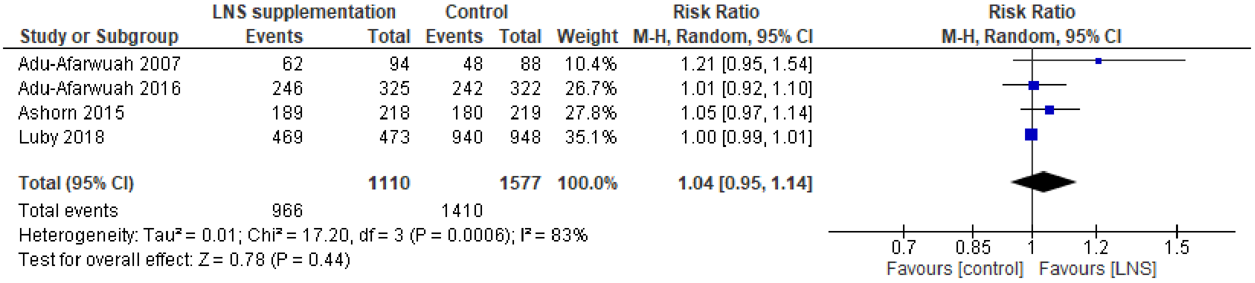

Motor development (walking independently at 12 months of age)

Study or Subgroup	LNS supplementation Events	Total	Control Events	Total	Weight	Risk Ratio M-H, Random, 95% CI
Adu-Afarwuah 2007	46	94	22	88	22.9%	1.96 [1.29, 2.97]
Adu-Afarwuah 2016	176	329	142	327	25.6%	1.23 [1.05, 1.44]
Ashorn 2015	127	218	108	221	25.4%	1.19 [1.00, 1.42]
Luby 2018	467	473	940	948	26.1%	1.00 [0.98, 1.01]
**Total (95% CI)**		**1114**		**1584**	**100.0%**	**1.29 [0.72, 2.28]**
Total events	816		1212			

Heterogeneity: Tau² = 0.33; Chi² = 198.25, df = 3 (P < 0.00001); I² = 98%
Test for overall effect: Z = 0.85 (P = 0.39)

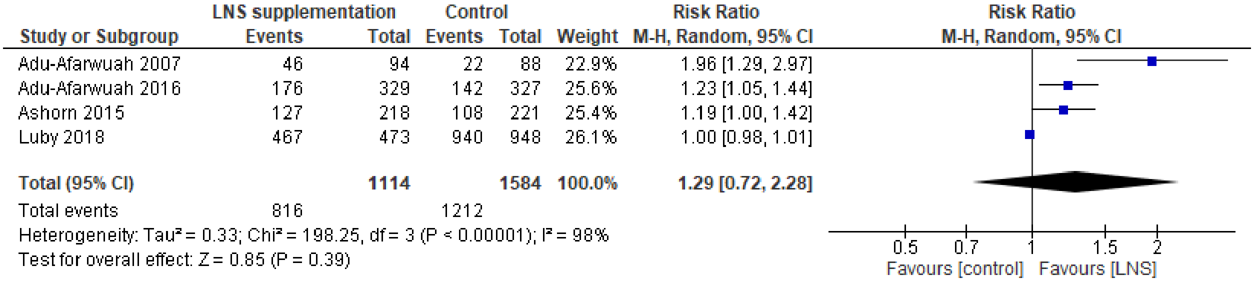

Comparison 7: Subgroup Analyses for Lipid Nutrient Supplementation vs. Placebo/No Intervention (Efficacy)

Anemia: WHO region

Study or Subgroup	LNS supplementation Events	LNS supplementation Total	Control Events	Control Total	Weight	Risk Ratio M-H, Random, 95% CI
**10.1.1 Africa**						
Adu-Afarwuah 2007 (1)	10	98	31	96	2.6%	0.32 [0.16, 0.61]
Hess 2015 (2)	516	658	403	443	27.4%	0.86 [0.82, 0.91]
Huybregts 2012	185	326	156	233	20.7%	0.85 [0.74, 0.97]
Mangani 2014	121	191	120	185	18.9%	0.98 [0.84, 1.14]
Smuts 2019 (3)	36	151	70	201	7.8%	0.68 [0.49, 0.96]
**Subtotal (95% CI)**		**1424**		**1158**	**77.3%**	**0.83 [0.72, 0.95]**
Total events	868		780			
Heterogeneity: Tau² = 0.01; Chi² = 14.43, df = 4 (P = 0.006); I² = 72%						
Test for overall effect: Z = 2.66 (P = 0.008)						
**10.1.2 Americas**						
Rosado 2011	11	55	12	61	2.1%	1.02 [0.49, 2.11]
Siega-Riz 2014 (4)	60	116	52	94	11.6%	0.94 [0.73, 1.20]
**Subtotal (95% CI)**		**171**		**155**	**13.8%**	**0.94 [0.74, 1.20]**
Total events	71		64			
Heterogeneity: Tau² = 0.00; Chi² = 0.05, df = 1 (P = 0.83); I² = 0%						
Test for overall effect: Z = 0.48 (P = 0.63)						
**10.1.3 South-east Asia**						
Dewey 2017	44	151	67	160	9.0%	0.70 [0.51, 0.95]
**Subtotal (95% CI)**		**151**		**160**	**9.0%**	**0.70 [0.51, 0.95]**
Total events	44		67			
Heterogeneity: Not applicable						
Test for overall effect: Z = 2.30 (P = 0.02)						
**Total (95% CI)**		**1746**		**1473**	**100.0%**	**0.84 [0.75, 0.93]**
Total events	983		911			
Heterogeneity: Tau² = 0.01; Chi² = 17.07, df = 7 (P = 0.02); I² = 59%						
Test for overall effect: Z = 3.15 (P = 0.002)						
Test for subgroup differences: Chi² = 2.35, df = 2 (P = 0.31), I² = 15.0%						

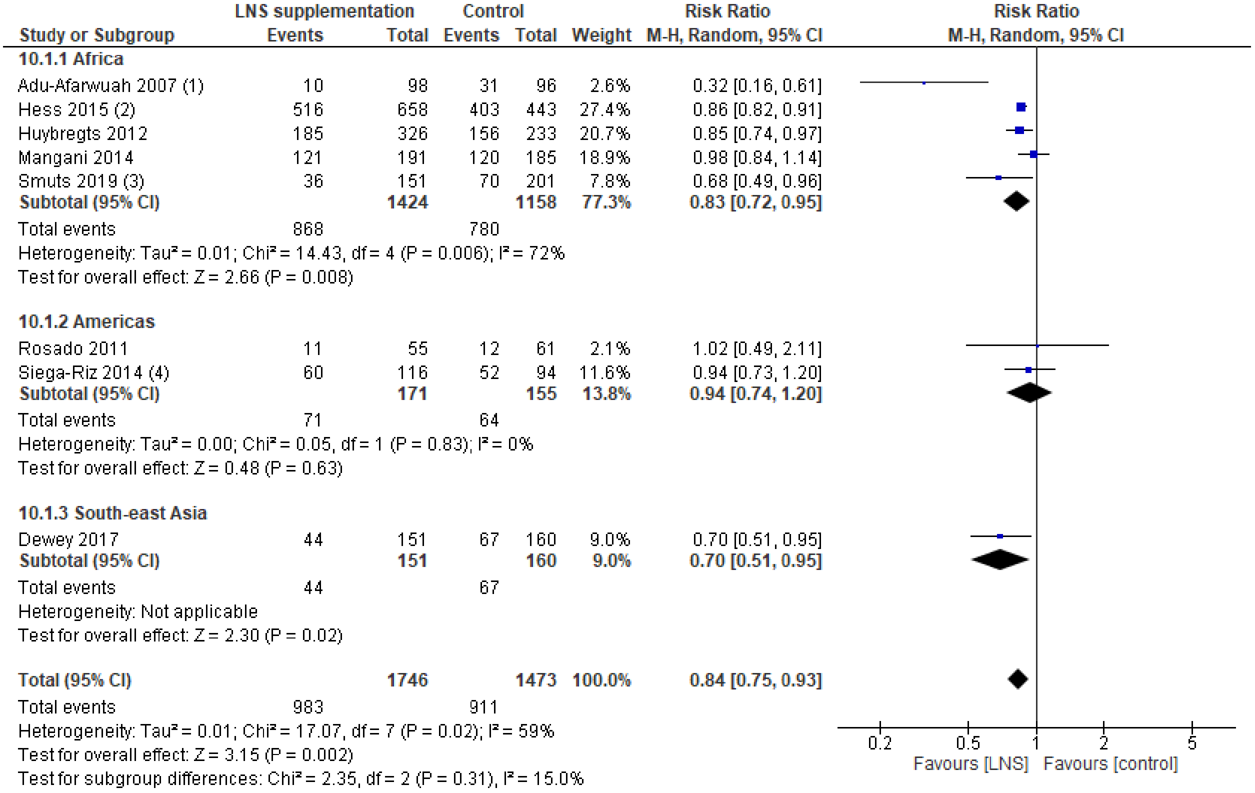

Footnotes
(1) Hemoglobin concentration <100g/L
(2) LNS (Zn 5mg) and LNS (Zn 10mg) groups were combined into one group
(3) SQ LNS group only
(4) Hemoglobin concentration <100 g/L for <12 month olds, <110 g/L for 12-24 month olds, and <111 g/l for 2-5 year olds

Stunting: WHO region

Study or Subgroup	log[Risk Ratio]	SE	Weight	Risk Ratio IV, Random, 95% CI
**10.3.1 Africa**				
Adu-Afarwuah 2016	-0.5271	0.2145	5.4%	0.59 [0.39, 0.90]
Ashorn 2015	0.1454	0.1304	10.8%	1.16 [0.90, 1.49]
Hess 2015 (1)	-0.2699	0.0864	16.0%	0.76 [0.64, 0.90]
Null 2018	-0.1	0.07	18.4%	0.90 [0.79, 1.04]
Smuts 2019 (2)	0.1227	0.135	10.4%	1.13 [0.87, 1.47]
**Subtotal (95% CI)**			**61.0%**	**0.90 [0.75, 1.09]**
Heterogeneity: Tau² = 0.03; Chi² = 14.03, df = 4 (P = 0.007); I² = 71%				
Test for overall effect: Z = 1.08 (P = 0.28)				
**10.3.2 South-east Asia**				
Christian 2015 (3)	-0.0937	0.0561	20.5%	0.91 [0.82, 1.02]
Luby 2018	-0.2	0.07	18.4%	0.82 [0.71, 0.94]
**Subtotal (95% CI)**			**39.0%**	**0.87 [0.79, 0.96]**
Heterogeneity: Tau² = 0.00; Chi² = 1.40, df = 1 (P = 0.24); I² = 29%				
Test for overall effect: Z = 2.64 (P = 0.008)				
**Total (95% CI)**			**100.0%**	**0.89 [0.80, 0.99]**
Heterogeneity: Tau² = 0.01; Chi² = 15.58, df = 6 (P = 0.02); I² = 61%				
Test for overall effect: Z = 2.10 (P = 0.04)				
Test for subgroup differences: Chi² = 0.11, df = 1 (P = 0.74), I² = 0%				

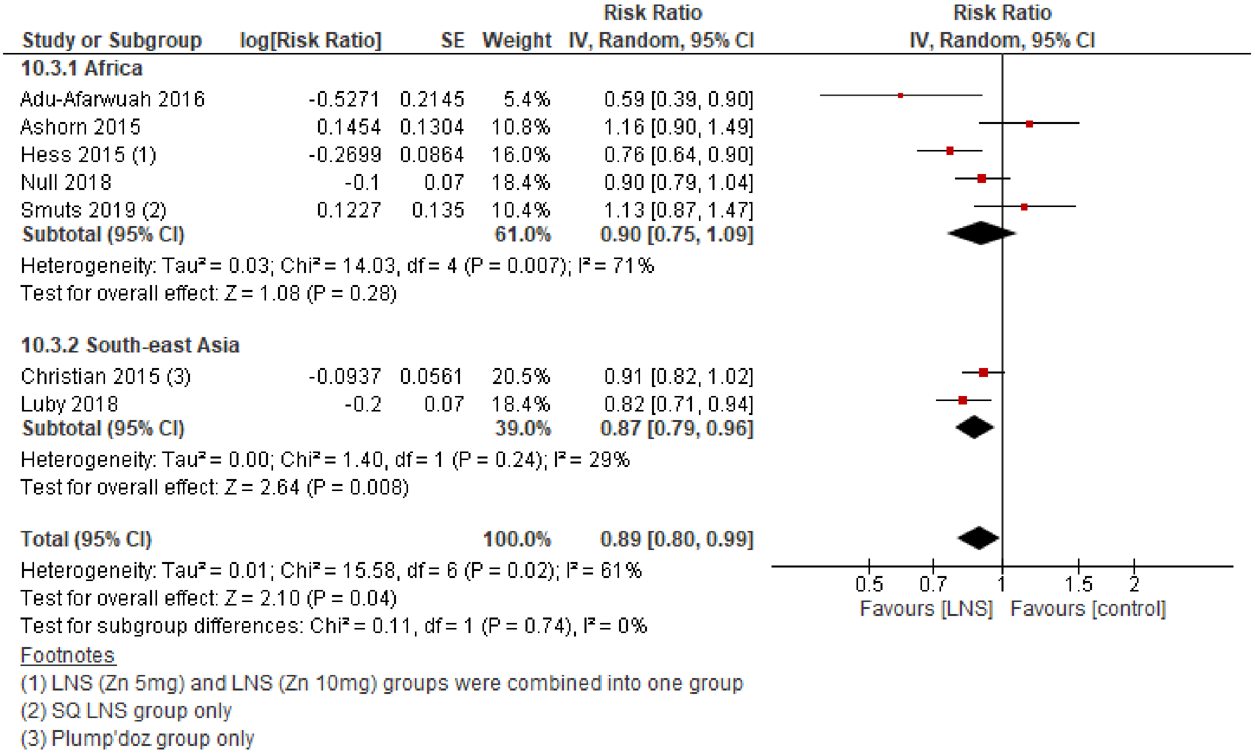

Footnotes
(1) LNS (Zn 5mg) and LNS (Zn 10mg) groups were combined into one group
(2) SQ LNS group only
(3) Plump'doz group only

Wasting: WHO region

Study or Subgroup	LNS supplementation Events	LNS supplementation Total	Control Events	Control Total	Weight	Risk Ratio M-H, Random, 95% CI
**10.5.1 Africa**						
Adu-Afarwuah 2016	22	347	31	337	2.8%	0.69 [0.41, 1.17]
Ashorn 2015	7	214	8	220	0.8%	0.90 [0.33, 2.44]
Hess 2015 (1)	72	811	60	523	6.6%	0.77 [0.56, 1.07]
Null 2018	695	811	1536	1864	39.6%	1.04 [1.00, 1.08]
**Subtotal (95% CI)**		**2183**		**2944**	**49.7%**	**0.88 [0.67, 1.16]**
Total events	796		1635			
Heterogeneity: Tau² = 0.04; Chi² = 7.38, df = 3 (P = 0.06); I² = 59%						
Test for overall effect: Z = 0.90 (P = 0.37)						
**10.5.2 South-east Asia**						
Christian 2015 (2)	123	896	138	843	11.7%	0.84 [0.67, 1.05]
Luby 2018	567	699	1104	1302	38.5%	0.96 [0.92, 1.00]
**Subtotal (95% CI)**		**1595**		**2145**	**50.3%**	**0.93 [0.82, 1.05]**
Total events	690		1242			
Heterogeneity: Tau² = 0.01; Chi² = 1.76, df = 1 (P = 0.18); I² = 43%						
Test for overall effect: Z = 1.15 (P = 0.25)						
**Total (95% CI)**		**3778**		**5089**	**100.0%**	**0.95 [0.87, 1.04]**
Total events	1486		2877			
Heterogeneity: Tau² = 0.01; Chi² = 18.77, df = 5 (P = 0.002); I² = 73%						
Test for overall effect: Z = 1.09 (P = 0.28)						
Test for subgroup differences: Chi² = 0.11, df = 1 (P = 0.74), I² = 0%						

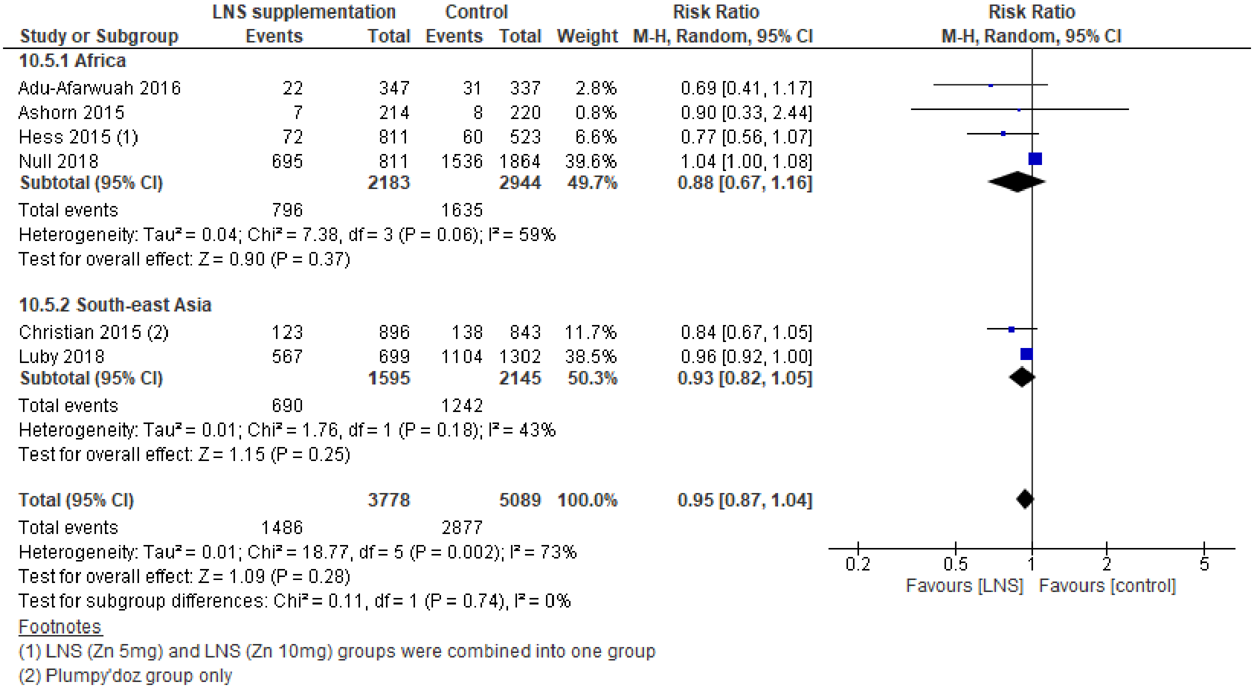

Footnotes
(1) LNS (Zn 5mg) and LNS (Zn 10mg) groups were combined into one group
(2) Plumpy'doz group only

Underweight: WHO region

Study or Subgroup	LNS supplementation Events	LNS supplementation Total	Control Events	Control Total	Weight	Risk Ratio M-H, Random, 95% CI
**10.6.1 Africa**						
Adu-Afarwuah 2016	37	347	55	337	6.3%	0.65 [0.44, 0.96]
Ashorn 2015	28	214	22	220	3.8%	1.31 [0.77, 2.21]
Hess 2015 (1)	154	811	137	523	14.5%	0.72 [0.59, 0.89]
Null 2018	698	811	1548	1864	27.5%	1.04 [1.00, 1.07]
**Subtotal (95% CI)**		**2183**		**2944**	**52.2%**	**0.88 [0.64, 1.21]**
Total events	917		1762			
Heterogeneity: Tau² = 0.08; Chi² = 24.17, df = 3 (P < 0.0001); I² = 88%						
Test for overall effect: Z = 0.78 (P = 0.43)						
**10.6.2 South-east Asia**						
Christian 2015 (2)	292	896	330	843	20.7%	0.83 [0.73, 0.94]
Luby 2018	573	699	1121	1302	27.2%	0.95 [0.91, 0.99]
**Subtotal (95% CI)**		**1595**		**2145**	**47.8%**	**0.90 [0.77, 1.05]**
Total events	865		1451			
Heterogeneity: Tau² = 0.01; Chi² = 5.62, df = 1 (P = 0.02); I² = 82%						
Test for overall effect: Z = 1.34 (P = 0.18)						
**Total (95% CI)**		**3778**		**5089**	**100.0%**	**0.90 [0.81, 1.01]**
Total events	1782		3213			
Heterogeneity: Tau² = 0.01; Chi² = 41.29, df = 5 (P < 0.00001); I² = 88%						
Test for overall effect: Z = 1.85 (P = 0.06)						
Test for subgroup differences: Chi² = 0.01, df = 1 (P = 0.91), I² = 0%						

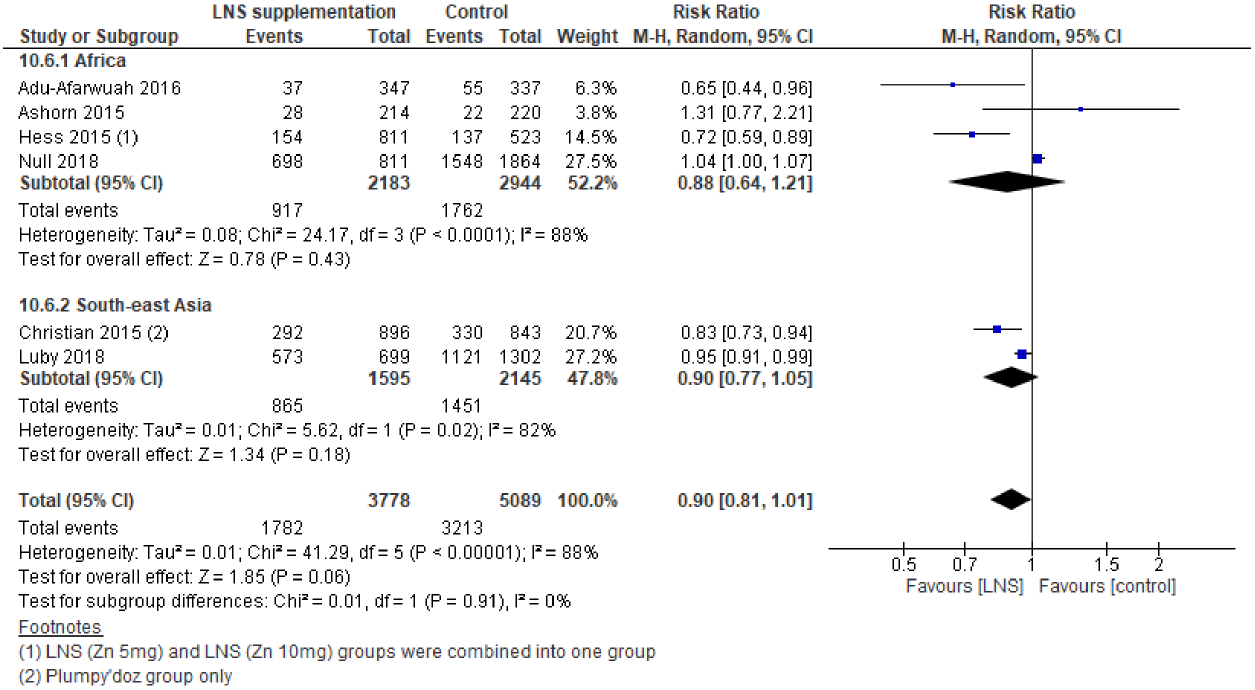

Footnotes
(1) LNS (Zn 5mg) and LNS (Zn 10mg) groups were combined into one group
(2) Plumpy'doz group only

Comparison 8: Targeted Fortification (with MMN) vs. Placebo/No Intervention (Efficacy)

Height (cm)

Study or Subgroup	Fortification Mean	Fortification SD	Fortification Total	Control Mean	Control SD	Control Total	Weight	Mean Difference IV, Random, 95% CI
Christian 2015	1.58	0.555	513	1.57	0.7113	843	94.8%	0.01 [-0.06, 0.08]
Faber 2005	7	1.8148	143	7.1	1.2184	145	3.4%	-0.10 [-0.46, 0.26]
Lartey 1999	7	1.4	47	7	1.2	50	1.6%	0.00 [-0.52, 0.52]
Oelofse 2003	74.4	1.8	16	74.5	3.1	14	0.1%	-0.10 [-1.95, 1.75]
**Total (95% CI)**			**719**			**1052**	**100.0%**	**0.01 [-0.06, 0.07]**
Heterogeneity: Tau² = 0.00; Chi² = 0.36, df = 3 (P = 0.95); I² = 0%								
Test for overall effect: Z = 0.18 (P = 0.86)								

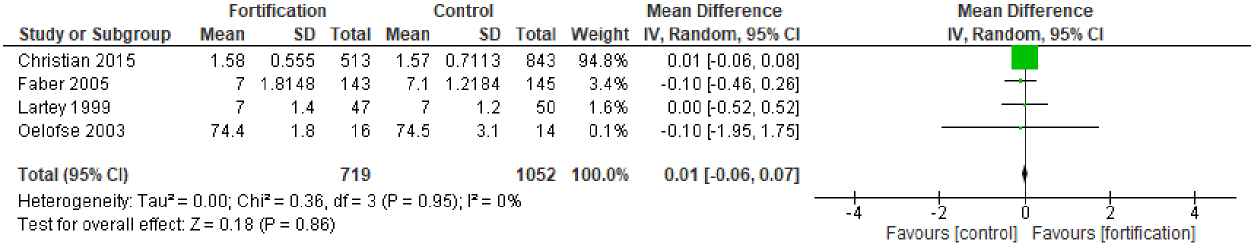

Weight (kg)

Study or Subgroup	Fortification Mean	Fortification SD	Fortification Total	Control Mean	Control SD	Control Total	Weight	Mean Difference IV, Random, 95% CI
Christian 2015	0.26	0.2775	513	0.24	0.3557	843	95.8%	0.02 [-0.01, 0.05]
Faber 2005	1.6	1.2099	143	1.6	1.2184	145	1.4%	0.00 [-0.28, 0.28]
Lartey 1999	1.3	0.5	47	1.2	0.6	50	2.3%	0.10 [-0.12, 0.32]
Oelofse 2003	9.6	1	16	9.9	1.8	14	0.1%	-0.30 [-1.36, 0.76]
Owino 2007	9	1.5	37	8.6	1.1	69	0.4%	0.40 [-0.15, 0.95]
**Total (95% CI)**			**756**			**1121**	**100.0%**	**0.02 [-0.01, 0.06]**
Heterogeneity: Tau² = 0.00; Chi² = 2.70, df = 4 (P = 0.61); I² = 0%								
Test for overall effect: Z = 1.33 (P = 0.18)								

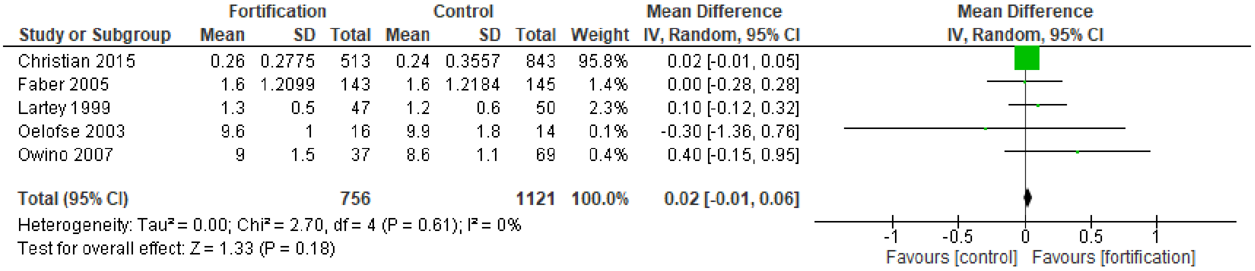

Length-for-age (z-score)

Study or Subgroup	Fortification Mean	Fortification SD	Fortification Total	Control Mean	Control SD	Control Total	Weight	Mean Difference IV, Random, 95% CI
Christian 2015	-1.87	1	513	-1.91	1.02	843	40.6%	0.04 [-0.07, 0.15]
Faber 2005	-0.94	1.05	143	-1.06	1.15	145	15.4%	0.12 [-0.13, 0.37]
Ma 2016	-1.83	0.95	140	-1.67	0.97	133	18.1%	-0.16 [-0.39, 0.07]
Oelofse 2003	-0.94	0.7	16	-0.72	1.1	14	2.8%	-0.22 [-0.89, 0.45]
Owino 2007	0.1	0.9	37	-0.2	0.9	69	8.7%	0.30 [-0.06, 0.66]
Sazawal 2014	-0.05	0.49	27	-0.21	0.47	24	14.5%	0.16 [-0.10, 0.42]
**Total (95% CI)**			**876**			**1228**	**100.0%**	**0.05 [-0.06, 0.16]**

Heterogeneity: Tau² = 0.01; Chi² = 6.71, df = 5 (P = 0.24); I² = 26%
Test for overall effect: Z = 0.84 (P = 0.40)

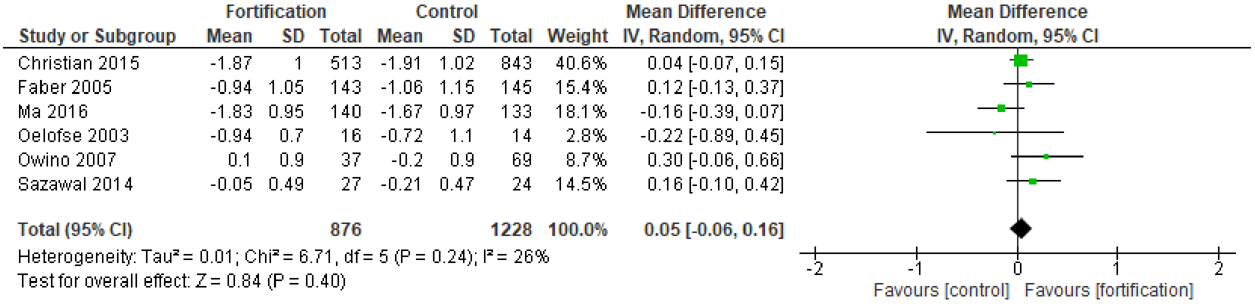

Weight-for-age (z-score)

Study or Subgroup	Fortification Mean	Fortification SD	Fortification Total	Control Mean	Control SD	Control Total	Weight	Mean Difference IV, Random, 95% CI
Christian 2015	-0.08	0.2809	513	-0.1	0.3622	843	94.9%	0.02 [-0.01, 0.05]
Faber 2005	0.05	1.25	143	0.09	1.37	145	1.2%	-0.04 [-0.34, 0.26]
Ma 2016	-1.04	0.89	140	-0.91	0.88	133	2.6%	-0.13 [-0.34, 0.08]
Oelofse 2003	-0.55	0.99	16	-0.52	1.6	14	0.1%	-0.03 [-1.00, 0.94]
Owino 2007	0.24	1.2	37	0.09	1.1	69	0.5%	0.15 [-0.32, 0.62]
Sazawal 2014	0.72	0.81	27	0.52	0.67	24	0.7%	0.20 [-0.21, 0.61]
**Total (95% CI)**			**876**			**1228**	**100.0%**	**0.02 [-0.02, 0.05]**

Heterogeneity: Tau² = 0.00; Chi² = 3.15, df = 5 (P = 0.68); I² = 0%
Test for overall effect: Z = 1.01 (P = 0.31)

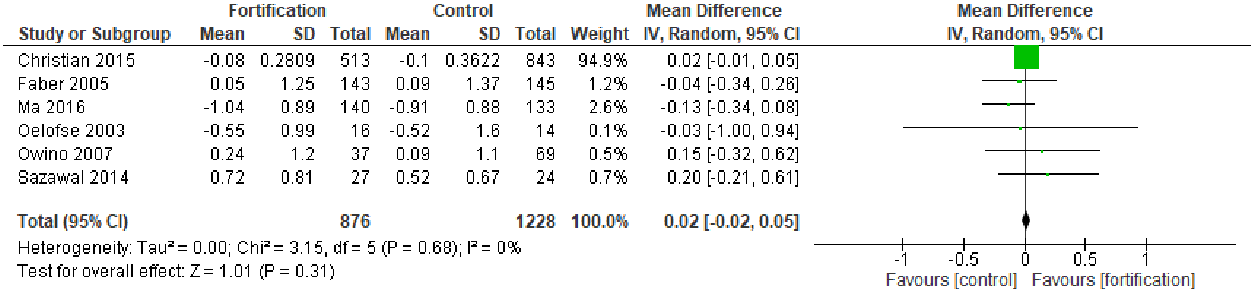

Weight-for-height (z-score)

Study or Subgroup	Fortification Mean	Fortification SD	Fortification Total	Control Mean	Control SD	Control Total	Weight	Mean Difference IV, Random, 95% CI
Christian 2015	-0.2	0.2775	513	-0.22	0.3557	843	95.1%	0.02 [-0.01, 0.05]
Faber 2005	0.82	1.15	143	0.99	1.23	145	1.5%	-0.17 [-0.44, 0.10]
Ma 2016	-0.24	0.84	140	-0.15	0.82	133	2.8%	-0.09 [-0.29, 0.11]
Oelofse 2003	0.11	1.1	16	0.42	1.6	14	0.1%	-0.31 [-1.31, 0.69]
Sazawal 2014	0.79	0.86	27	0.66	0.81	24	0.5%	0.13 [-0.33, 0.59]
**Total (95% CI)**			**839**			**1159**	**100.0%**	**0.01 [-0.02, 0.05]**

Heterogeneity: Tau² = 0.00; Chi² = 3.56, df = 4 (P = 0.47); I² = 0%
Test for overall effect: Z = 0.85 (P = 0.40)

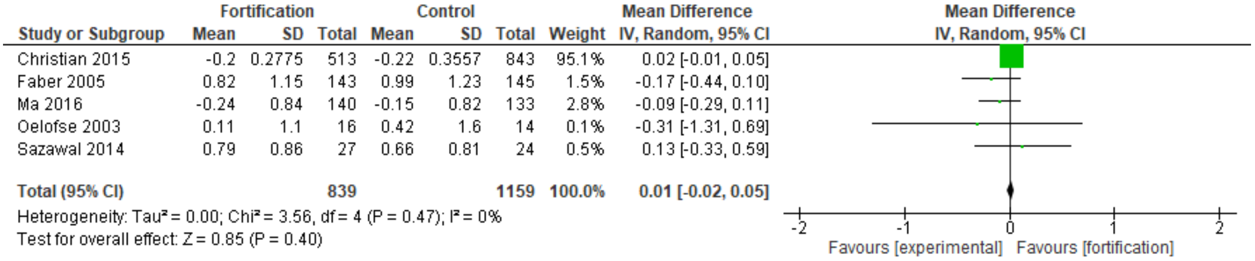

Iron-deficiency anemia

Study or Subgroup	Fortification Events	Fortification Total	Control Events	Control Total	Weight	Risk Ratio M-H, Random, 95% CI
Ma 2016	10	140	22	133	36.1%	0.43 [0.21, 0.88]
Phu 2010	7	133	27	71	33.7%	0.14 [0.06, 0.30]
Shamah-Levy 2008	8	271	11	144	30.2%	0.39 [0.16, 0.94]
**Total (95% CI)**		**544**		**348**	**100.0%**	**0.28 [0.14, 0.59]**
Total events	25		60			

Heterogeneity: Tau² = 0.25; Chi² = 5.08, df = 2 (P = 0.08); I² = 61%
Test for overall effect: Z = 3.39 (P = 0.0007)

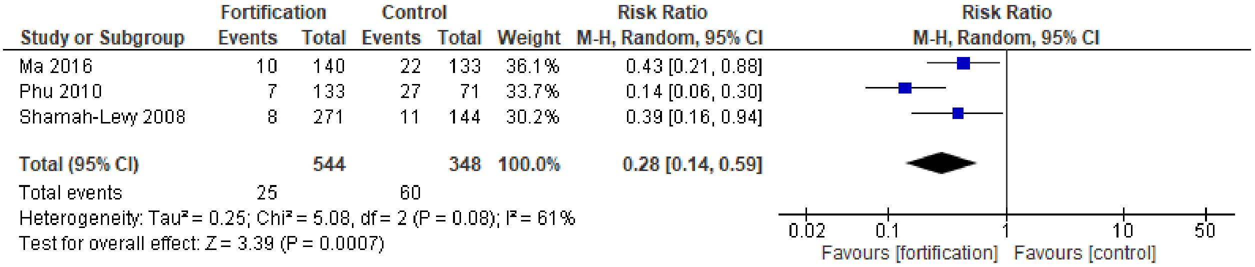

Iron deficiency

Study or Subgroup	Fortification Events	Fortification Total	Control Events	Control Total	Weight	Risk Ratio M-H, Random, 95% CI
Anand 2007	26	93	46	88	25.6%	0.53 [0.36, 0.78]
Glinz 2015 (1)	7	97	13	44	14.5%	0.24 [0.10, 0.57]
Lartey 1999	3	28	17	31	10.3%	0.20 [0.06, 0.60]
Ma 2016	32	140	53	133	26.0%	0.57 [0.40, 0.83]
Phu 2010	19	135	42	73	23.5%	0.24 [0.15, 0.39]
**Total (95% CI)**		**493**		**369**	**100.0%**	**0.36 [0.24, 0.56]**
Total events	87		171			

Heterogeneity: Tau² = 0.16; Chi² = 13.03, df = 4 (P = 0.01); I² = 69%
Test for overall effect: Z = 4.53 (P < 0.00001)

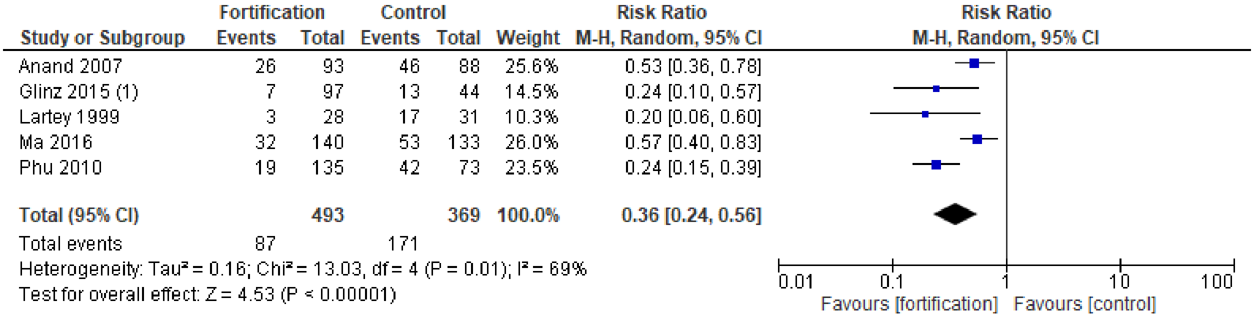

Footnotes
(1) Fortified with iron only

Hemoglobin (g/L)

Study or Subgroup	Fortification Mean	Fortification SD	Fortification Total	Control Mean	Control SD	Control Total	Weight	Mean Difference IV, Random, 95% CI
Anand 2007	121	10	94	110	10	94	10.2%	11.00 [8.14, 13.86]
Arcanjo 2012 (1)	8.6	3.1	15	2.6	2.9	15	10.6%	6.00 [3.85, 8.15]
Faber 2005	119	11	144	110	14	142	10.2%	9.00 [6.08, 11.92]
Glinz 2015 (2)	104.5179	11.9745	97	103	13	44	9.1%	1.52 [-3.00, 6.04]
Lartey 1999	0	14	47	-5	19	48	7.4%	5.00 [-1.70, 11.70]
Ma 2016	121.6	11.7	140	119.5	12.1	133	10.2%	2.10 [-0.73, 4.93]
Oelofse 2003	108	9	16	106	13	14	6.4%	2.00 [-6.11, 10.11]
Owino 2007	105	12	37	98	15	69	8.5%	7.00 [1.76, 12.24]
Phu 2010	7.4947	11.5059	135	2.6	9.9	73	10.1%	4.89 [1.91, 7.88]
Sazawal 2014	12.9	16	30	2.3	13	22	6.6%	10.60 [2.71, 18.49]
Shamah-Lewy 2008	120.2996	9.7342	271	123.6	10.8	144	10.6%	-3.30 [-5.41, -1.19]
**Total (95% CI)**			**1026**			**798**	**100.0%**	**4.97 [1.81, 8.12]**

Heterogeneity: Tau² = 23.25; Chi² = 90.96, df = 10 (P < 0.00001); I² = 89%
Test for overall effect: Z = 3.08 (P = 0.002)

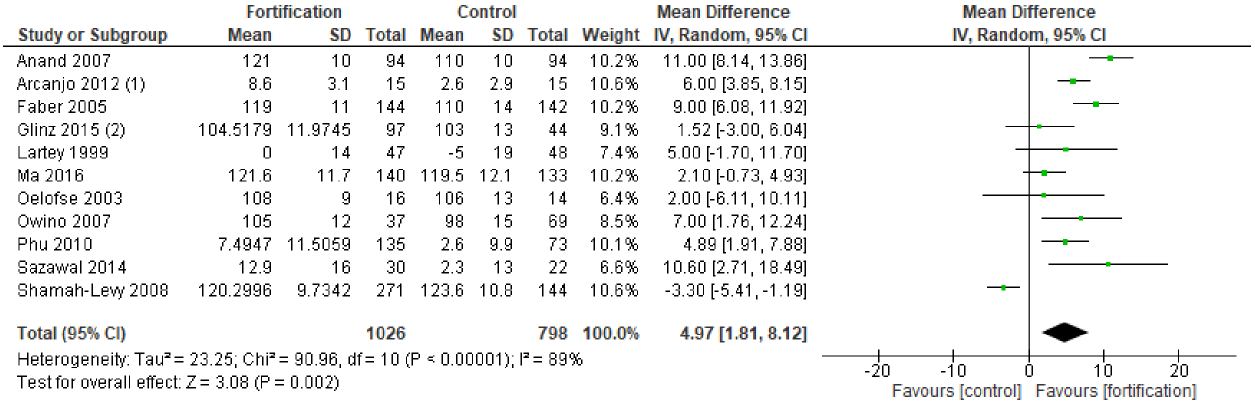

Footnotes
(1) Fortified with iron only
(2) Fortified with iron only

Serum/plasma ferritin (µg/L)

Study or Subgroup	Fortification Mean	Fortification SD	Fortification Total	Control Mean	Control SD	Control Total	Weight	Mean Difference IV, Random, 95% CI
Anand 2007	25.9	2.3	93	11.3	2.6	88	16.3%	14.60 [13.88, 15.32]
Faber 2005	6.9	9.2	141	-2.43	4.96	138	16.2%	9.33 [7.60, 11.06]
Glinz 2015 (1)	73.3073	50.9404	97	57.27	51.7	44	7.5%	16.04 [-2.30, 34.37]
Lartey 1999	22.1	13.1525	28	14.6	14.1765	31	14.0%	7.50 [0.53, 14.47]
Ma 2016	20.93	14.26	140	16.97	13.18	133	15.8%	3.96 [0.70, 7.22]
Phu 2010	-5.7239	26.8184	135	-14.7	24.3	72	13.8%	8.98 [1.77, 16.19]
Shamah-Lewy 2008	21.1539	2.6179	271	20.1	2.1	144	16.4%	1.05 [0.59, 1.52]
**Total (95% CI)**			**905**			**650**	**100.0%**	**8.19 [1.35, 15.03]**

Heterogeneity: Tau² = 74.36; Chi² = 993.51, df = 6 (P < 0.00001); I² = 99%
Test for overall effect: Z = 2.35 (P = 0.02)

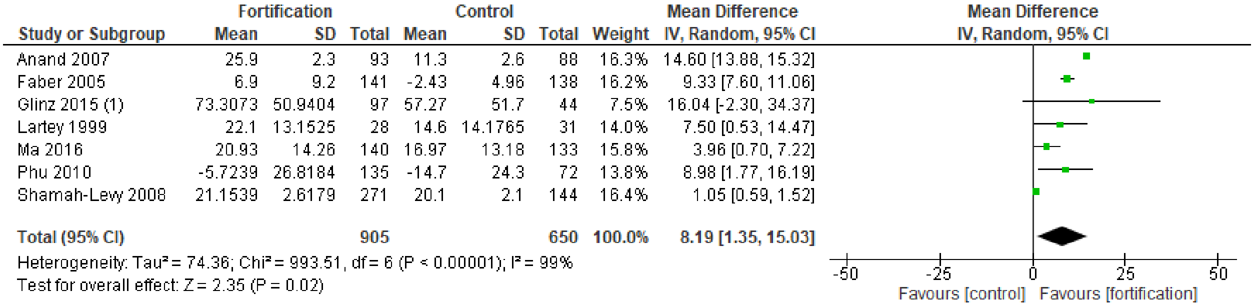

Footnotes
(1) Fortified with iron only

Serum/plasma retinol (µg/dL)

Study or Subgroup	Fortification Mean	Fortification SD	Fortification Total	Control Mean	Control SD	Control Total	Weight	Mean Difference IV, Random, 95% CI
Anand 2007	33.3	9.7	86	31.8	10.6	87	20.3%	1.50 [-1.53, 4.53]
Faber 2005	28.0161	10.5346	139	28.7789	8.129	133	24.0%	-0.76 [-2.99, 1.47]
Lartey 1999	3.92	8.4	29	-2.24	8.4	37	15.8%	6.16 [2.08, 10.24]
Oelofse 2003	26.8	5.8	16	21.4	5.7	14	15.7%	5.40 [1.28, 9.52]
Phu 2010	5.434	6.7066	135	3.92	8.12	73	24.3%	1.51 [-0.67, 3.69]
**Total (95% CI)**			**405**			**344**	**100.0%**	**2.31 [-0.02, 4.63]**

Heterogeneity: Tau² = 4.57; Chi² = 12.38, df = 4 (P = 0.01); I² = 68%
Test for overall effect: Z = 1.94 (P = 0.05)

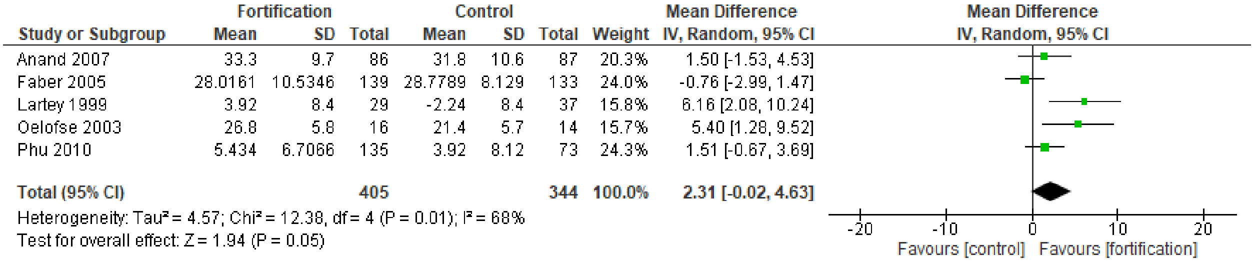

Serum/plasma zinc (µg/dL)

Study or Subgroup	Fortification Mean	SD	Total	Control Mean	SD	Total	Weight	Mean Difference IV, Random, 95% CI
Faber 2005	69.3	13.73	140	69.3	11.77	132	32.0%	0.00 [-3.03, 3.03]
Lartey 1999	-7.1918	30.07	30	1.3076	39.88	31	4.0%	-8.50 [-26.19, 9.19]
Lo 2011 (1)	62.2	2.2	29	63.3	1.9	32	39.4%	-1.10 [-2.14, -0.06]
Oelofse 2003	85	9.1	16	73.6	12.1	14	14.7%	11.40 [3.65, 19.15]
Phu 2010	12.8113	33.2498	133	7.19	36.61	72	10.0%	5.62 [-4.55, 15.79]
**Total (95% CI)**			**348**			**281**	**100.0%**	**1.46 [-2.25, 5.17]**

Heterogeneity: Tau² = 8.81; Chi² = 12.36, df = 4 (P = 0.01); I² = 68%
Test for overall effect: Z = 0.77 (P = 0.44)

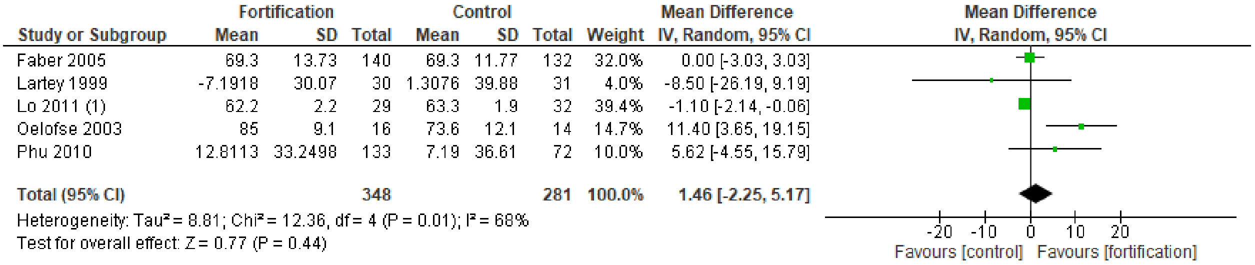

Footnotes
(1) Fortified with zinc only

Comparison 9a: Large-Scale Food Fortification (with MMN) vs. Placebo/No Intervention (Efficacy)

Hemoglobin (g/dL)

Study or Subgroup	Fortification Mean	SD	Total	Control Mean	SD	Total	Weight	Mean Difference IV, Random, 95% CI
Le Port 2017	10.62	1.37	191	10.38	1.4	232	24.8%	0.24 [-0.02, 0.50]
Lucia 2017	12.54	0.88	49	12.57	0.95	50	23.4%	-0.03 [-0.39, 0.33]
Sazawal 2007	10.9	1.14	233	9.54	1.54	232	25.1%	1.36 [1.11, 1.61]
Villalpando 2006	12.74	0.05	58	12.41	0.059	57	26.7%	0.33 [0.31, 0.35]
**Total (95% CI)**			**531**			**571**	**100.0%**	**0.48 [-0.01, 0.97]**

Heterogeneity: Tau² = 0.23; Chi² = 71.16, df = 3 (P < 0.00001); I² = 96%
Test for overall effect: Z = 1.93 (P = 0.05)

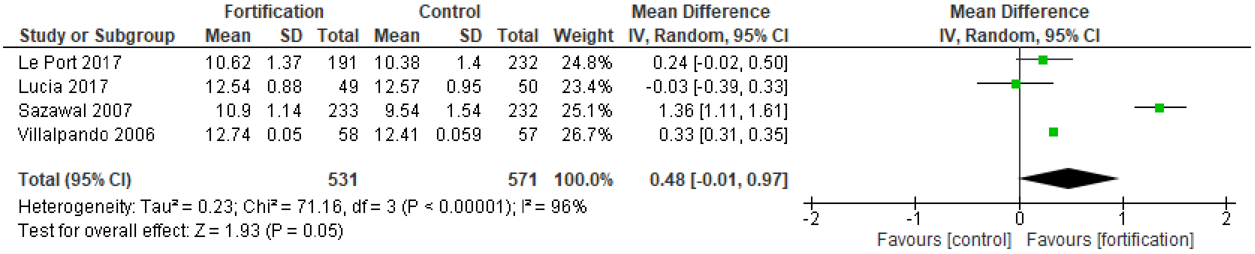

Serum/plasma ferritin (µg/L)

Study or Subgroup	Fortification Mean	SD	Total	Control Mean	SD	Total	Weight	Mean Difference IV, Random, 95% CI
Lucia 2017	19.72	5.08	49	17.58	4.23	50	32.2%	2.14 [0.30, 3.98]
Sazawal 2007	15.3	11.2	233	9.8	8.6	232	32.4%	5.50 [3.69, 7.31]
Villalpando 2006	2.4564	2.937	58	1.5175	3.5155	57	35.4%	0.94 [-0.25, 2.12]
**Total (95% CI)**			**340**			**339**	**100.0%**	**2.80 [0.11, 5.50]**

Heterogeneity: Tau² = 4.98; Chi² = 17.04, df = 2 (P = 0.0002); I² = 88%
Test for overall effect: Z = 2.04 (P = 0.04)

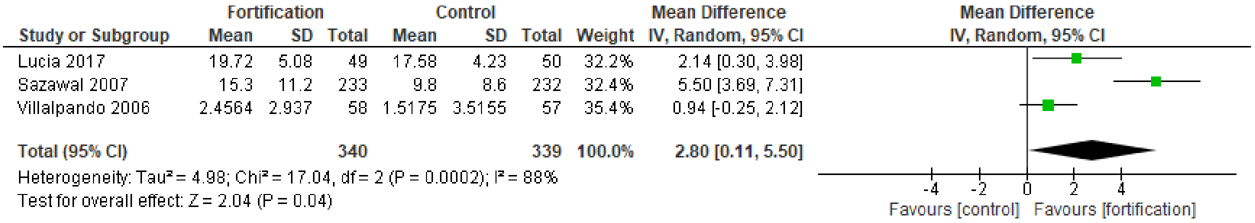

Serum/plasma zinc (µg/dL)

Study or Subgroup	Fortification Mean	SD	Total	Control Mean	SD	Total	Weight	Mean Difference IV, Random, 95% CI
Lucia 2017	110.21	22.76	49	92.13	15.92	50	30.2%	18.08 [10.33, 25.83]
Sazawal 2007	61.4	26.8	233	63.4	29.4	232	33.6%	-2.00 [-7.11, 3.11]
Villalpando 2006	82.44	6.28	58	85.45	6.54	57	36.2%	-3.01 [-5.35, -0.67]
**Total (95% CI)**			**340**			**339**	**100.0%**	**3.69 [-6.27, 13.66]**

Heterogeneity: Tau² = 70.01; Chi² = 26.13, df = 2 (P < 0.00001); I² = 92%
Test for overall effect: Z = 0.73 (P = 0.47)

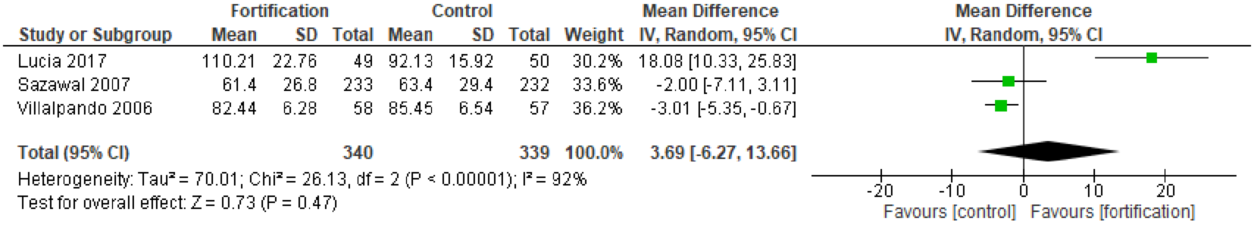

Comparison 9b: Large-Scale Food fortification (with Iron) vs. Placebo/No Intervention (Efficacy)

Hemoglobin (g/dL)

Study or Subgroup	Fortification Mean	SD	Total	Control Mean	SD	Total	Weight	Mean Difference IV, Random, 95% CI
Bagni 2009	11.75	1.16	180	11.51	1.16	174	24.5%	0.24 [-0.00, 0.48]
Barbosa 2012	12.6	1.1	88	12.3	1.1	85	22.4%	0.30 [-0.03, 0.63]
Chen 2005	11.8185	0.9135	202	11.4033	0.8874	126	25.5%	0.42 [0.22, 0.61]
Nestel 2004	0.2006	0.2754	559	0.3659	0.2895	311	27.6%	-0.17 [-0.20, -0.13]
**Total (95% CI)**			**1029**			**696**	**100.0%**	**0.19 [-0.17, 0.54]**

Heterogeneity: Tau² = 0.12; Chi² = 47.31, df = 3 (P < 0.00001); I² = 94%
Test for overall effect: Z = 1.03 (P = 0.30)

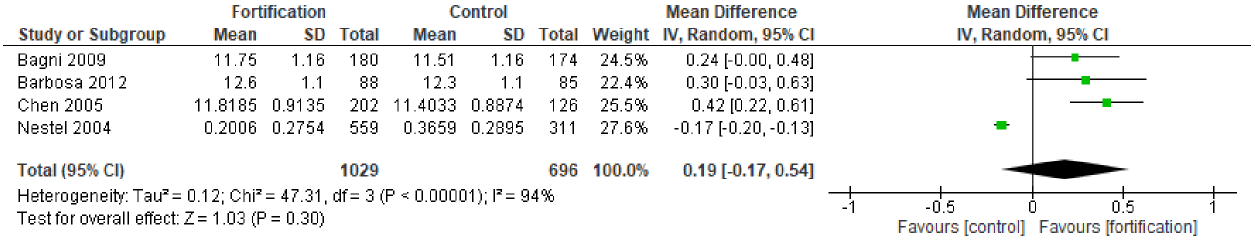

Comparison 10: Micronutrient Powder Supplementation vs. Placebo/No Intervention (Effectiveness)

Hemoglobin (g/L)

Study or Subgroup	Mean Difference	SE	Weight	Mean Difference IV, Random, 95% CI
Attanasio 2014	0.272	0.9587	11.3%	0.27 [-1.61, 2.15]
Baum 2017	2.8	1.4	7.2%	2.80 [0.06, 5.54]
Larson 2018	0	0.6224	16.0%	0.00 [-1.22, 1.22]
Lopez 2014 (1)	2.24	0.53	17.4%	2.24 [1.20, 3.28]
Luo 2017	0.127	1.0087	10.8%	0.13 [-1.85, 2.10]
Menon 2007	3.9	2.0374	4.1%	3.90 [-0.09, 7.89]
Samuel 2018 (2)	3.2	1.6546	5.7%	3.20 [-0.04, 6.44]
Somasse 2018	0.41	0.138	22.3%	0.41 [0.14, 0.68]
Suchdev 2012	4	1.7623	5.2%	4.00 [0.55, 7.45]
**Total (95% CI)**			**100.0%**	**1.28 [0.38, 2.17]**

Heterogeneity: Tau² = 0.91; Chi² = 23.92, df = 8 (P = 0.002); I² = 67%
Test for overall effect: Z = 2.80 (P = 0.005)

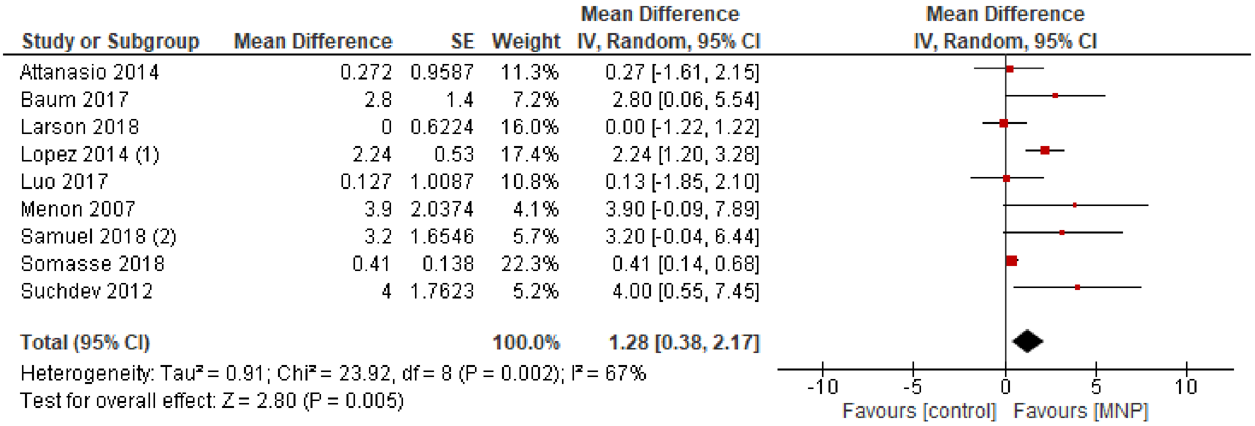

Footnotes
(1) Controlled before-after study
(2) Quasi experimental matched control design

Comparison 10: Subgroup Analyses for Micronutrient Powders vs. Placebo/No Intervention (Effectiveness)

Anemia: WHO region:

Study or Subgroup	log[Risk Ratio]	SE	Weight	Risk Ratio IV, Random, 95% CI
**4.1.1 Africa**				
Samuel 2018 (1)	-0.1719	0.2051	4.1%	0.84 [0.56, 1.26]
Somasse 2018	-0.1478	0.5298	0.7%	0.86 [0.31, 2.44]
Suchdev 2012	-0.3197	0.1718	5.5%	0.73 [0.52, 1.02]
**Subtotal (95% CI)**			**10.3%**	**0.78 [0.60, 1.00]**
Heterogeneity: Tau² = 0.00; Chi² = 0.35, df = 2 (P = 0.84); I² = 0%				
Test for overall effect: Z = 1.97 (P = 0.05)				
**4.1.2 Americas**				
Attanasio 2014	-0.3901	0.2363	3.2%	0.68 [0.43, 1.08]
Baum 2017	-0.4463	0.16	6.2%	0.64 [0.47, 0.88]
Lopez 2014 (2)	-0.041	0.016	27.6%	0.96 [0.93, 0.99]
Menon 2007 (3)	-0.868	0.2159	3.7%	0.42 [0.27, 0.64]
**Subtotal (95% CI)**			**40.7%**	**0.67 [0.45, 0.98]**
Heterogeneity: Tau² = 0.13; Chi² = 22.87, df = 3 (P < 0.0001); I² = 87%				
Test for overall effect: Z = 2.04 (P = 0.04)				
**4.1.3 South-East Asia**				
Larson 2018	-0.0198	0.0324	24.9%	0.98 [0.92, 1.04]
**Subtotal (95% CI)**			**24.9%**	**0.98 [0.92, 1.04]**
Heterogeneity: Not applicable				
Test for overall effect: Z = 0.61 (P = 0.54)				
**4.1.4 Western Pacific**				
Luo 2017	-0.0001	0.0362	24.1%	1.00 [0.93, 1.07]
**Subtotal (95% CI)**			**24.1%**	**1.00 [0.93, 1.07]**
Heterogeneity: Not applicable				
Test for overall effect: Z = 0.00 (P = 1.00)				
**Total (95% CI)**			**100.0%**	**0.89 [0.82, 0.97]**
Heterogeneity: Tau² = 0.01; Chi² = 28.06, df = 8 (P = 0.0005); I² = 71%				
Test for overall effect: Z = 2.55 (P = 0.01)				
Test for subgroup differences: Chi² = 7.33, df = 3 (P = 0.06), I² = 59.1%				

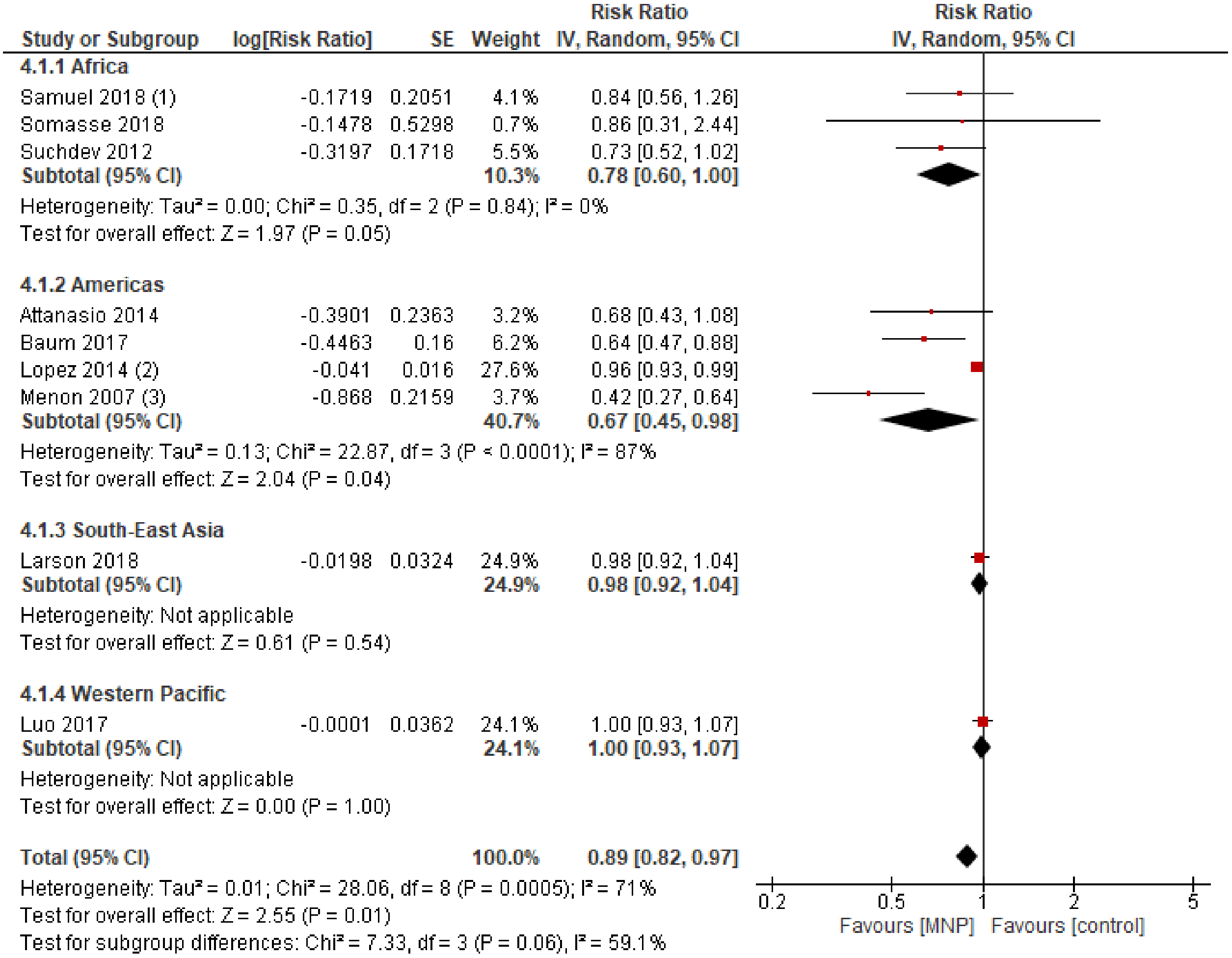

Footnotes
(1) Quasi experimental matched control design
(2) Controlled before-after study
(3) Both groups received iron-fortified wheat-soy blend

Anemia: intervention duration

Study or Subgroup	log[Risk Ratio]	SE	Weight	Risk Ratio IV, Random, 95% CI
**4.2.1 Less than 3 months**				
Lopez 2014 (1)	-0.041	0.016	27.6%	0.96 [0.93, 0.99]
Menon 2007 (2)	-0.868	0.2159	3.7%	0.42 [0.27, 0.64]
**Subtotal (95% CI)**			**31.4%**	**0.65 [0.29, 1.47]**
Heterogeneity: Tau² = 0.32; Chi² = 14.59, df = 1 (P = 0.0001); I² = 93%				
Test for overall effect: Z = 1.03 (P = 0.30)				
**4.2.2 3-5 months**				
Baum 2017	-0.4463	0.16	6.1%	0.64 [0.47, 0.88]
Somasse 2018	-0.1478	0.5298	0.7%	0.86 [0.31, 2.44]
**Subtotal (95% CI)**			**6.8%**	**0.66 [0.49, 0.89]**
Heterogeneity: Tau² = 0.00; Chi² = 0.29, df = 1 (P = 0.59); I² = 0%				
Test for overall effect: Z = 2.75 (P = 0.006)				
**4.2.3 12-17 months**				
Suchdev 2012	-0.3197	0.1718	5.5%	0.73 [0.52, 1.02]
**Subtotal (95% CI)**			**5.5%**	**0.73 [0.52, 1.02]**
Heterogeneity: Not applicable				
Test for overall effect: Z = 1.86 (P = 0.06)				
**4.2.4 18 months and above**				
Attanasio 2014	-0.3901	0.2363	3.2%	0.68 [0.43, 1.08]
Larson 2018	-0.0198	0.0324	24.9%	0.98 [0.92, 1.04]
Luo 2017	-0.001	0.0357	24.2%	1.00 [0.93, 1.07]
**Subtotal (95% CI)**			**52.3%**	**0.98 [0.93, 1.04]**
Heterogeneity: Tau² = 0.00; Chi² = 2.70, df = 2 (P = 0.26); I² = 26%				
Test for overall effect: Z = 0.56 (P = 0.57)				
**4.2.5 6-11 months**				
Samuel 2018 (3)	-0.1719	0.2051	4.1%	0.84 [0.56, 1.26]
**Subtotal (95% CI)**			**4.1%**	**0.84 [0.56, 1.26]**
Heterogeneity: Not applicable				
Test for overall effect: Z = 0.84 (P = 0.40)				
**Total (95% CI)**			**100.0%**	**0.89 [0.82, 0.97]**
Heterogeneity: Tau² = 0.01; Chi² = 28.03, df = 8 (P = 0.0005); I² = 71%				
Test for overall effect: Z = 2.55 (P = 0.01)				
Test for subgroup differences: Chi² = 10.63, df = 4 (P = 0.03), I² = 62.4%				

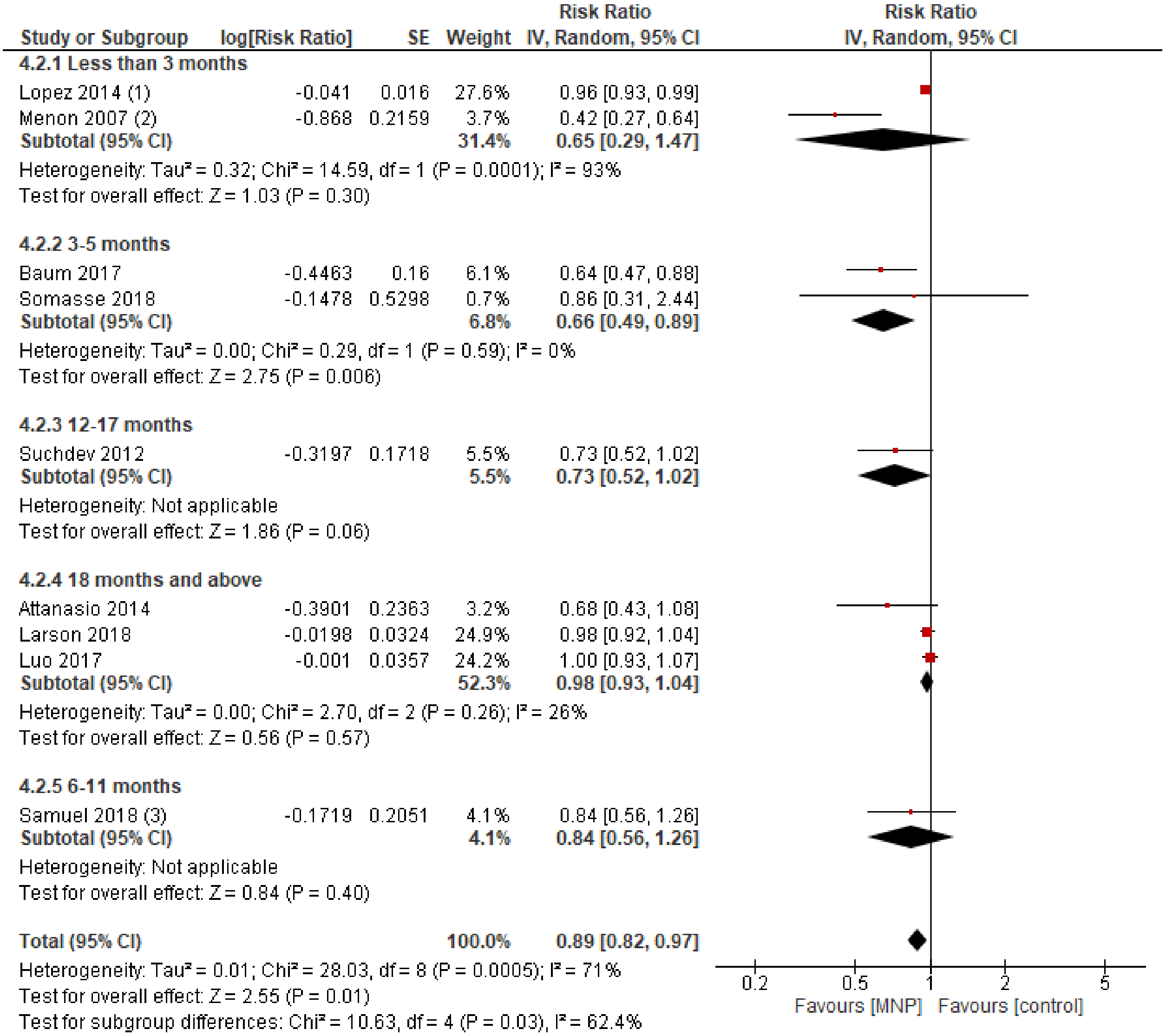

Footnotes
(1) Controlled before-after study
(2) Both groups received iron-fortified wheat-soy blend
(3) Quasi experimental matched control design

Anemia: nutritional status at baseline (anemic vs. non anemic)

Study or Subgroup	log[Risk Ratio]	SE	Weight	Risk Ratio IV, Random, 95% CI
**4.3.1 Non-anemic at baseline**				
Attanasio 2014	-0.3901	0.2363	3.2%	0.68 [0.43, 1.08]
Lopez 2014 (1)	-0.041	0.016	27.6%	0.96 [0.93, 0.99]
Samuel 2018 (2)	-0.1719	0.2051	4.1%	0.84 [0.56, 1.26]
**Subtotal (95% CI)**			**34.9%**	**0.92 [0.79, 1.06]**
Heterogeneity: Tau² = 0.01; Chi² = 2.57, df = 2 (P = 0.28); I² = 22%				
Test for overall effect: Z = 1.17 (P = 0.24)				
**4.3.2 Anemic at baseline**				
Baum 2017	-0.4463	0.16	6.2%	0.64 [0.47, 0.88]
Larson 2018	-0.0198	0.0324	24.9%	0.98 [0.92, 1.04]
Luo 2017	-0.0001	0.0362	24.1%	1.00 [0.93, 1.07]
Menon 2007 (3)	-0.868	0.2159	3.7%	0.42 [0.27, 0.64]
Somasse 2018	-0.1478	0.5298	0.7%	0.86 [0.31, 2.44]
Suchdev 2012	-0.3197	0.1718	5.5%	0.73 [0.52, 1.02]
**Subtotal (95% CI)**			**65.1%**	**0.82 [0.70, 0.96]**
Heterogeneity: Tau² = 0.02; Chi² = 25.43, df = 5 (P = 0.0001); I² = 80%				
Test for overall effect: Z = 2.47 (P = 0.01)				
**Total (95% CI)**			**100.0%**	**0.89 [0.82, 0.97]**
Heterogeneity: Tau² = 0.01; Chi² = 28.06, df = 8 (P = 0.0005); I² = 71%				
Test for overall effect: Z = 2.55 (P = 0.01)				
Test for subgroup differences: Chi² = 1.04, df = 1 (P = 0.31), I² = 3.5%				

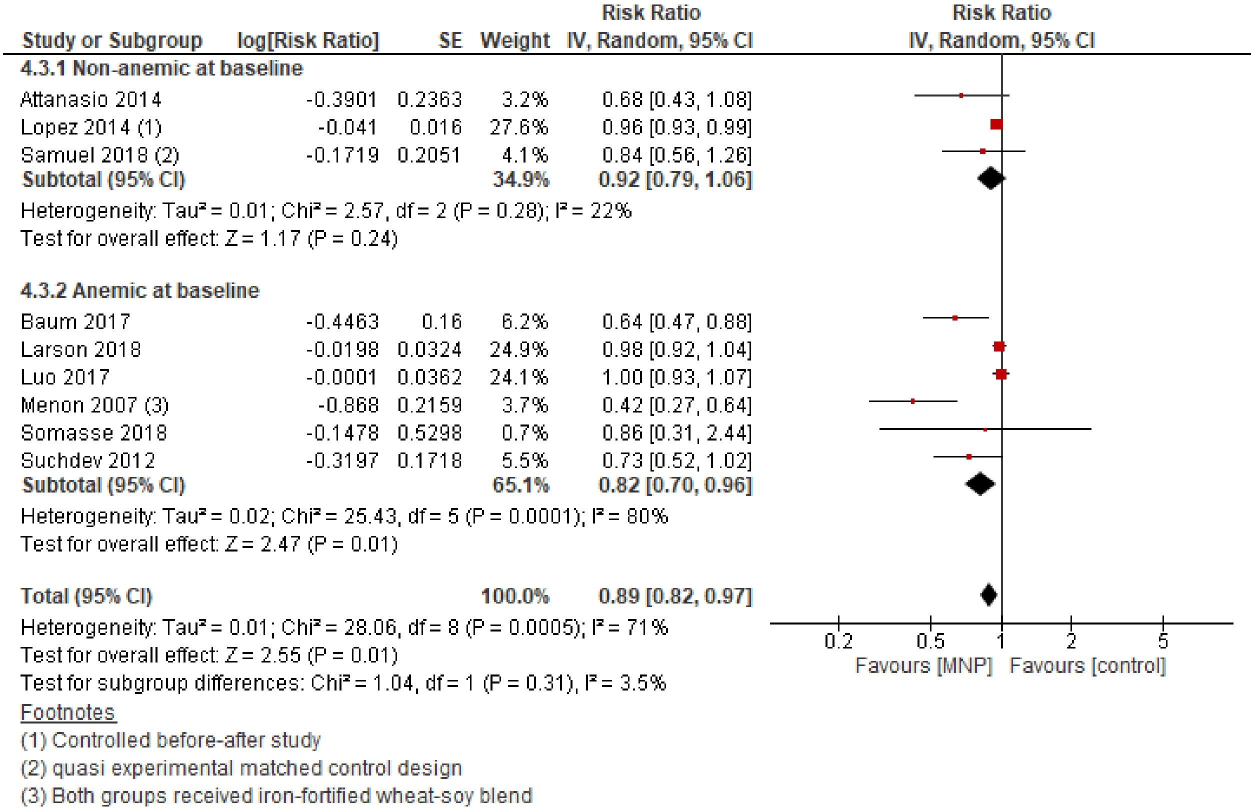

Footnotes
(1) Controlled before-after study
(2) quasi experimental matched control design
(3) Both groups received iron-fortified wheat-soy blend

Comparison 11: Lipid-based Nutrient Supplementation vs. Placebo/No Intervention (Effectiveness)

Stunting

Study or Subgroup	LNS supplementation Events	LNS supplementation Total	Control Events	Control Total	Weight	Risk Ratio M-H, Random, 95% CI
Dewey 2017	303	772	337	802	18.0%	0.93 [0.83, 1.05]
Huybregts 2012	157	341	131	251	12.0%	0.88 [0.75, 1.04]
Luby 2018	457	564	890	1050	33.7%	0.96 [0.91, 1.00]
Null 2018	560	654	1238	1503	35.5%	1.04 [1.00, 1.08]
Rosado 2011	10	59	10	62	0.7%	1.05 [0.47, 2.34]
**Total (95% CI)**		**2390**		**3668**	**100.0%**	**0.97 [0.91, 1.04]**
Total events	1487		2606			
Heterogeneity: Tau² = 0.00; Chi² = 11.94, df = 4 (P = 0.02); I² = 67%						
Test for overall effect: Z = 0.81 (P = 0.42)						

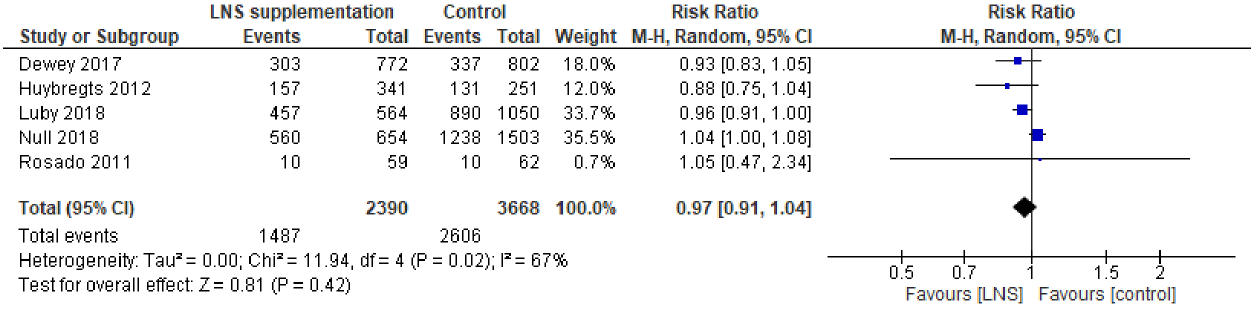

Hemoglobin (g/L)

Study or Subgroup	LNS supplementation Mean	LNS supplementation SD	LNS supplementation Total	Control Mean	Control SD	Control Total	Weight	Mean Difference IV, Random, 95% CI
Batra 2016	-5	2	85	0.03	1	58	26.3%	-5.03 [-5.53, -4.53]
Dewey 2017	115.6	9.1702	151	112.4	10.0835	160	25.4%	3.20 [1.06, 5.34]
Huybregts 2012	105.8	14.3	326	102.5	15.2	233	25.1%	3.30 [0.81, 5.79]
Rosado 2011	6	11	55	4	11	61	23.2%	2.00 [-2.01, 6.01]
**Total (95% CI)**			**617**			**512**	**100.0%**	**0.78 [-4.81, 6.37]**
Heterogeneity: Tau² = 30.89; Chi² = 100.55, df = 3 (P < 0.00001); I² = 97%								
Test for overall effect: Z = 0.27 (P = 0.78)								

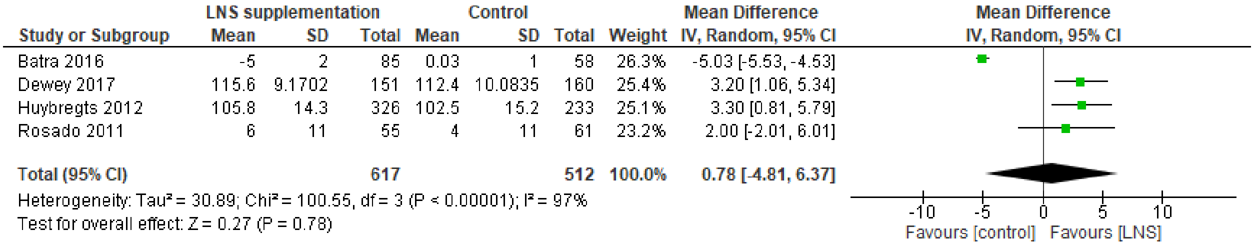

Length-for-age (z-score)

Study or Subgroup	LNS supplementation Mean	SD	Total	Control Mean	SD	Total	Weight	Mean Difference IV, Random, 95% CI
Batra 2016	0.72	0.1	85	0.7	0.1	58	25.0%	0.02 [-0.01, 0.05]
Dewey 2017	-1.73	0.95	772	-1.8	0.94	802	19.9%	0.07 [-0.02, 0.16]
Huybregts 2012	-1.79	1.46	341	-2.06	1.39	251	9.1%	0.27 [0.04, 0.50]
Luby 2018	-1.53	1.05	457	-1.79	1.01	890	17.6%	0.26 [0.14, 0.38]
Null 2018	-1.44	1.11	560	-1.54	1.11	1238	18.2%	0.10 [-0.01, 0.21]
Rosado 2011	0.1	0.6	59	0.1	0.6	62	10.1%	0.00 [-0.21, 0.21]
**Total (95% CI)**			**2274**			**3301**	**100.0%**	**0.11 [0.02, 0.19]**

Heterogeneity: Tau² = 0.01; Chi² = 20.12, df = 5 (P = 0.001); I² = 75%
Test for overall effect: Z = 2.43 (P = 0.02)

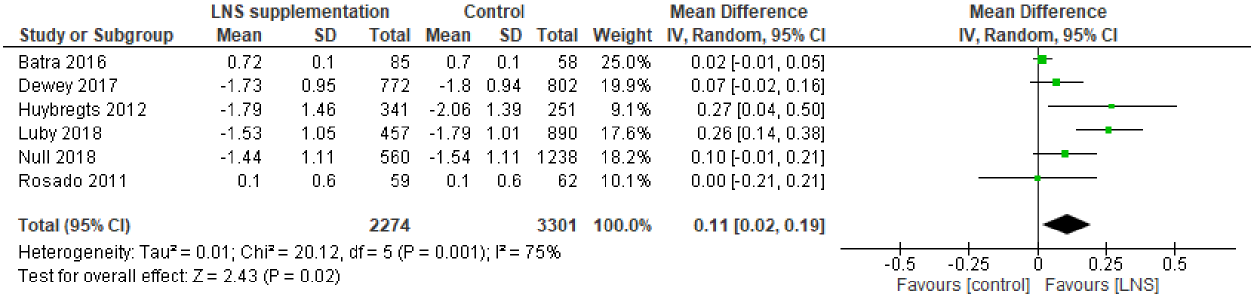

Weight-for-age (z-score)

Study or Subgroup	LNS supplementation Mean	SD	Total	Control Mean	SD	Total	Weight	Mean Difference IV, Random, 95% CI
Batra 2016	0.35	0.1	85	0.24	0.1	58	31.3%	0.11 [0.08, 0.14]
Dewey 2017	-1.65	0.93	772	-1.75	0.93	802	20.3%	0.10 [0.01, 0.19]
Luby 2018	-1.29	1.07	462	-1.54	1	904	16.2%	0.25 [0.13, 0.37]
Null 2018	-0.65	0.68	563	-0.72	1.01	1248	22.7%	0.07 [-0.01, 0.15]
Rosado 2011	0	0.5	56	0.1	0.5	62	9.4%	-0.10 [-0.28, 0.08]
**Total (95% CI)**			**1938**			**3074**	**100.0%**	**0.10 [0.04, 0.17]**

Heterogeneity: Tau² = 0.00; Chi² = 11.64, df = 4 (P = 0.02); I² = 66%
Test for overall effect: Z = 3.06 (P = 0.002)

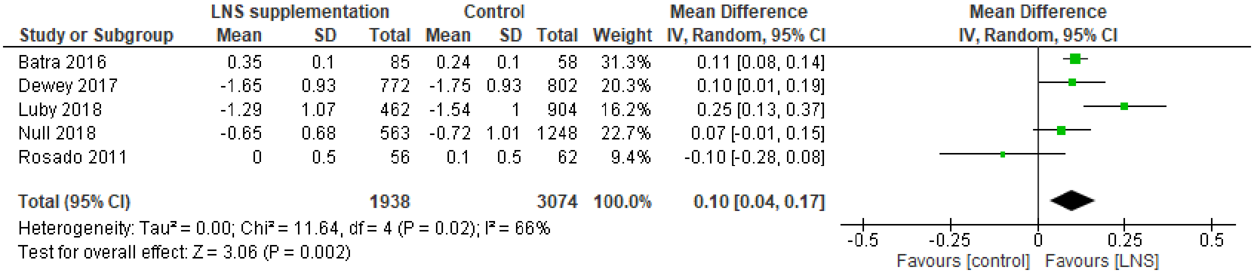

Weight-for-height (z-score)

Study or Subgroup	LNS supplementation Mean	SD	Total	Control Mean	SD	Total	Weight	Mean Difference IV, Random, 95% CI
Batra 2016	-0.16	0.1	82	-0.31	0.1	54	28.4%	0.15 [0.12, 0.18]
Dewey 2017	-1.03	0.87	772	-1.11	0.89	802	19.5%	0.08 [-0.01, 0.17]
Huybregts 2012	-1.05	0.93	341	-1.09	0.95	251	10.9%	0.04 [-0.11, 0.19]
Luby 2018	-0.71	1	457	-0.88	0.93	890	15.9%	0.17 [0.06, 0.28]
Null 2018	0.14	0.92	560	0.11	0.94	1239	18.6%	0.03 [-0.06, 0.12]
Rosado 2011	0	0.6	57	0.1	0.6	62	6.7%	-0.10 [-0.32, 0.12]
**Total (95% CI)**			**2269**			**3298**	**100.0%**	**0.09 [0.03, 0.15]**

Heterogeneity: Tau² = 0.00; Chi² = 13.11, df = 5 (P = 0.02); I² = 62%
Test for overall effect: Z = 2.75 (P = 0.006)

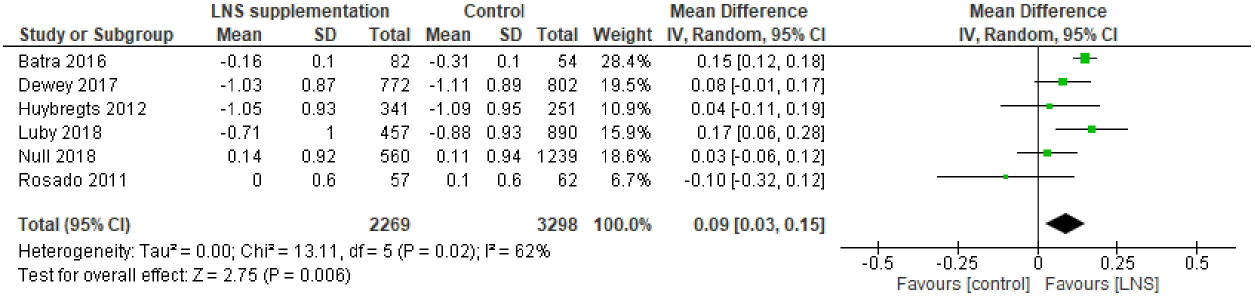

Supplement: Supplementary file 1 [file nutrients-12-00289-s001.pdf]
